# Supplementary material for: Genomic analysis defines clonal relationships of ductal carcinoma in situ and recurrent invasive breast cancer
Source: Nat Genet. 2022 Jun 9;54(6):850–60. doi: 10.1038/s41588-022-01082-3 (PMC9197769; doi:10.1038/s41588-022-01082-3)

nature portfolio

https://doi.org/10.1038/s41588-022-01082-3

Supplementary information

Genomic analysis defines clonal relationships of ductal carcinoma in situ and recurrent invasive breast cancer

In the format provided by the authors and unedited

Supplementary Figure 1: Individual copy number profiles

All the individual copy number profiles are shown for each primary DCIS and recurrent pairs and synchronous DCIS-invasive pairs.

For SNP-array processed samples, segmented LRR and BAF plots for the complete genome generated by ASCAT are shown - original log-ratio of microarray intensities and BAF values in black and segmented values in green.

For sequenced samples, segmented log2 ratio plots for the complete genome generated by QDNAseq are shown - original log2 ratio in black and segmented log2 ratios in orange.

The genomic position is indicated by chromosome 1 on the left and up to chromosome X on the right in all the graphs.

The following metadata for each pair of samples is shown for each pair above the copy number plot: synchronous or metachronous tumor, time from first surgery to second event in months, side of second event, histology of second event, surgery, treatment of primary disease - RT (radiotherapy) or HT (hormone therapy), ER and Her2 status of primary disease and second event, grade of primary disease and second event, quadrant of second event, margins and screening. P-values of clonality algorithm are also shown for all the platforms available for each pair of samples and final verdict

P001

Syn/Meta	Time from 1st surgery to 2nd event (Months)	Side 2nd event	Histology 2nd event	Surgery	AdjuvanT Treatment Pri (RT/ HT)	ER Pri	ER 2nd event	Her2 Pri	Her2 2nd event	Grade Pri	Grade 2nd event	Quadrant 2nd event	Margins	Screening	Clonality P value Copy N	Clonality P value Panel seq	Clonality P value WES	Final verdict
metachronous	63	Ipsilateral	DCIS only	lumpectomy	RT	-	NA	+	+	3	NA	NA	Clear	screen-detected	0.000466	0.05	0.00075244	Related

Primary event

LogR

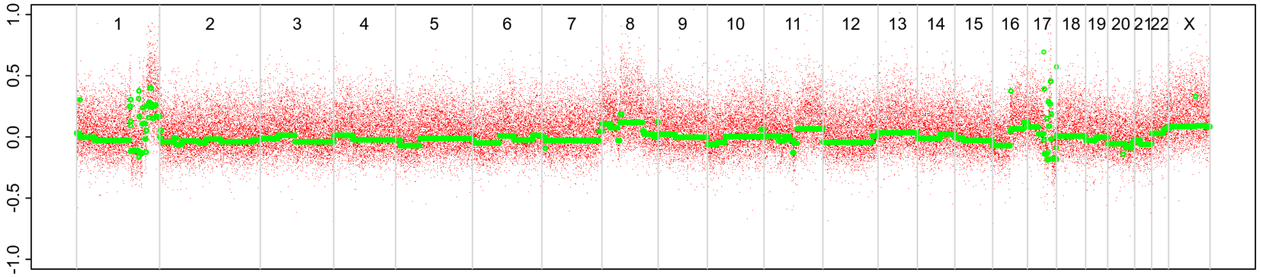

BAF

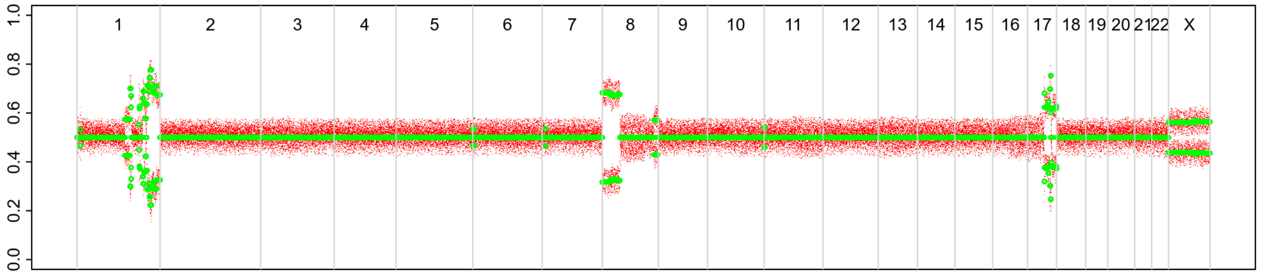

2nd event

LogR

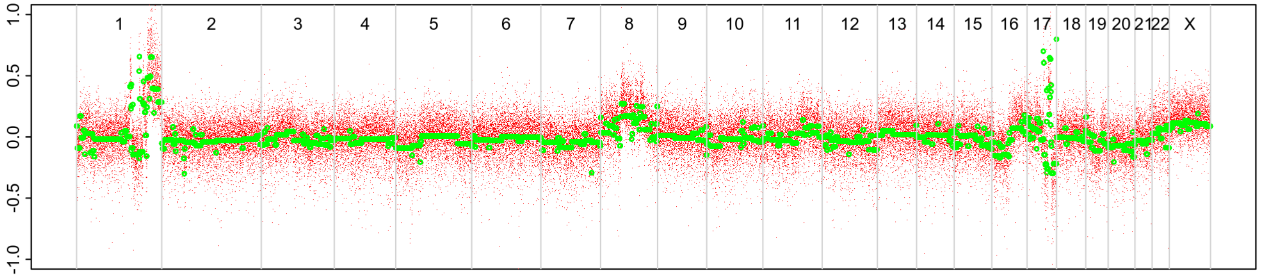

BAF

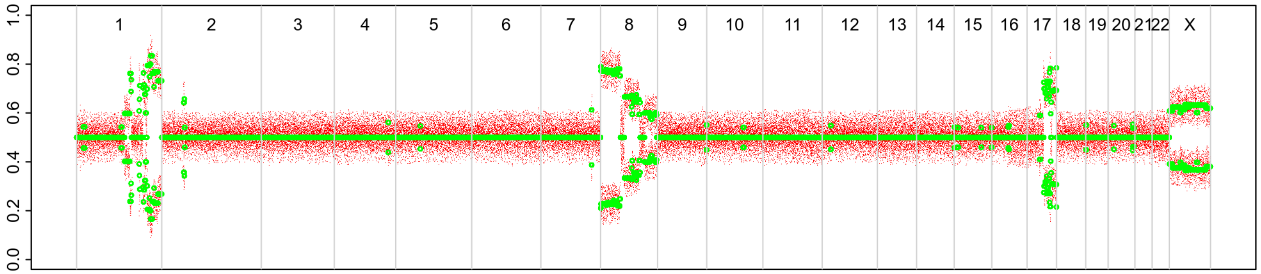

P002

Syn/Meta	Time from 1st surgery to 2nd event (Months)	Side 2nd event	Histology 2nd event	Surgery	AdjuvanT Treatment Pri (RT/ HT)	ER Pri	ER 2nd event	Her2 Pri	Her2 2nd event	Grade Pri	Grade 2nd event	Quadrant 2nd event	Margins	Screening	Clonality P value Copy N	Clonality P value Panel seq	Clonality P value WES	Final verdict
metachronous	72	Ipsilateral	DCIS only	lumpectomy	None	NA	NA	NA	NA	3	3	Unknown	Clear	screen-detected	0.003663	NA	NA	Related

Primary event

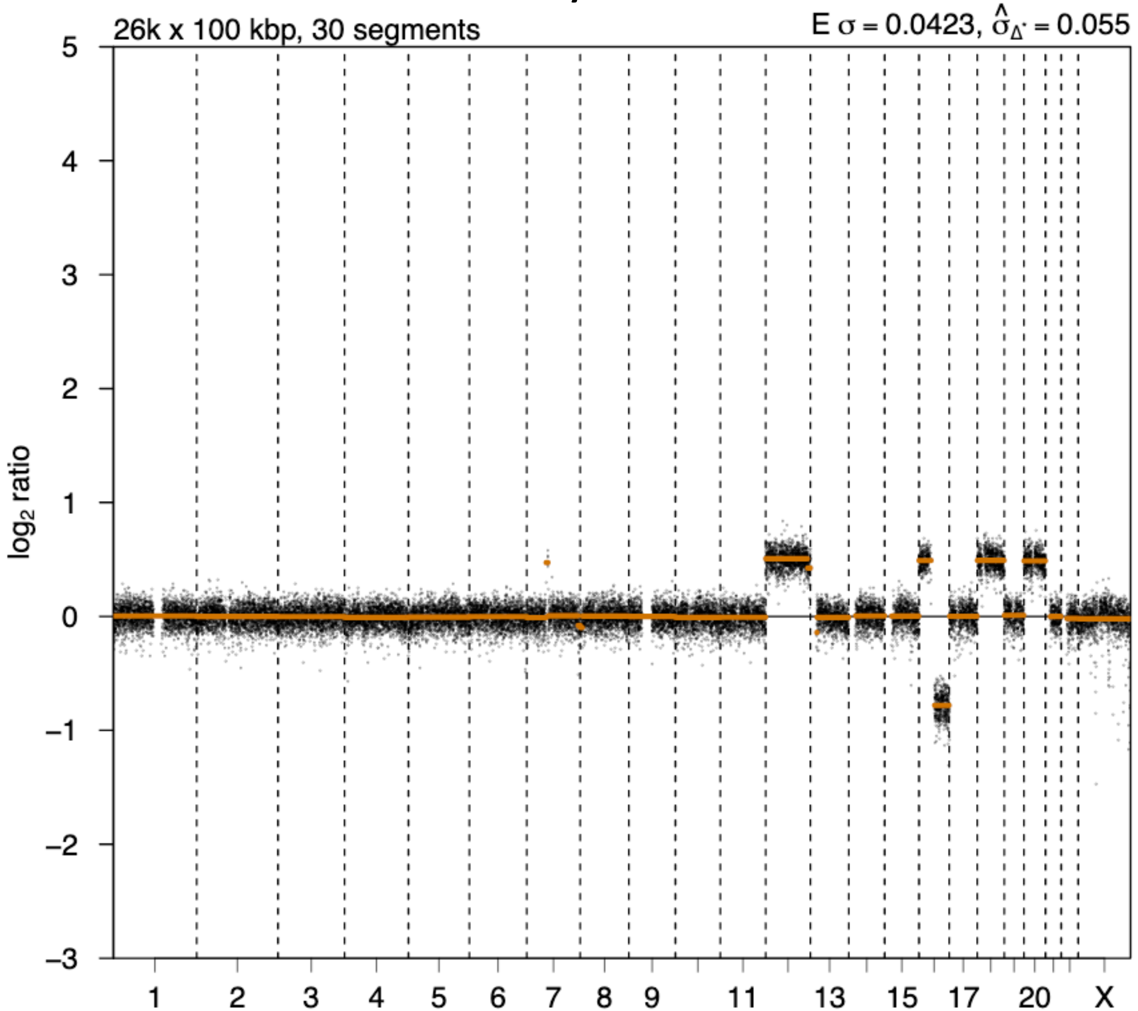

2nd event

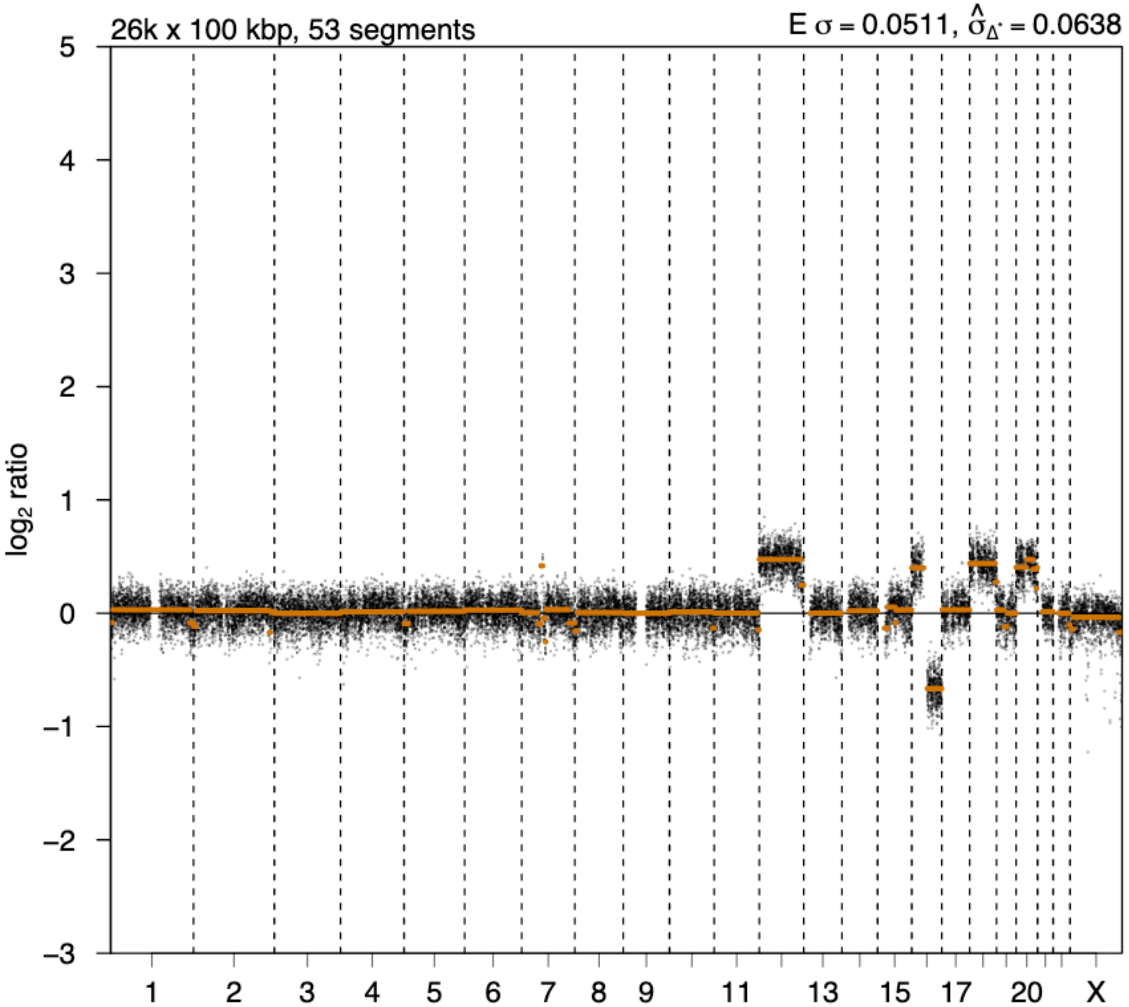

P004

Syn/Meta	Time from 1st surgery to 2nd event (Months)	Side 2nd event	Histology 2nd event	Surgery	AdjuvanT Treatment Pri (RT/ HT)	ER Pri	ER 2nd event	Her2 Pri	Her2 2nd event	Grade Pri	Grade 2nd event	Quadrant 2nd event	Margins	Screening	Clonality P value Copy N	Clonality P value Panel seq	Clonality P value WES	Final verdict
metachronous	37	Ipsilateral	DCIS only	lumpectomy	RT	+	NA	-	NA	2	NA	NA	Clear	screen-detected	0.002331	NA	NA	Related

Primary event

LogR

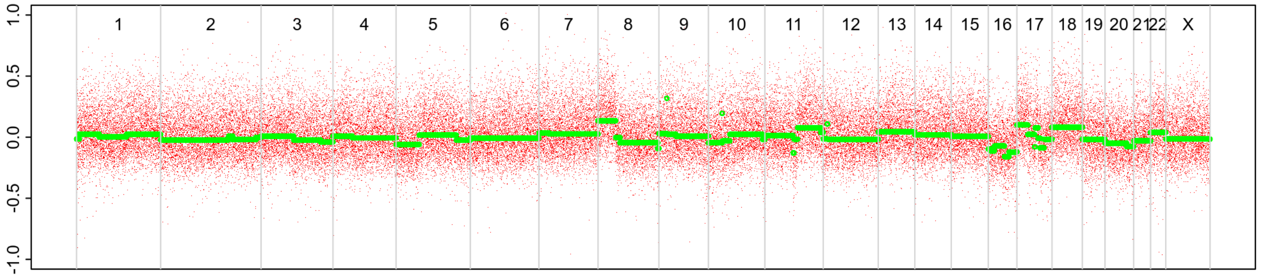

BAF

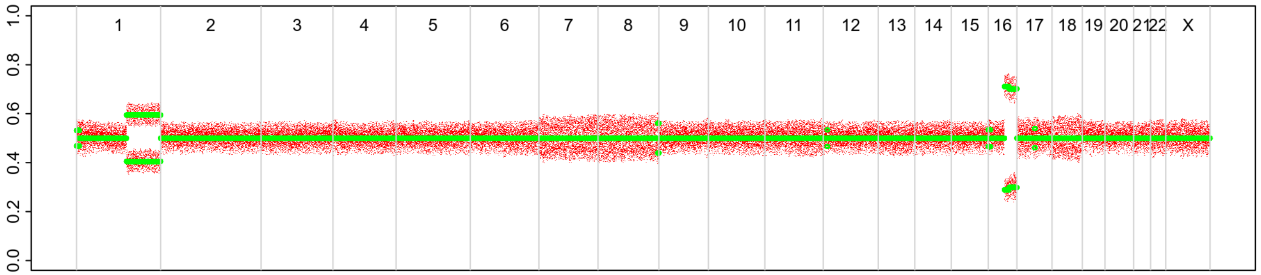

2nd event

LogR

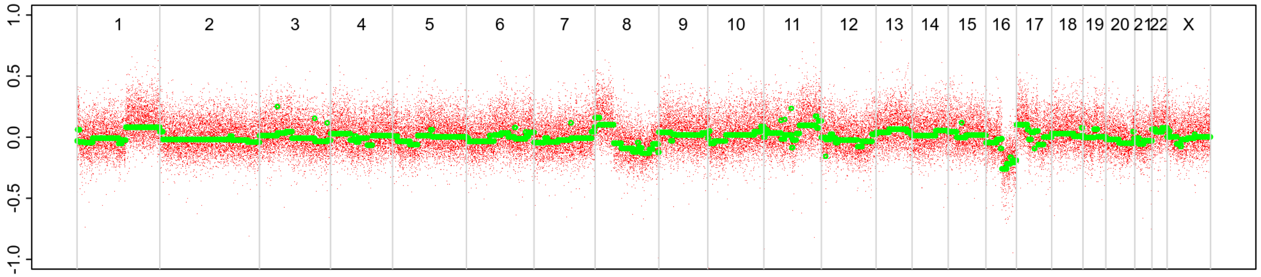

BAF

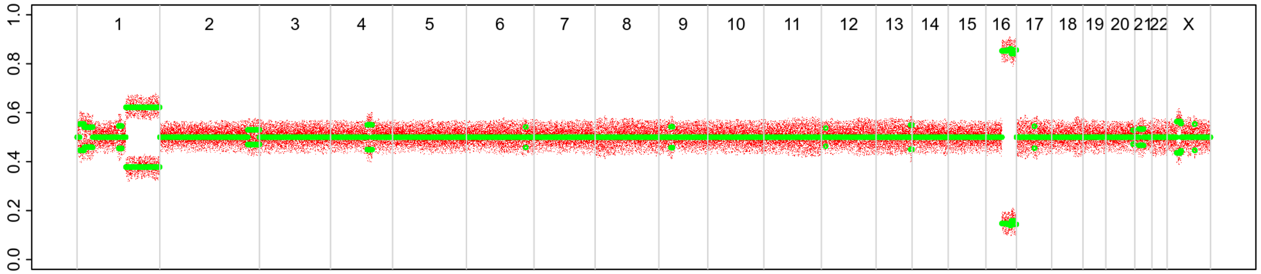

P005

Syn/Meta	Time from 1st surgery to 2nd event (Months)	Side 2nd event	Histology 2nd event	Surgery	AdjuvanT Treatment Pri (RT/ HT)	ER Pri	ER 2nd event	Her2 Pri	Her2 2nd event	Grade Pri	Grade 2nd event	Quadrant 2nd event	Margins	Screening	Clonality P value Copy N	Clonality P value Panel seq	Clonality P value WES	Final verdict
metachronous	35	Ipsilateral	DCIS only	lumpectomy	RT	NA	NA	+	NA	3	3	NA	Clear	screen-detected	0.003663	NA	NA	Related

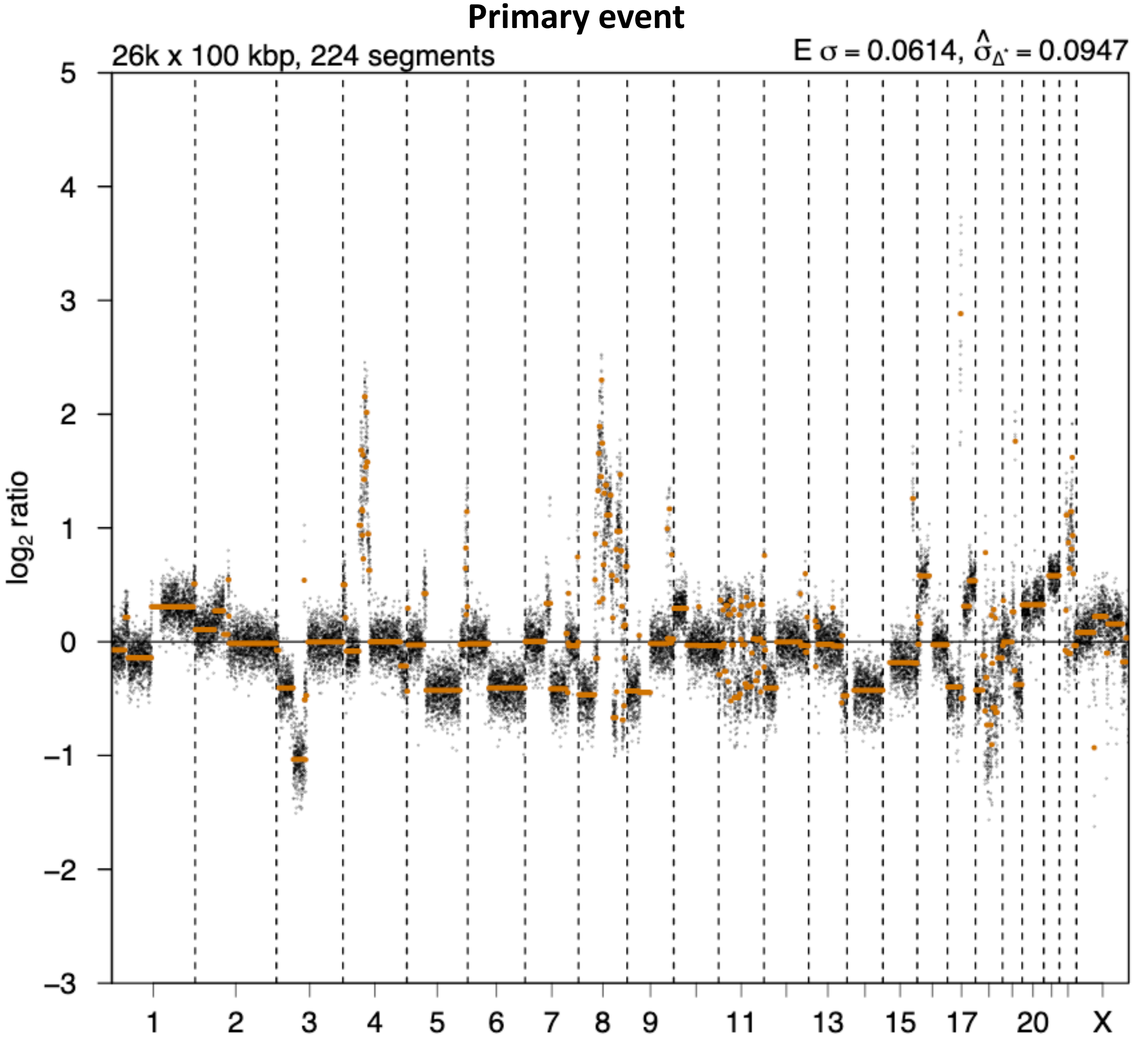

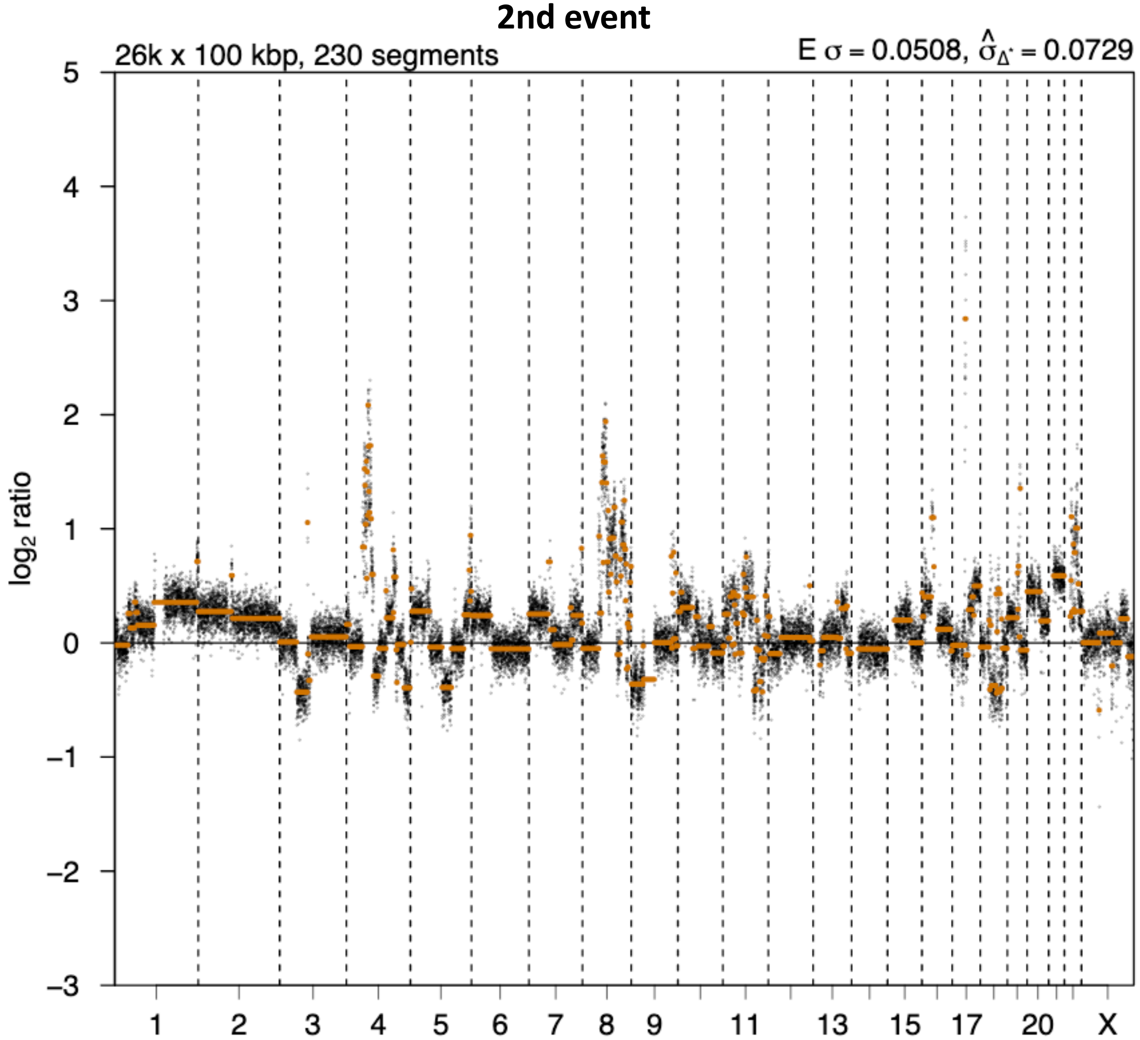

P006

Syn/Meta	Time from 1st surgery to 2nd event (Months)	Side 2nd event	Histology 2nd event	Surgery	AdjuvanT Treatment Pri (RT/ HT)	ER Pri	ER 2nd event	Her2 Pri	Her2 2nd event	Grade Pri	Grade 2nd event	Quadrant 2nd event	Margins	Screening	Clonality P value Copy N	Clonality P value Panel seq	Clonality P value WES	Final verdict
metachronous	46	Ipsilateral	DCIS only	lumpectomy	RTHT	+	NA	NA	NA	3	3	NA	<2mm	screen-detected	0.000466	0	0.00075244	Related

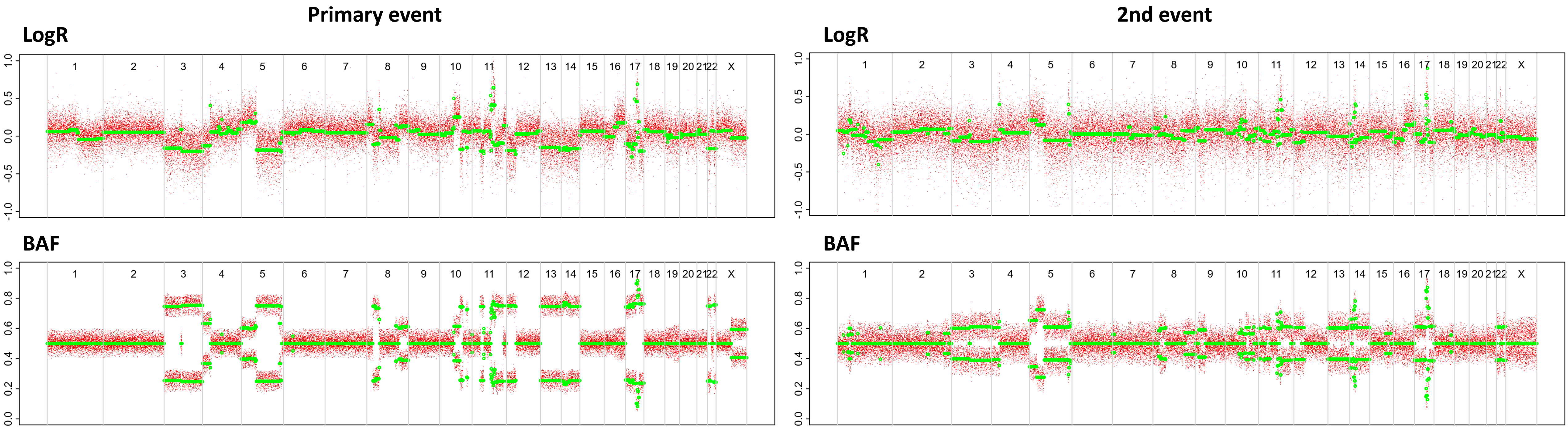

P008

Syn/Meta	Time from 1st surgery to 2nd event (Months)	Side 2nd event	Histology 2nd event	Surgery	AdjuvanT Treatment Pri (RT/ HT)	ER Pri	ER 2nd event	Her2 Pri	Her2 2nd event	Grade Pri	Grade 2nd event	Quadrant 2nd event	Margins	Screening	Clonality P value Copy N	Clonality P value Panel seq	Clonality P value WES	Final verdict
metachronous	48	Ipsilateral	DCIS only	lumpectomy	HT	+	+	-	NA	3	3	NA	NA	screen-detected	0.003663	NA	NA	Related

Primary event

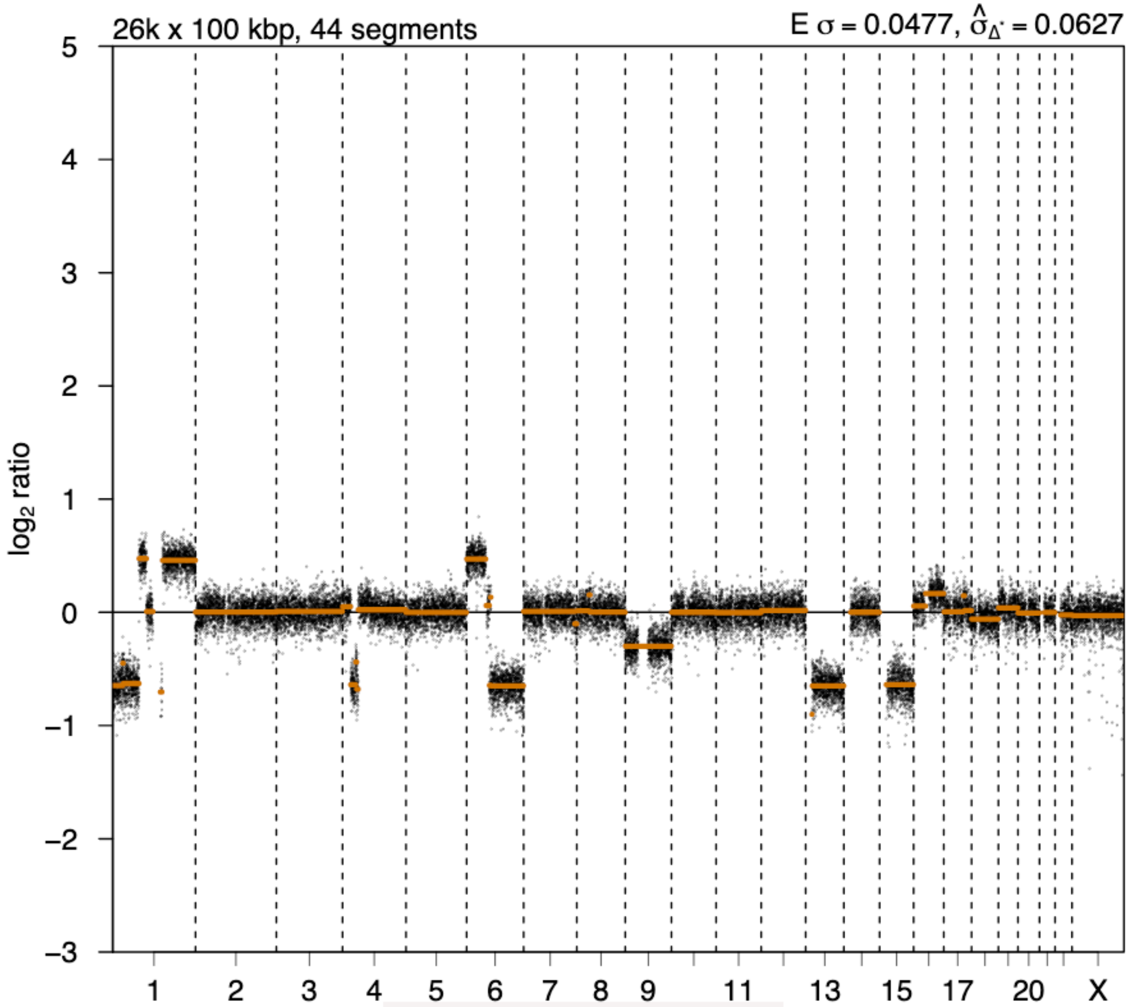

2nd event

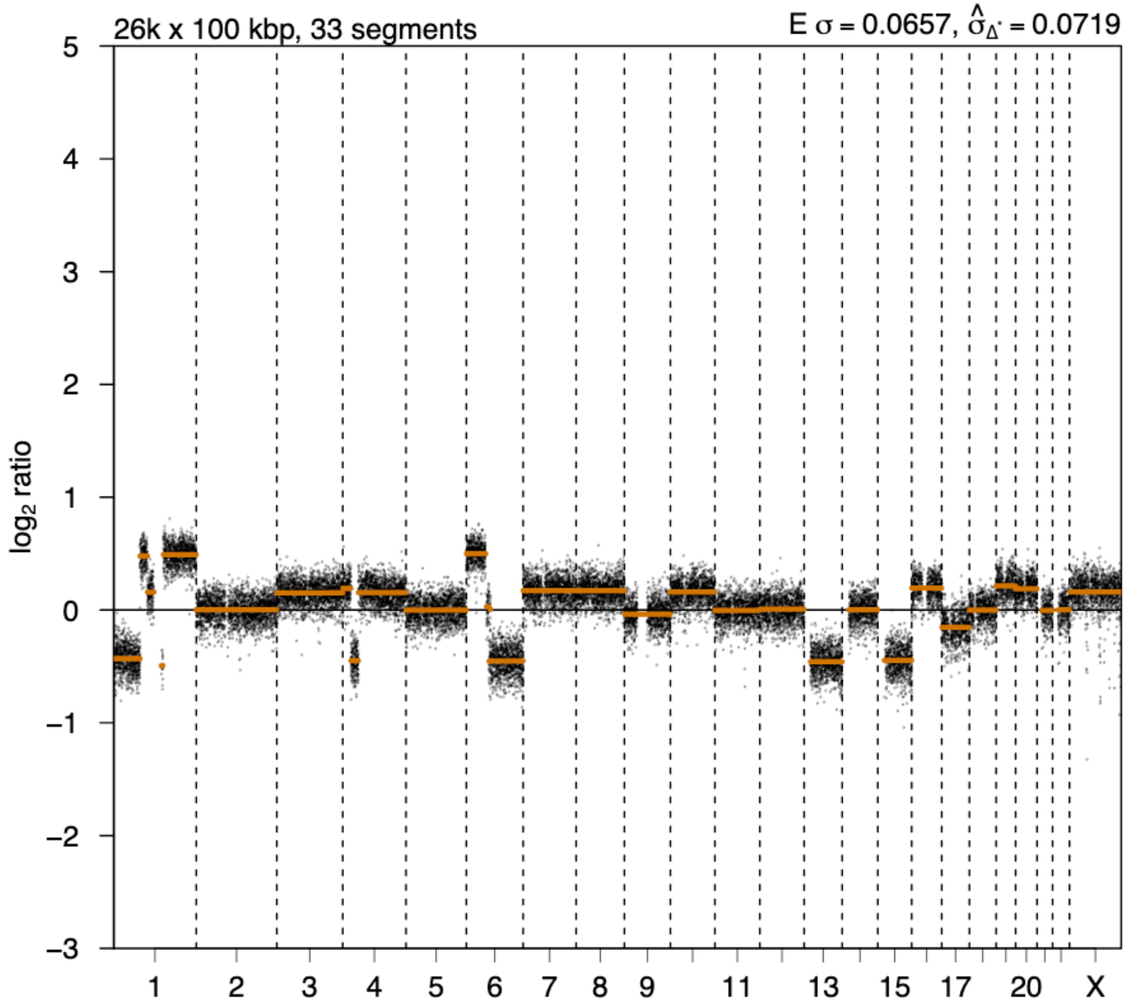

P010

Syn/Meta	Time from 1st surgery to 2nd event (Months)	Side 2nd event	Histology 2nd event	Surgery	AdjuvanT Treatment Pri (RT/ HT)	ER Pri	ER 2nd event	Her2 Pri	Her2 2nd event	Grade Pri	Grade 2nd event	Quadrant 2nd event	Margins	Screening	Clonality P value Copy N	Clonality P value Panel seq	Clonality P value WES	Final verdict
metachronous	11	Ipsilateral	DCIS only	lumpectomy	RT	+	-	NA	NA	2	3	NA	Clear	screen-detected	0.003663	NA	NA	Related

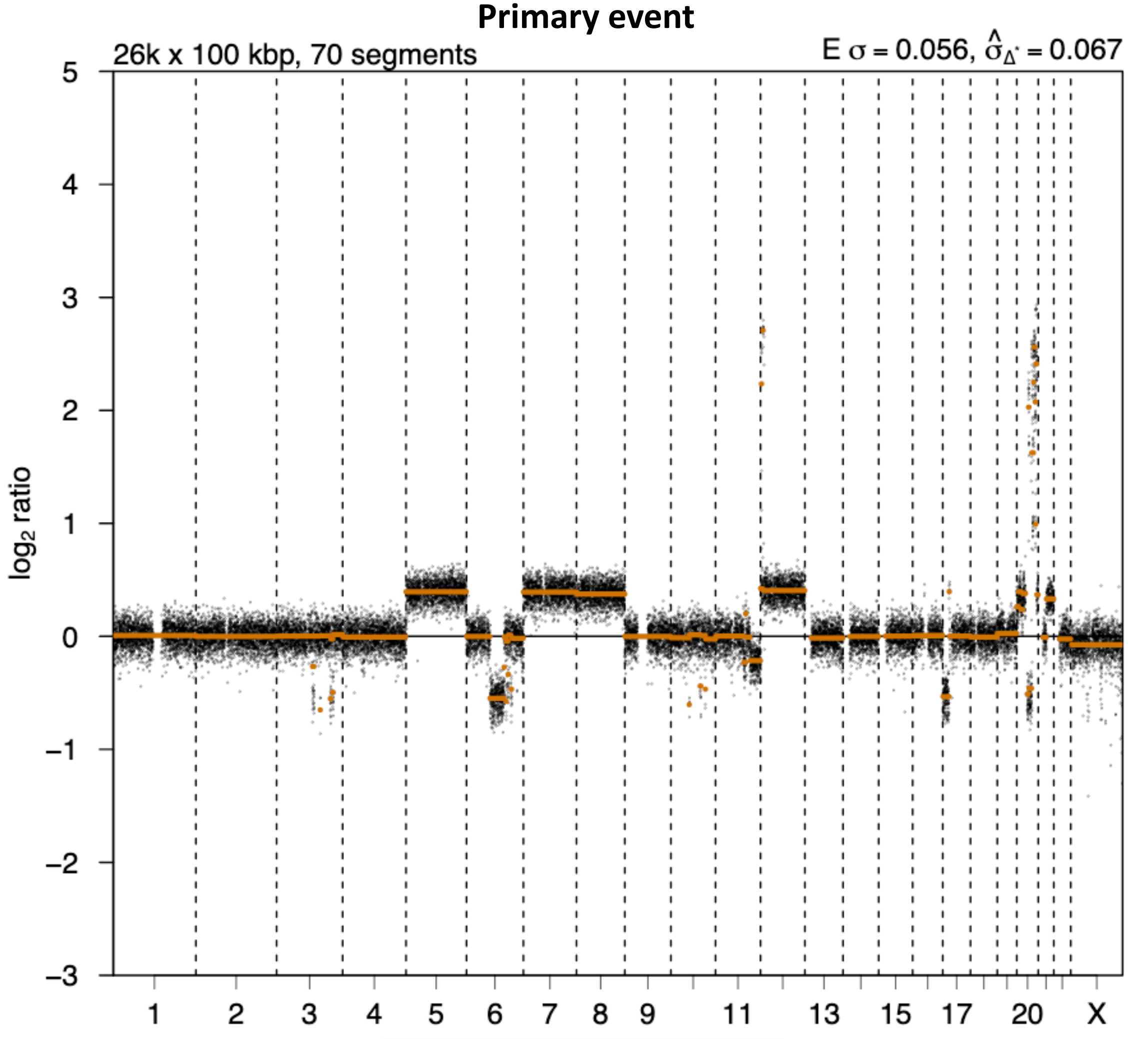

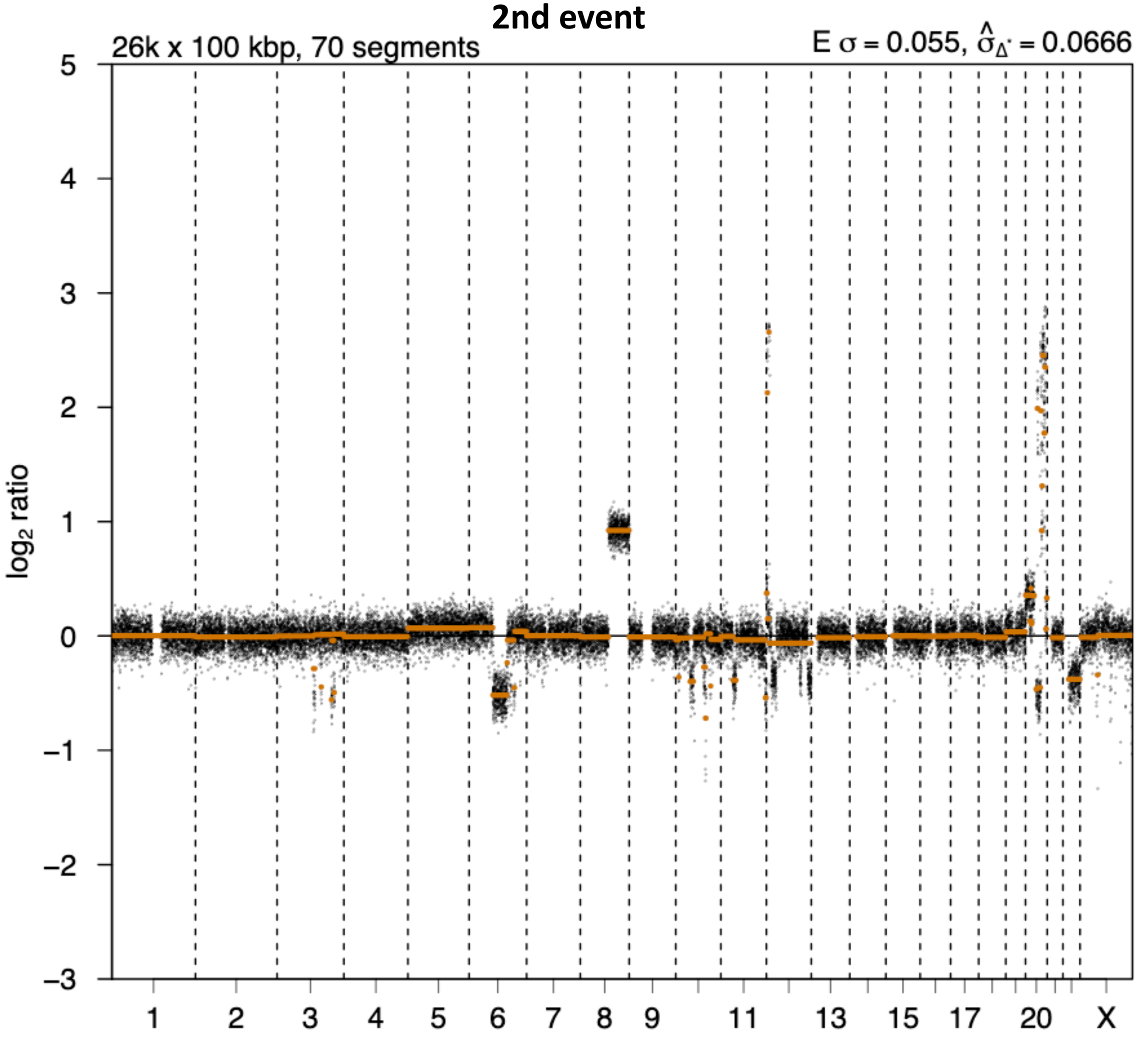

P011

Syn/Meta	Time from 1st surgery to 2nd event (Months)	Side 2nd event	Histology 2nd event	Surgery	AdjuvanT Treatment Pri (RT/ HT)	ER Pri	ER 2nd event	Her2 Pri	Her2 2nd event	Grade Pri	Grade 2nd event	Quadrant 2nd event	Margins	Screening	Clonality P value Copy N	Clonality P value Panel seq	Clonality P value WES	Final verdict
metachronous	25	Ipsilateral	DCIS only	lumpectomy	RT	-	-	+	NA	3	3	NA	Clear	screen-detected	0.003663	NA	NA	Related

Primary event

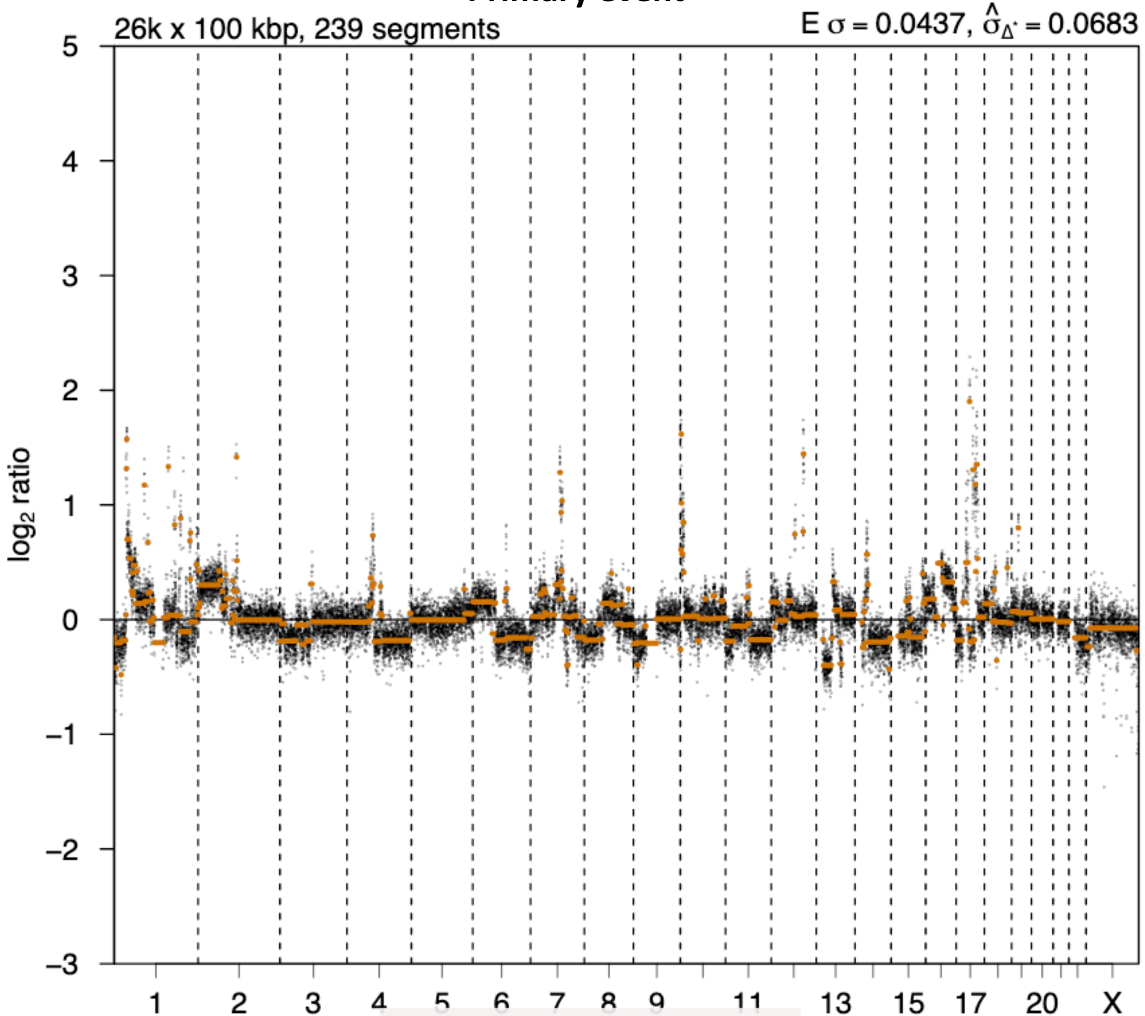

2nd event

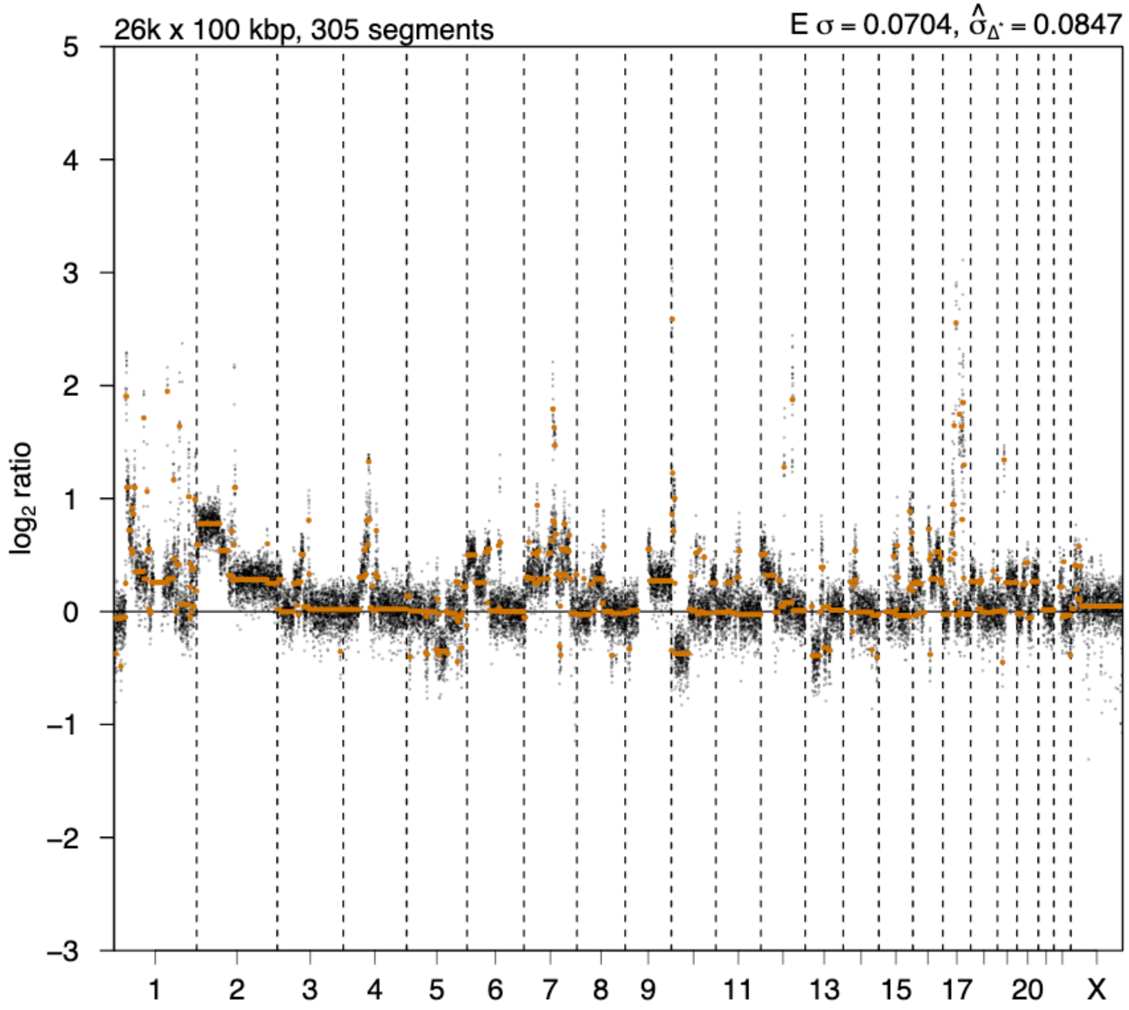

P012

Syn/Meta	Time from 1st surgery to 2nd event (Months)	Side 2nd event	Histology 2nd event	Surgery	AdjuvanT Treatment Pri (RT/ HT)	ER Pri	ER 2nd event	Her2 Pri	Her2 2nd event	Grade Pri	Grade 2nd event	Quadrant 2nd event	Margins	Screening	Clonality P value Copy N	Clonality P value Panel seq	Clonality P value WES	Final verdict
metachronous	24	Ipsilateral	DCIS only	lumpectomy	RT	-	NA	+	NA	3	3	NA	<2mm	screen-detected	0.003663004	NA	NA	Related

Primary event

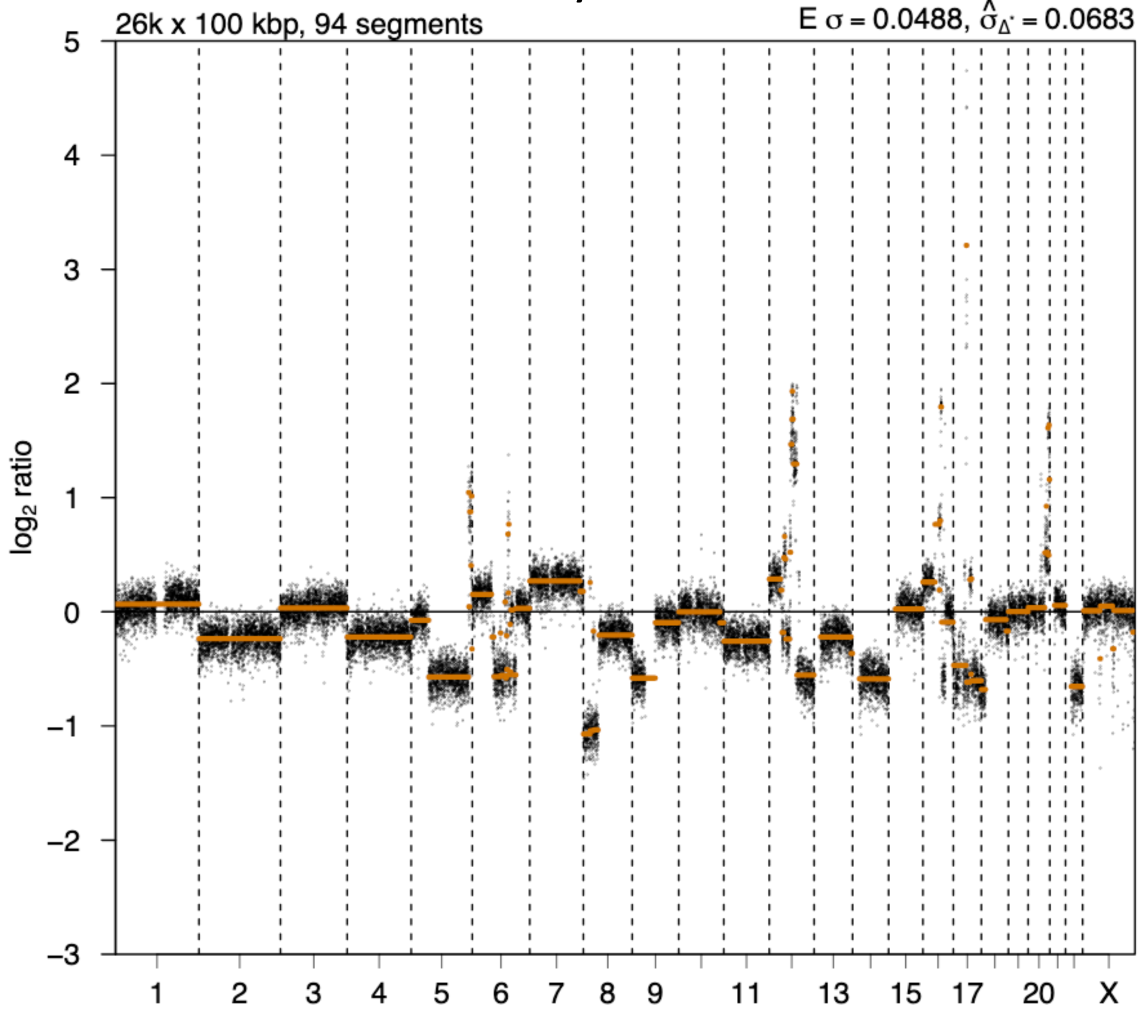

2nd event

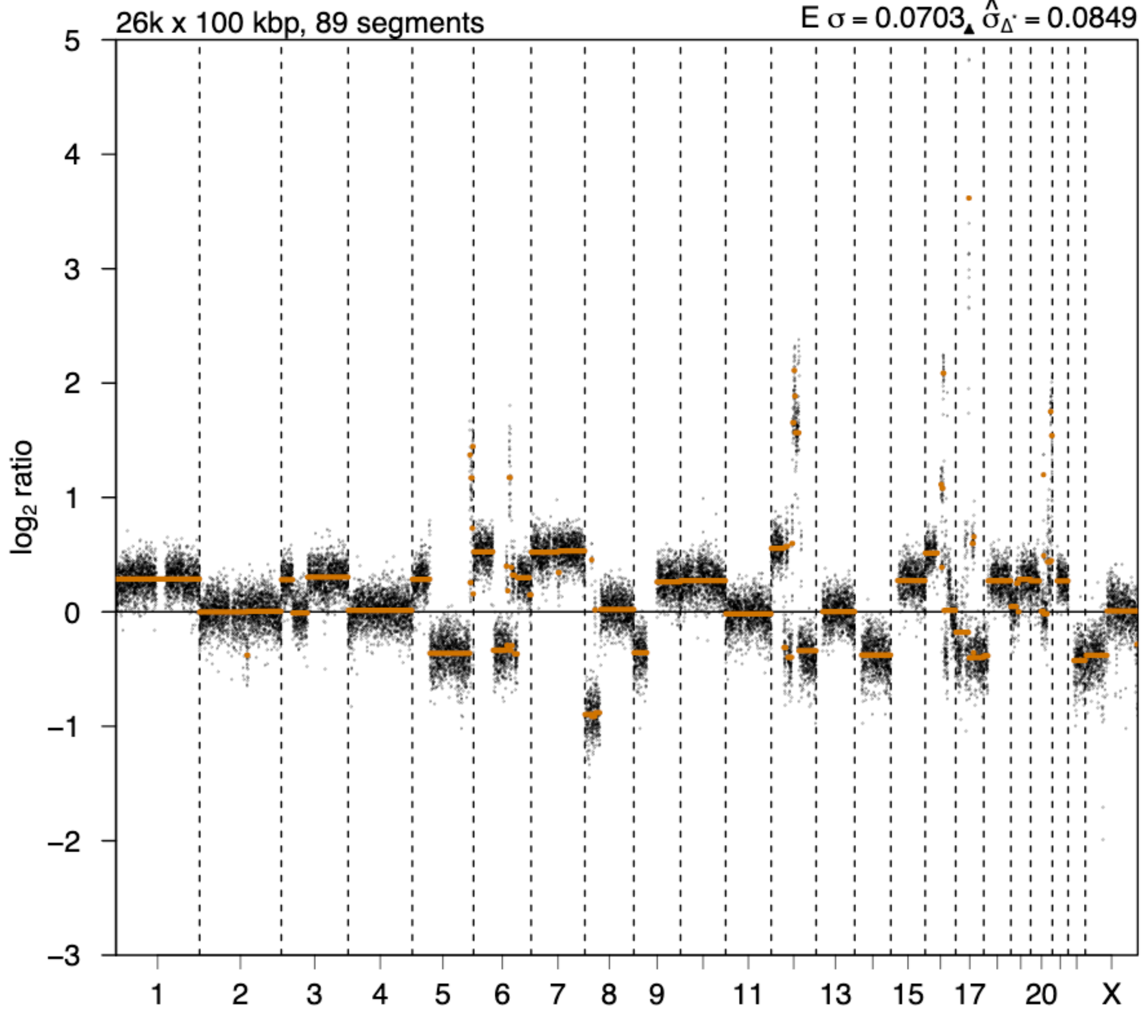

P014

Syn/Meta	Time from 1st surgery to 2nd event (Months)	Side 2nd event	Histology 2nd event	Surgery	AdjuvanT Treatment Pri (RT/ HT)	ER Pri	ER 2nd event	Her2 Pri	Her2 2nd event	Grade Pri	Grade 2nd event	Quadrant 2nd event	Margins	Screening	Clonality P value Copy N	Clonality P value Panel seq	Clonality P value WES	Final verdict
metachronous	20	Ipsilateral	DCIS only	lumpectomy	None	+	NA	NA	NA	3	3	NA	Clear	screen-detected	0.000466	0.16	NA	Related

Primary event

LogR

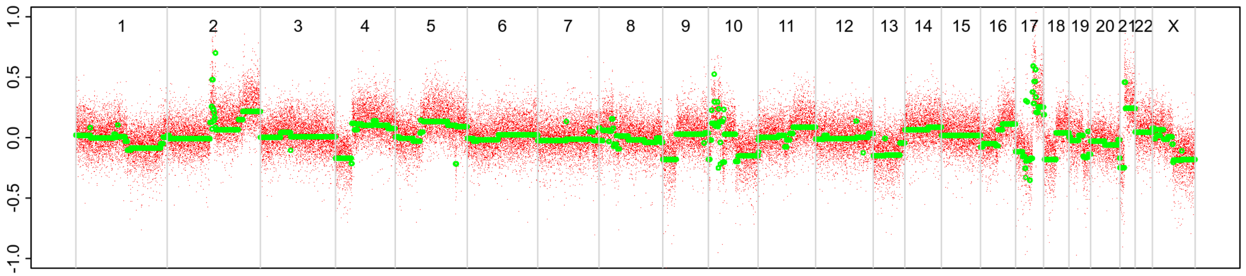

BAF

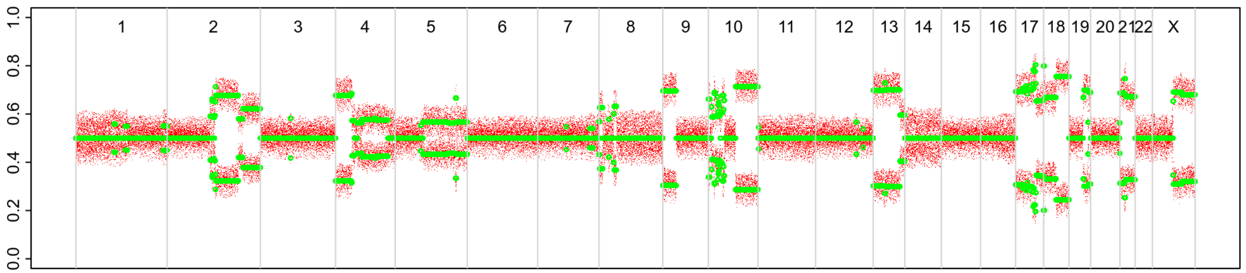

2nd event

LogR

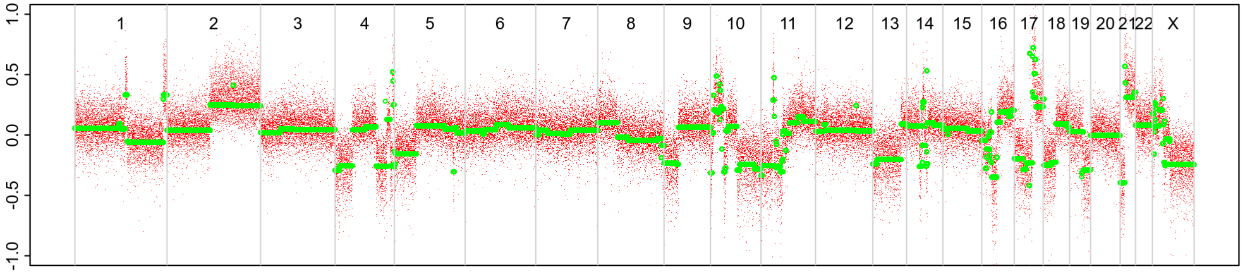

BAF

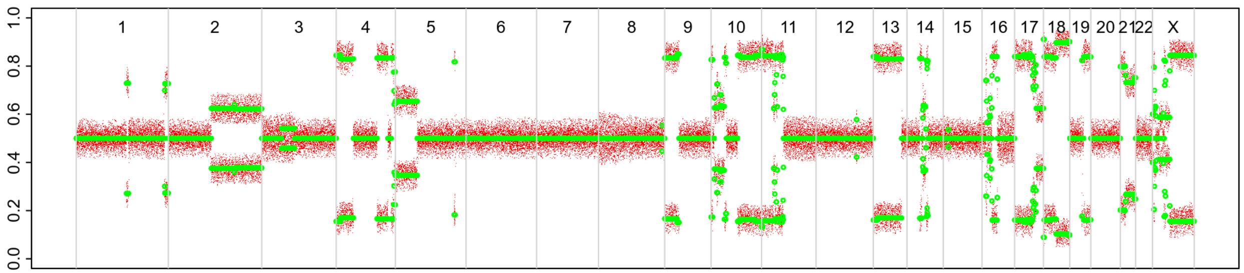

P015

Syn/Meta	Time from 1st surgery to 2nd event (Months)	Side 2nd event	Histology 2nd event	Surgery	AdjuvanT Treatment Pri (RT/ HT)	ER Pri	ER 2nd event	Her2 Pri	Her2 2nd event	Grade Pri	Grade 2nd event	Quadrant 2nd event	Margins	Screening	Clonality P value Copy N	Clonality P value Panel seq	Clonality P value WES	Final verdict
metachronous	13	Ipsilateral	DCIS only	lumpectomy	None	+	-	NA	NA	3	2	NA	Clear	screen-detected	0.003663004	NA	0.000752445	Related

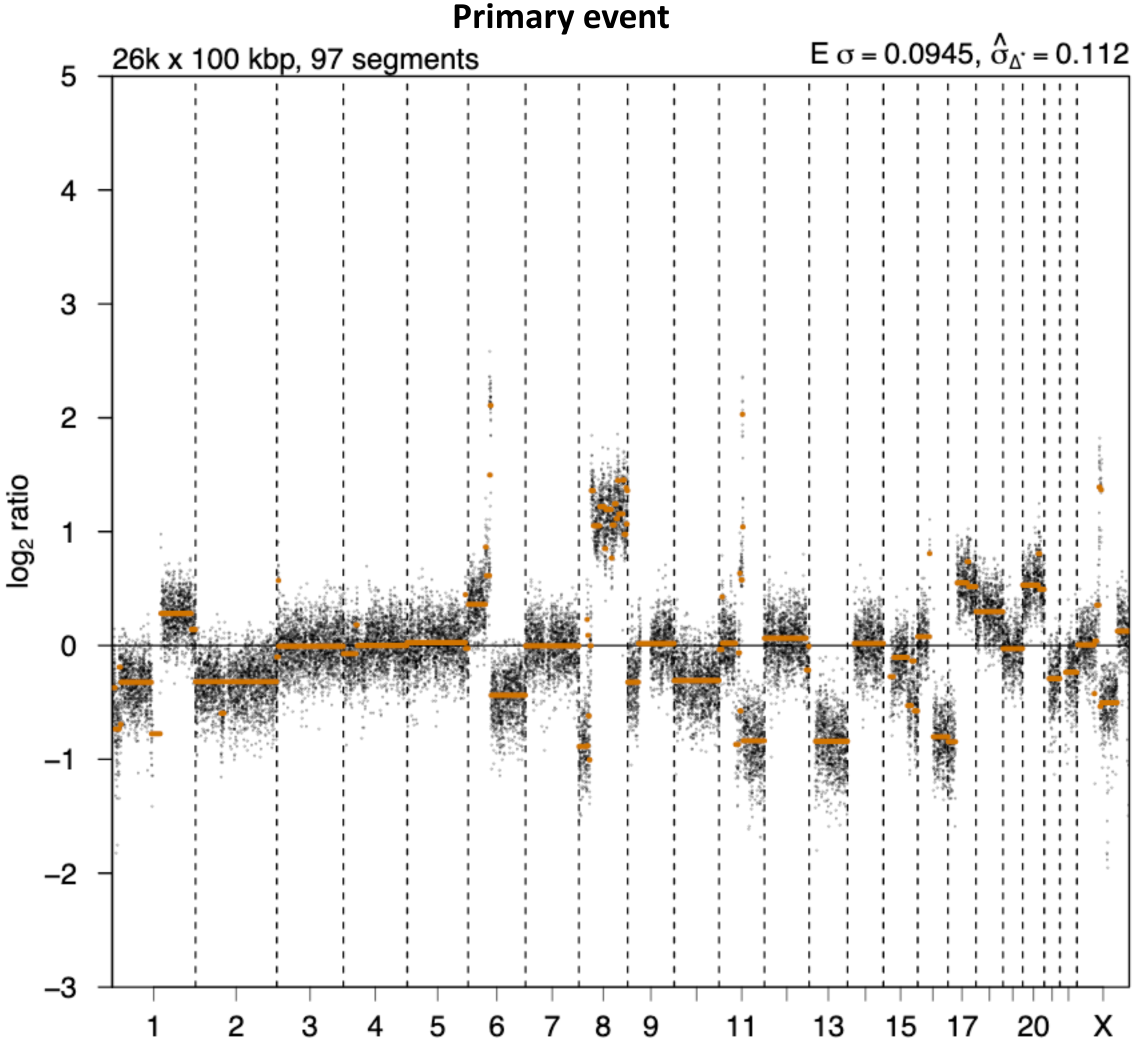

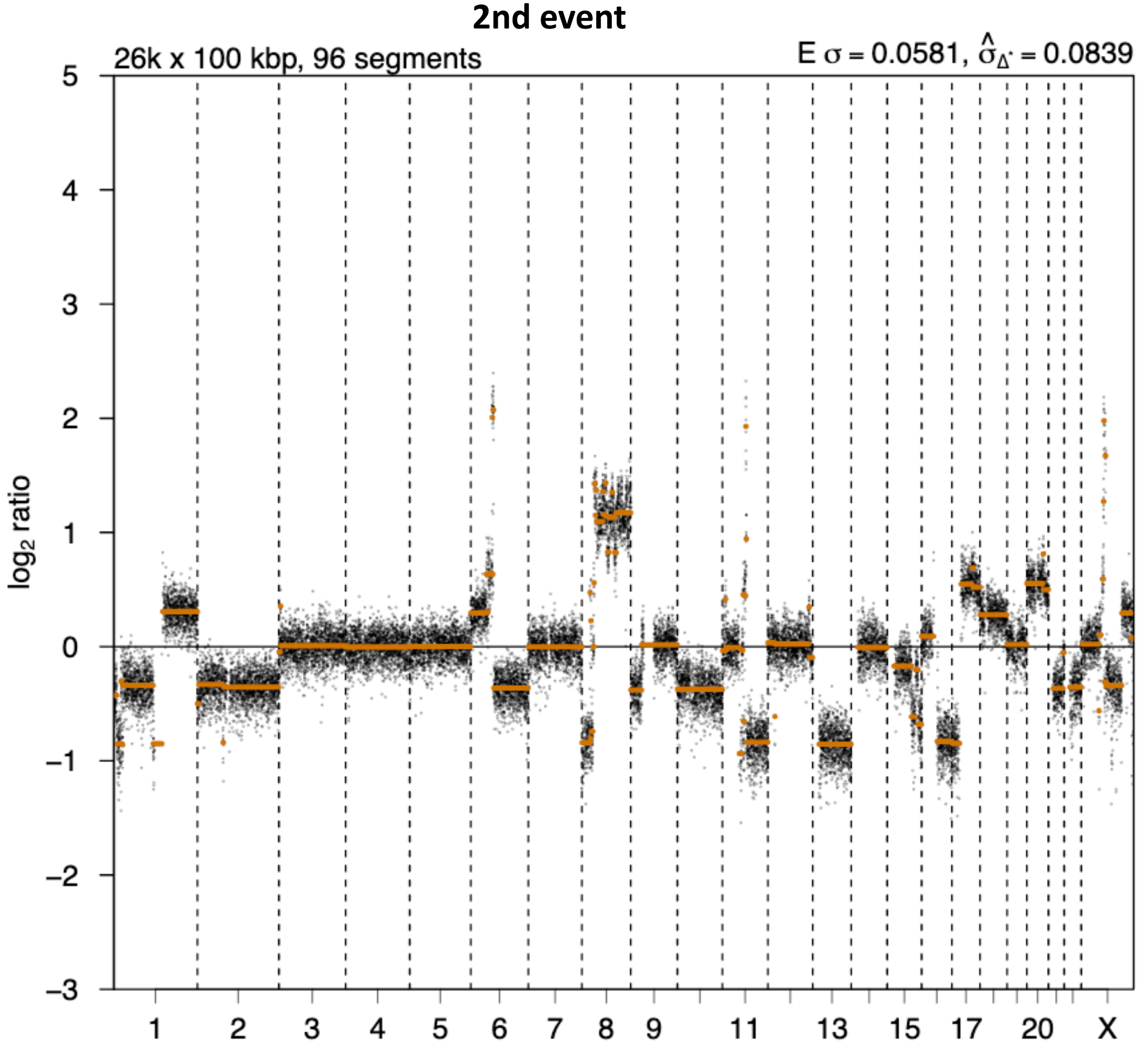

P016

Syn/Meta	Time from 1st surgery to 2nd event (Months)	Side 2nd event	Histology 2nd event	Surgery	AdjuvanT Treatment Pri (RT/ HT)	ER Pri	ER 2nd event	Her2 Pri	Her2 2nd event	Grade Pri	Grade 2nd event	Quadrant 2nd event	Margins	Screening	Clonality P value Copy N	Clonality P value Panel seq	Clonality P value WES	Final verdict
metachronous	20	Ipsilateral	DCIS only	lumpectomy	RT	+	+	NA	NA	3	3	NA	Clear	screen-detected	0.003663 004	NA	NA	Related

Primary event

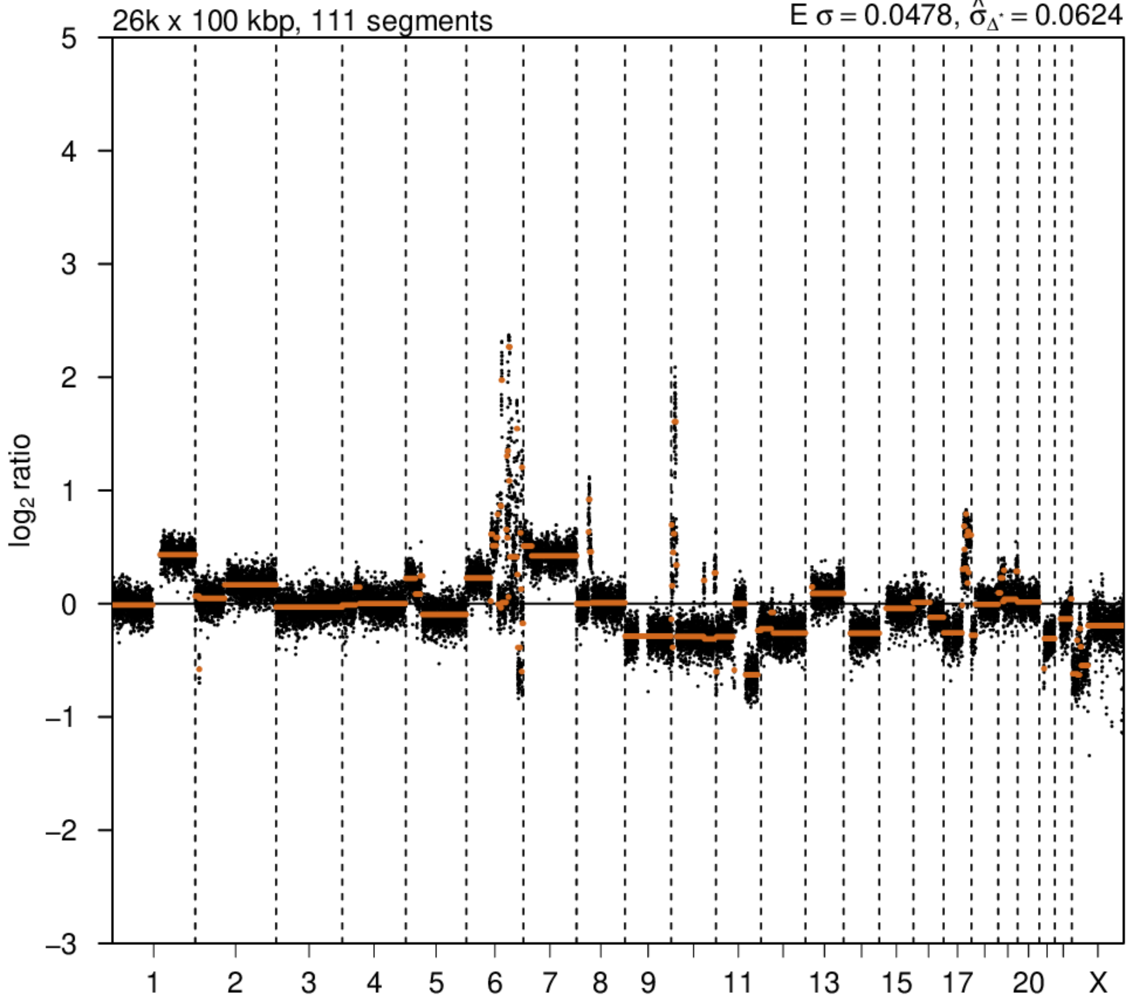

2nd event

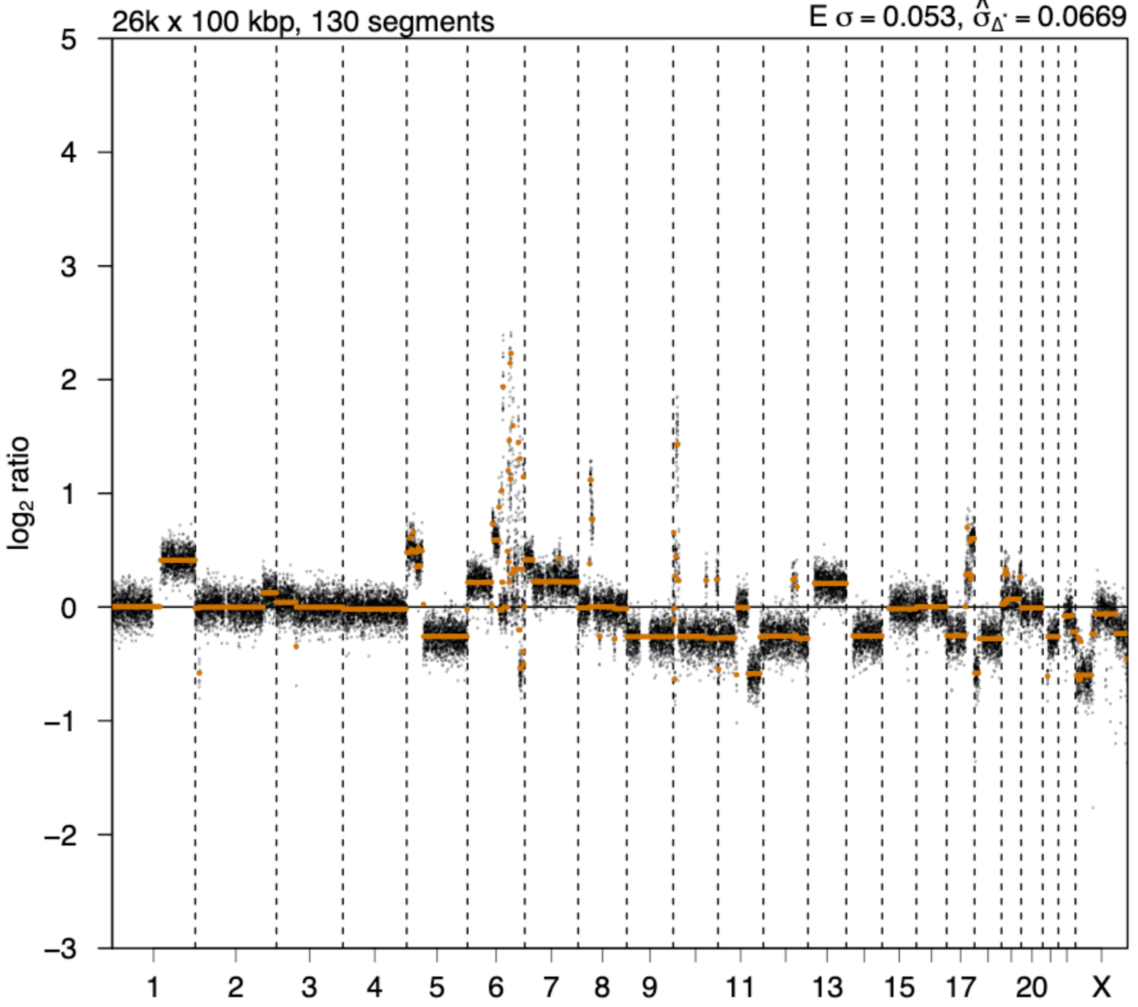

P018

Syn/Meta	Time from 1st surgery to 2nd event (Months)	Side 2nd event	Histology 2nd event	Surgery	AdjuvanT Treatment Pri (RT/ HT)	ER Pri	ER 2nd event	Her2 Pri	Her2 2nd event	Grade Pri	Grade 2nd event	Quadrant 2nd event	Margins	Screening	Clonality P value Copy N	Clonality P value Panel seq	Clonality P value WES	Final verdict
metachronous	24	Ipsilateral	DCIS only	lumpectomy	None	NA	NA	NA	NA	3	3	NA	NA	screen-detected	0.003663004	NA	NA	Related

Primary event

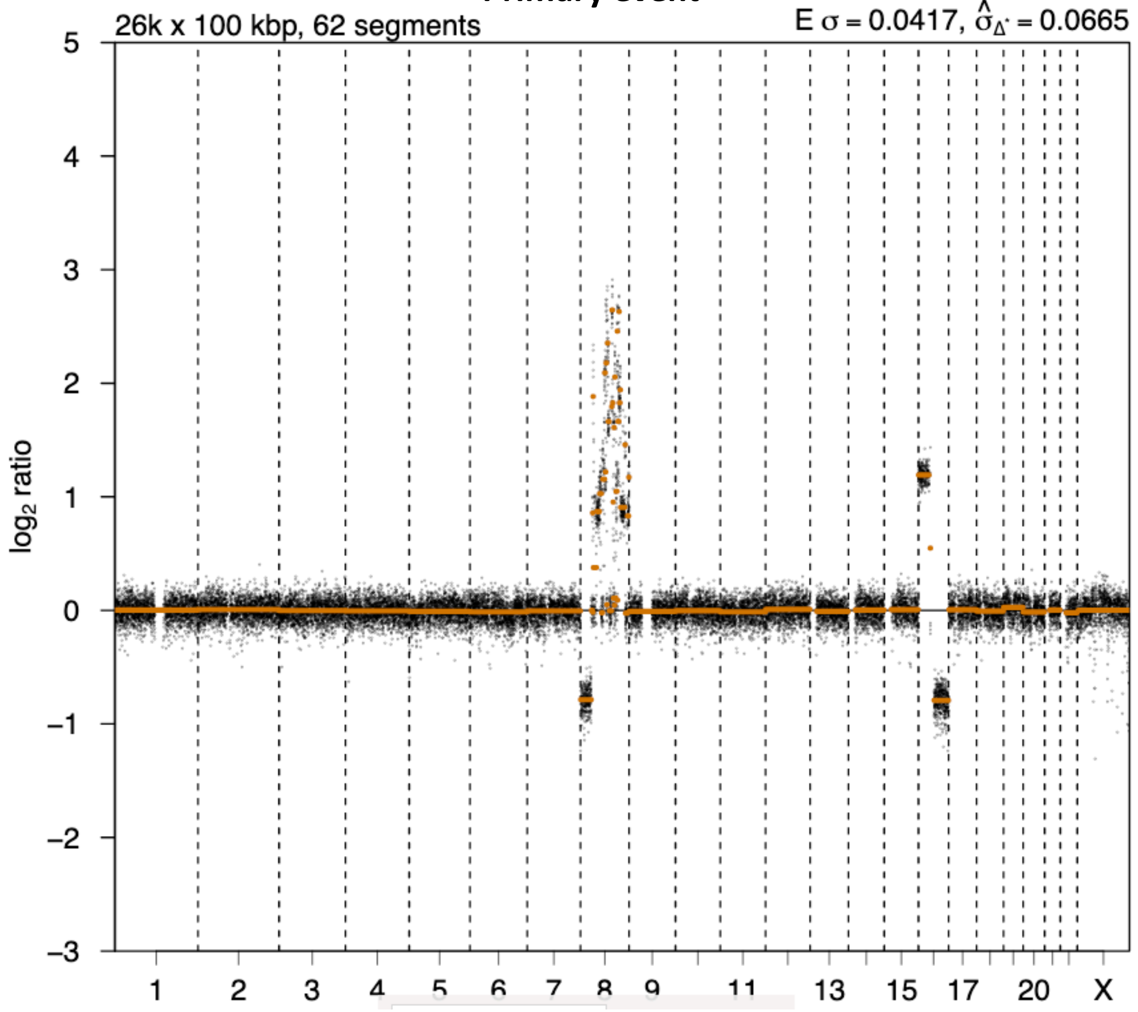

2nd event

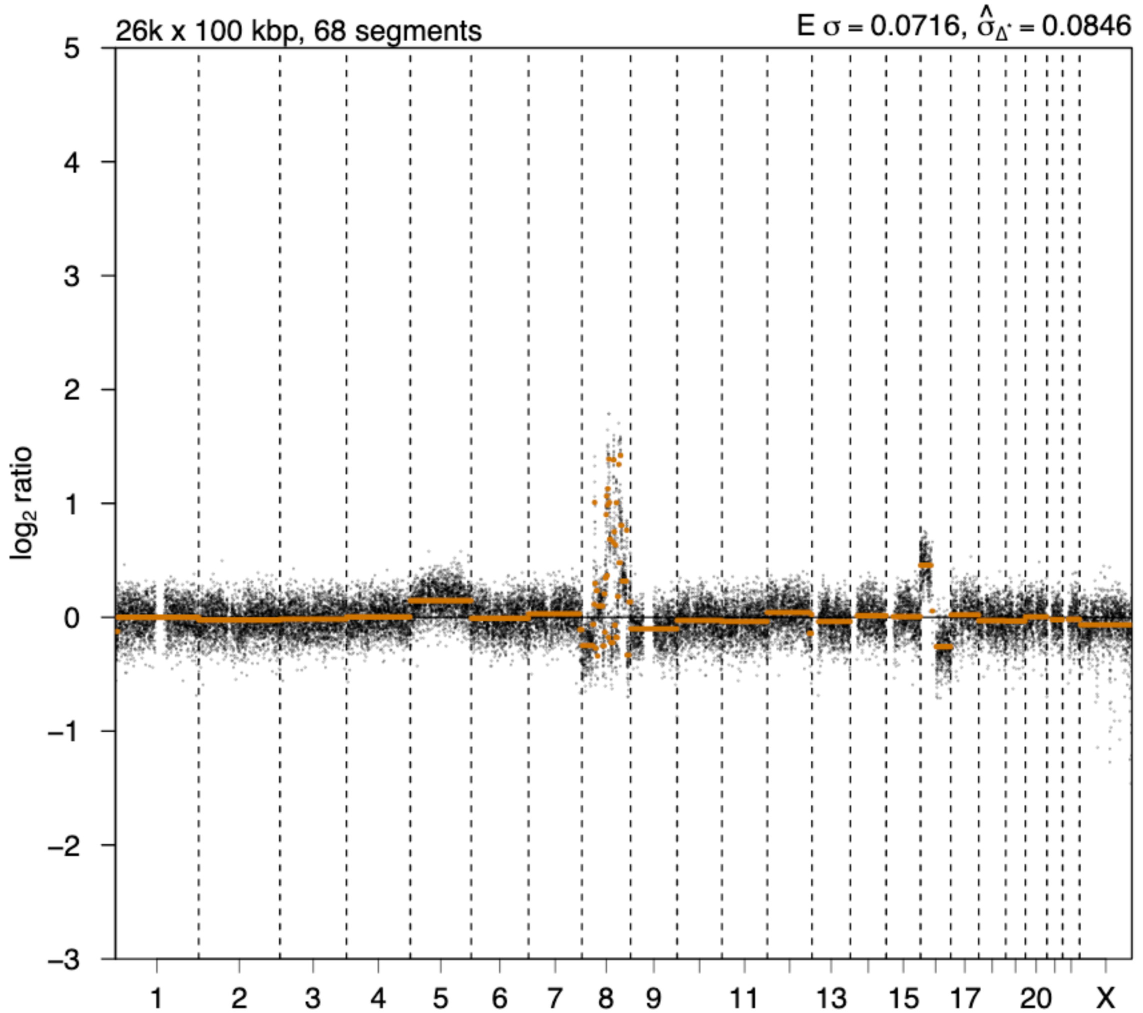

P019

Syn/Meta	Time from 1st surgery to 2nd event (Months)	Side 2nd event	Histology 2nd event	Surgery	AdjuvanT Treatment Pri (RT/ HT)	ER Pri	ER 2nd event	Her2 Pri	Her2 2nd event	Grade Pri	Grade 2nd event	Quadrant 2nd event	Margins	Screening	Clonality P value Copy N	Clonality P value Panel seq	Clonality P value WES	Final verdict
metachronous	18	Ipsilateral	DCIS only	lumpectomy	None	-	-	NA	NA	3	3	NA	Involved	screen-detected	0.003663004	NA	NA	Related

Primary event

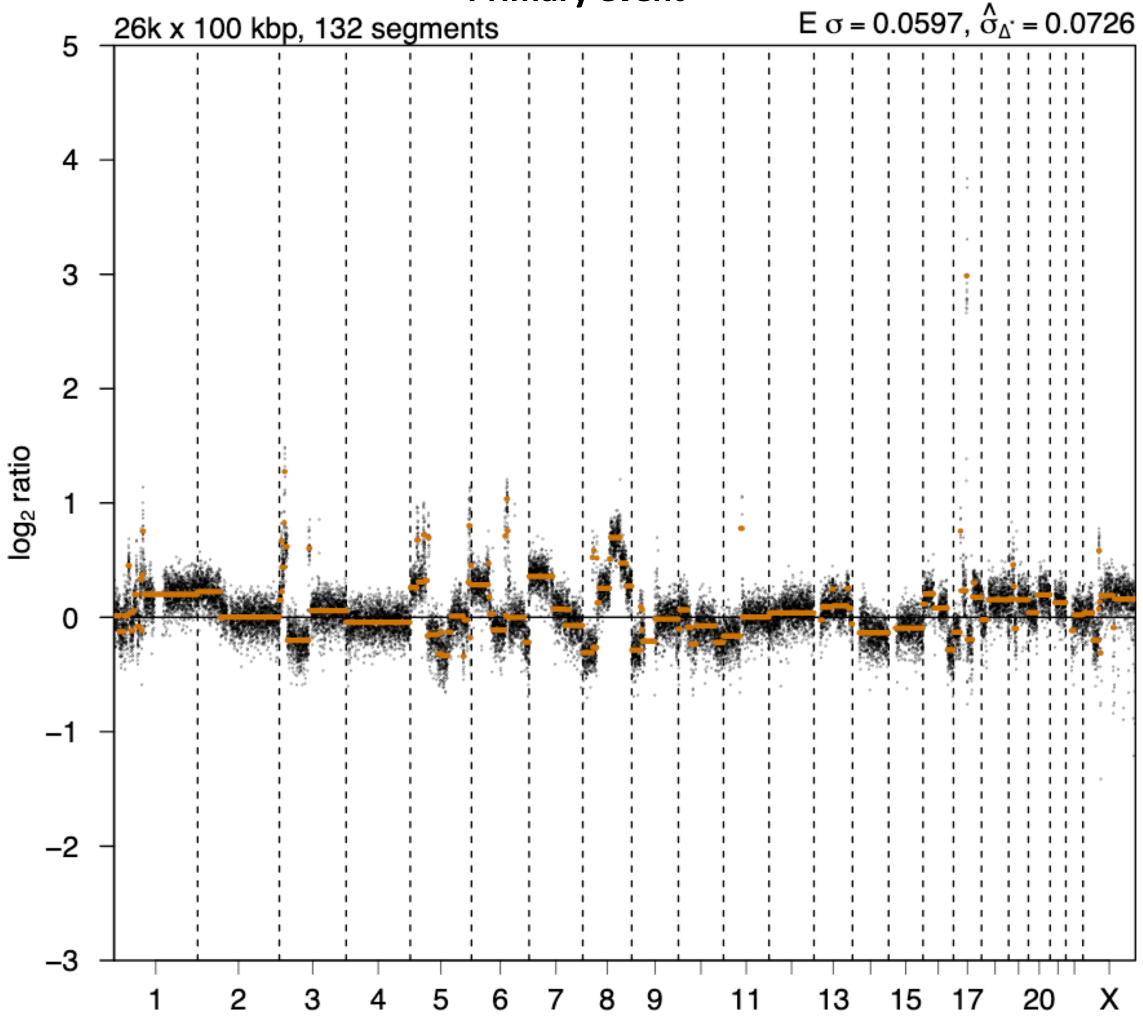

2nd event

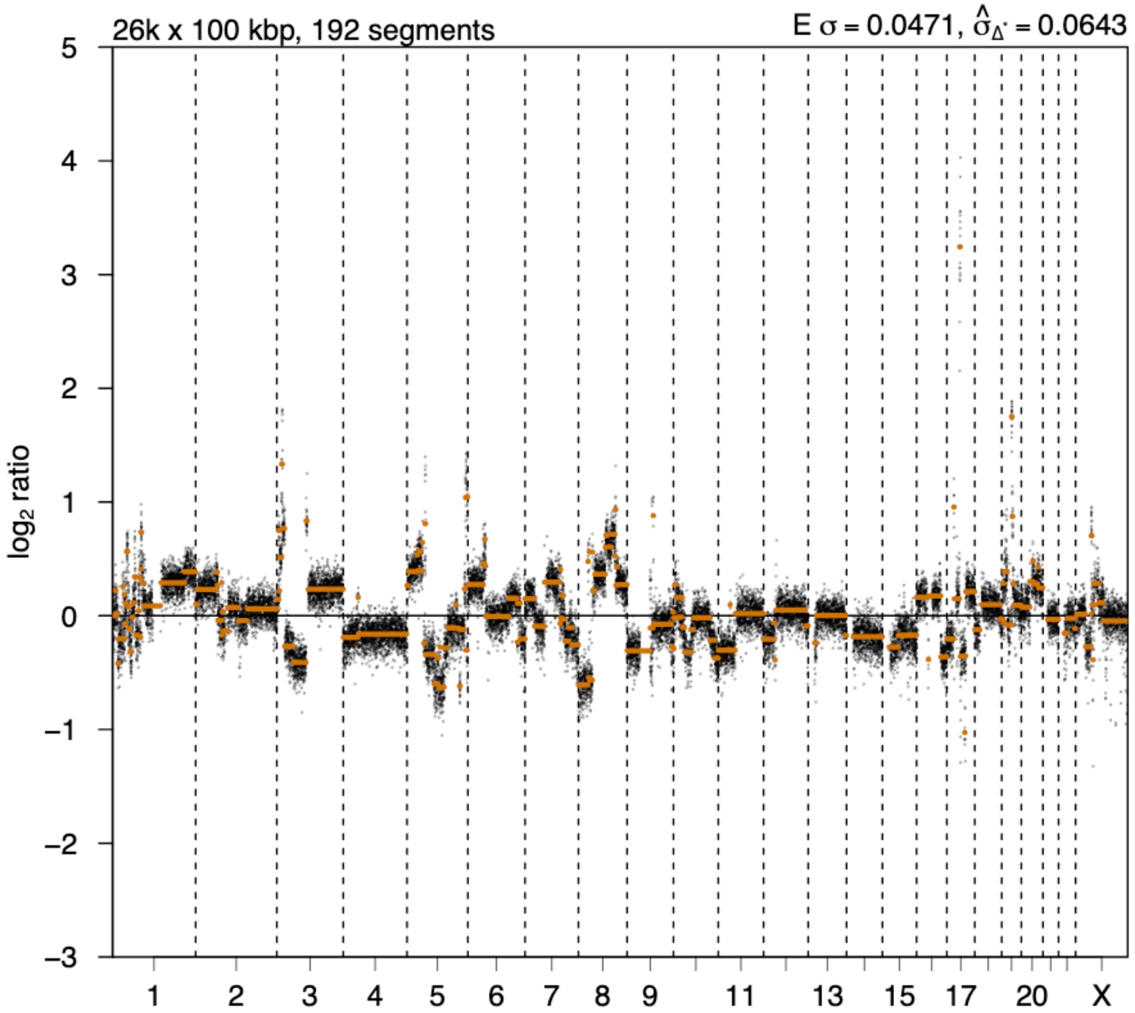

P020

Syn/Meta	Time from 1st surgery to 2nd event (Months)	Side 2nd event	Histology 2nd event	Surgery	AdjuvanT Treatment Pri (RT/ HT)	ER Pri	ER 2nd event	Her2 Pri	Her2 2nd event	Grade Pri	Grade 2nd event	Quadrant 2nd event	Margins	Screening	Clonality P value Copy N	Clonality P value Panel seq	Clonality P value WES	Final verdict
metachronous	17	Ipsilateral	DCIS only	lumpectomy	None	-	NA	NA	NA	3	3	NA	Clear	screen-detected	0.003663004	NA	NA	Related

Primary event

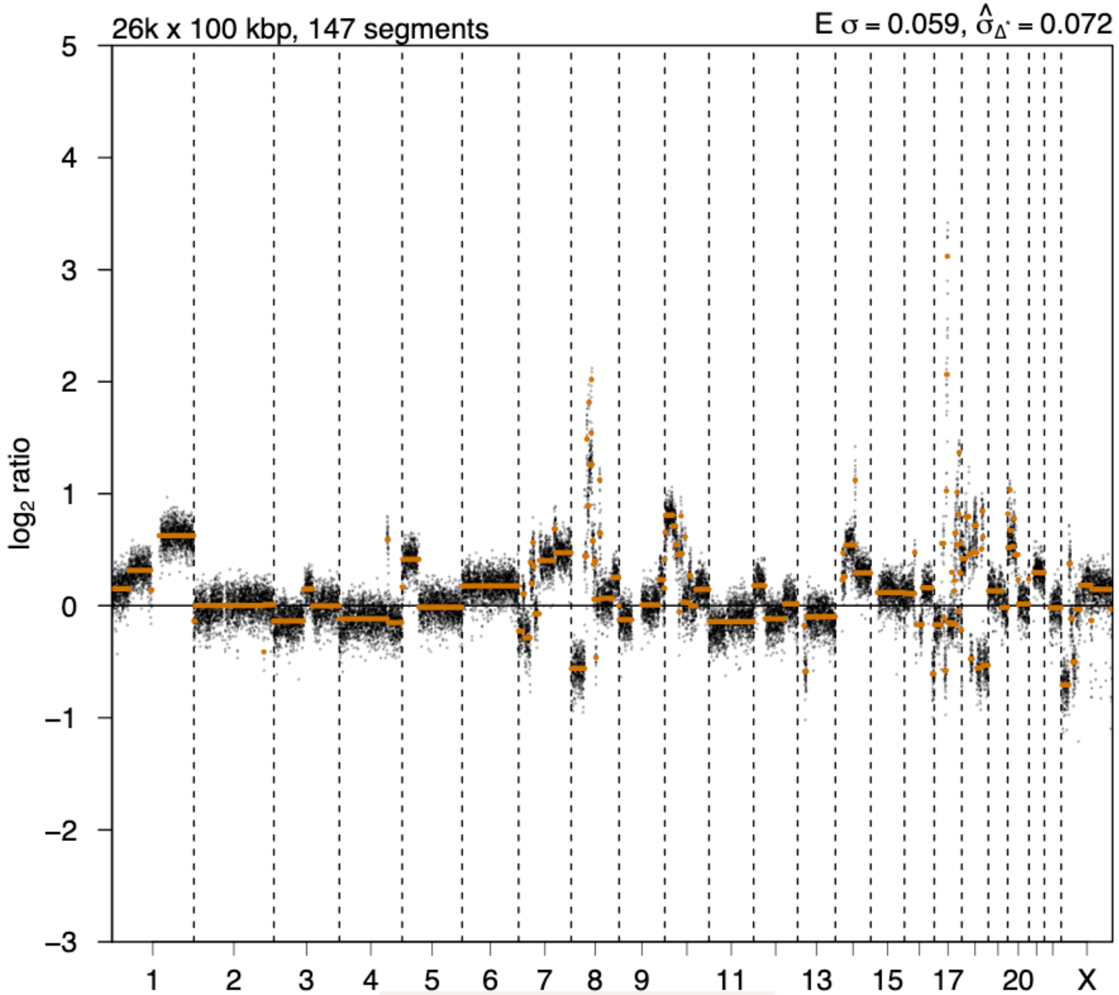

2nd event

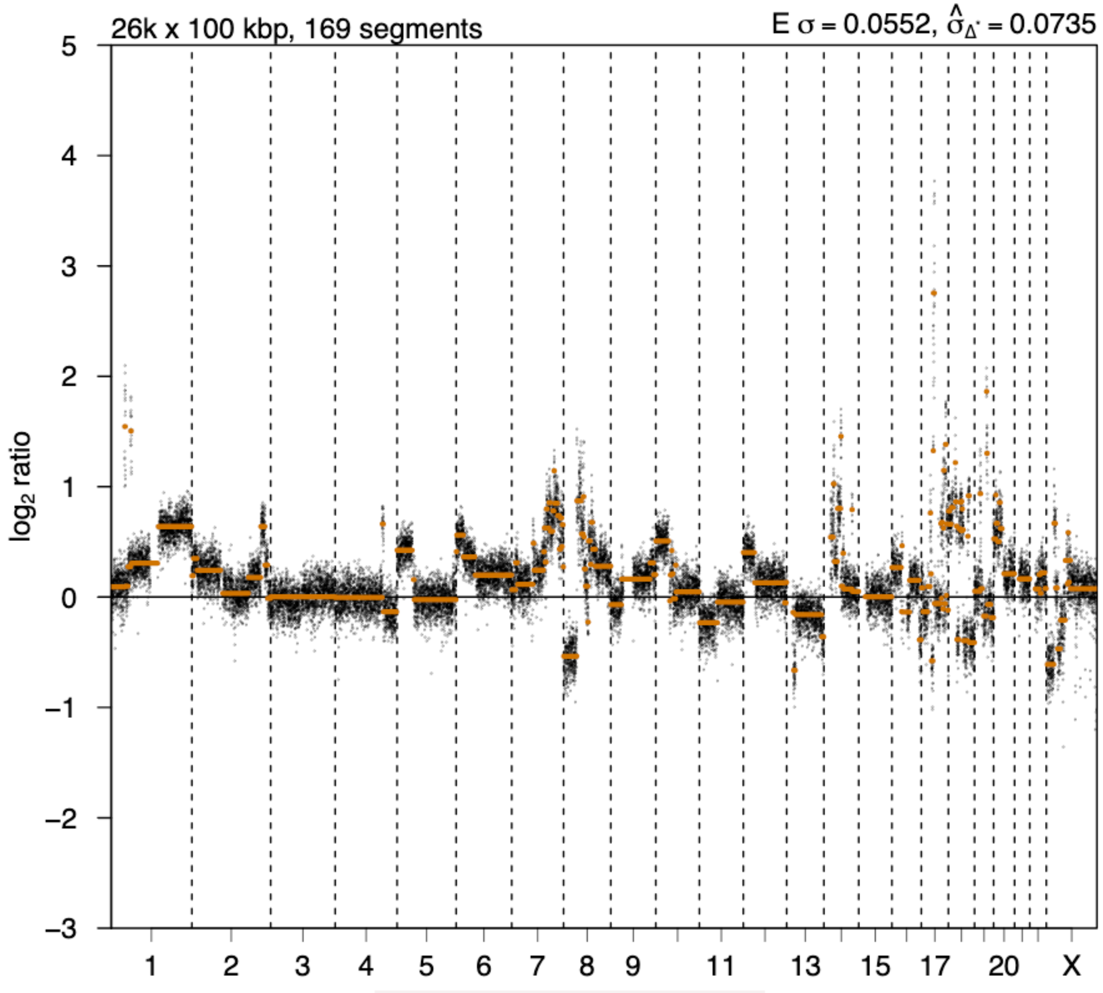

P021

Syn/Meta	Time from 1st surgery to 2nd event (Months)	Side 2nd event	Histology 2nd event	Surgery	AdjuvanT Treatment Pri (RT/ HT)	ER Pri	ER 2nd event	Her2 Pri	Her2 2nd event	Grade Pri	Grade 2nd event	Quadrant 2nd event	Margins	Screening	Clonality P value Copy N	Clonality P value Panel seq	Clonality P value WES	Final verdict
metachronous	18	Ipsilateral	DCIS only	lumpectomy	None	-	-	NA	NA	3	3	NA	Clear	screen-detected	0.003663004	NA	NA	Related

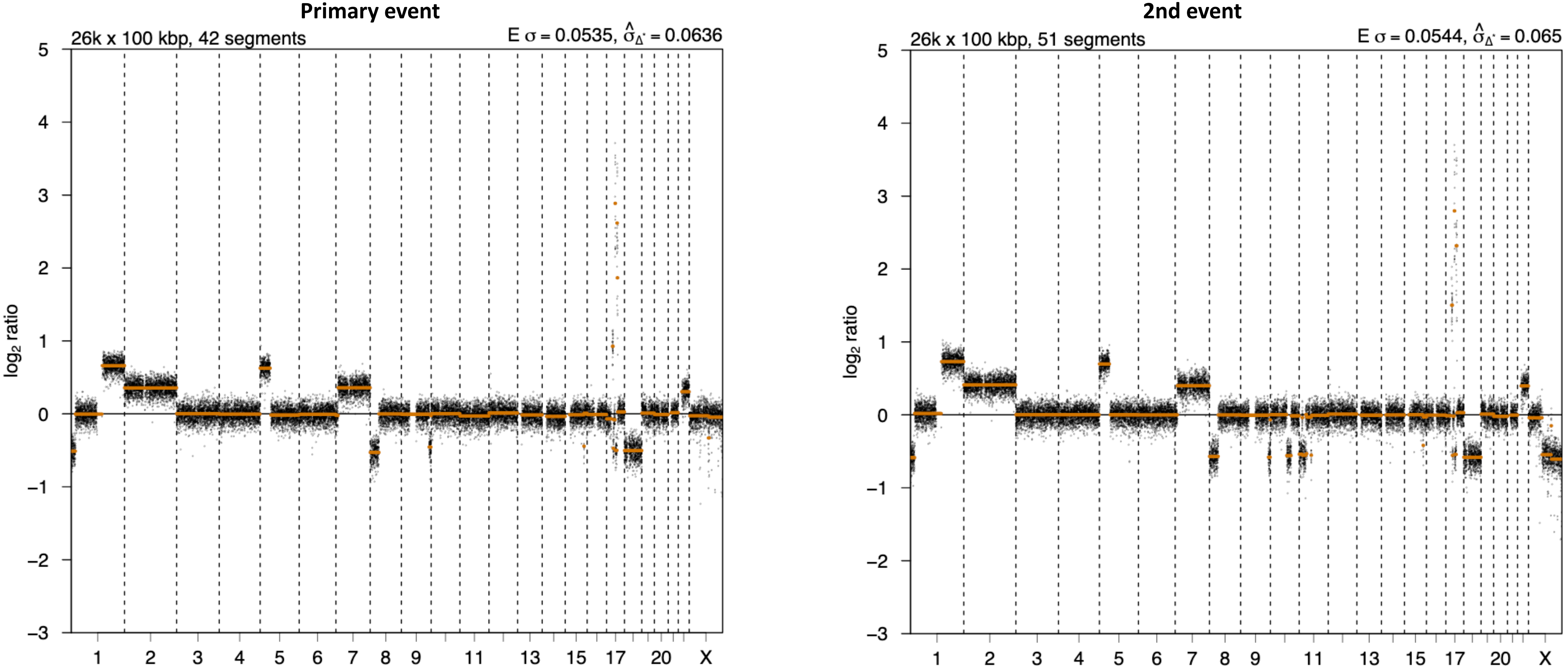

P023

Syn/Meta	Time from 1st surgery to 2nd event (Months)	Side 2nd event	Histology 2nd event	Surgery	AdjuvanT Treatment Pri (RT/ HT)	ER Pri	ER 2nd event	Her2 Pri	Her2 2nd event	Grade Pri	Grade 2nd event	Quadrant 2nd event	Margins	Screening	Clonality P value Copy N	Clonality P value Panel seq	Clonality P value WES	Final verdict
metachronous	25	Ipsilateral	DCIS only	lumpectomy	RT	NA	NA	-	NA	3	3	NA	Clear	screen-detected	0.003663004	NA	NA	Related

Primary event

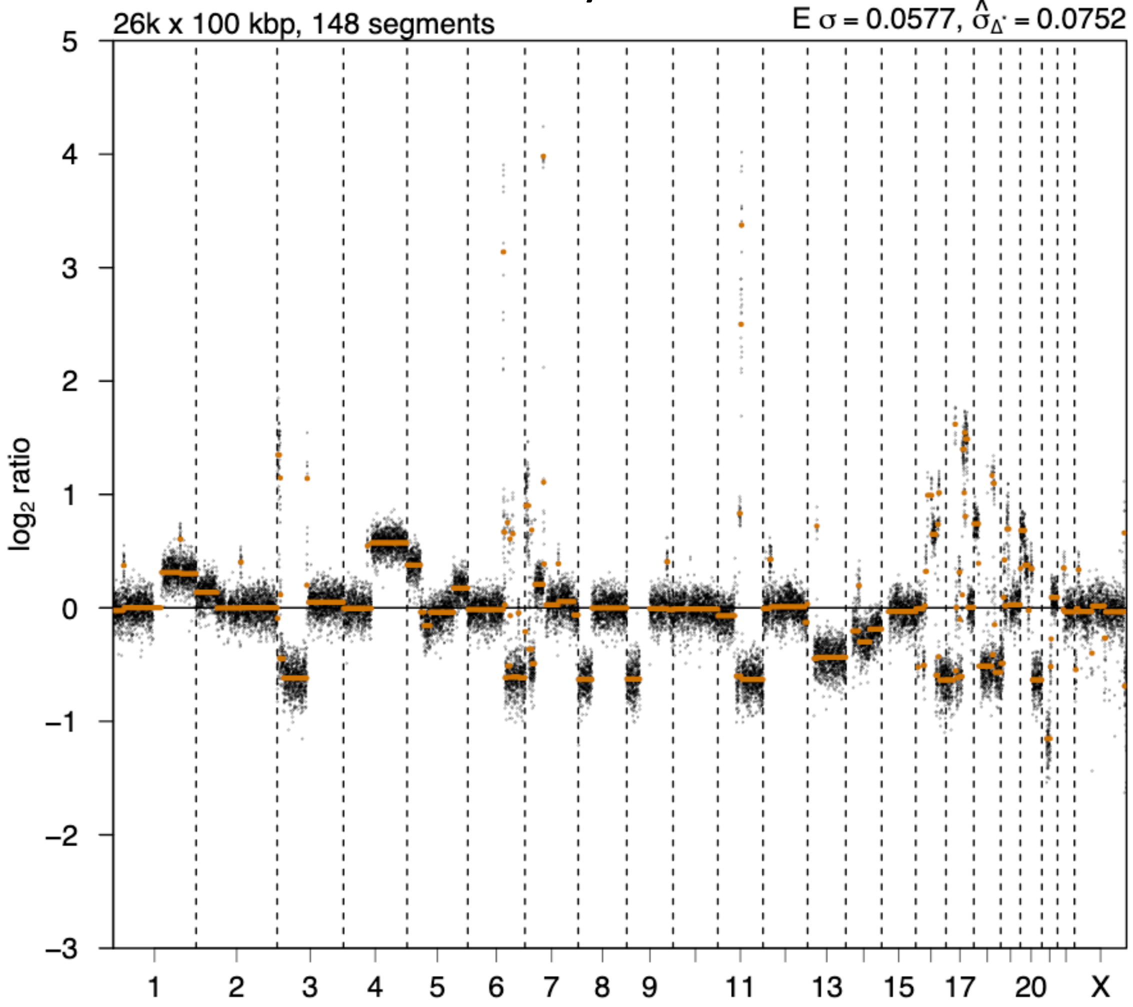

2nd event

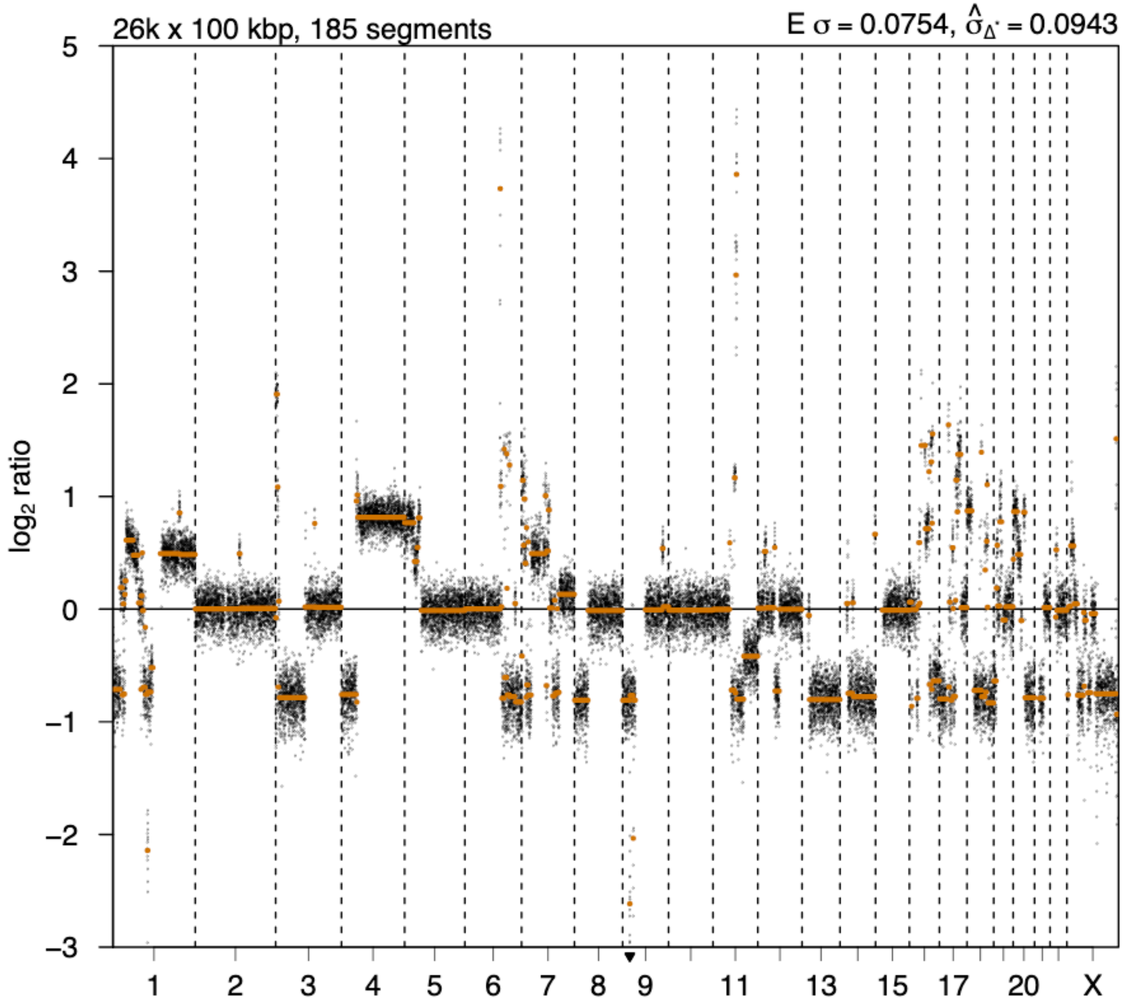

P024

Syn/Meta	Time from 1st surgery to 2nd event (Months)	Side 2nd event	Histology 2nd event	Surgery	AdjuvanT Treatment Pri (RT/ HT)	ER Pri	ER 2nd event	Her2 Pri	Her2 2nd event	Grade Pri	Grade 2nd event	Quadrant 2nd event	Margins	Screening	Clonality P value Copy N	Clonality P value Panel seq	Clonality P value WES	Final verdict
metachronous	27	Ipsilateral	DCIS only	lumpectomy	None	+	NA	-	NA	2	3	at or adjacent to primary	Clear	screen-detected	0.000466	NA	NA	Related

Primary event

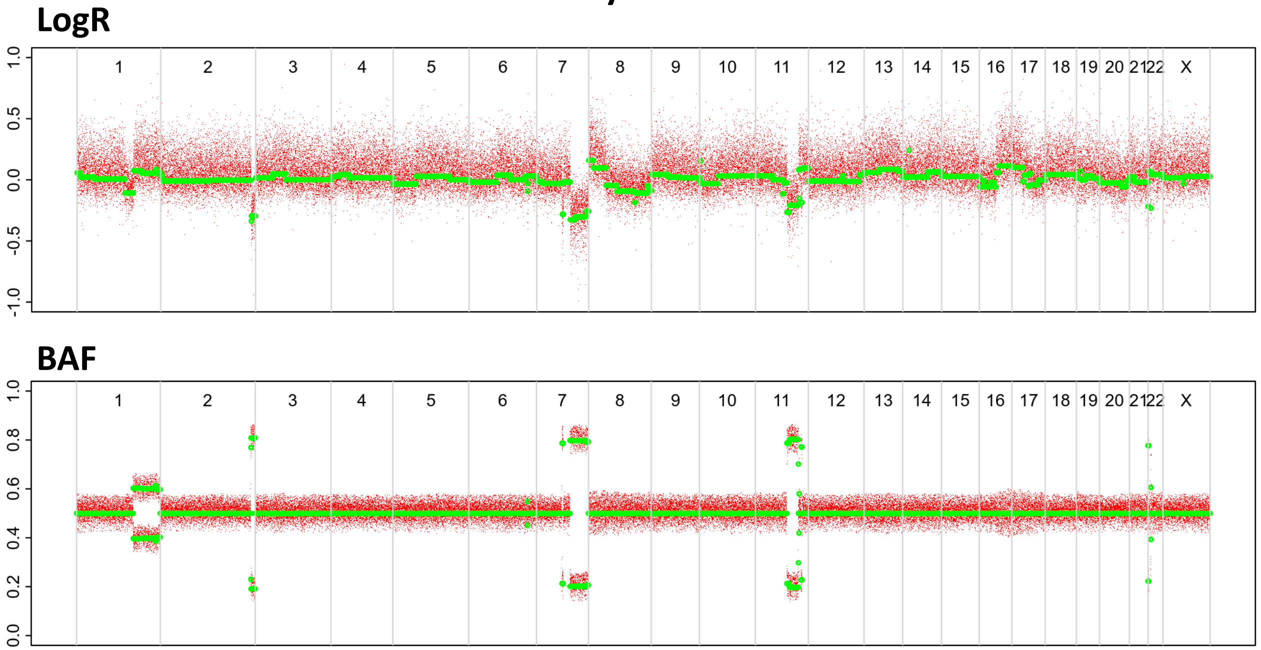

2nd event

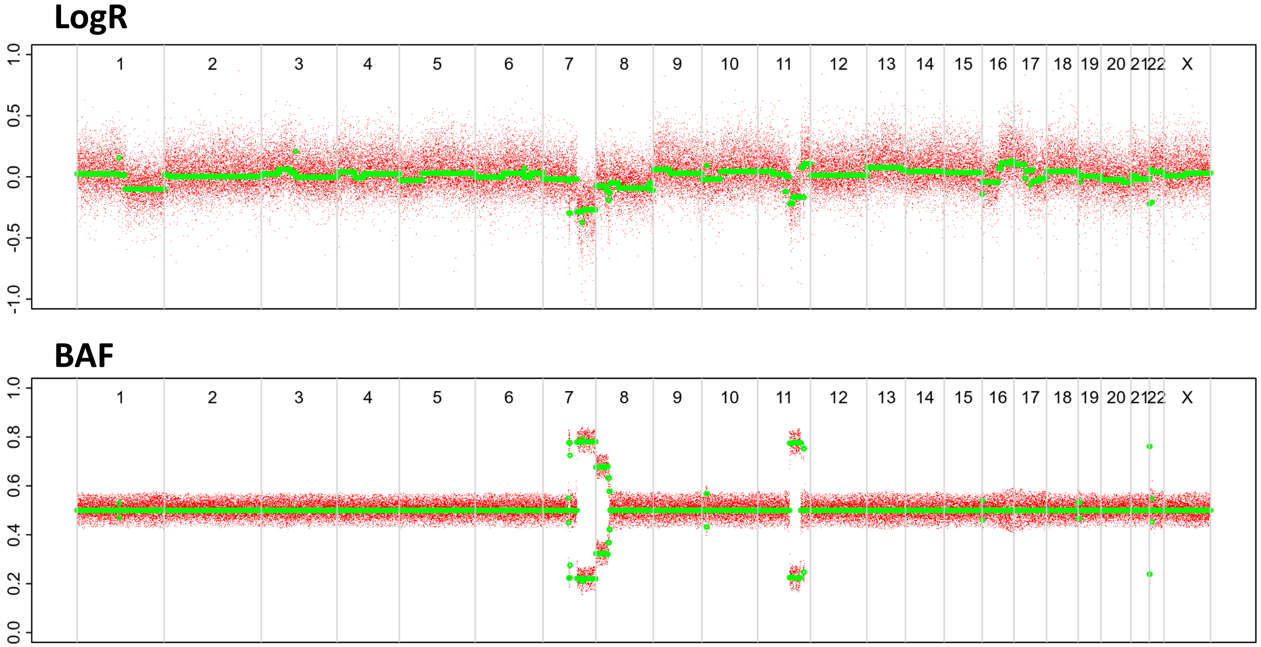

P026

Syn/Meta	Time from 1st surgery to 2nd event (Months)	Side 2nd event	Histology 2nd event	Surgery	AdjuvanT Treatment Pri (RT/ HT)	ER Pri	ER 2nd event	Her2 Pri	Her2 2nd event	Grade Pri	Grade 2nd event	Quadrant 2nd event	Margins	Screening	Clonality P value Copy N	Clonality P value Panel seq	Clonality P value WES	Final verdict
metachronous	26	Ipsilateral	DCIS only	lumpectomy	RT	+	+	+	+	3	3	at or adjacent to primary	Clear	screen-detected	0.000466	NA	NA	Related

Primary event

LogR

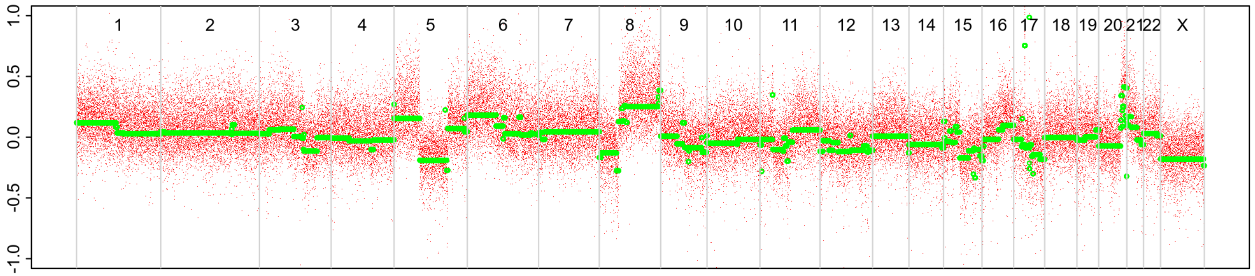

BAF

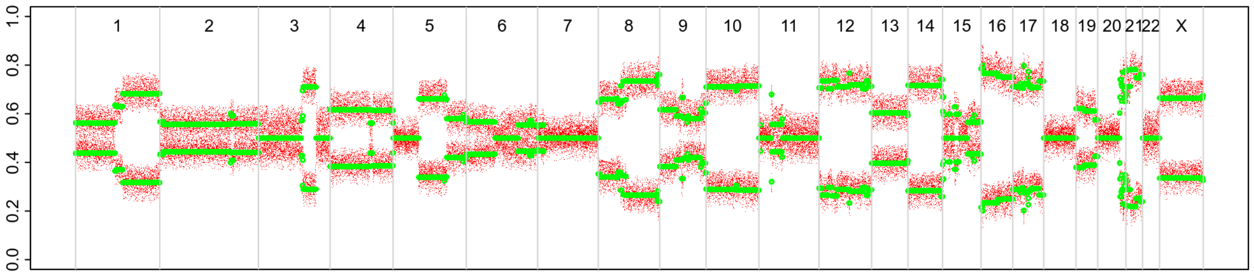

2nd event

LogR

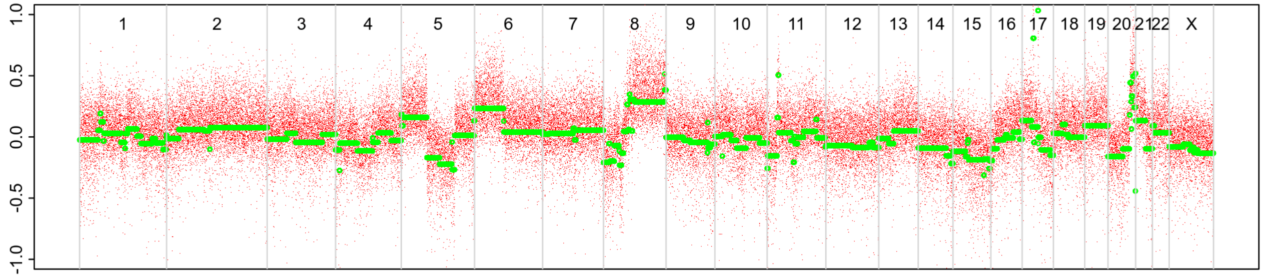

BAF

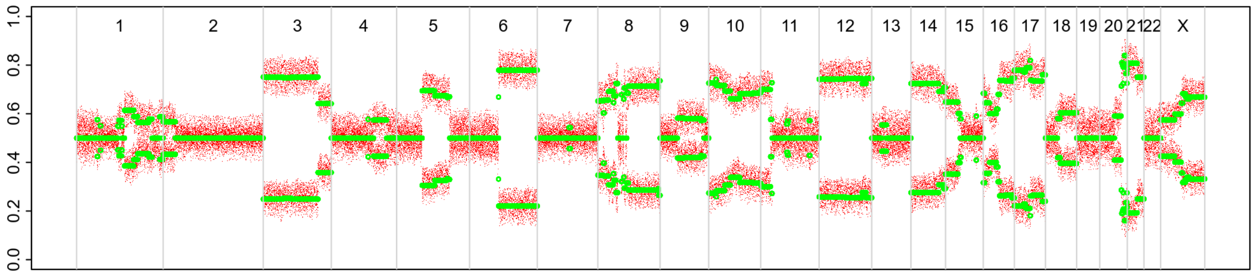

P027

Syn/Meta	Time from 1st surgery to 2nd event (Months)	Side 2nd event	Histology 2nd event	Surgery	AdjuvanT Treatment Pri (RT/ HT)	ER Pri	ER 2nd event	Her2 Pri	Her2 2nd event	Grade Pri	Grade 2nd event	Quadrant 2nd event	Margins	Screening	Clonality P value Copy N	Clonality P value Panel seq	Clonality P value WES	Final verdict
metachronous	15	Ipsilateral	DCIS only	lumpectomy	RT	-	NA	+	+	3	3	at or adjacent to primary	Clear	screen-detected	0.000466	0	NA	Related

Primary event

LogR

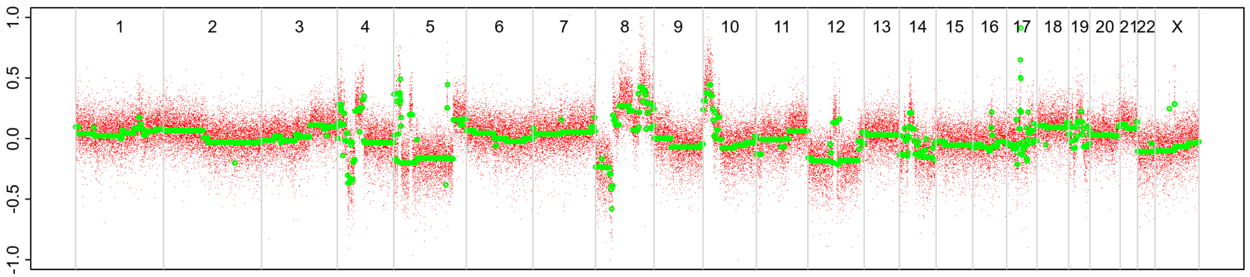

BAF

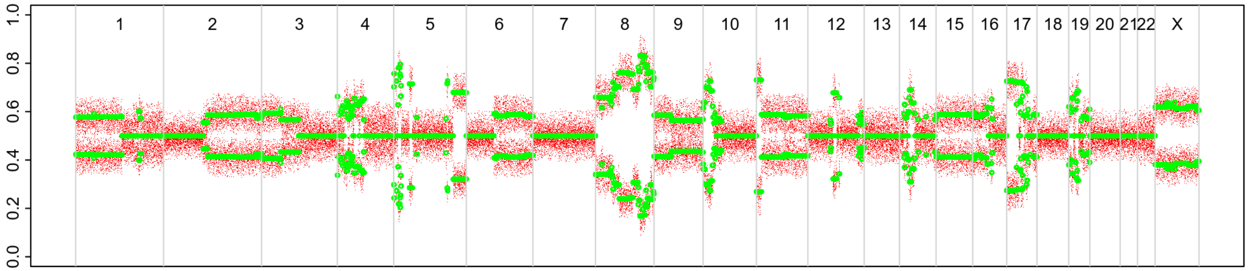

2nd event

LogR

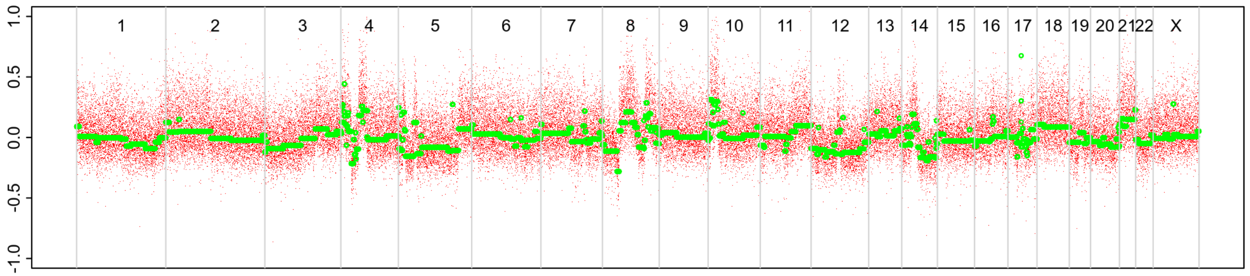

BAF

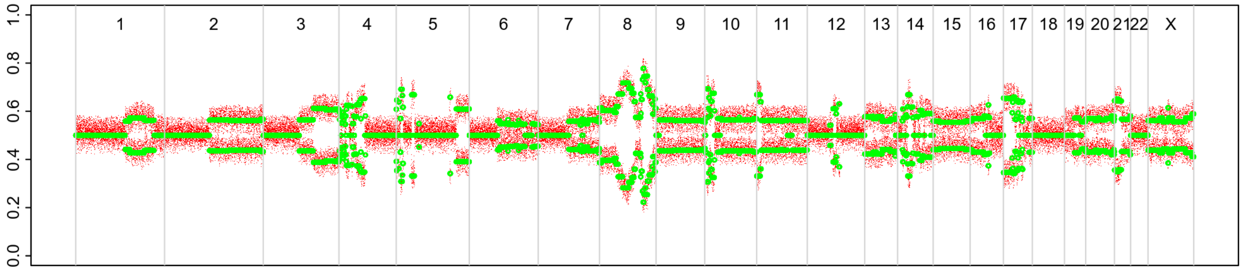

P028

Syn/Meta	Time from 1st surgery to 2nd event (Months)	Side 2nd event	Histology 2nd event	Surgery	AdjuvanT Treatment Pri (RT/ HT)	ER Pri	ER 2nd event	Her2 Pri	Her2 2nd event	Grade Pri	Grade 2nd event	Quadrant 2nd event	Margins	Screening	Clonality P value Copy N	Clonality P value Panel seq	Clonality P value WES	Final verdict
metachronous	16	Ipsilateral	DCIS only	lumpectomy	RT	-	NA	+	NA	3	3	at or adjacent to primary	Clear	screen-detected	0.000466	NA	NA	Related

Primary event

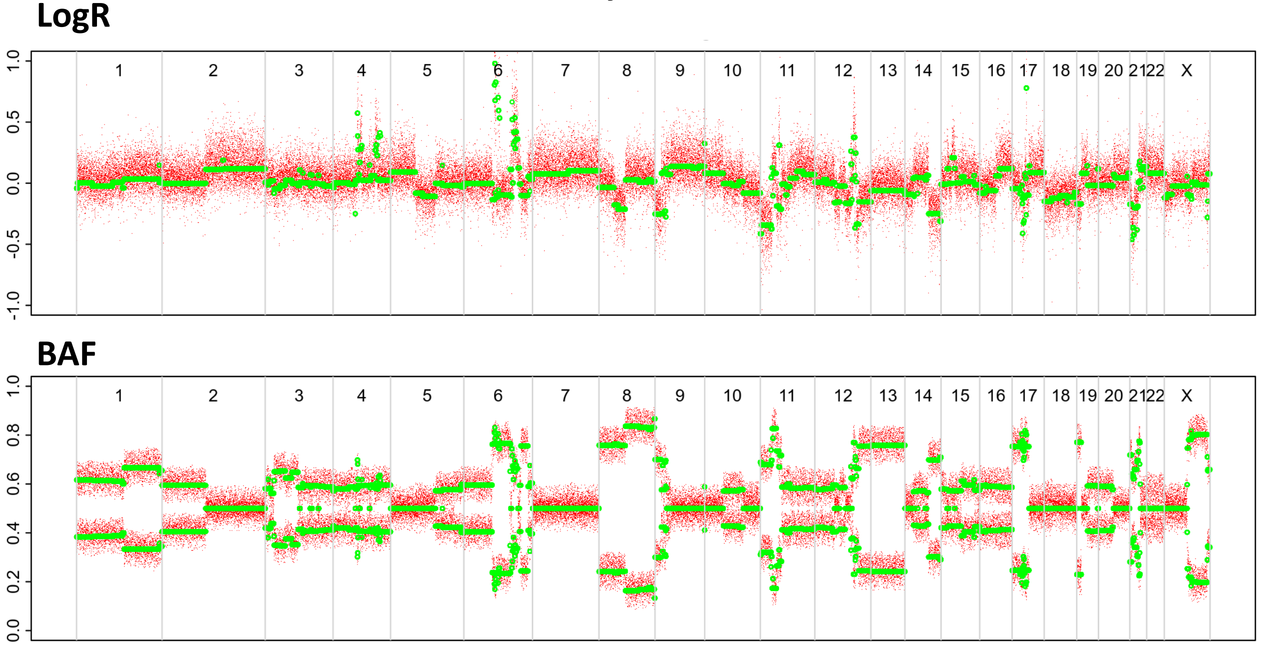

2nd event

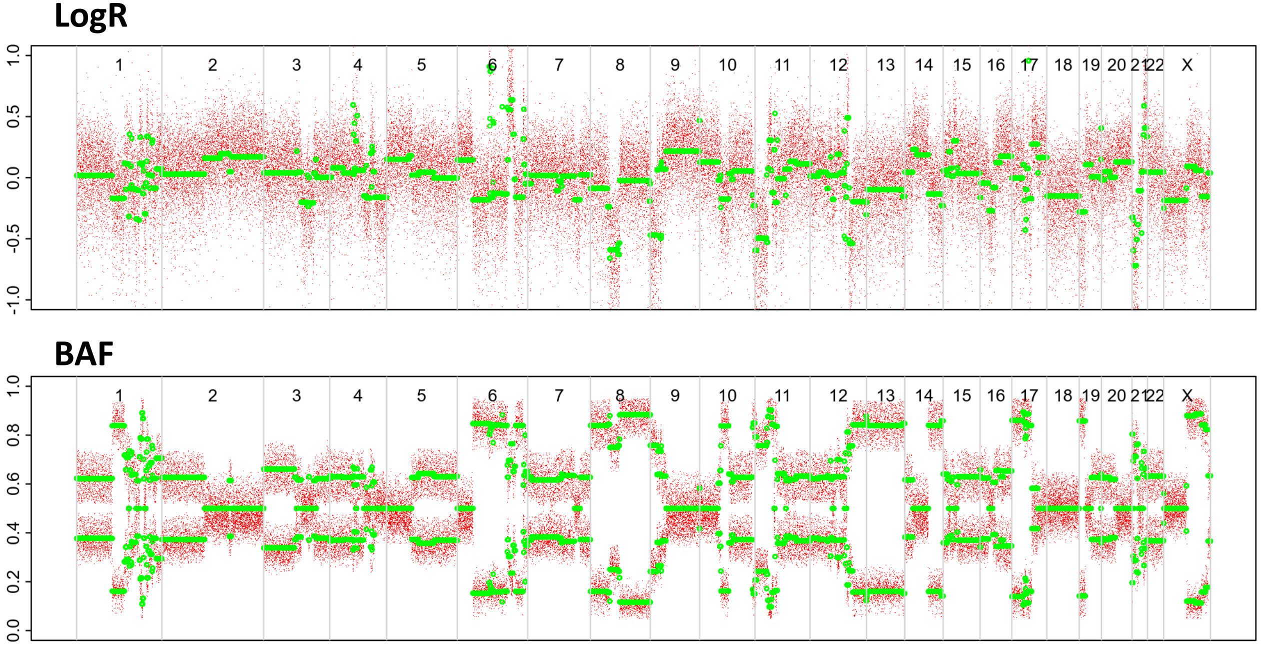

P029

Syn/Meta	Time from 1st surgery to 2nd event (Months)	Side 2nd event	Histology 2nd event	Surgery	AdjuvanT Treatment Pri (RT/ HT)	ER Pri	ER 2nd event	Her2 Pri	Her2 2nd event	Grade Pri	Grade 2nd event	Quadrant 2nd event	Margins	Screening	Clonality P value Copy N	Clonality P value Panel seq	Clonality P value WES	Final verdict
metachronous	26	Ipsilateral	DCIS only	lumpectomy	None	-	NA	NA	NA	3	3	at or adjacent to primary	Clear	screen-detected	0.000466	0	NA	Related

Primary event

LogR

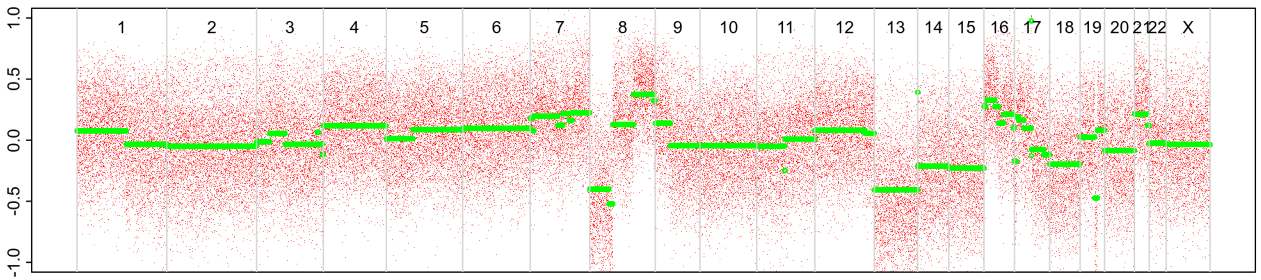

BAF

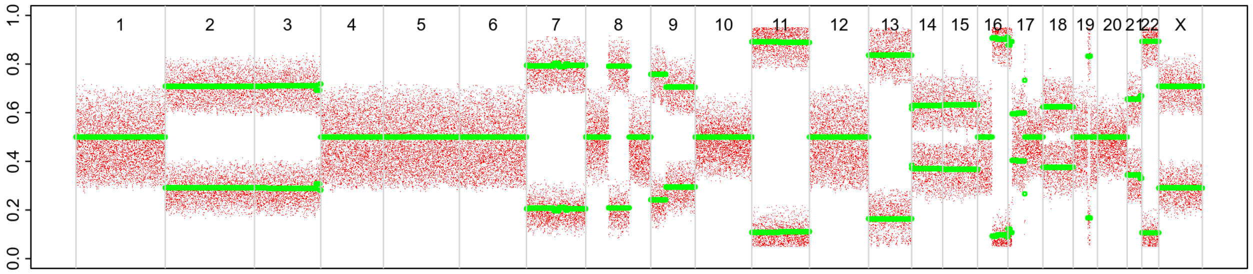

2nd event

LogR

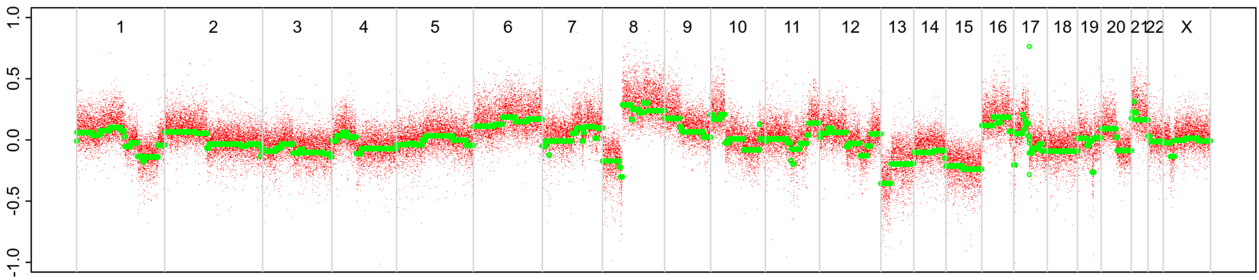

BAF

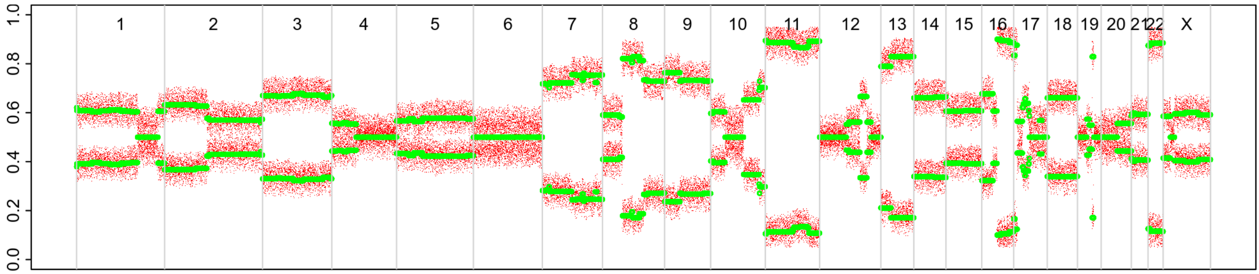

P058

Syn/Meta	Time from 1st surgery to 2nd event (Months)	Side 2nd event	Histology 2nd event	Surgery	AdjuvanT Treatment Pri (RT/ HT)	ER Pri	ER 2nd event	Her2 Pri	Her2 2nd event	Grade Pri	Grade 2nd event	Quadrant 2nd event	Margins	Screening	Clonality P value Copy N	Clonality P value Panel seq	Clonality P value WES	Final verdict
metachronous	62	Ipsilateral	IDC no DCIS	lumpectomy	RTHT	+	NA	-	NA	3	3	at or adjacent to primary	Clear	screen-detected	0.000466	0	NA	Related

Primary event

LogR

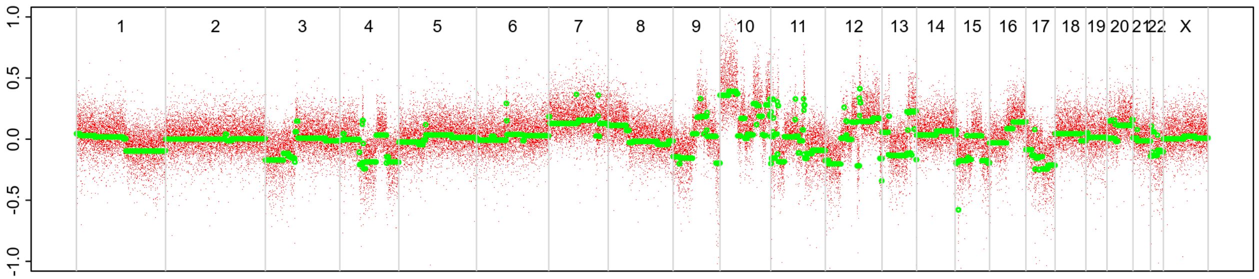

BAF

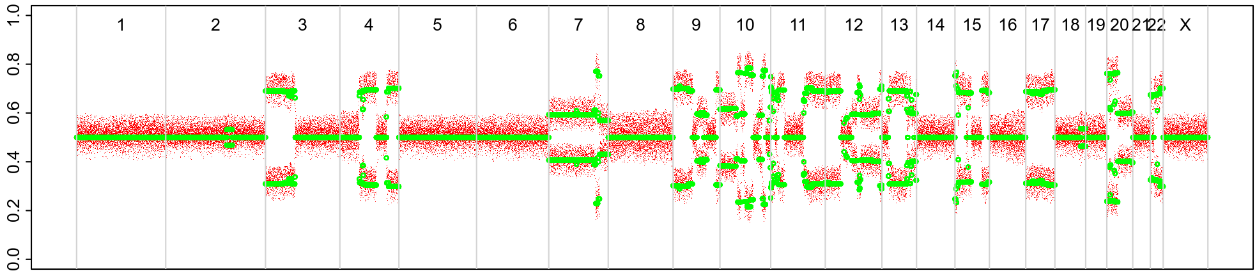

2nd event

LogR

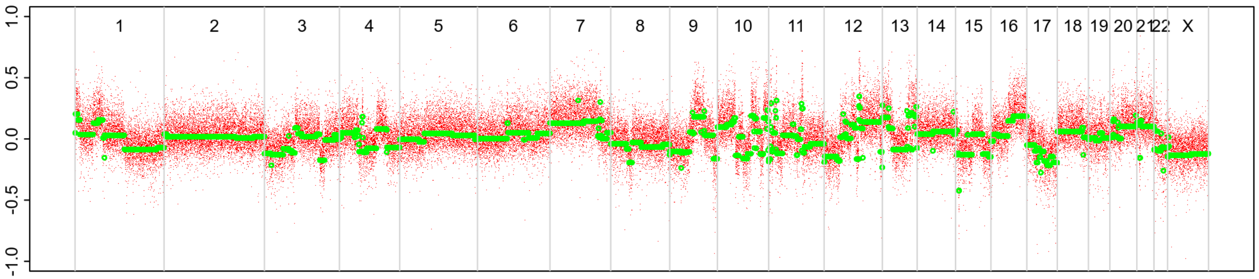

BAF

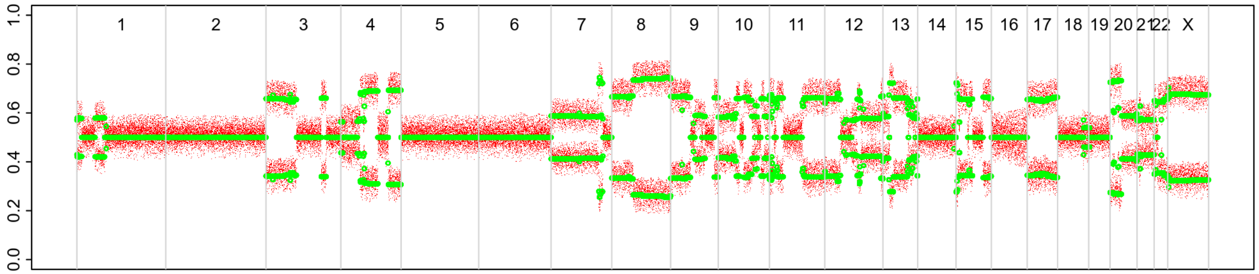

P064

Syn/Meta	Time from 1st surgery to 2nd event (Months)	Side 2nd event	Histology 2nd event	Surgery	AdjuvanT Treatment Pri (RT/ HT)	ER Pri	ER 2nd event	Her2 Pri	Her2 2nd event	Grade Pri	Grade 2nd event	Quadrant 2nd event	Margins	Screening	Clonality P value Copy N	Clonality P value Panel seq	Clonality P value WES	Final verdict
metachronous	54	Ipsilateral	IDC with DCIS	lumpectomy	None	+	+	-	NA	3	2	NA	Clear	screen-detected	0.000466	0	NA	Related

Primary event

LogR

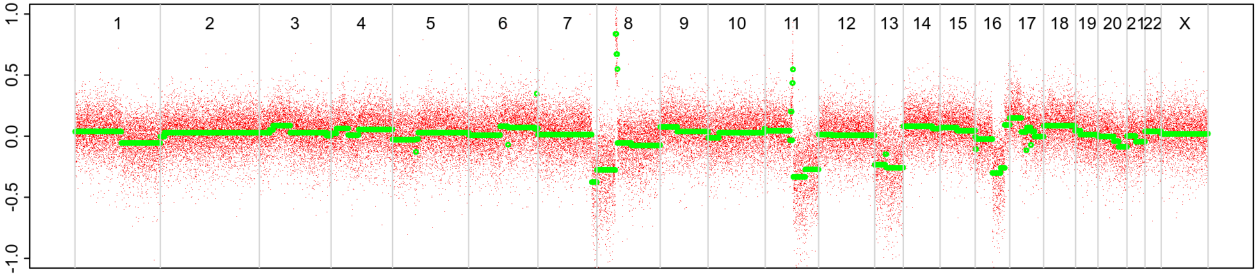

BAF

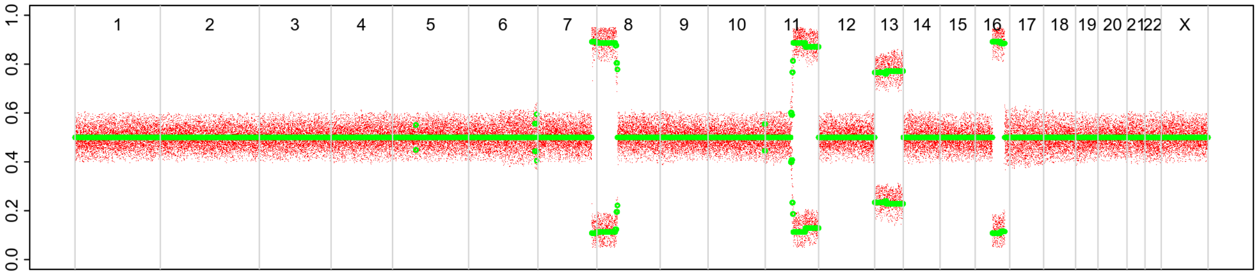

2nd event

LogR

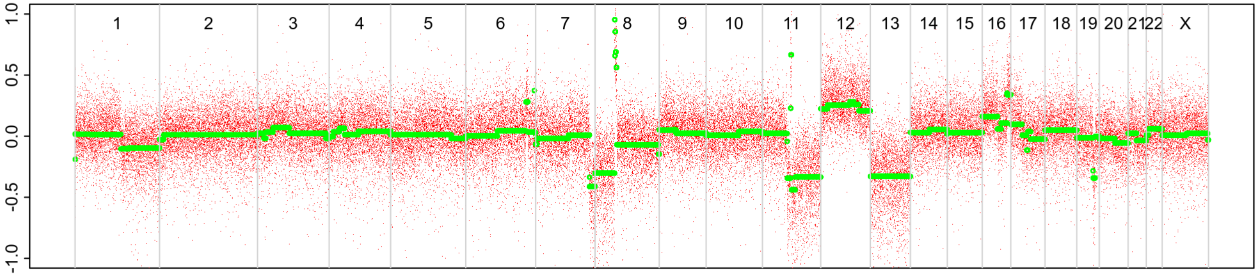

BAF

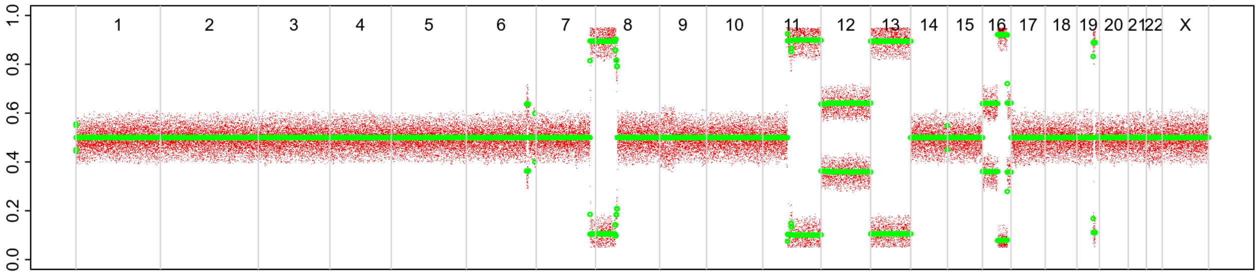

P070

Syn/Meta	Time from 1st surgery to 2nd event (Months)	Side 2nd event	Histology 2nd event	Surgery	AdjuvanT Treatment Pri (RT/ HT)	ER Pri	ER 2nd event	Her2 Pri	Her2 2nd event	Grade Pri	Grade 2nd event	Quadrant 2nd event	Margins	Screening	Clonality P value Copy N	Clonality P value Panel seq	Clonality P value WES	Final verdict
metachronous	35	Ipsilateral	IDC no DCIS	lumpectomy	None	+	+	+	-	3	2	at or adjacent to primary	Clear	screen-detected	0.000466	0	NA	Related

Primary event

LogR

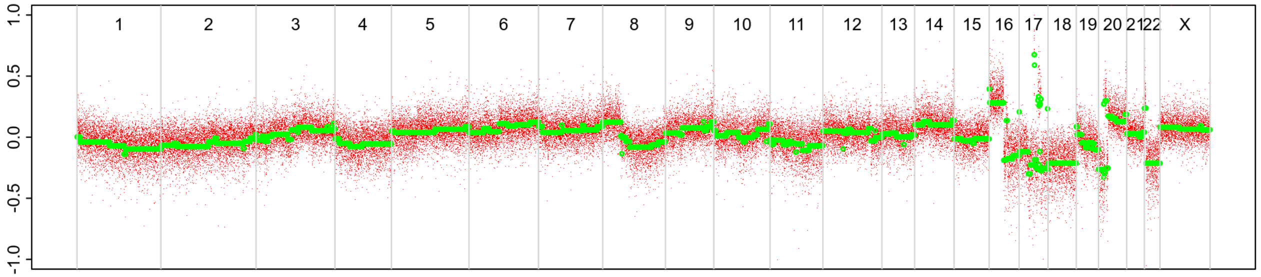

BAF

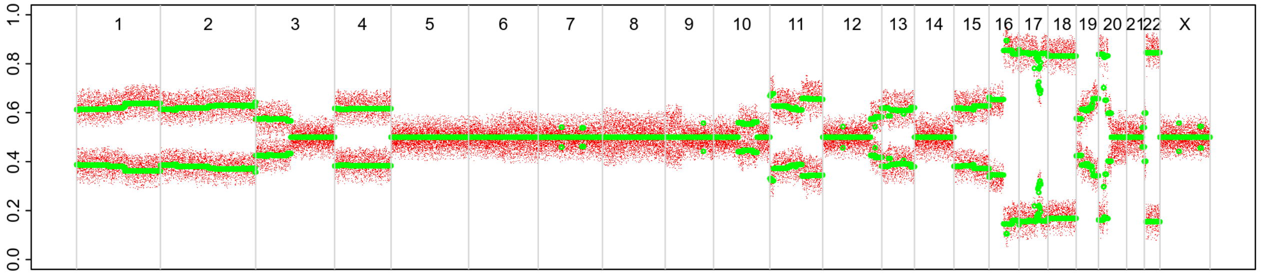

2nd event

LogR

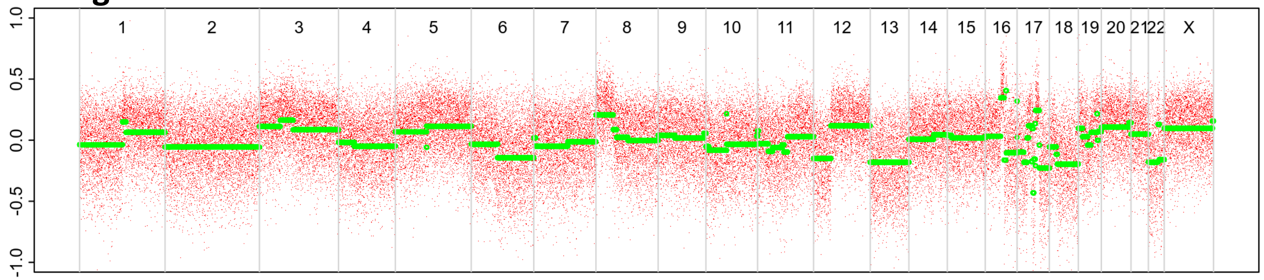

BAF

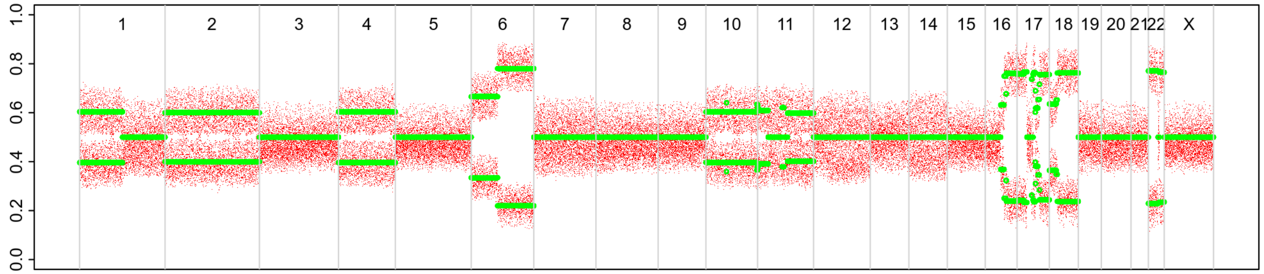

P071

Syn/Meta	Time from 1st surgery to 2nd event (Months)	Side 2nd event	Histology 2nd event	Surgery	AdjuvanT Treatment Pri (RT/ HT)	ER Pri	ER 2nd event	Her2 Pri	Her2 2nd event	Grade Pri	Grade 2nd event	Quadrant 2nd event	Margins	Screening	Clonality P value Copy N	Clonality P value Panel seq	Clonality P value WES	Final verdict
metachronous	52	Ipsilateral	IDC with DCIS*	lumpectomy	RT	+	-	+	NA	3	3	at or adjacent to primary	Clear	screen-detected	0.000932	NA	NA	Related

Clonality P value for ipsilateral synchronous 2nd event Copy Number
0.004662

Primary event

LogR

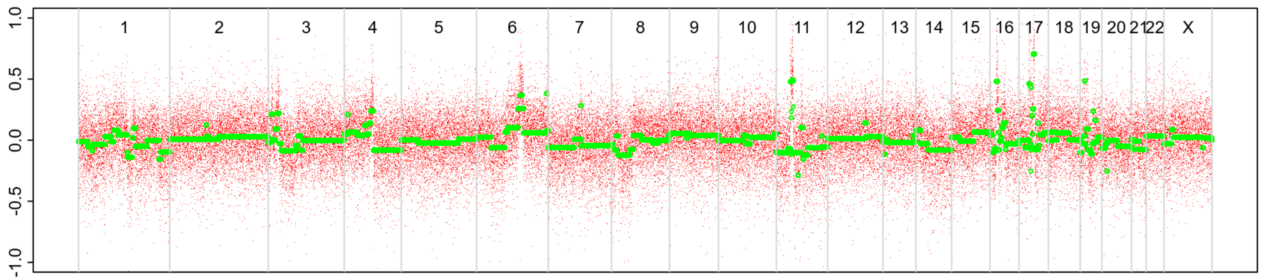

BAF

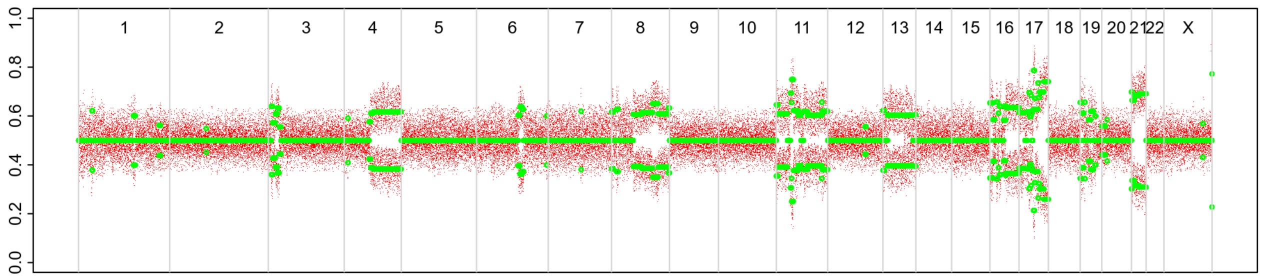

2nd event (INV)

LogR

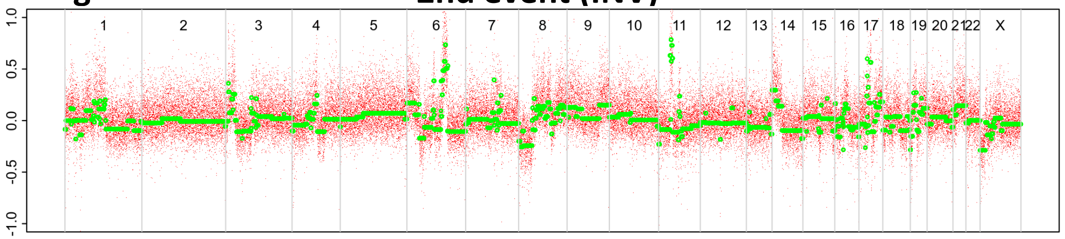

BAF

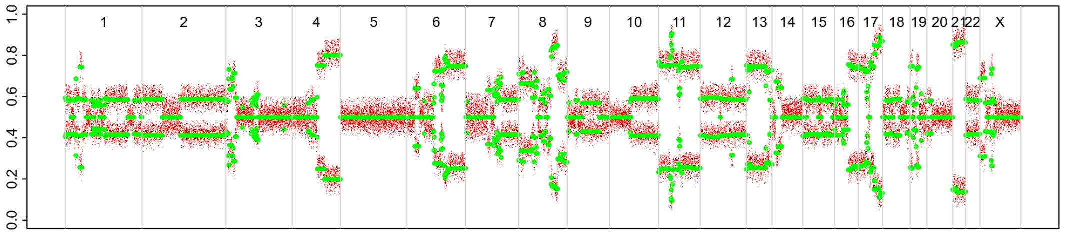

2nd event (DCIS)

LogR

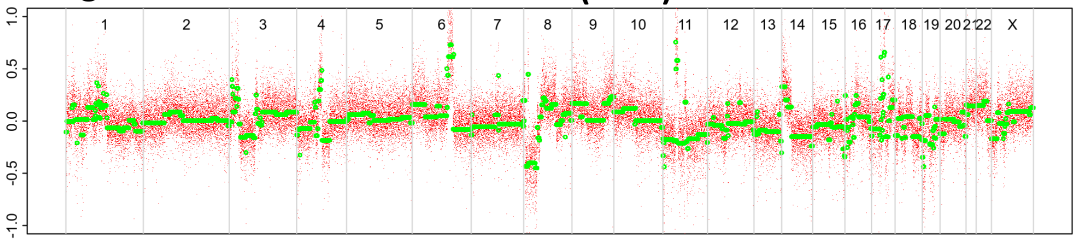

BAF

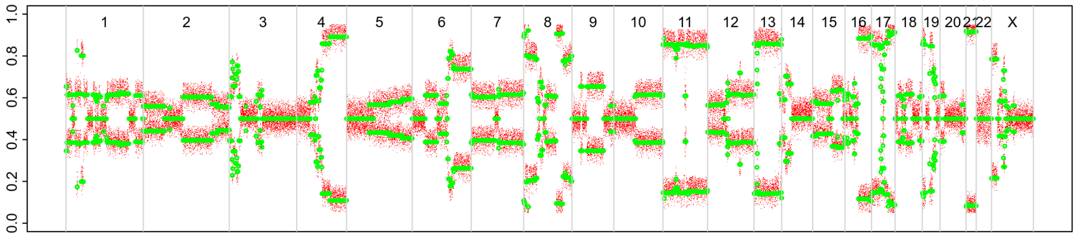

P074

Syn/Meta	Time from 1st surgery to 2nd event (Months)	Side 2nd event	Histology 2nd event	Surgery	AdjuvanT Treatment Pri (RT/ HT)	ER Pri	ER 2nd event	Her2 Pri	Her2 2nd event	Grade Pri	Grade 2nd event	Quadrant 2nd event	Margins	Screening	Clonality P value Copy N	Clonality P value Panel seq	Clonality P value WES	Final verdict
metachronous	53	Ipsilateral	IDC no DCIS	lumpectomy	None	-	NA	+	+	3	3	at or adjacent to primary	Clear	screen-detected	0.000466	0.001009082	NA	Related

Primary event

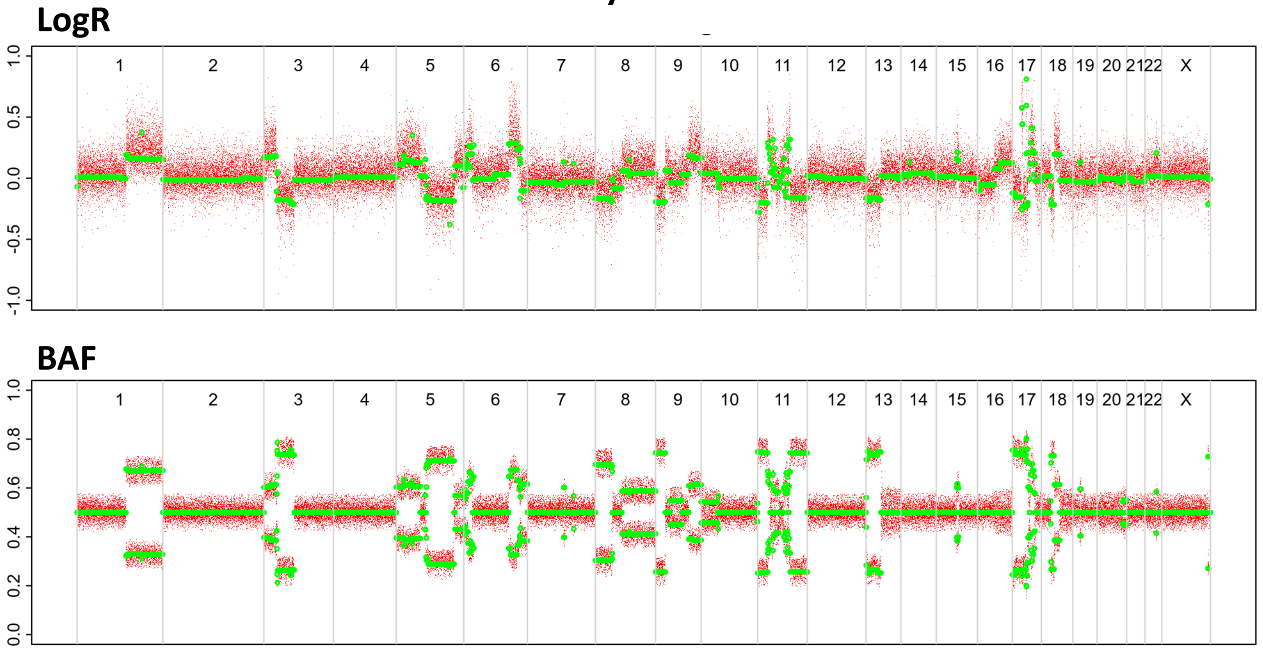

2nd event

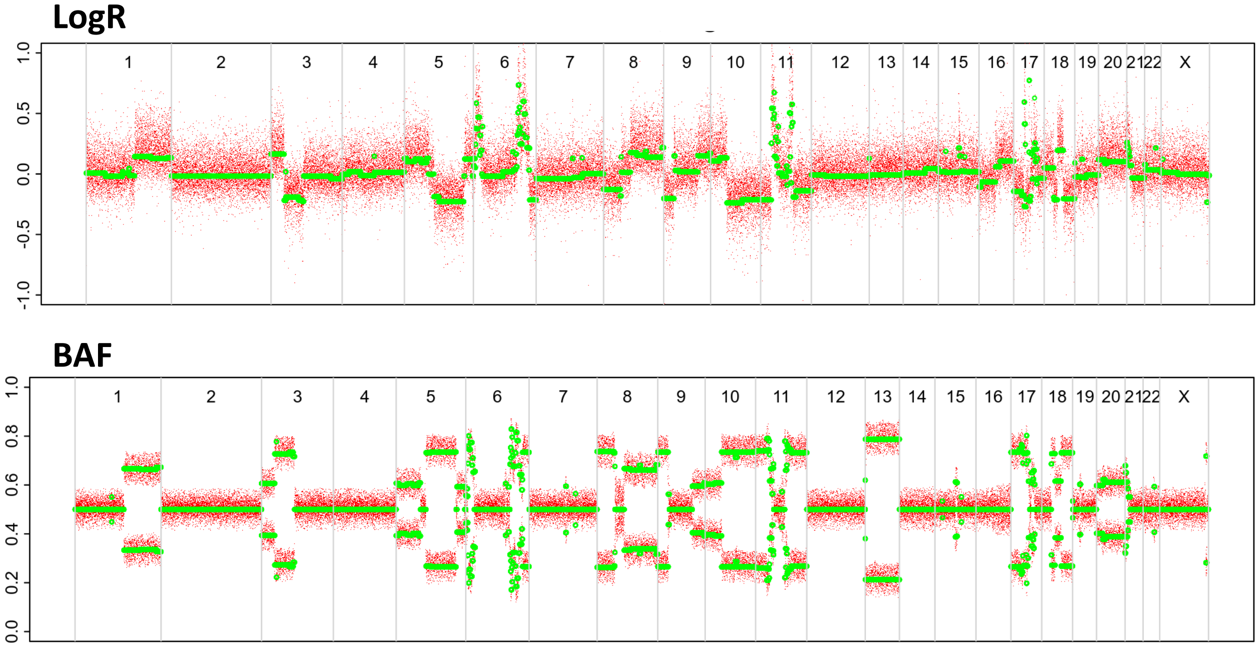

P075

Syn/Meta	Time from 1st surgery to 2nd event (Months)	Side 2nd event	Histology 2nd event	Surgery	AdjuvanT Treatment Pri (RT/ HT)	ER Pri	ER 2nd event	Her2 Pri	Her2 2nd event	Grade Pri	Grade 2nd event	Quadrant 2nd event	Margins	Screening	Clonality P value Copy N	Clonality P value Panel seq	Clonality P value WES	Final verdict
metachronous	31	Ipsilateral	IDC with DCIS*	lumpectomy	None	+	NA	NA	NA	3	2	at or adjacent to primary	Clear	screen-detected	0.0004662	NA	NA	Related

Clonality P value for ipsilateral synchronous 2nd event Copy Number
0.004662

Primary event

LogR

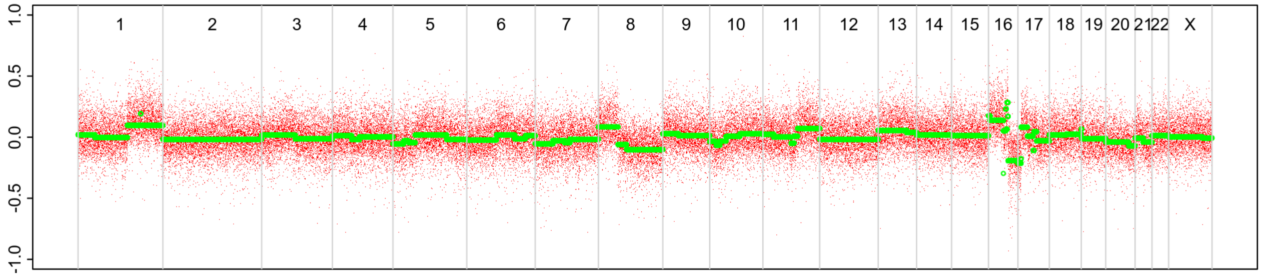

BAF

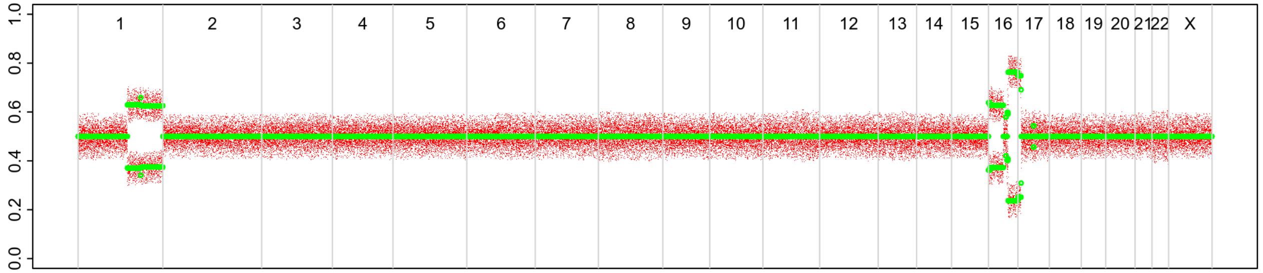

2nd event (INV)

LogR

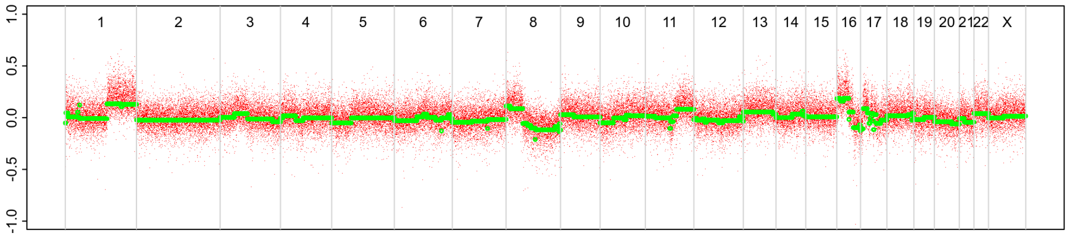

BAF

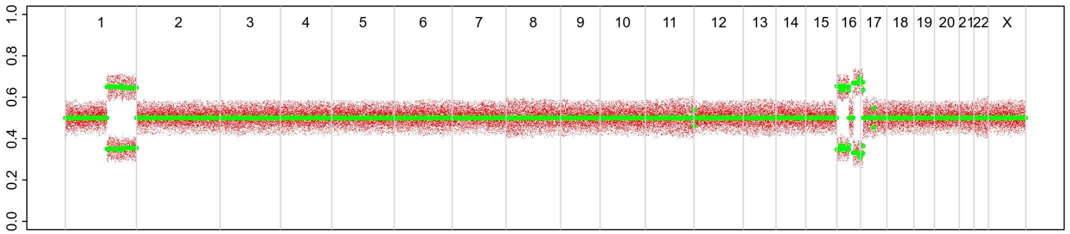

2nd event (DCIS)

LogR

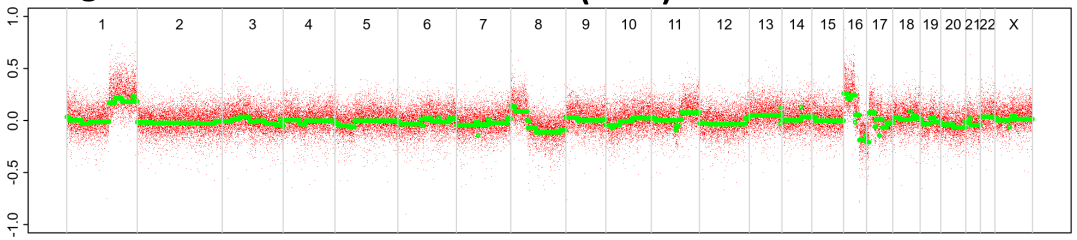

BAF

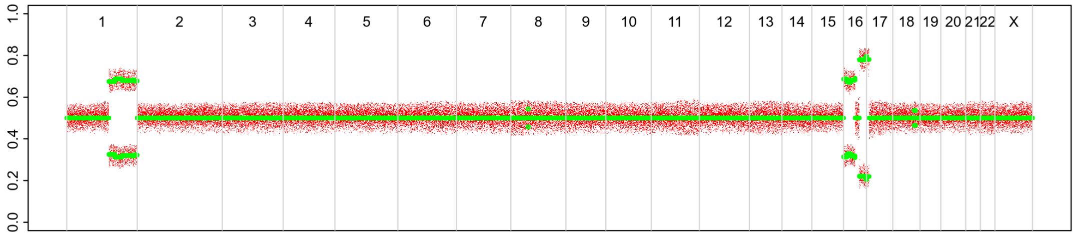

P076

Syn/Meta	Time from 1st surgery to 2nd event (Months)	Side 2nd event	Histology 2nd event	Surgery	AdjuvanT Treatment Pri (RT/ HT)	ER Pri	ER 2nd event	Her2 Pri	Her2 2nd event	Grade Pri	Grade 2nd event	Quadrant 2nd event	Margins	Screening	Clonality P value Copy N	Clonality P value Panel seq	Clonality P value WES	Final verdict
metachronous	49	Ipsilateral	IDC with DCIS	lumpectomy	None	+	+	-	NA	3	2	at or adjacent to primary	Clear	screen-detected	0.000466 2	0	NA	Related

Primary event

LogR

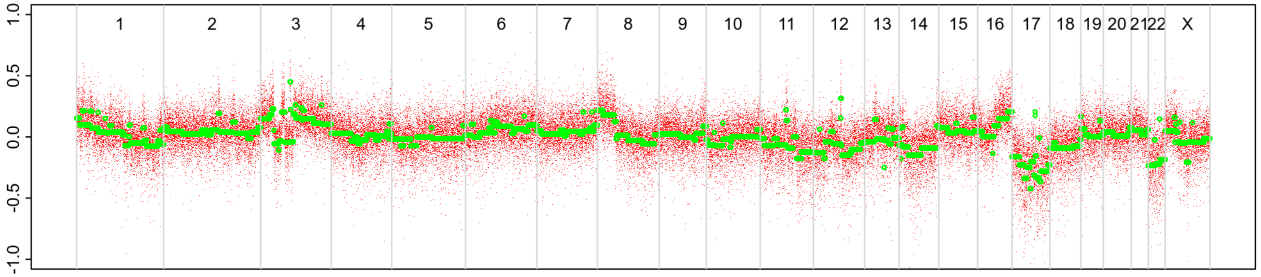

BAF

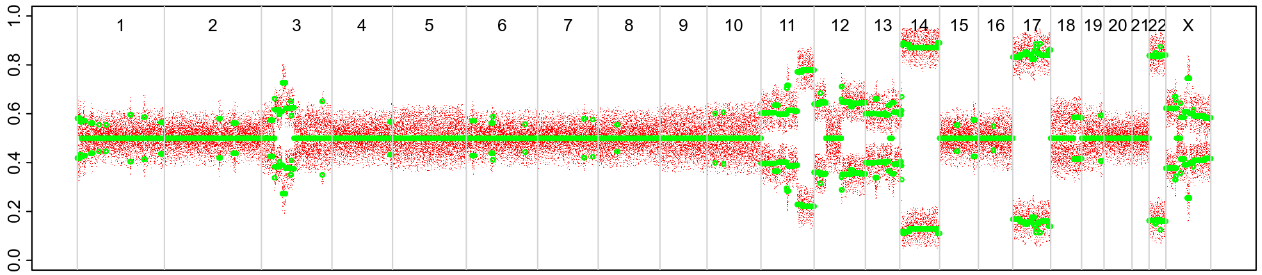

2nd event

LogR

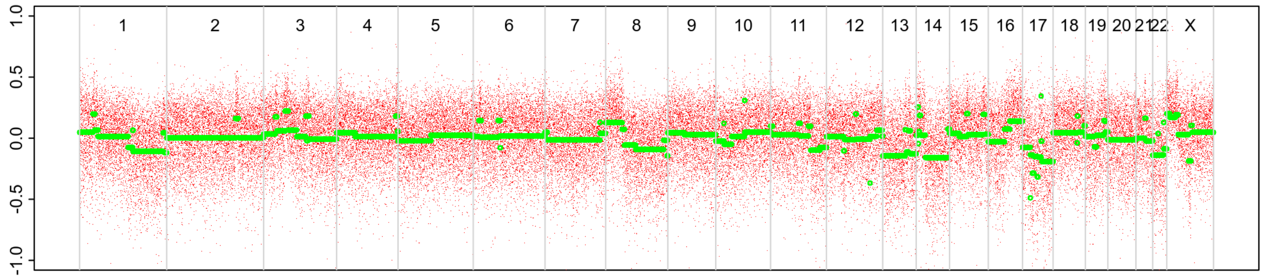

BAF

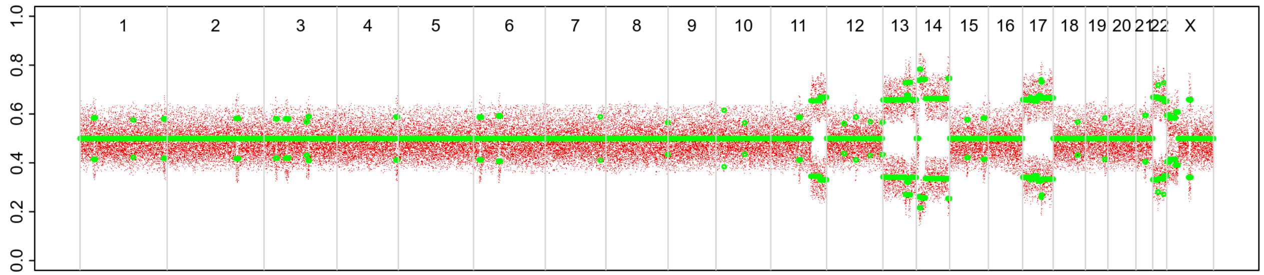

P077

Syn/Meta	Time from 1st surgery to 2nd event (Months)	Side 2nd event	Histology 2nd event	Surgery	AdjuvanT Treatment Pri (RT/ HT)	ER Pri	ER 2nd event	Her2 Pri	Her2 2nd event	Grade Pri	Grade 2nd event	Quadrant 2nd event	Margins	Screening	Clonality P value Copy N	Clonality P value Panel seq	Clonality P value WES	Final verdict
metachronous	54	Ipsilateral	IDC with DCIS*	lumpectomy	RT	+	+	-	NA	3	3	at or adjacent to primary	Clear	screen-detected	0.000466 2	NA	NA	Related

Clonality P value for ipsilateral synchronous 2nd event Copy Number
0.004662

Primary event

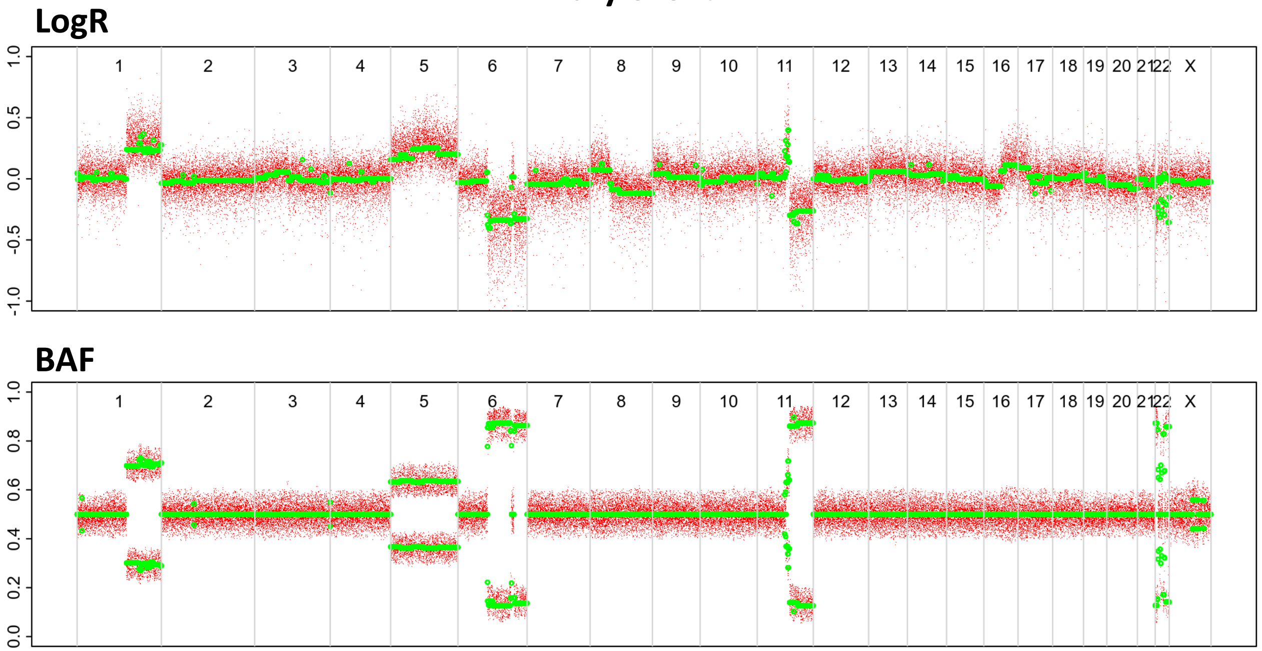

2nd event (INV)

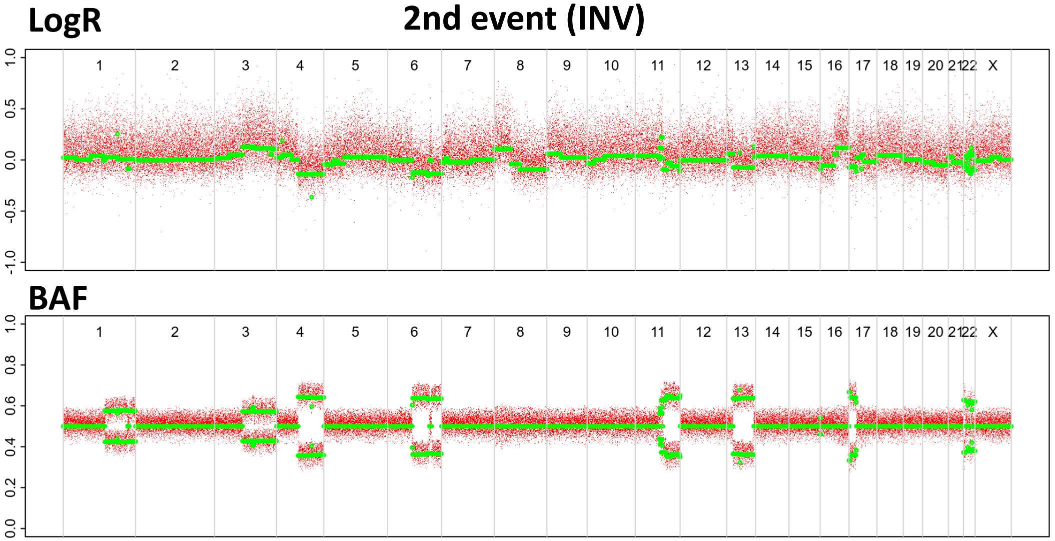

2nd event (DCIS)

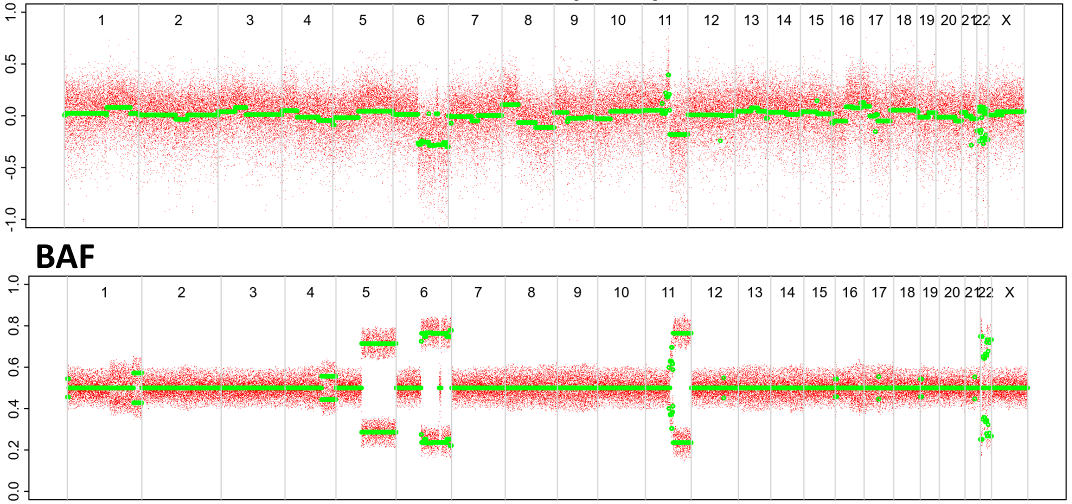

P078

Syn/Meta	Time from 1st surgery to 2nd event (Months)	Side 2nd event	Histology 2nd event	Surgery	AdjuvanT Treatment Pri (RT/ HT)	ER Pri	ER 2nd event	Her2 Pri	Her2 2nd event	Grade Pri	Grade 2nd event	Quadrant 2nd event	Margins	Screening	Clonality P value Copy N	Clonality P value Panel seq	Clonality P value WES	Final verdict
metachronous	51	Ipsilateral	IDC no DCIS	lumpectomy	CTRTHT	+	NA	-	NA	3	1	at or adjacent to primary	Clear	screen-detected	0.0004662	NA	NA	Related

Primary event

LogR

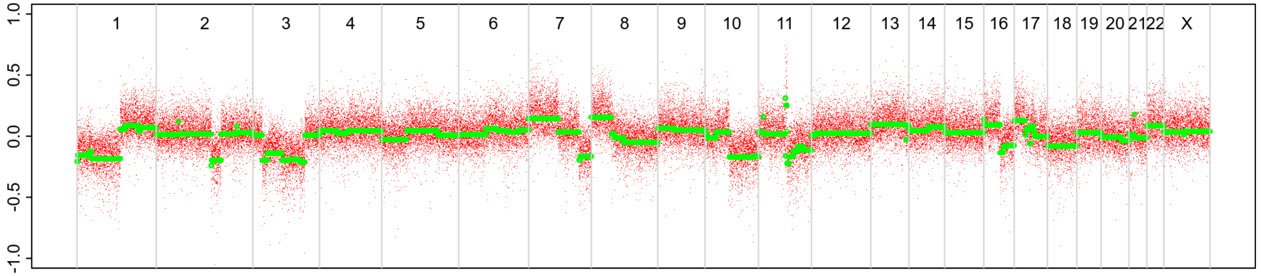

BAF

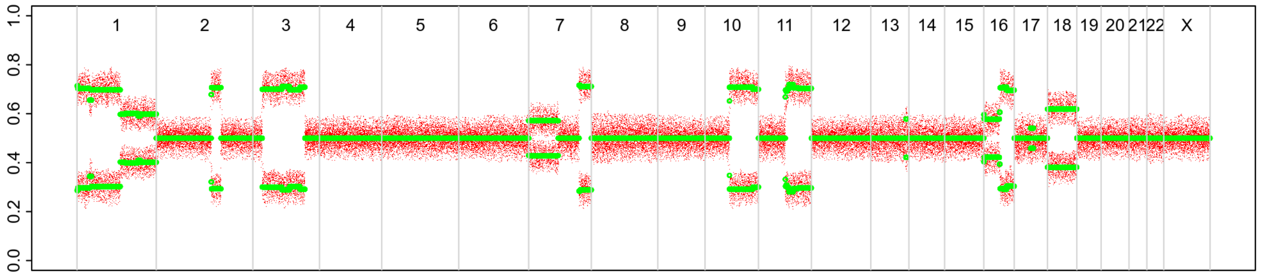

2nd event

LogR

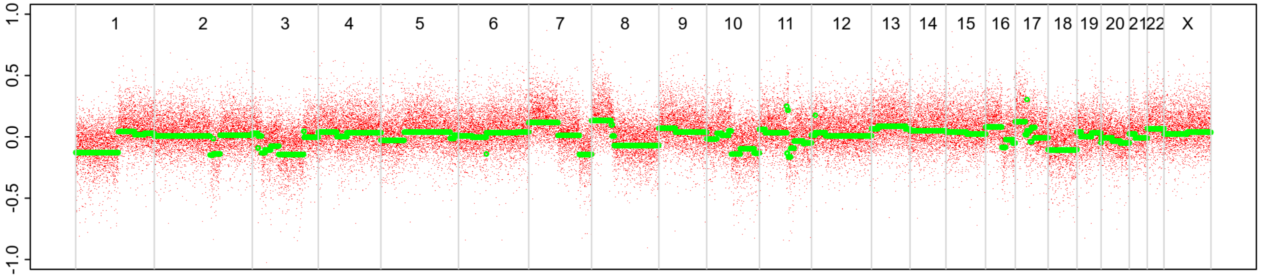

BAF

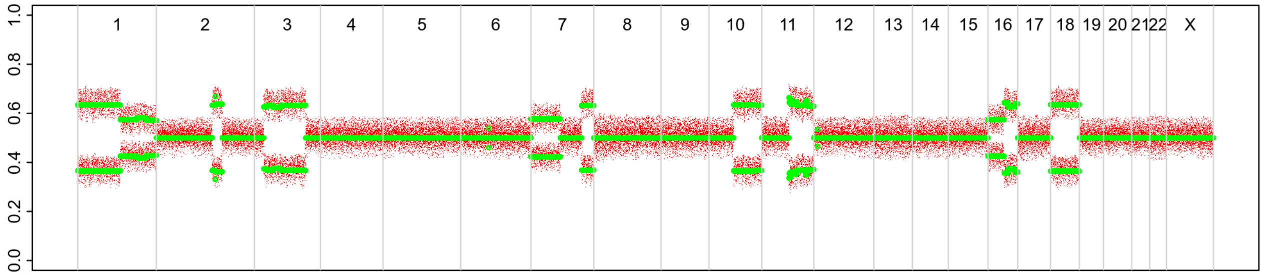

P079

Syn/Meta	Time from 1st surgery to 2nd event (Months)	Side 2nd event	Histology 2nd event	Surgery	AdjuvanT Treatment Pri (RT/ HT)	ER Pri	ER 2nd event	Her2 Pri	Her2 2nd event	Grade Pri	Grade 2nd event	Quadrant 2nd event	Margins	Screening	Clonality P value Copy N	Clonality P value Panel seq	Clonality P value WES	Final verdict
metachronous	38	Ipsilateral	IDC with DCIS*	lumpectomy	None	+	NA	-	-	3	3	at or adjacent to primary	Clear	screen-detected	0.001864802	0	NA	Related

Clonality P value for ipsilateral synchronous 2nd event Copy Number
0.004662

Primary event

LogR

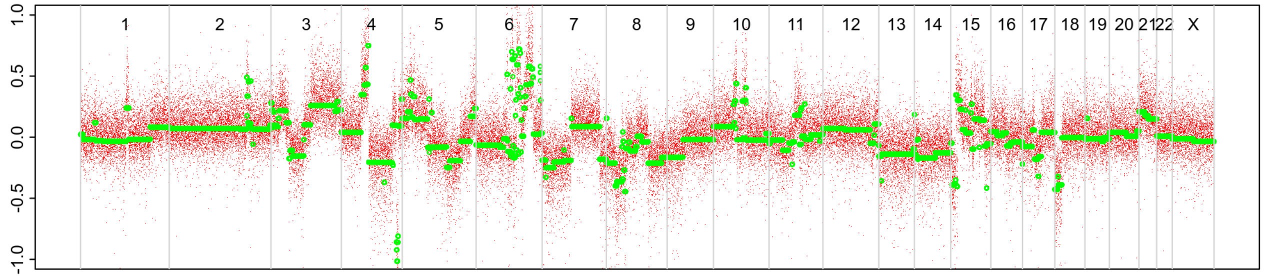

BAF

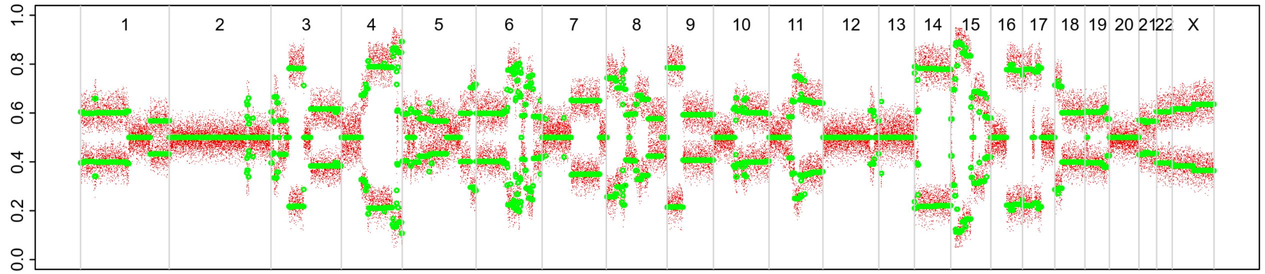

2nd event (INV)

LogR

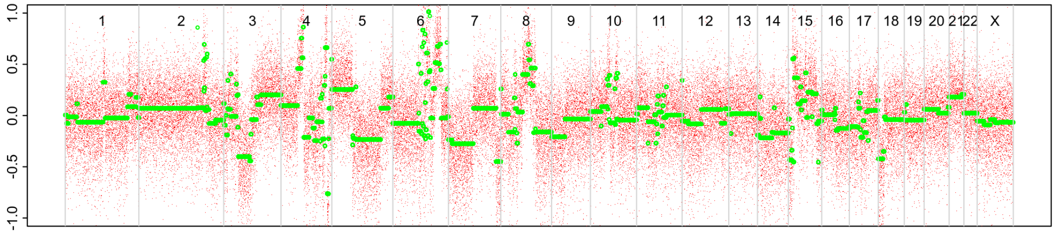

BAF

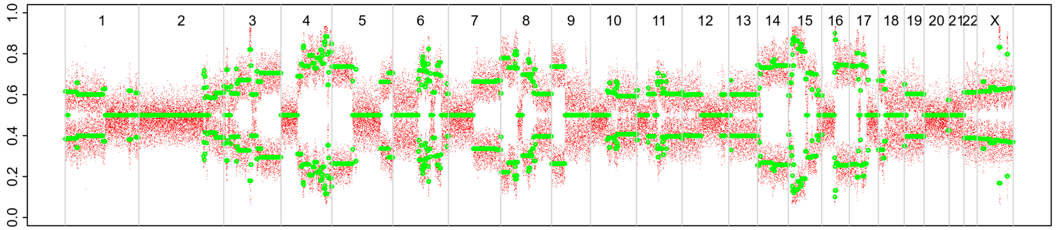

2nd event (DCIS)

LogR

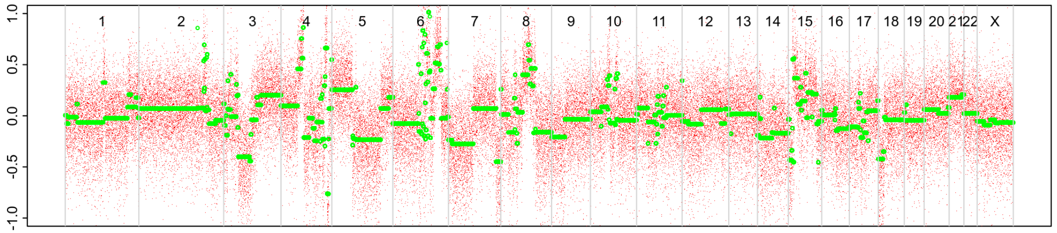

BAF

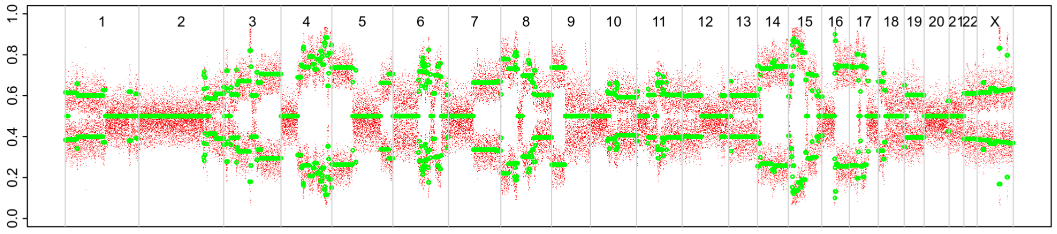

P080

Syn/Meta	Time from 1st surgery to 2nd event (Months)	Side 2nd event	Histology 2nd event	Surgery	AdjuvanT Treatment Pri (RT/ HT)	ER Pri	ER 2nd event	Her2 Pri	Her2 2nd event	Grade Pri	Grade 2nd event	Quadrant 2nd event	Margins	Screening	Clonality P value Copy N	Clonality P value Panel seq	Clonality P value WES	Final verdict
metachronous	35	Ipsilateral	IDC with DCIS	lumpectomy	RT	+	NA	-	NA	3	3	at or adjacent to primary	Clear	screen-detected	0.043822844	0.004036327	NA	Related

Primary event

LogR

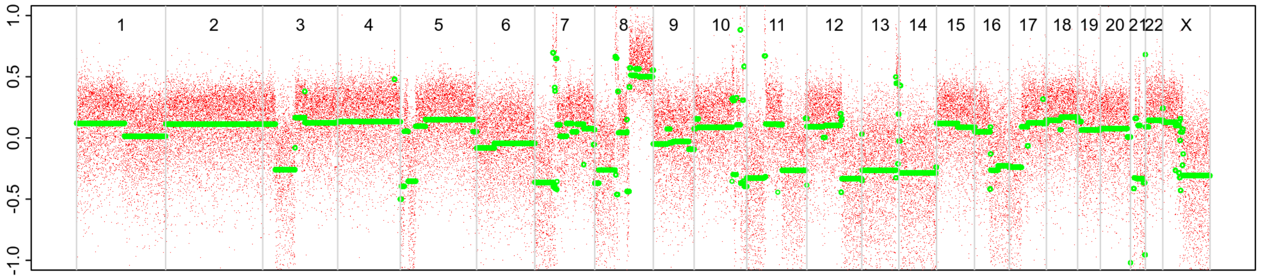

BAF

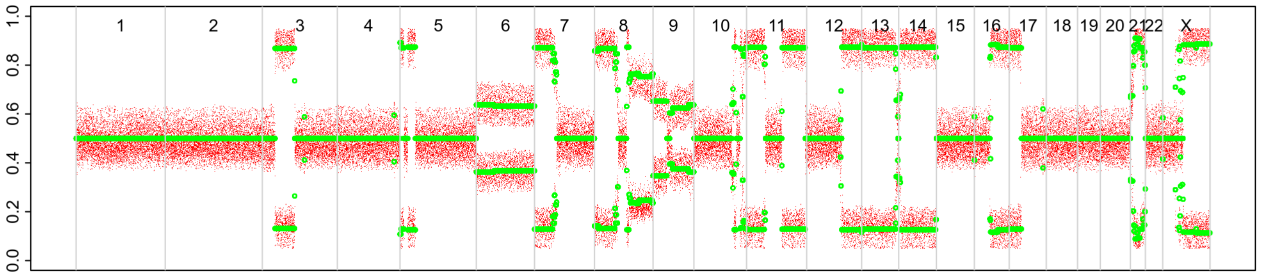

2nd event

LogR

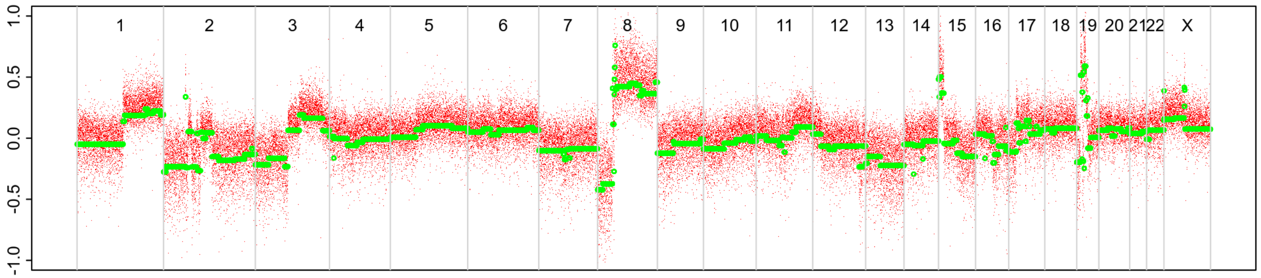

BAF

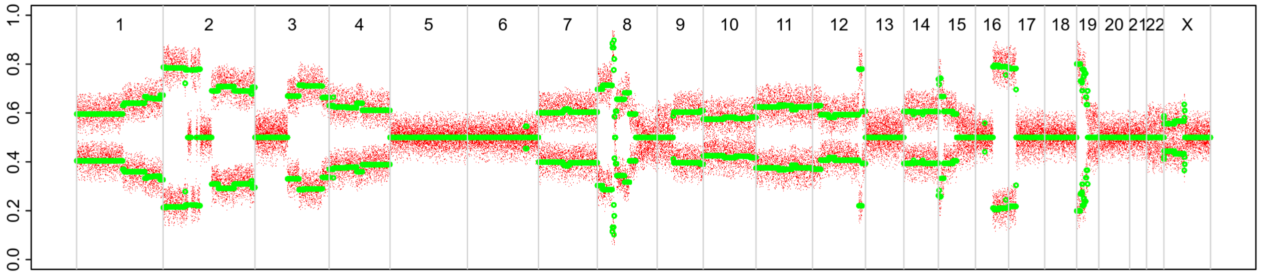

P092

Syn/Meta	Time from 1st surgery to 2nd event (Months)	Side 2nd event	Histology 2nd event	Surgery	AdjuvanT Treatment Pri (RT/ HT)	ER Pri	ER 2nd event	Her2 Pri	Her2 2nd event	Grade Pri	Grade 2nd event	Quadrant 2nd event	Margins	Screening	Clonality P value Copy N	Clonality P value Panel seq	Clonality P value WES	Final verdict
metachronous	22	Ipsilateral	IDC with DCIS*	lumpectomy	RT	+	+	NA	NA	2	2	at or adjacent to primary	Clear	screen-detected	0.000466 2	NA	0.00075244 5	Related

Clonality P value for ipsilateral synchronous 2nd event Copy Number
0.004662

Primary event

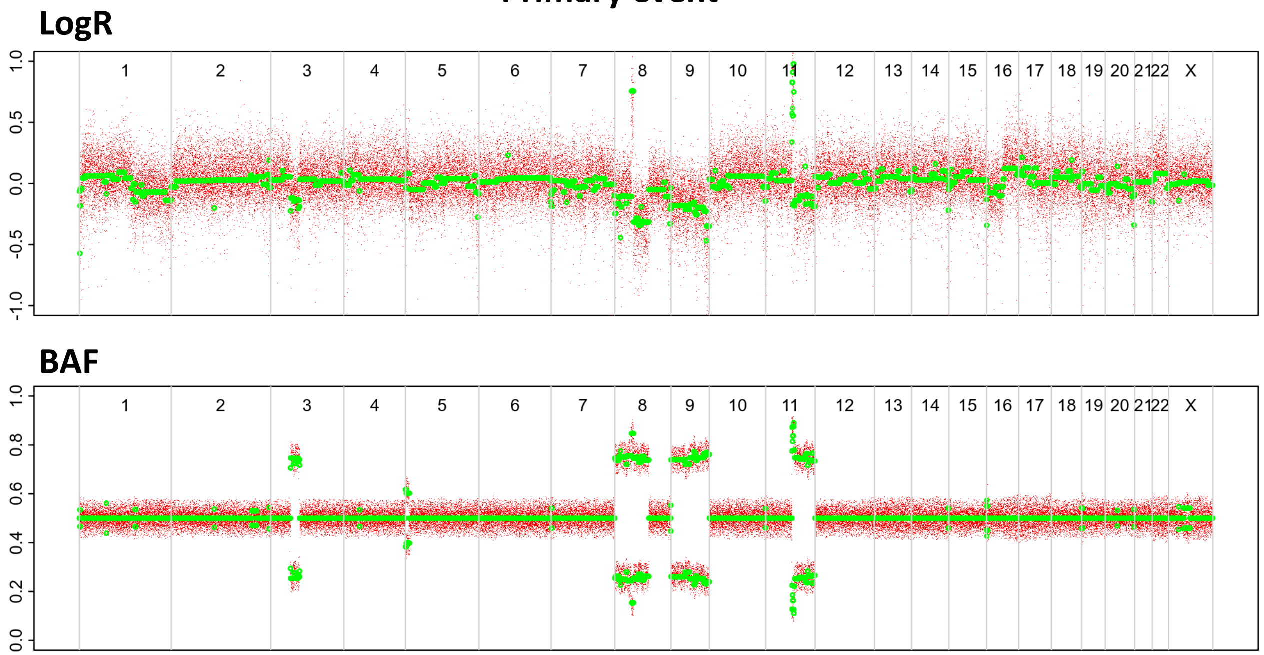

2nd event (INV)

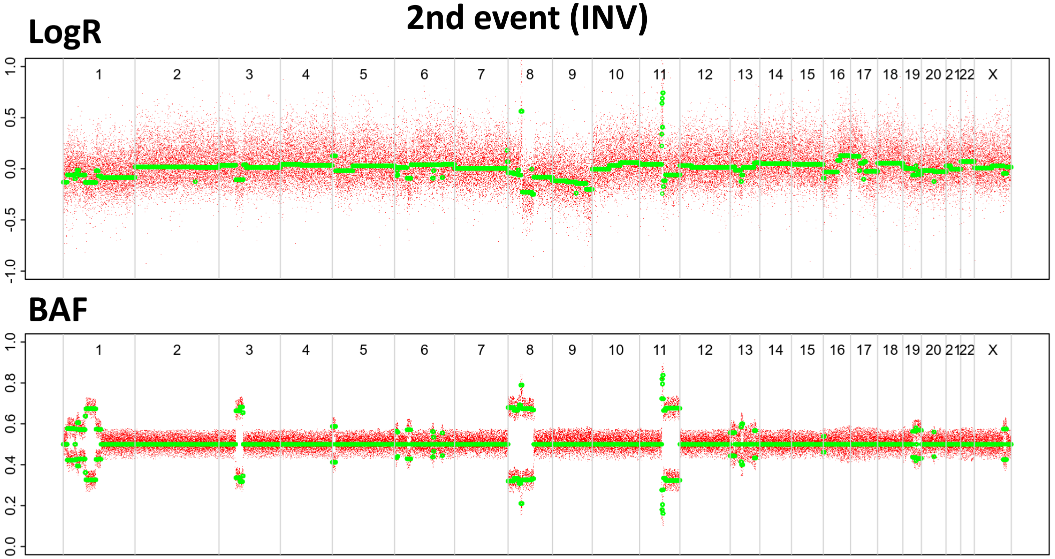

2nd event (DCIS)

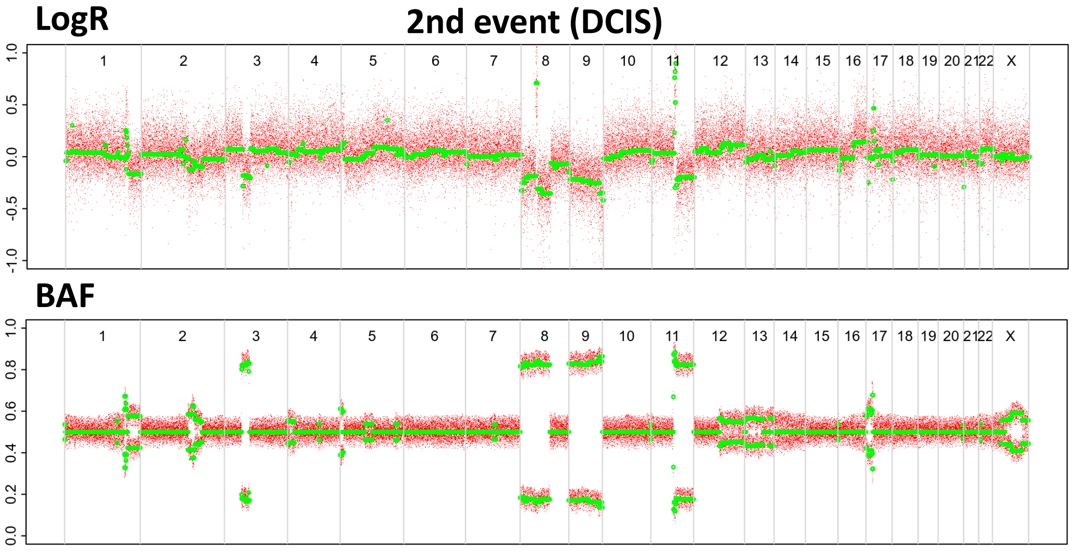

P099

Syn/Meta	Time from 1st surgery to 2nd event (Months)	Side 2nd event	Histology 2nd event	Surgery	AdjuvanT Treatment Pri (RT/ HT)	ER Pri	ER 2nd event	Her2 Pri	Her2 2nd event	Grade Pri	Grade 2nd event	Quadrant 2nd event	Margins	Screening	Clonality P value Copy N	Clonality P value Panel seq	Clonality P value WES	Final verdict
metachronous	22	Ipsilateral	IDC no DCIS	lumpectomy	None	+	+	-	-	3	3	at or adjacent to primary	Clear	screen-detected	0.000466 2	0	NA	Related

Primary event

LogR

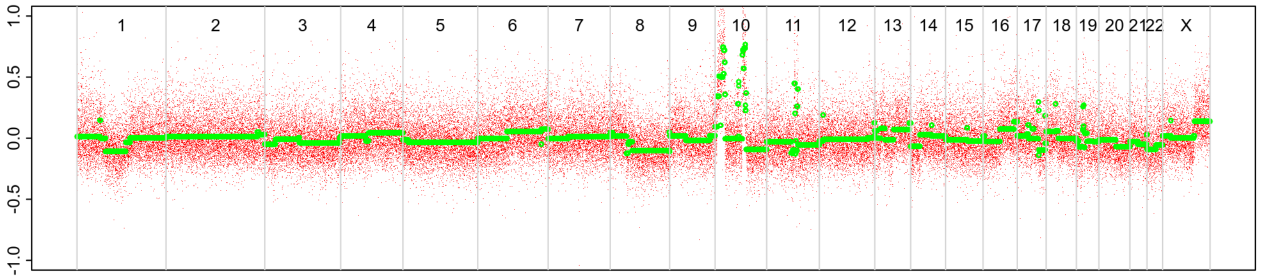

BAF

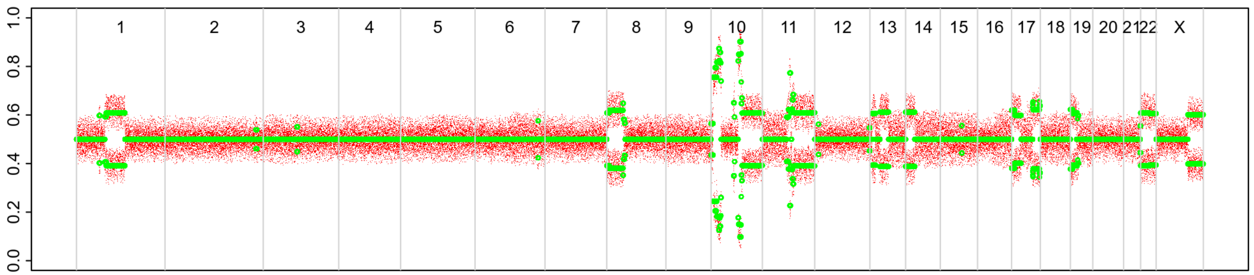

2nd event

LogR

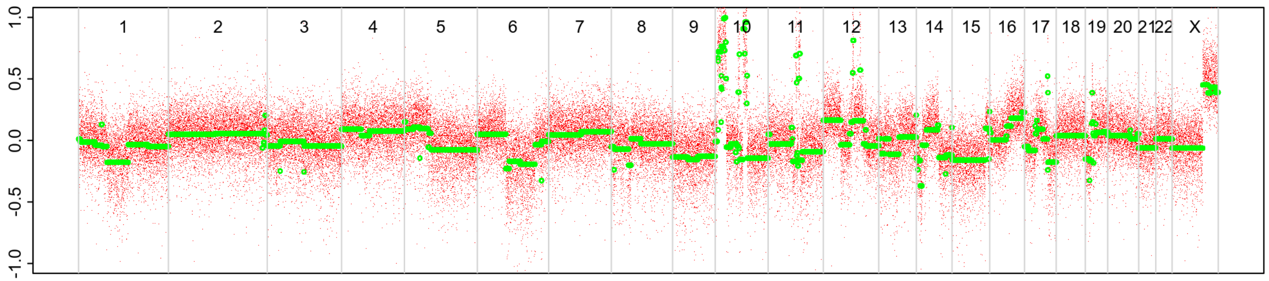

BAF

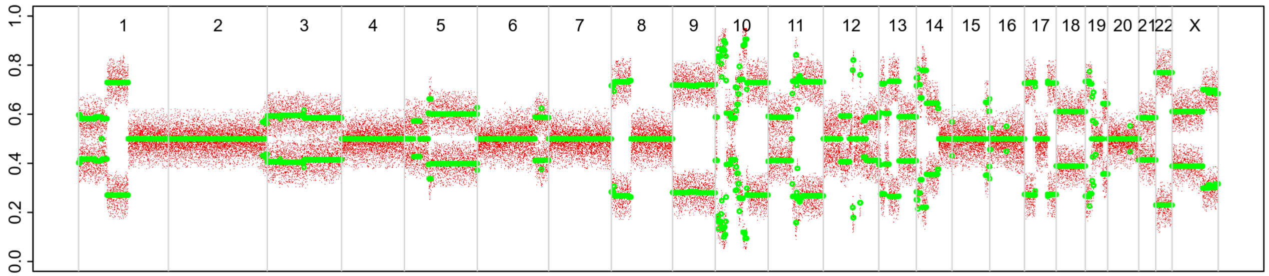

P100

Syn/Meta	Time from 1st surgery to 2nd event (Months)	Side 2nd event	Histology 2nd event	Surgery	AdjuvanT Treatment Pri (RT/ HT)	ER Pri	ER 2nd event	Her2 Pri	Her2 2nd event	Grade Pri	Grade 2nd event	Quadrant 2nd event	Margins	Screening	Clonality P value Copy N	Clonality P value Panel seq	Clonality P value WES	Final verdict
metachronous	59	Ipsilateral	DCIS only	lumpectomy	None	+	+	+	NA	3	3	NA	Clear	screen-detected	0.014652015	NA	NA	Equivocal

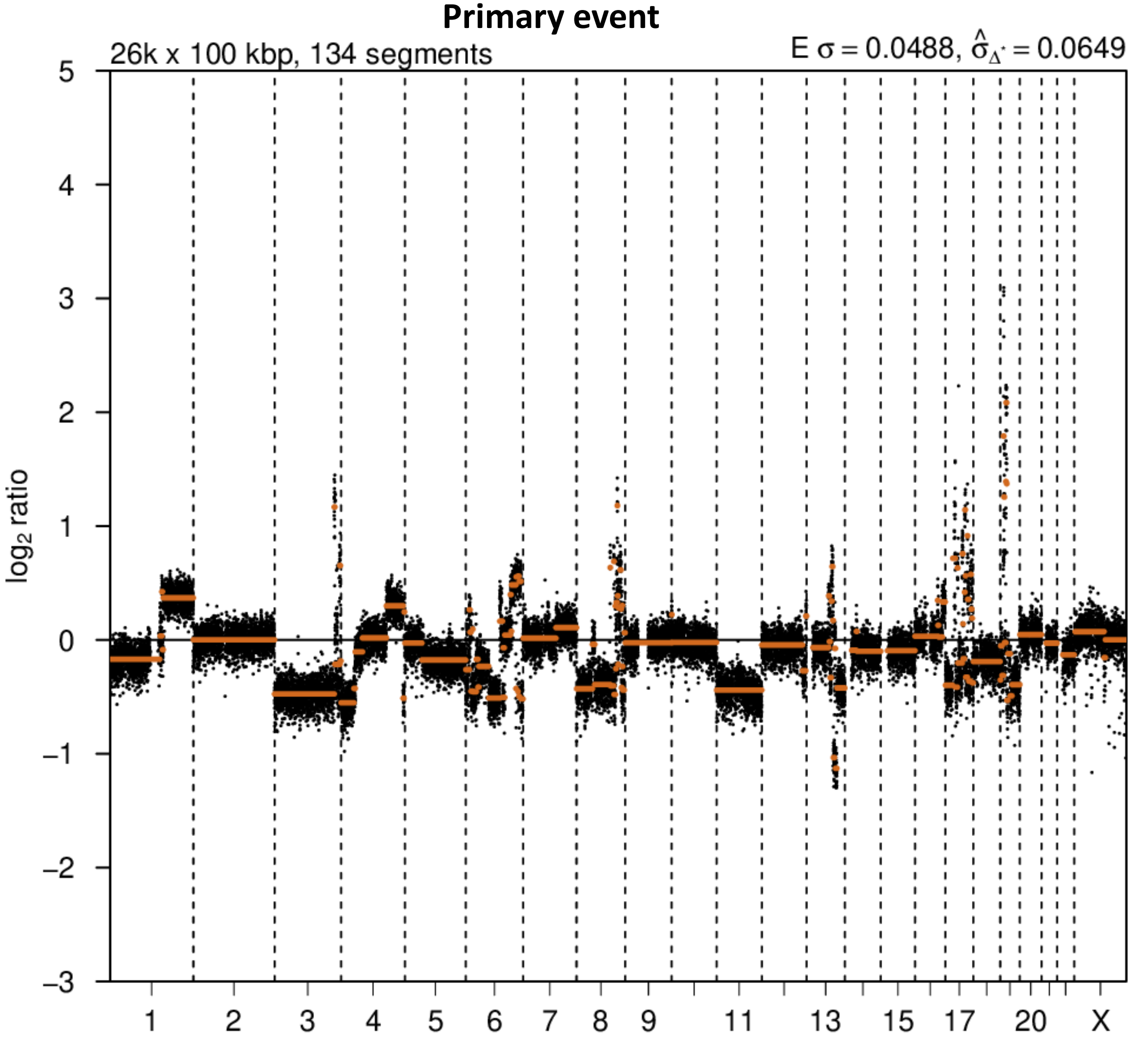

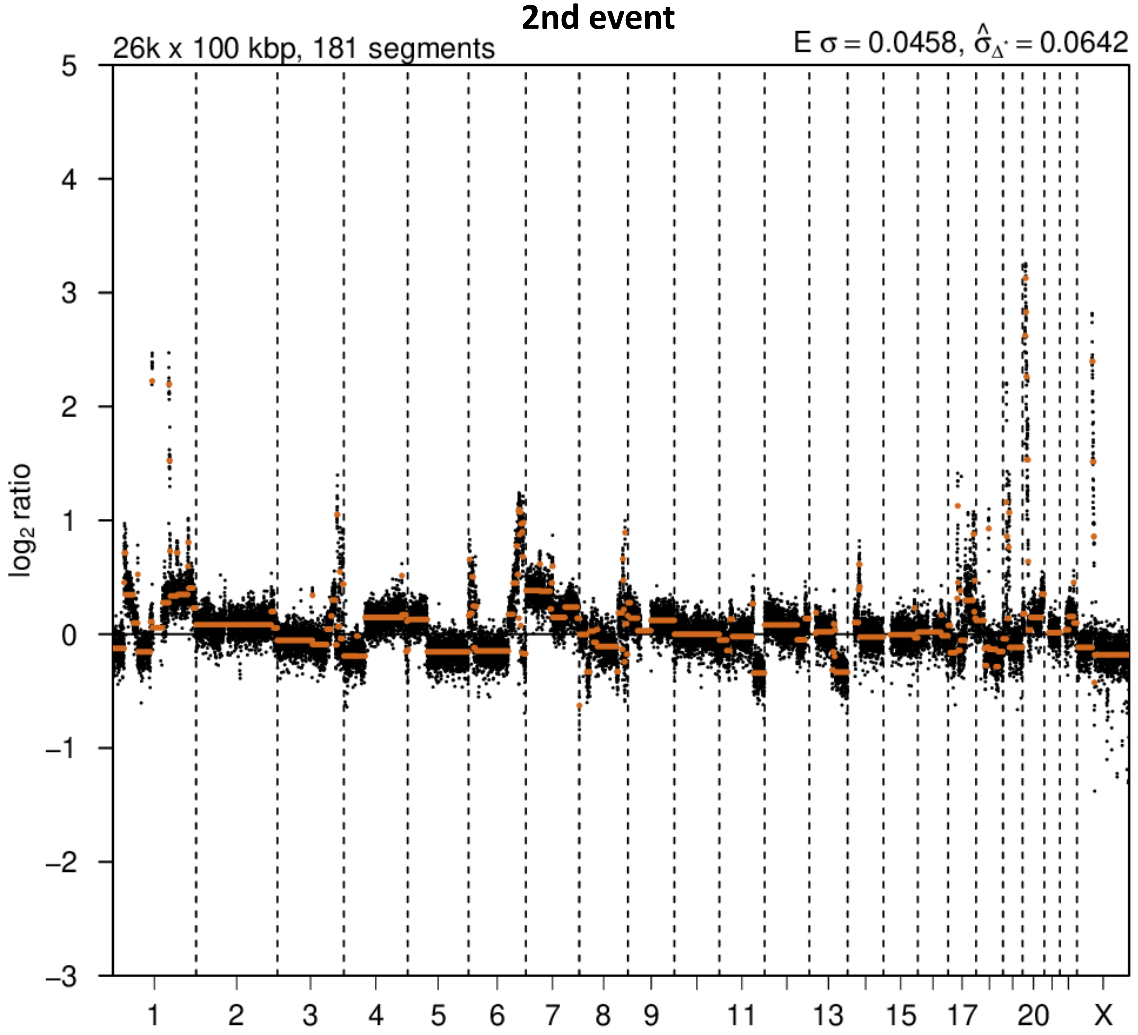

P101

Syn/Meta	Time from 1st surgery to 2nd event (Months)	Side 2nd event	Histology 2nd event	Surgery	AdjuvanT Treatment Pri (RT/ HT)	ER Pri	ER 2nd event	Her2 Pri	Her2 2nd event	Grade Pri	Grade 2nd event	Quadrant 2nd event	Margins	Screening	Clonality P value Copy N	Clonality P value Panel seq	Clonality P value WES	Final verdict
metachronous	41	Ipsilateral	DCIS only	lumpectomy	None	NA	NA	NA	NA	3	3	NA	Clear	screen-detected	0.025641026	NA	NA	Equivocal

Primary event

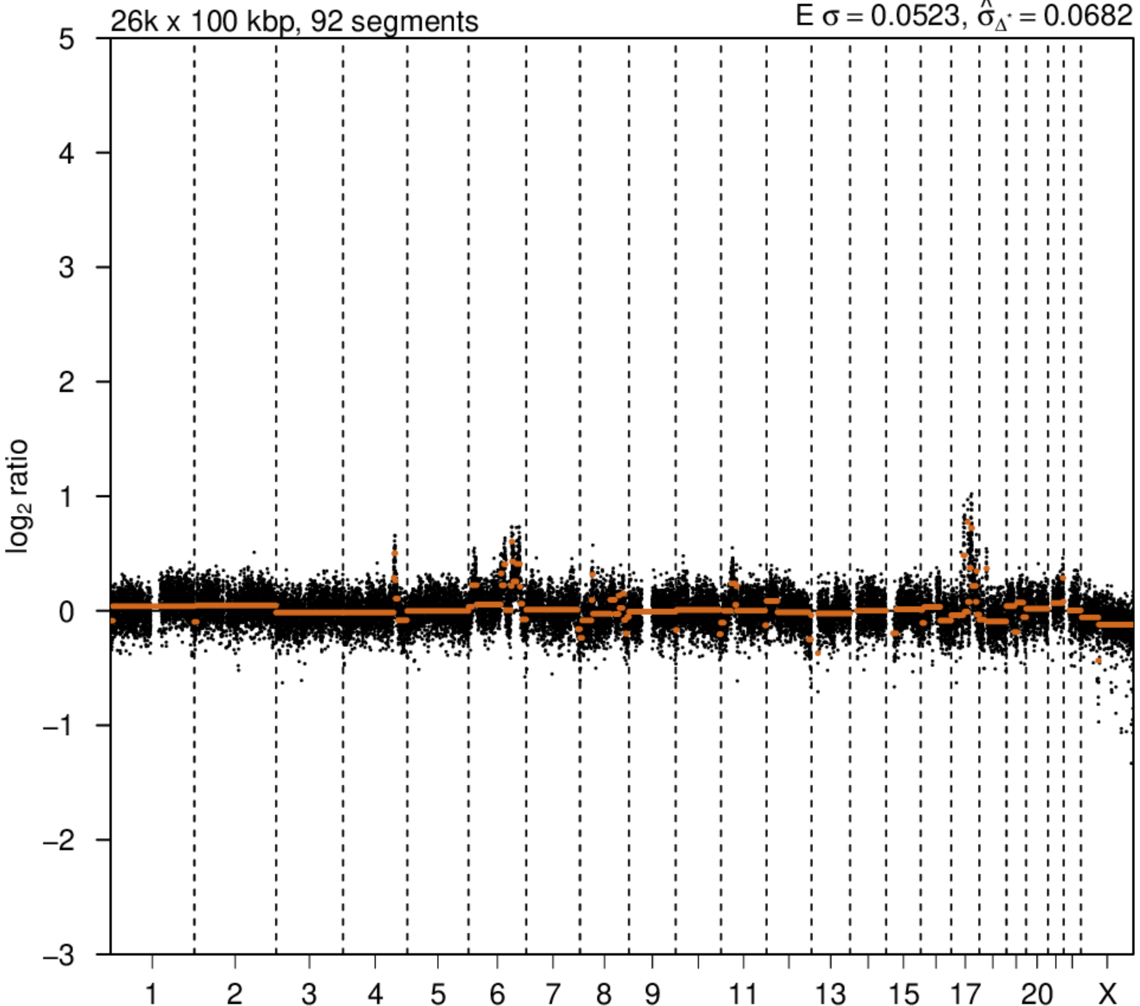

2nd event

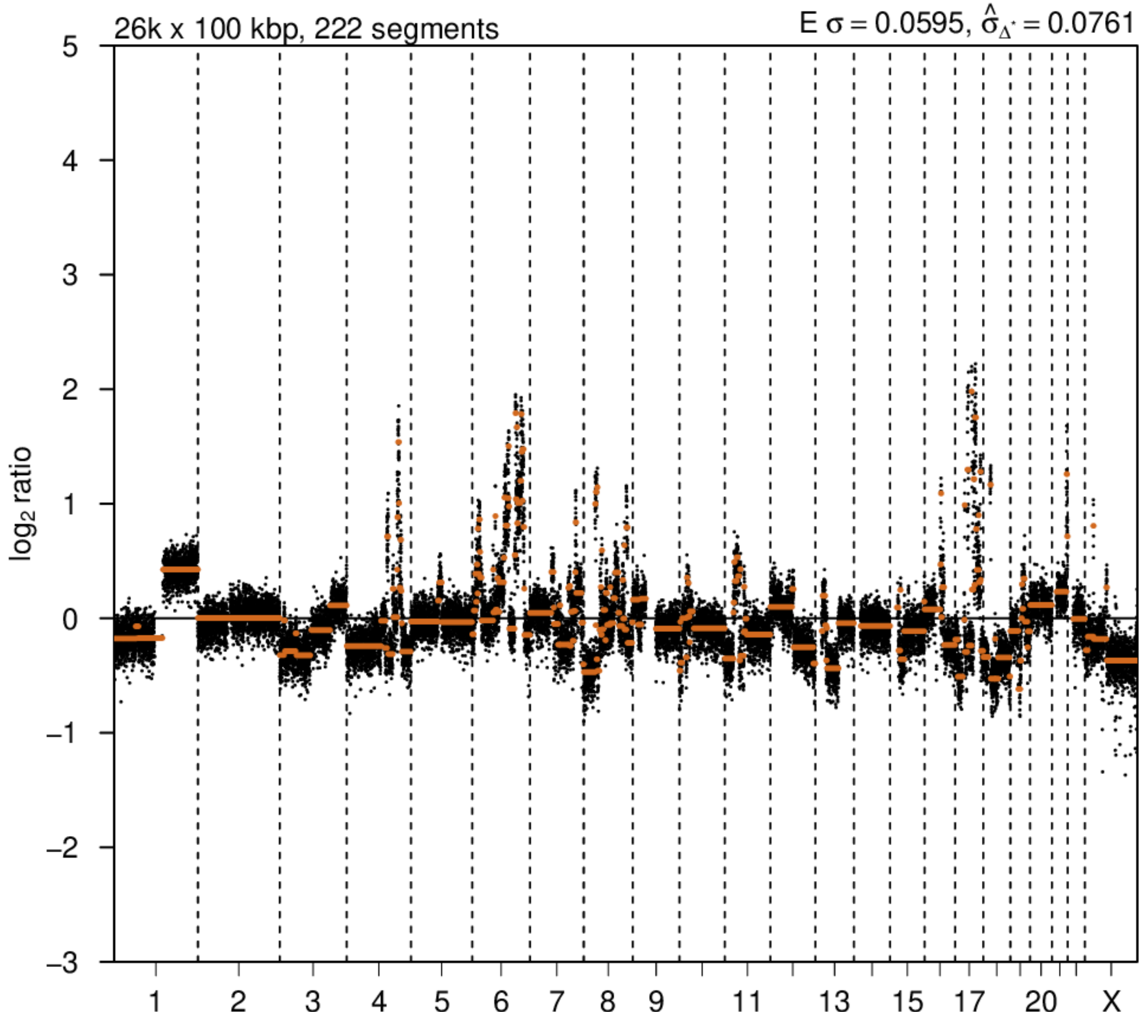

P108

Syn/Meta	Time from 1st surgery to 2nd event (Months)	Side 2nd event	Histology 2nd event	Surgery	AdjuvanT Treatment Pri (RT/ HT)	ER Pri	ER 2nd event	Her2 Pri	Her2 2nd event	Grade Pri	Grade 2nd event	Quadrant 2nd event	Margins	Screening	Clonality P value Copy N	Clonality P value Panel seq	Clonality P value WES	Final verdict
metachronous	74	Ipsilateral	IDC with DCIS*	lumpectomy	RT	+	+	-	-	3	3	at or adjacent to primary	Clear	screen-detected	0.009324<br>009	NA	NA	Related

Clonality P value for ipsilateral synchronous 2nd event Copy Number
0.004662

Primary event

LogR

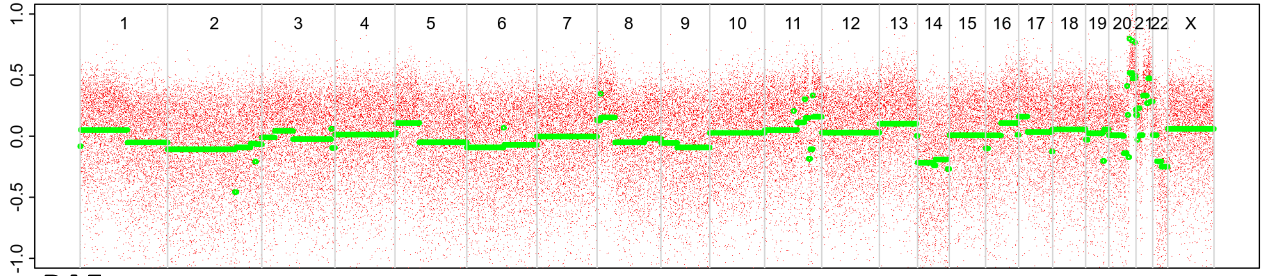

BAF

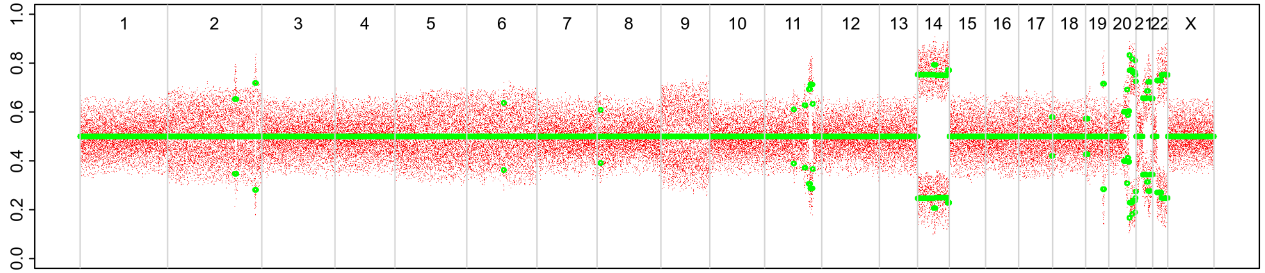

2nd event (INV)

LogR

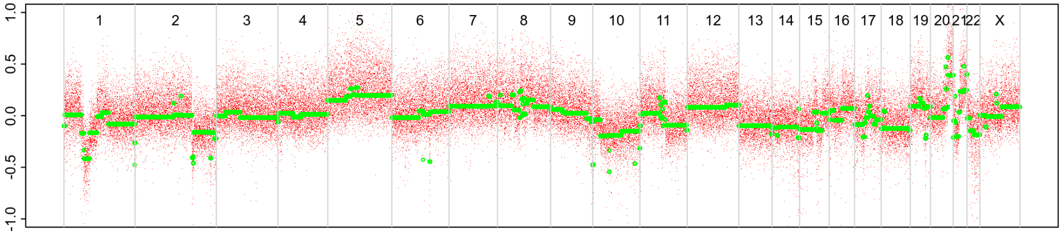

BAF

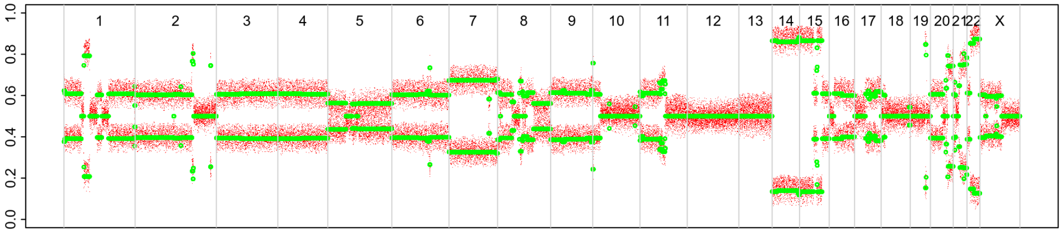

2nd event (DCIS)

LogR

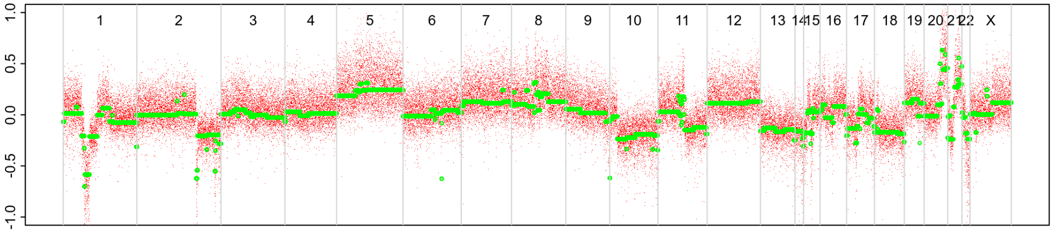

BAF

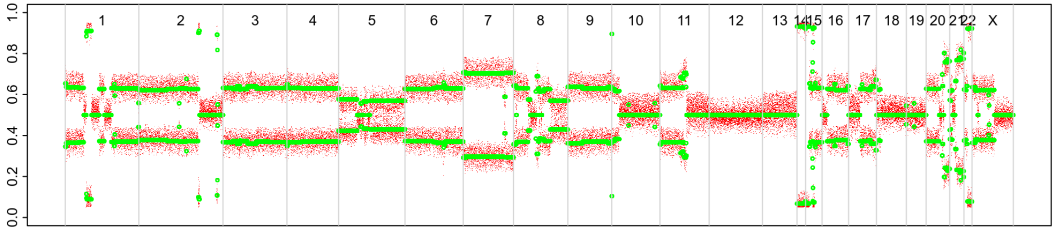

P112

Syn/Meta	Time from 1st surgery to 2nd event (Months)	Side 2nd event	Histology 2nd event	Surgery	AdjuvanT Treatment Pri (RT/ HT)	ER Pri	ER 2nd event	Her2 Pri	Her2 2nd event	Grade Pri	Grade 2nd event	Quadrant 2nd event	Margins	Screening	Clonality P value Copy N	Clonality P value Panel seq	Clonality P value WES	Final verdict
metachronous	24	Ipsilateral	DCIS only	lumpectomy	None	NA	NA	-	NA	3	3	NA	Clear	screen-detected	0.608058608	NA	NA	Unrelated

Primary event

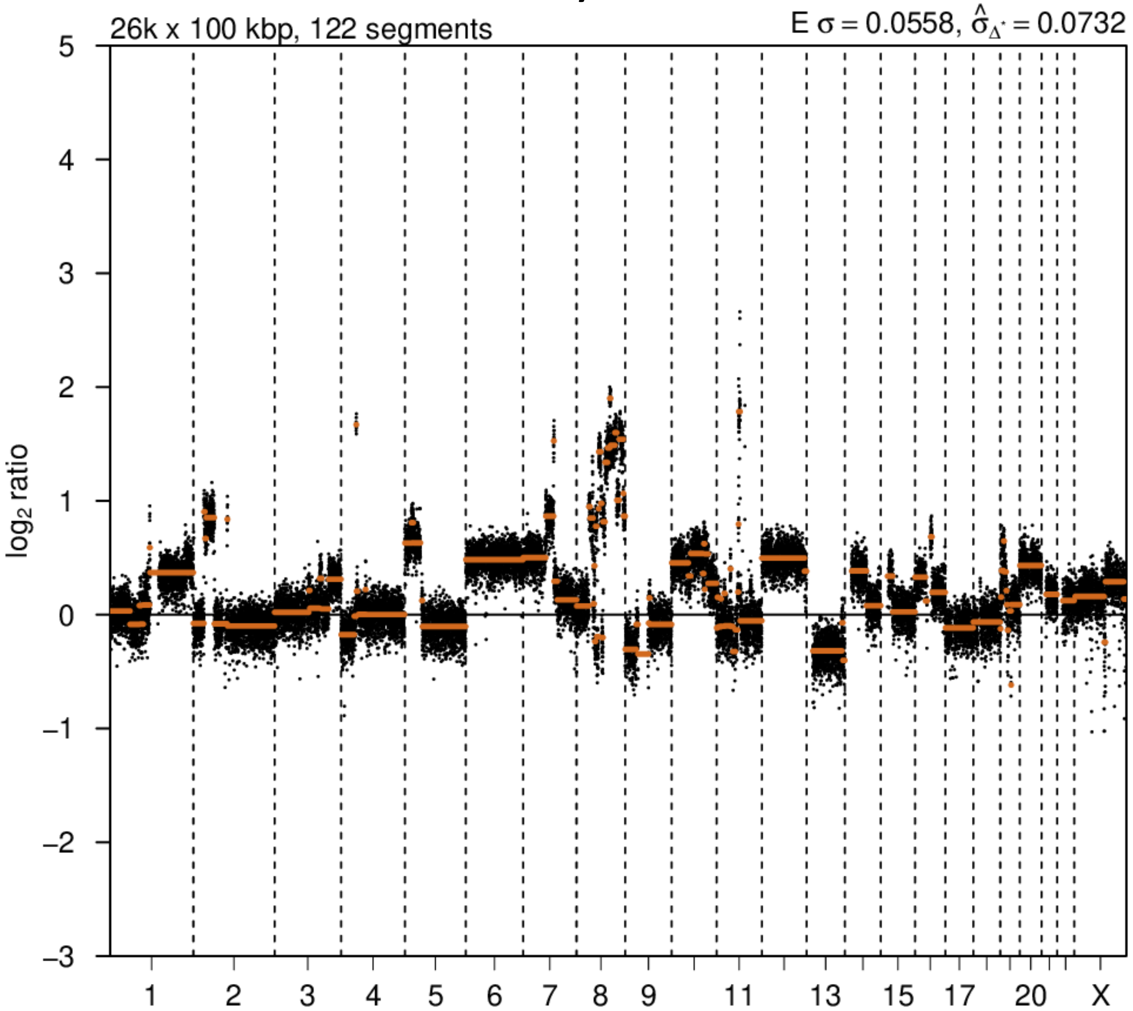

2nd event

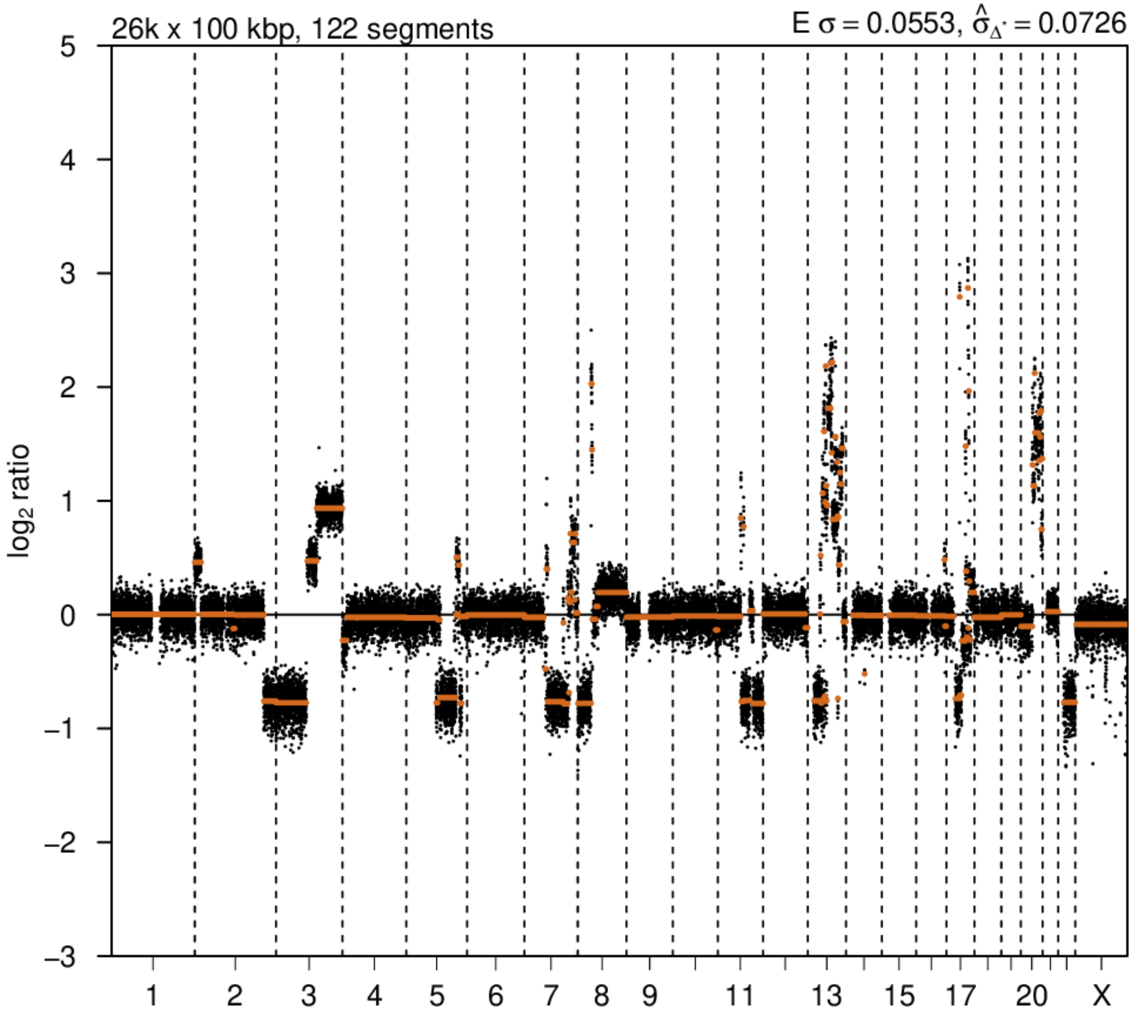

P122

Syn/Meta	Time from 1st surgery to 2nd event (Months)	Side 2nd event	Histology 2nd event	Surgery	AdjuvanT Treatment Pri (RT/ HT)	ER Pri	ER 2nd event	Her2 Pri	Her2 2nd event	Grade Pri	Grade 2nd event	Quadrant 2nd event	Margins	Screening	Clonality P value Copy N	Clonality P value Panel seq	Clonality P value WES	Final verdict
metachronous	88	Ipsilateral	IDC with DCIS	lumpectomy	RT	-	+	+	+	3	3	at or adjacent to primary	Clear	screen-detected	0.158508159	NA	NA	Unrelated

Clonality P value for ipsilateral synchronous 2nd event Copy Number
0.004662

LogR

2nd event (INV)

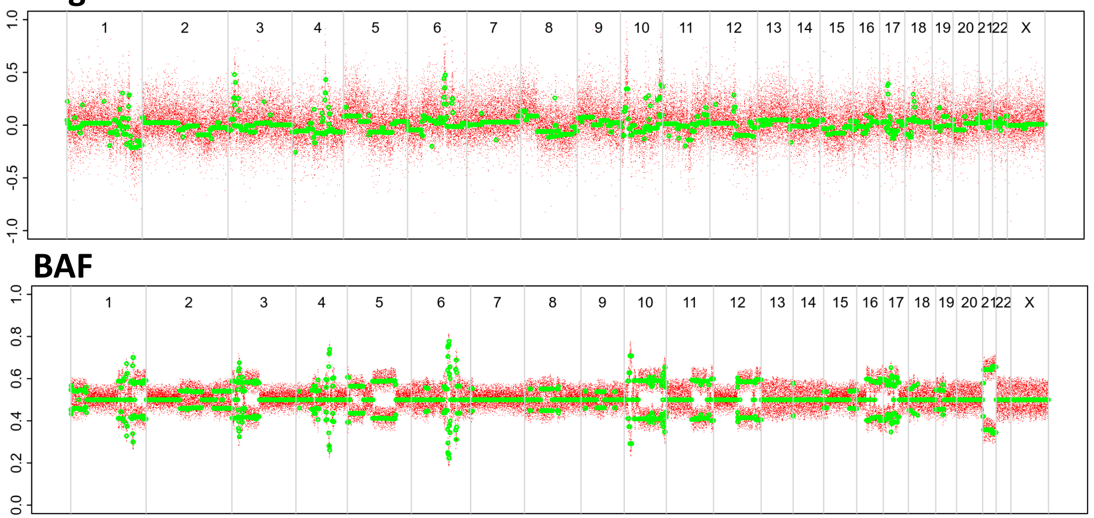

Primary event

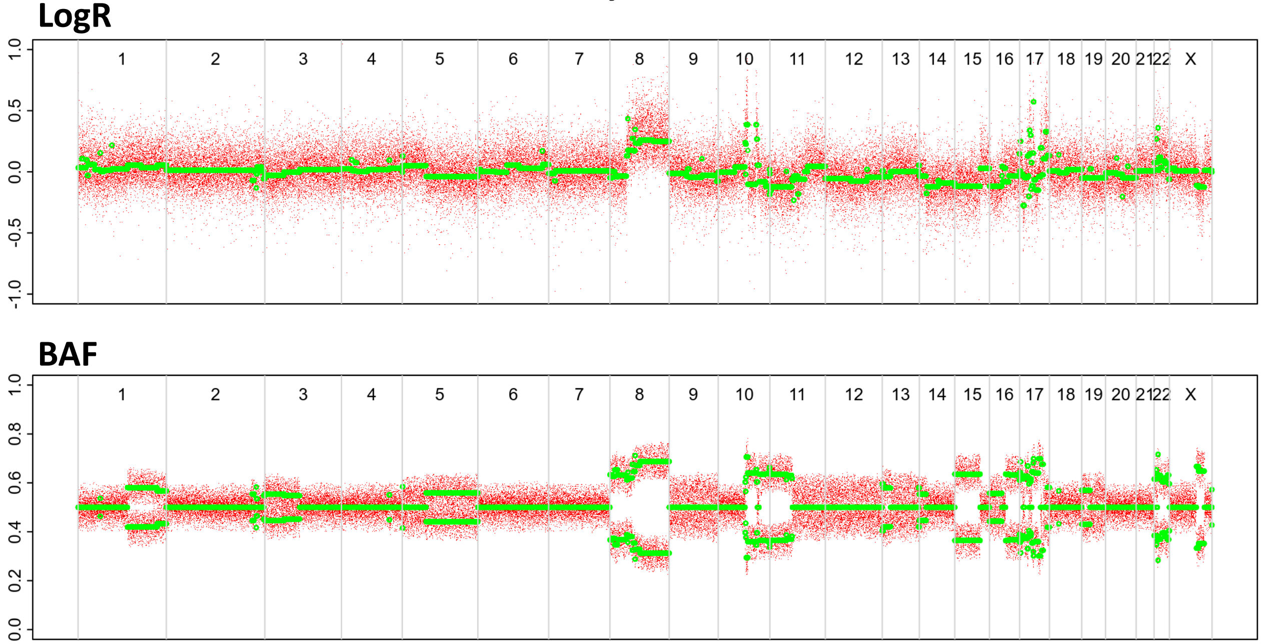

LogR

2nd event (DCIS)

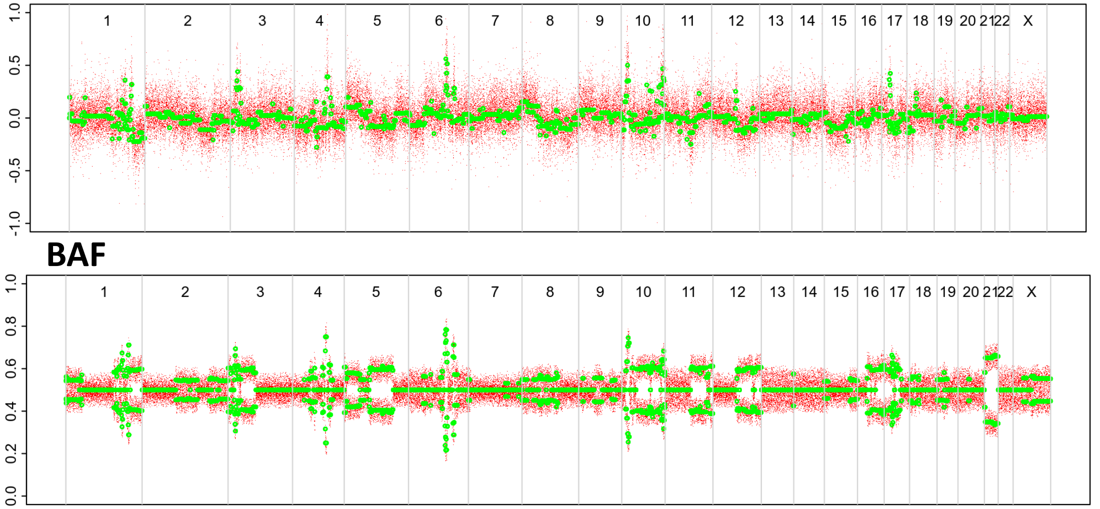

P128

Syn/Meta	Time from 1st surgery to 2nd event (Months)	Side 2nd event	Histology 2nd event	Surgery	AdjuvanT Treatment Pri (RT/ HT)	ER Pri	ER 2nd event	Her2 Pri	Her2 2nd event	Grade Pri	Grade 2nd event	Quadrant 2nd event	Margins	Screening	Clonality P value Copy N	Clonality P value Panel seq	Clonality P value WES	Final verdict
metachronous	24	Ipsilateral	IDC with DCIS*	lumpectomy	None	+	NA	NA	NA	3	2	distant from primary	Clear	screen-detected	0.592540793	1	NA	Unrelated

Clonality P value for ipsilateral synchronous 2nd event Copy Number
0.004662

2nd event (INV)

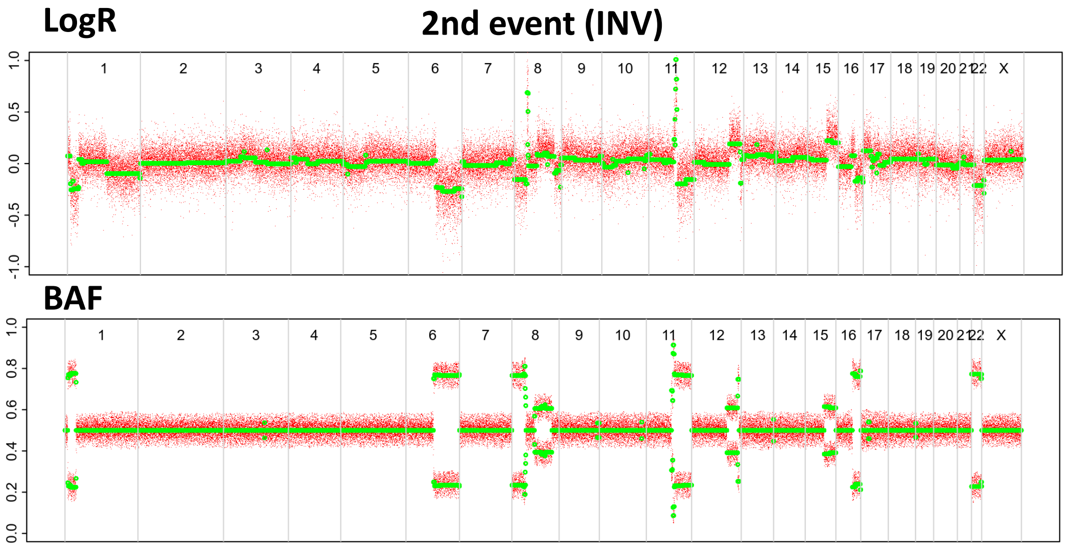

Primary event

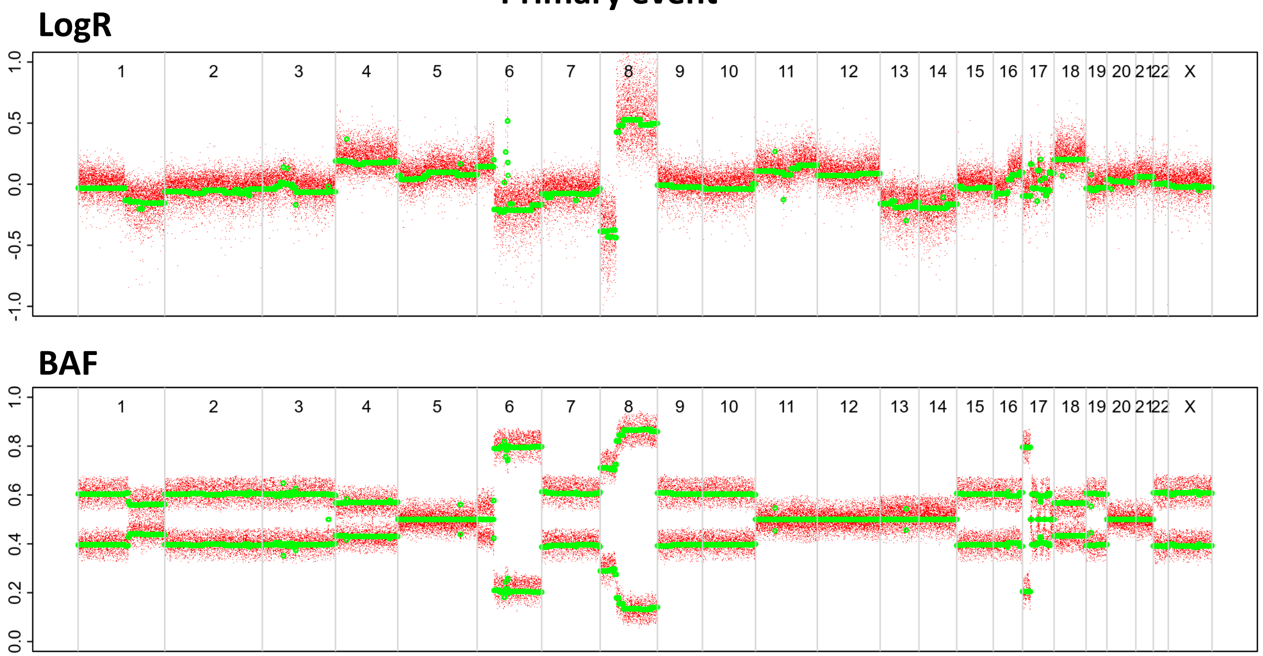

2nd event (DCIS)

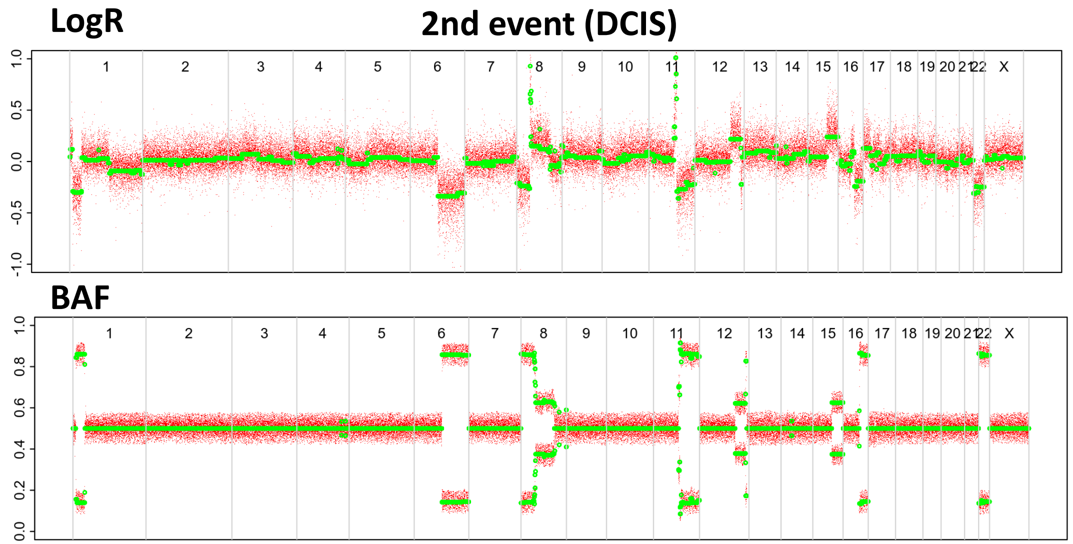

P130

Syn/Meta	Time from 1st surgery to 2nd event (Months)	Side 2nd event	Histology 2nd event	Surgery	AdjuvanT Treatment Pri (RT/ HT)	ER Pri	ER 2nd event	Her2 Pri	Her2 2nd event	Grade Pri	Grade 2nd event	Quadrant 2nd event	Margins	Screening	Clonality P value Copy N	Clonality P value Panel seq	Clonality P value WES	Final verdict
metachronous	34	Contralateral	IDC no DCIS	lumpectomy	None	+	NA	NA	NA	2	2	NA	Clear	screen-detected	1	NA	0.002851033	Related

Primary event

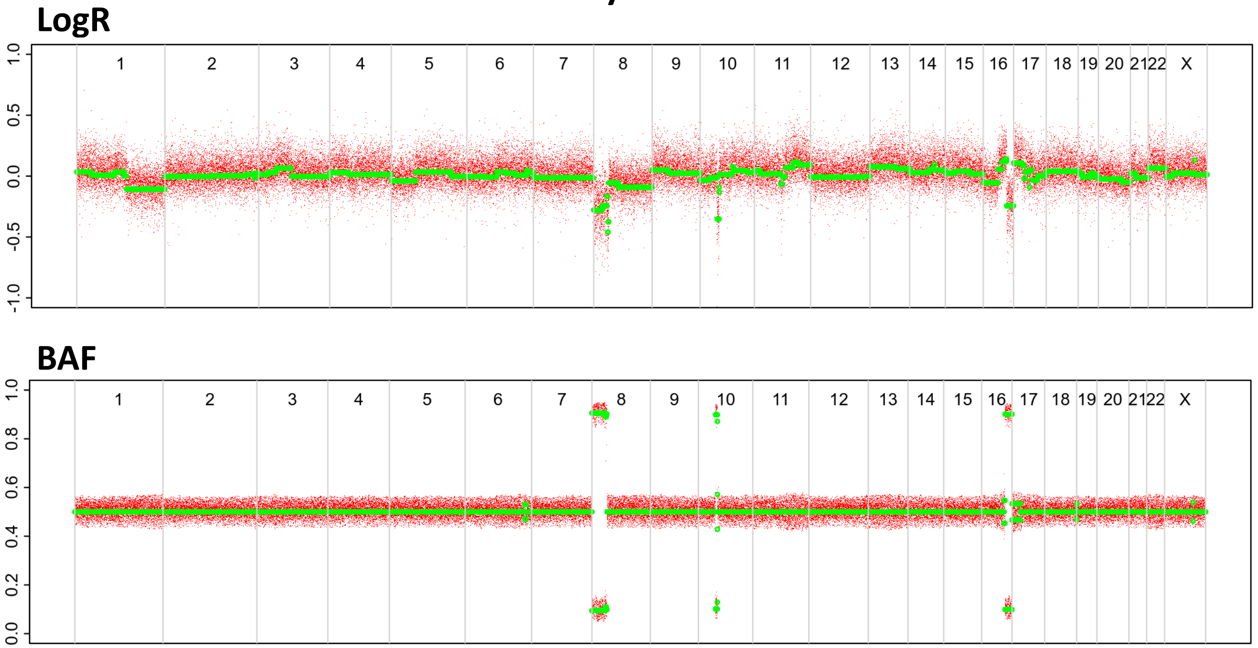

2nd event

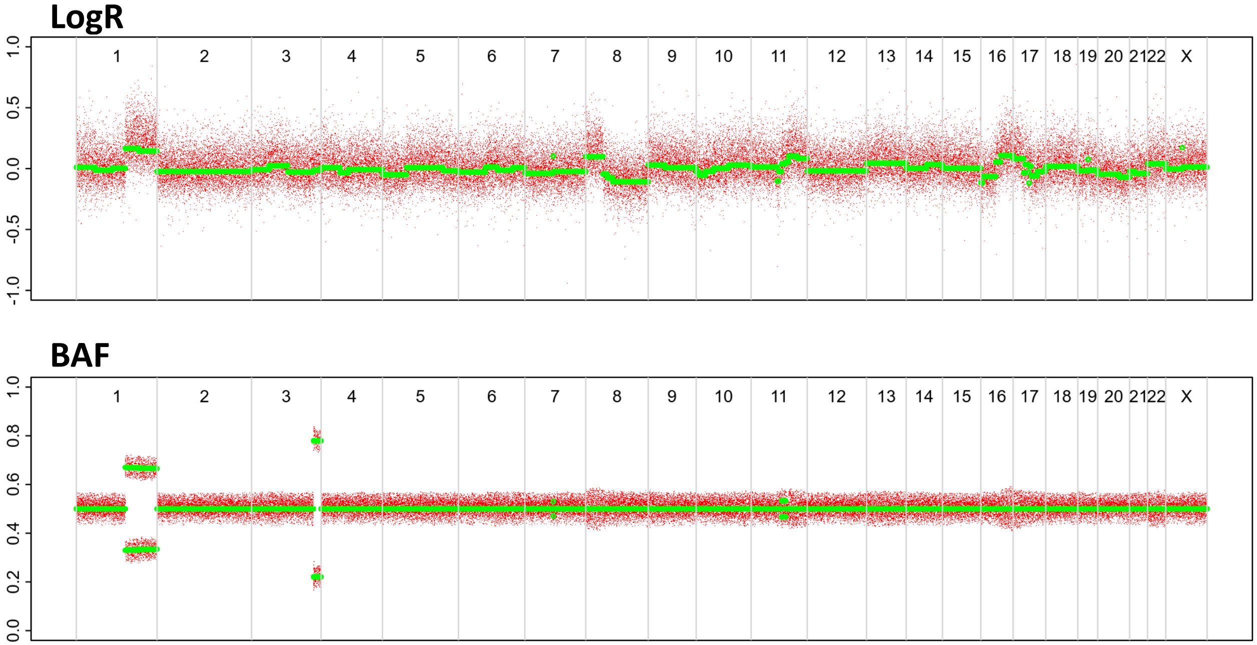

P131

Syn/Meta	Time from 1st surgery to 2nd event (Months)	Side 2nd event	Histology 2nd event	Surgery	AdjuvanT Treatment Pri (RT/ HT)	ER Pri	ER 2nd event	Her2 Pri	Her2 2nd event	Grade Pri	Grade 2nd event	Quadrant 2nd event	Margins	Screening	Clonality P value Copy N	Clonality P value Panel seq	Clonality P value WES	Final verdict
metachronous	15	Contralateral	IDC no DCIS	lumpectomy	RT	-	-	+	+	3	3	NA	<2mm	screen-detected	0.056410256	1	NA	Unrelated

Primary event

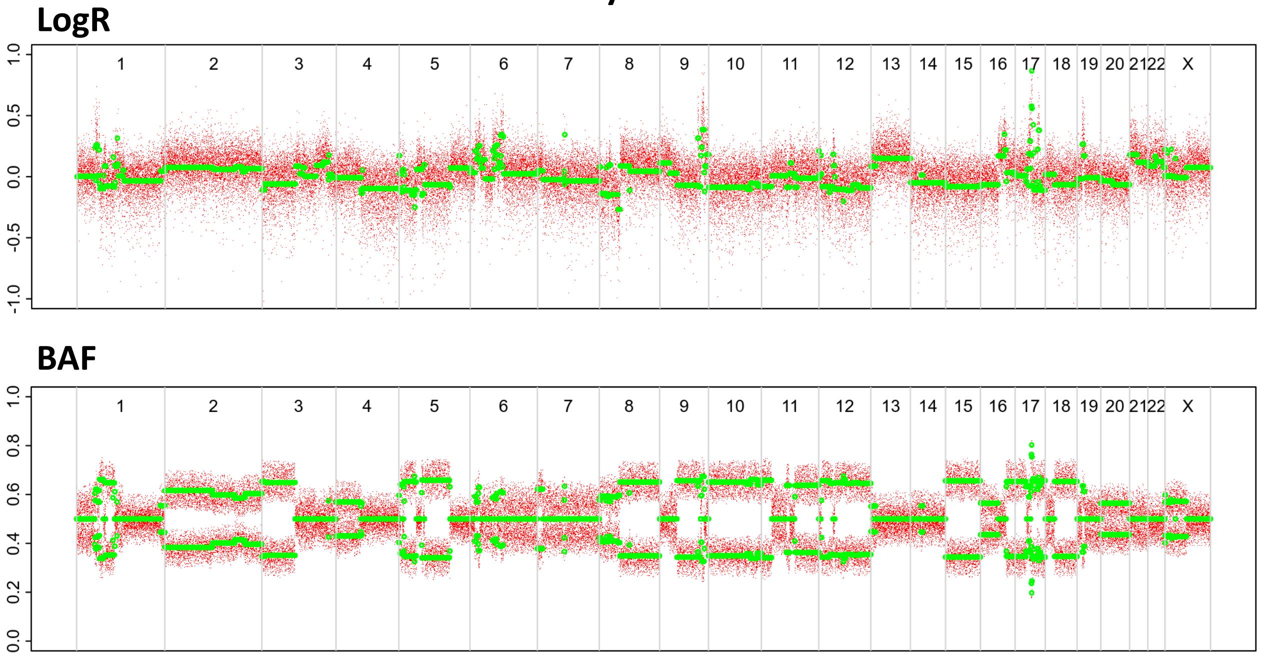

2nd event

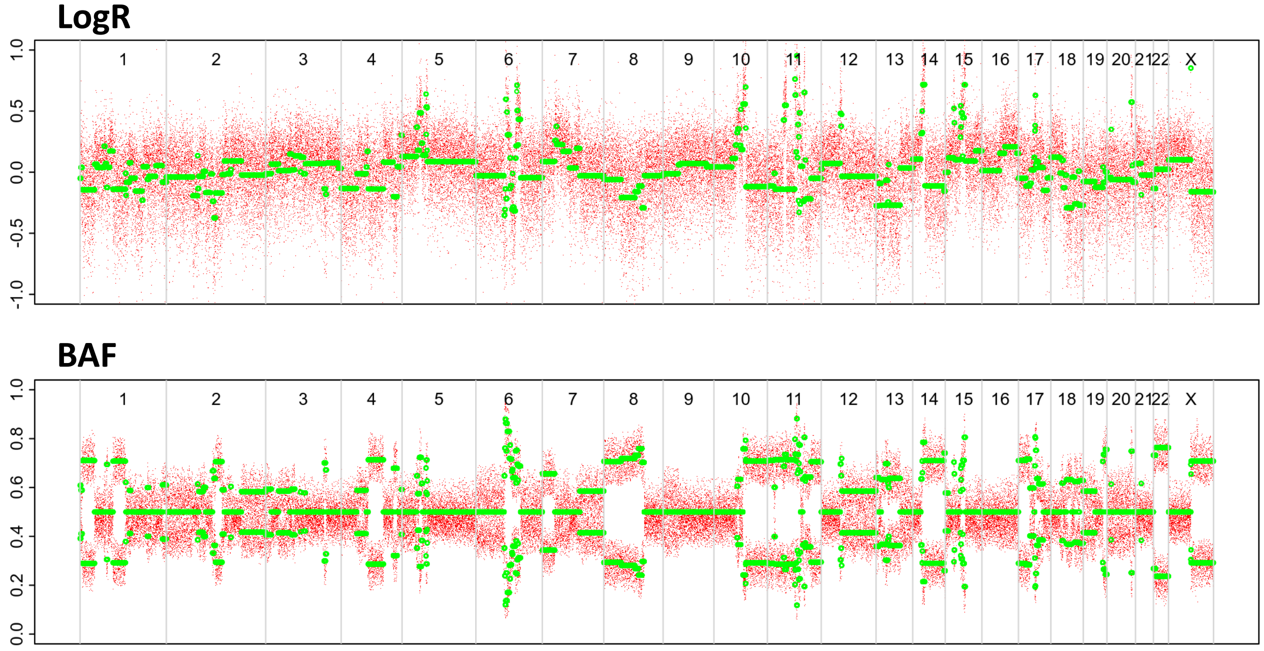

P132

Syn/Meta	Time from 1st surgery to 2nd event (Months)	Side 2nd event	Histology 2nd event	Surgery	AdjuvanT Treatment Pri (RT/ HT)	ER Pri	ER 2nd event	Her2 Pri	Her2 2nd event	Grade Pri	Grade 2nd event	Quadrant 2nd event	Margins	Screening	Clonality P value Copy N	Clonality P value Panel seq	Clonality P value WES	Final verdict
metachronous	29	Contralateral	IDC no DCIS	lumpectomy	RTHT	+	NA	NA	NA	2	2	NA	Clear	screen-detected	0.115151515	1	NA	Unrelated

Primary event

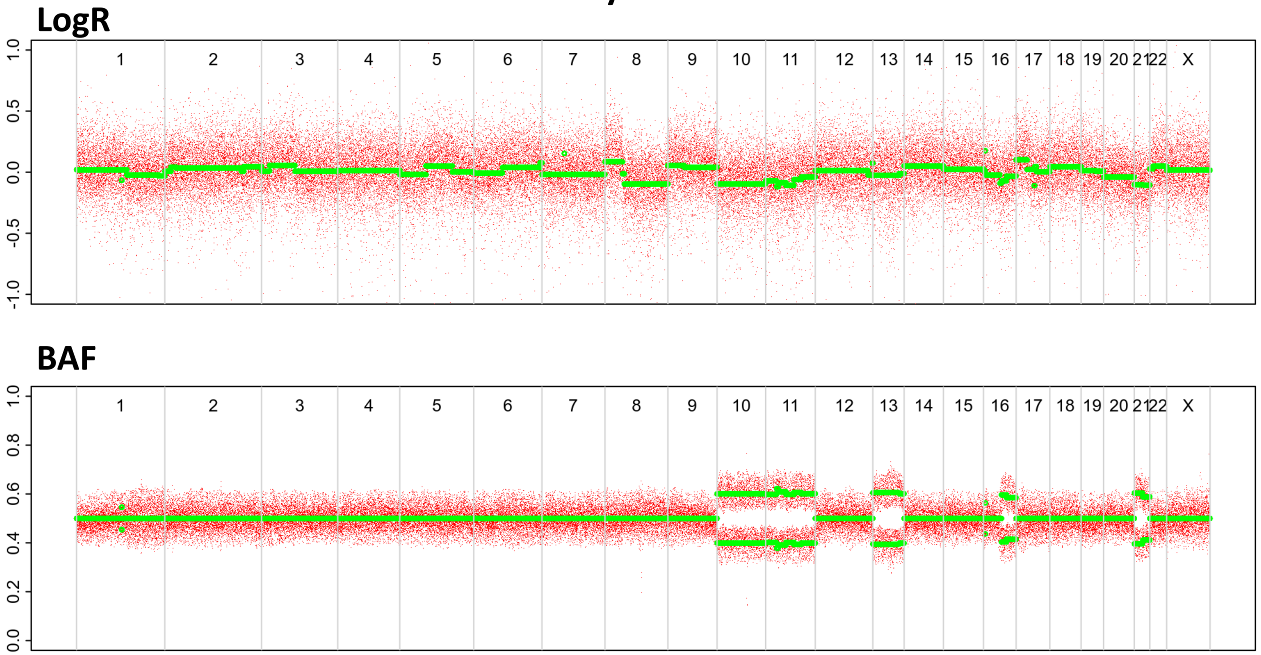

2nd event

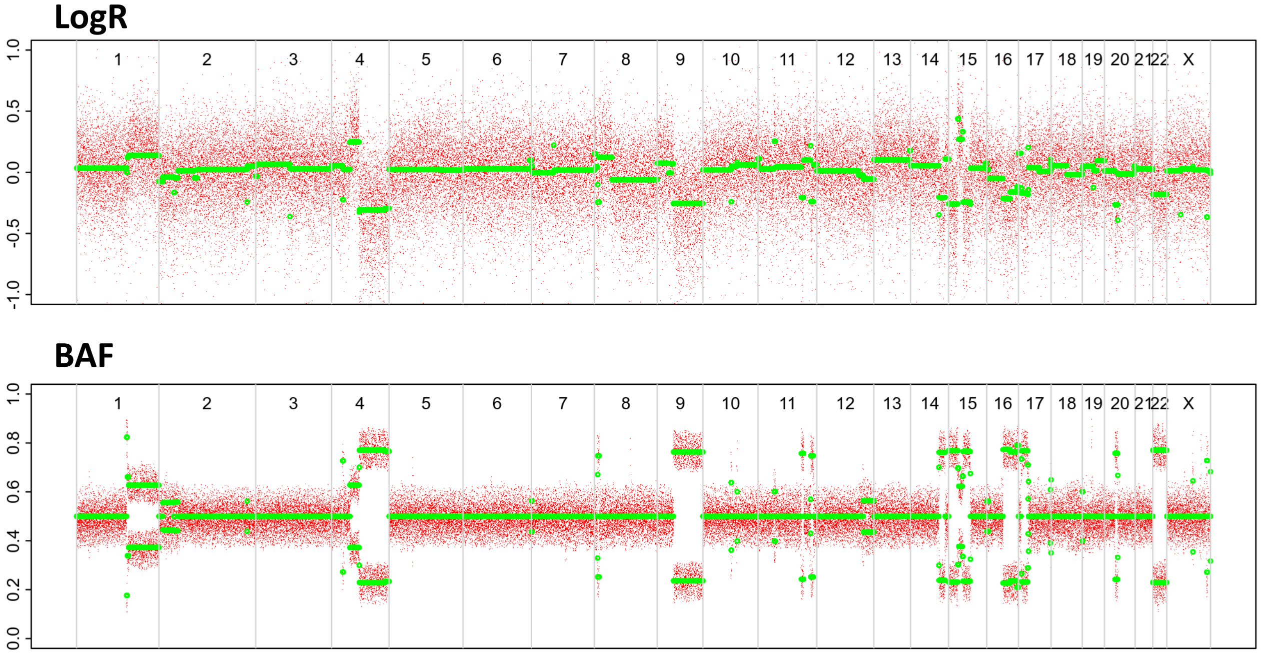

P133

Syn/Meta	Time from 1st surgery to 2nd event (Months)	Side 2nd event	Histology 2nd event	Surgery	AdjuvanT Treatment Pri (RT/ HT)	ER Pri	ER 2nd event	Her2 Pri	Her2 2nd event	Grade Pri	Grade 2nd event	Quadrant 2nd event	Margins	Screening	Clonality P value Copy N	Clonality P value Panel seq	Clonality P value WES	Final verdict
metachronous	63	Contralateral	IDC no DCIS	lumpectomy	HT	+	+	NA	NA	3	2	NA	Clear	screen-detected	0.134731935	NA	NA	Unrelated

Primary event

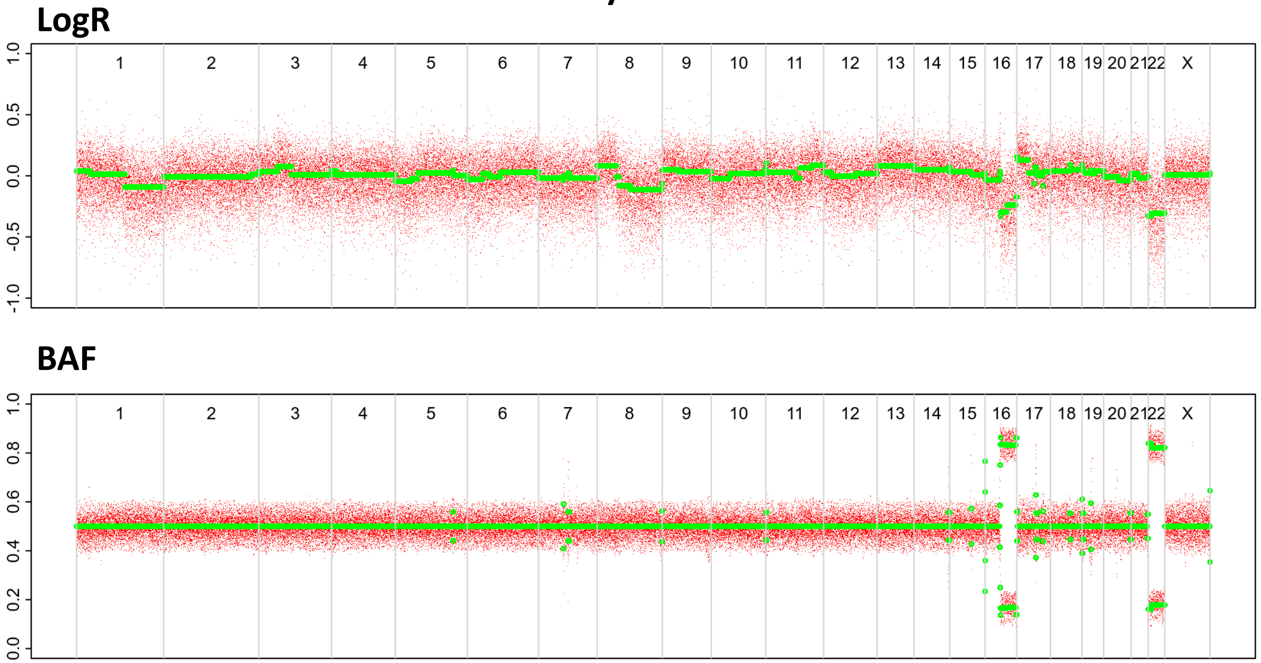

2nd event

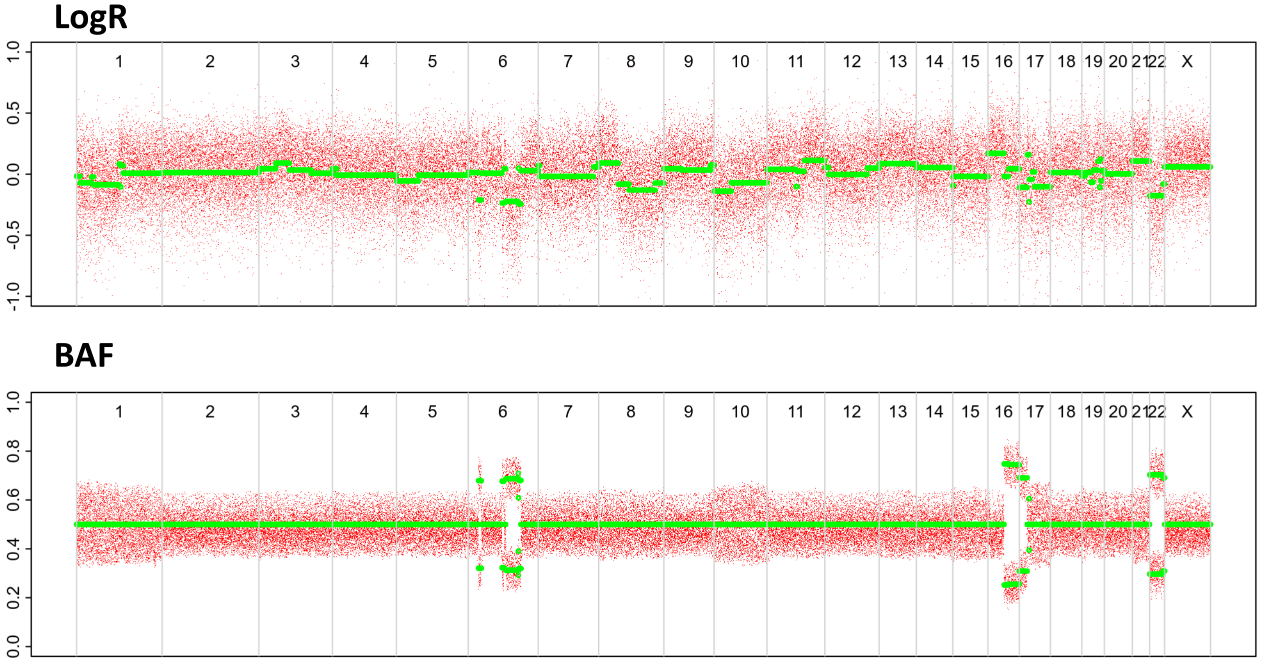

P134

Syn/Meta	Time from 1st surgery to 2nd event (Months)	Side 2nd event	Histology 2nd event	Surgery	AdjuvanT Treatment Pri (RT/ HT)	ER Pri	ER 2nd event	Her2 Pri	Her2 2nd event	Grade Pri	Grade 2nd event	Quadrant 2nd event	Margins	Screening	Clonality P value Copy N	Clonality P value Panel seq	Clonality P value WES	Final verdict
metachronous	61	Contralateral	IDC no DCIS	lumpectomy	RT	-	+	NA	NA	3	2	NA	Clear	screen-detected	0.216317016	NA	NA	Unrelated

Primary event

LogR

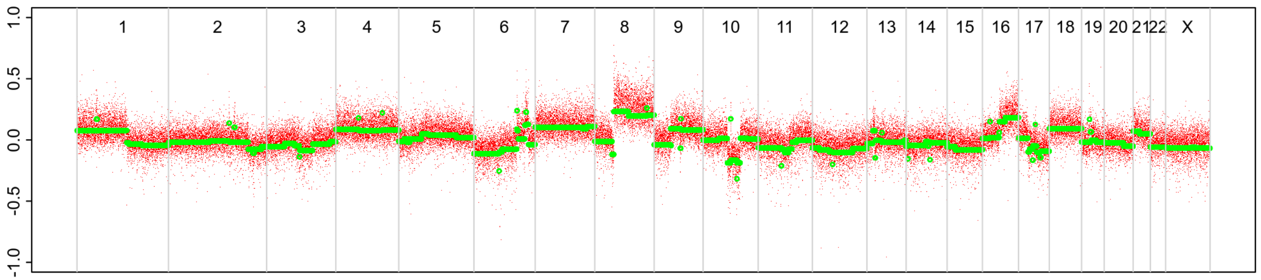

BAF

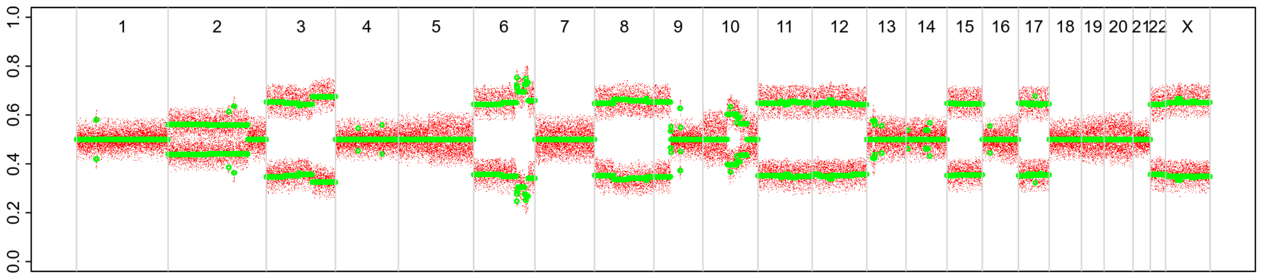

2nd event

LogR

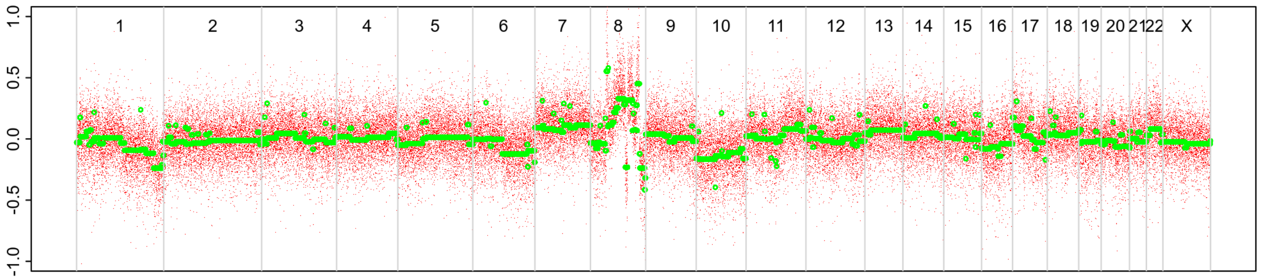

BAF

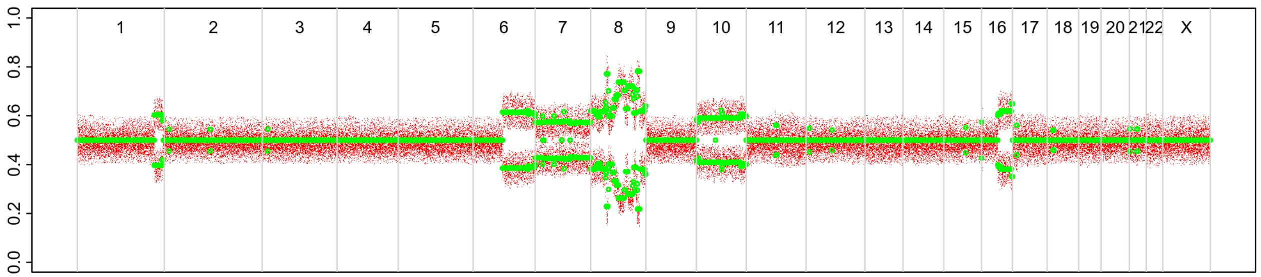

P135

Syn/Meta	Time from 1st surgery to 2nd event (Months)	Side 2nd event	Histology 2nd event	Surgery	AdjuvanT Treatment Pri (RT/ HT)	ER Pri	ER 2nd event	Her2 Pri	Her2 2nd event	Grade Pri	Grade 2nd event	Quadrant 2nd event	Margins	Screening	Clonality P value Copy N	Clonality P value Panel seq	Clonality P value WES	Final verdict
metachronous	34	Contralateral	IDC no DCIS	lumpectomy	RTHT	+	NA	-	NA	3	2	NA	Clear	screen-detected	0.244755245	NA	NA	Unrelated

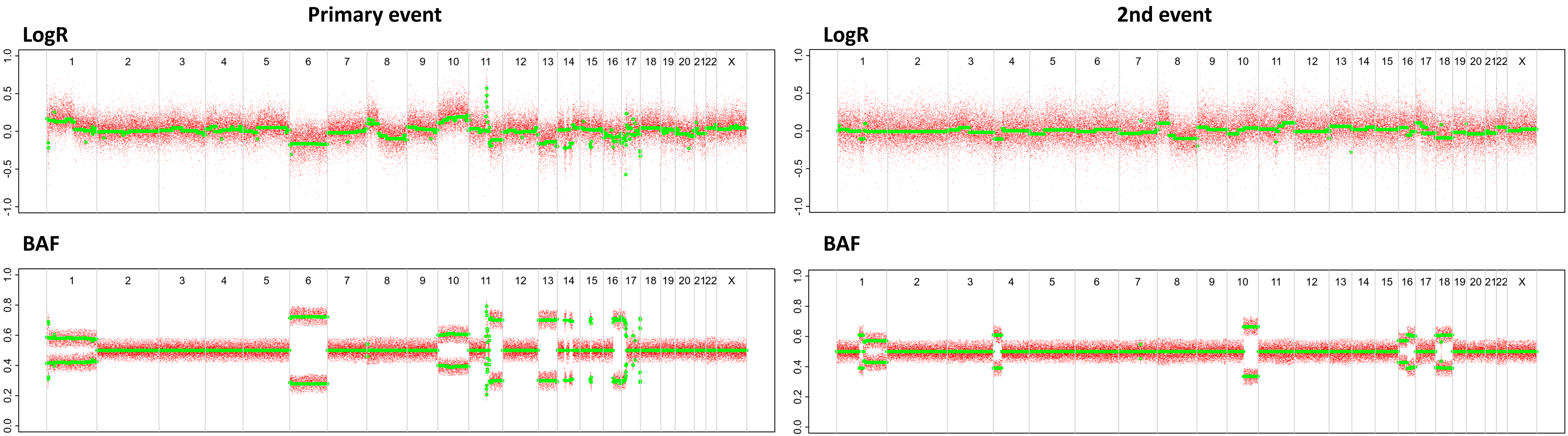

P136

Syn/Meta	Time from 1st surgery to 2nd event (Months)	Side 2nd event	Histology 2nd event	Surgery	AdjuvanT Treatment Pri (RT/ HT)	ER Pri	ER 2nd event	Her2 Pri	Her2 2nd event	Grade Pri	Grade 2nd event	Quadrant 2nd event	Margins	Screening	Clonality P value Copy N	Clonality P value Panel seq	Clonality P value WES	Final verdict
metachronous	20	Contralateral	IDC no DCIS	lumpectomy	RT	-	+	+	NA	3	2	NA	Clear	screen-detected	0.305361305	0.16	NA	Unrelated

Primary event

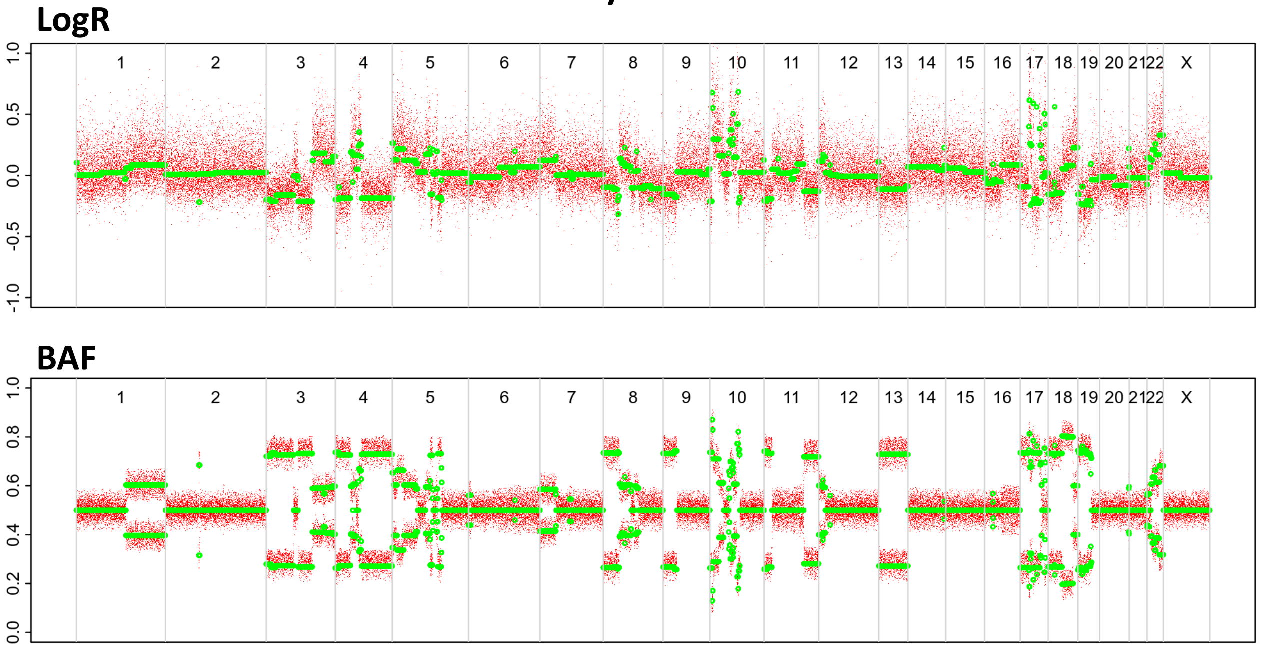

2nd event

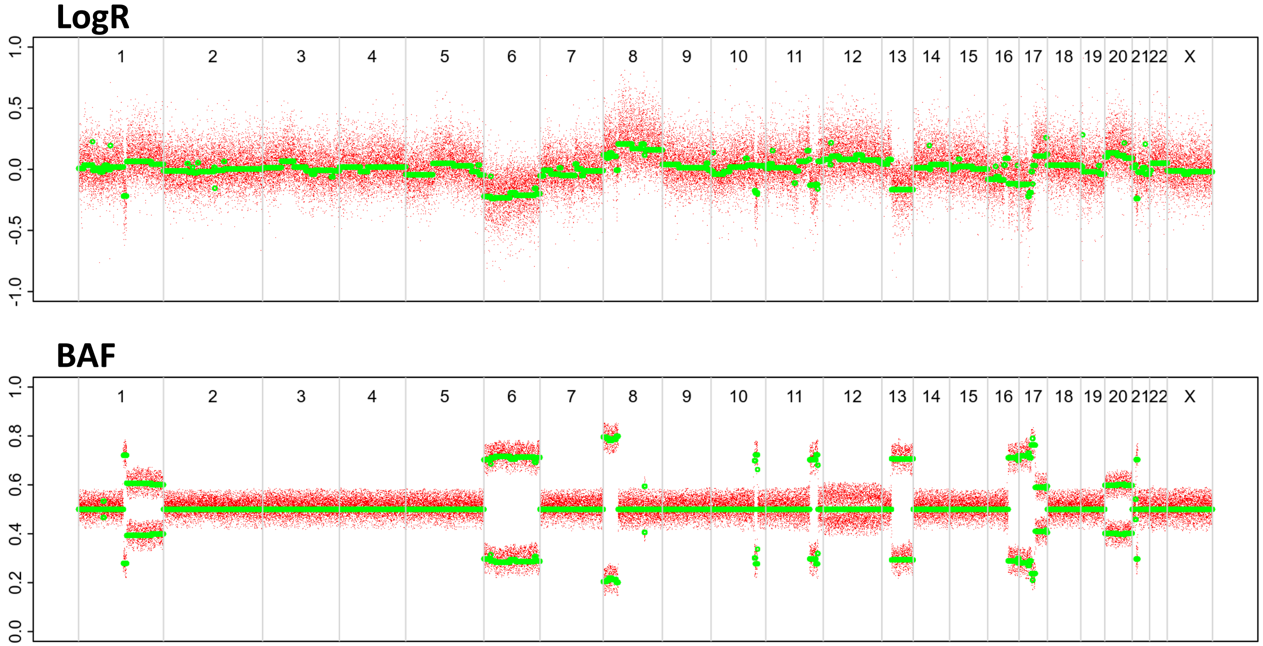

P137

Syn/Meta	Time from 1st surgery to 2nd event (Months)	Side 2nd event	Histology 2nd event	Surgery	AdjuvanT Treatment Pri (RT/ HT)	ER Pri	ER 2nd event	Her2 Pri	Her2 2nd event	Grade Pri	Grade 2nd event	Quadrant 2nd event	Margins	Screening	Clonality P value Copy N	Clonality P value Panel seq	Clonality P value WES	Final verdict
metachronous	111	Contralateral	IDC no DCIS	lumpectomy	None	+	NA	NA	NA	3	3	NA	Clear	screen-detected	0.373892774	1	NA	Unrelated

Primary event

LogR

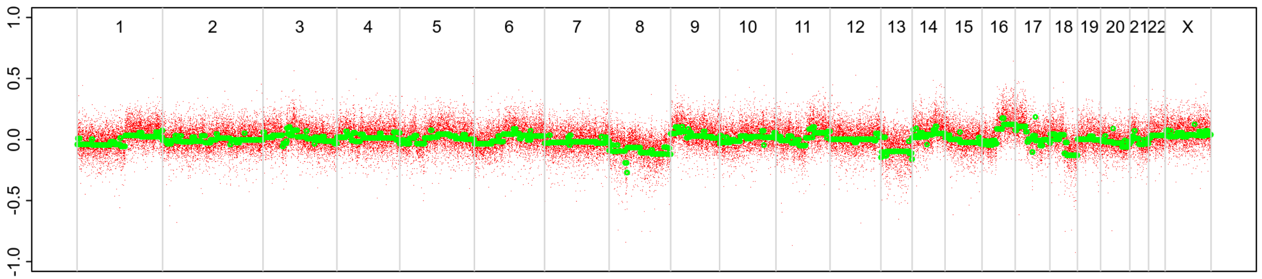

BAF

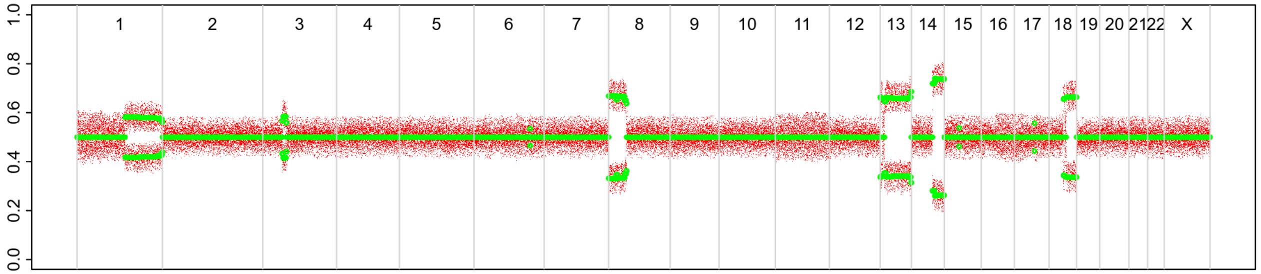

2nd event

LogR

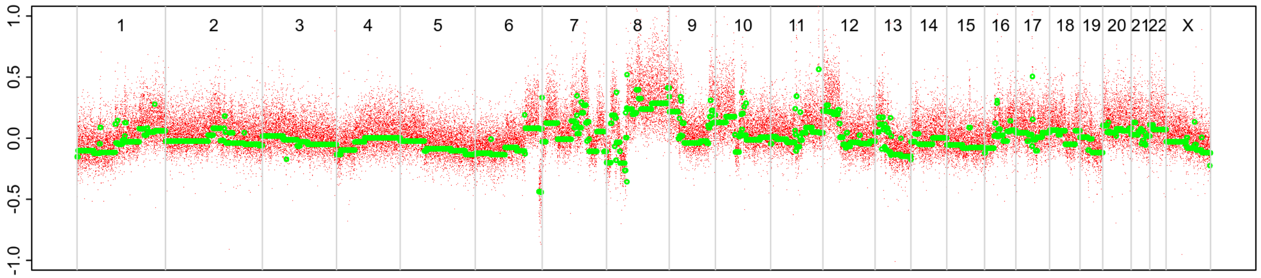

BAF

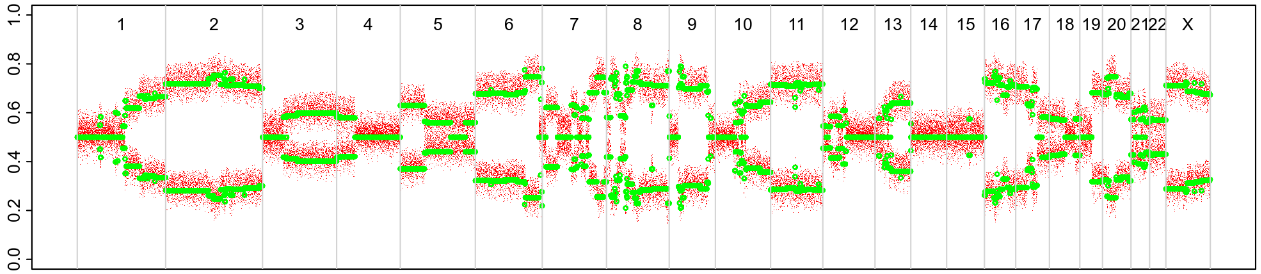

P138

Syn/Meta	Time from 1st surgery to 2nd event (Months)	Side 2nd event	Histology 2nd event	Surgery	AdjuvanT Treatment Pri (RT/ HT)	ER Pri	ER 2nd event	Her2 Pri	Her2 2nd event	Grade Pri	Grade 2nd event	Quadrant 2nd event	Margins	Screening	Clonality P value Copy N	Clonality P value Panel seq	Clonality P value WES	Final verdict
metachronous	27	Contralateral	IDC with DCIS	lumpectomy	None	+	NA	-	-	2	3	NA	Clear	screen-detected	0.436363636	1	NA	Unrelated

Primary event

LogR

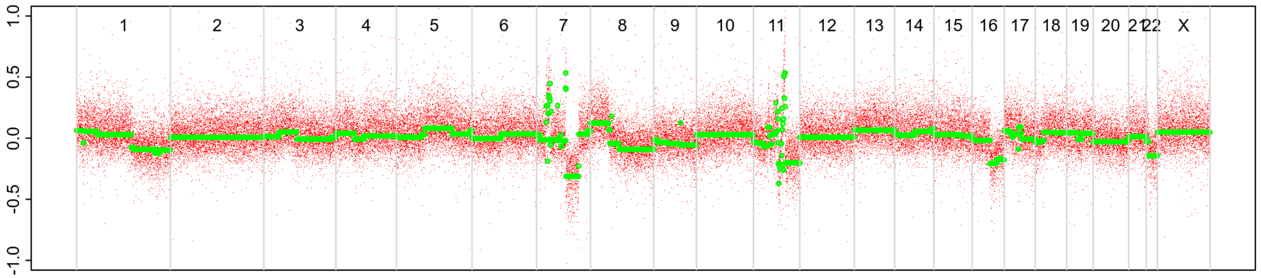

BAF

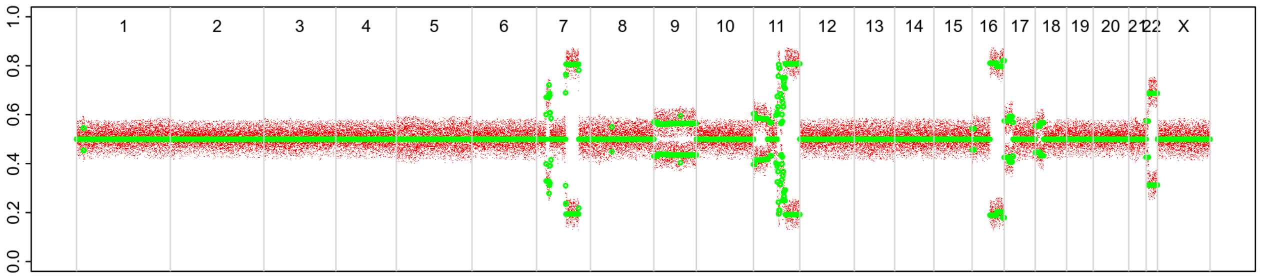

2nd event

LogR

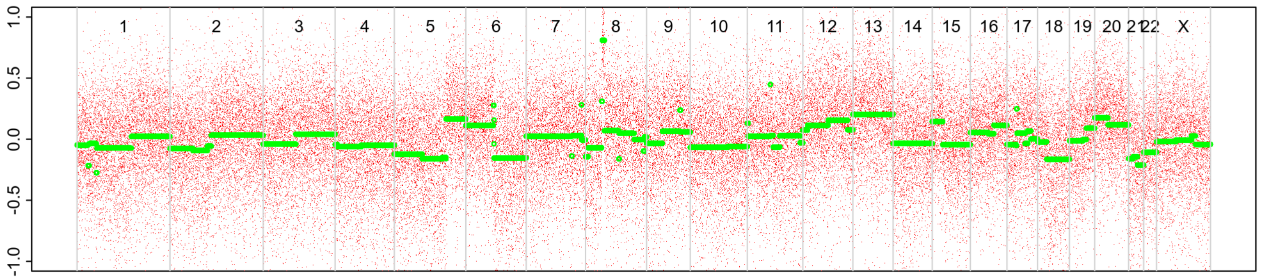

BAF

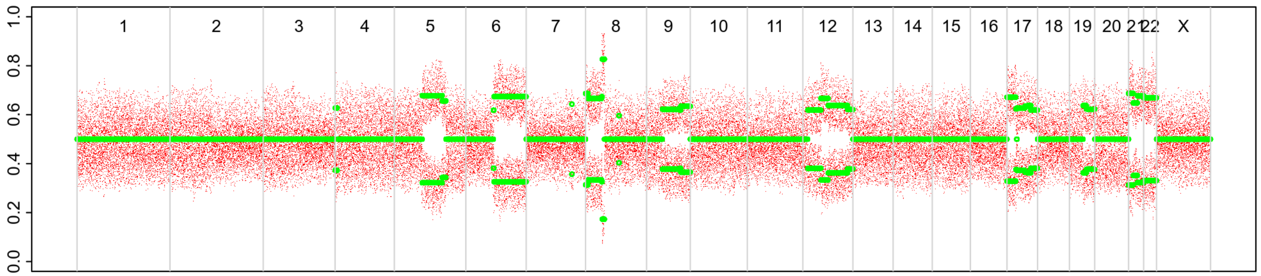

P139

Syn/Meta	Time from 1st surgery to 2nd event (Months)	Side 2nd event	Histology 2nd event	Surgery	AdjuvanT Treatment Pri (RT/ HT)	ER Pri	ER 2nd event	Her2 Pri	Her2 2nd event	Grade Pri	Grade 2nd event	Quadrant 2nd event	Margins	Screening	Clonality P value Copy N	Clonality P value Panel seq	Clonality P value WES	Final verdict
metachronous	56	Contralateral	IDC with DCIS	lumpectomy	RT	+	+	-	-	3	3	NA	Clear	screen-detected	0.468997669	0.14	NA	Unrelated

Primary event

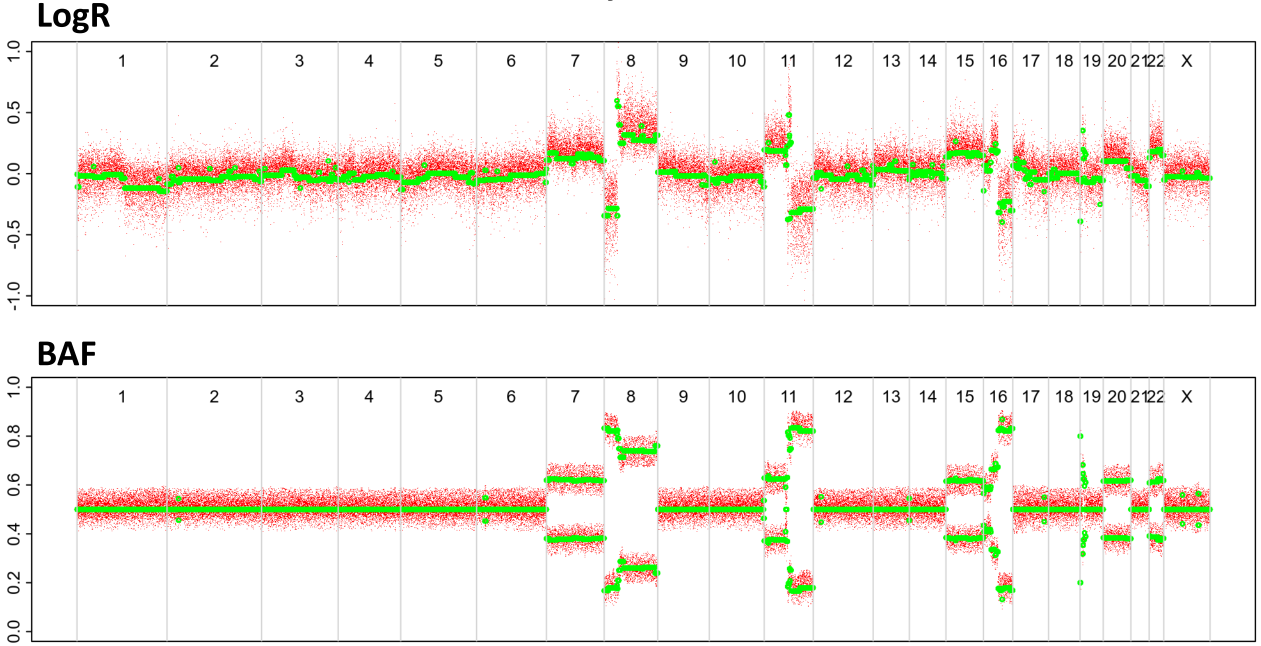

2nd event

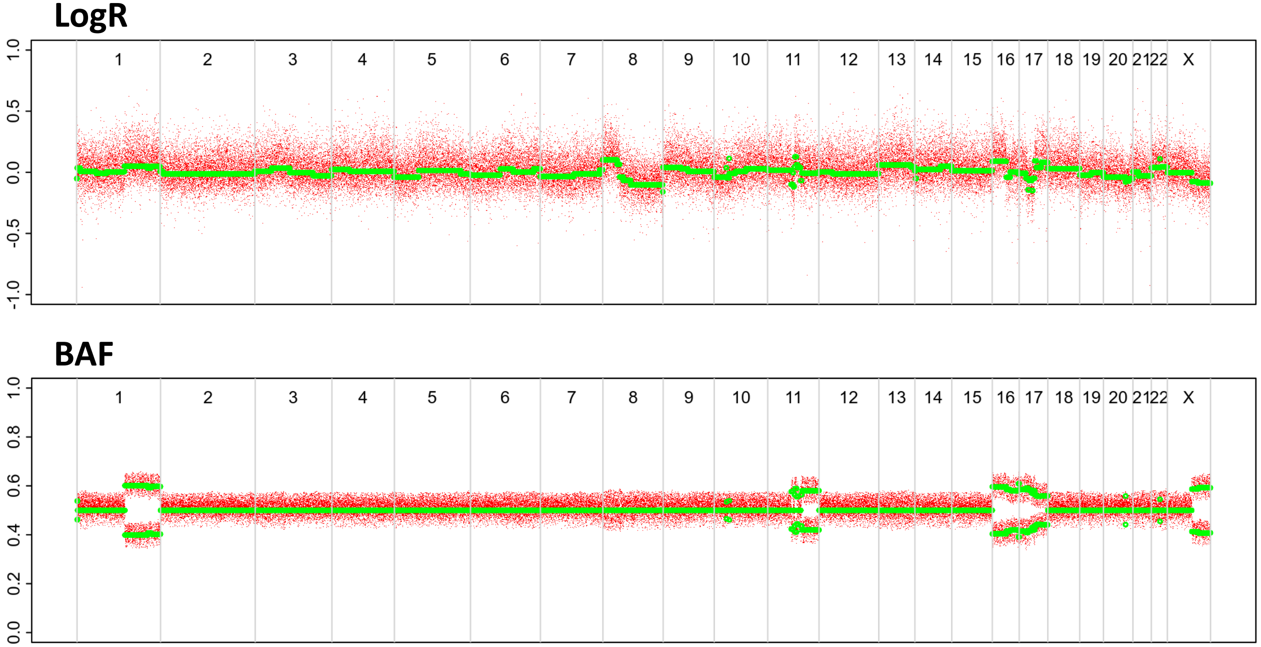

P140

Syn/Meta	Time from 1st surgery to 2nd event (Months)	Side 2nd event	Histology 2nd event	Surgery	AdjuvanT Treatment Pri (RT/ HT)	ER Pri	ER 2nd event	Her2 Pri	Her2 2nd event	Grade Pri	Grade 2nd event	Quadrant 2nd event	Margins	Screening	Clonality P value Copy N	Clonality P value Panel seq	Clonality P value WES	Final verdict
metachronous	12	Contralateral	IDC no DCIS	lumpectomy	HT	+	+	NA	NA	3	2	NA	Clear	screen-detected	0.712354312	NA	NA	Unrelated

Primary event

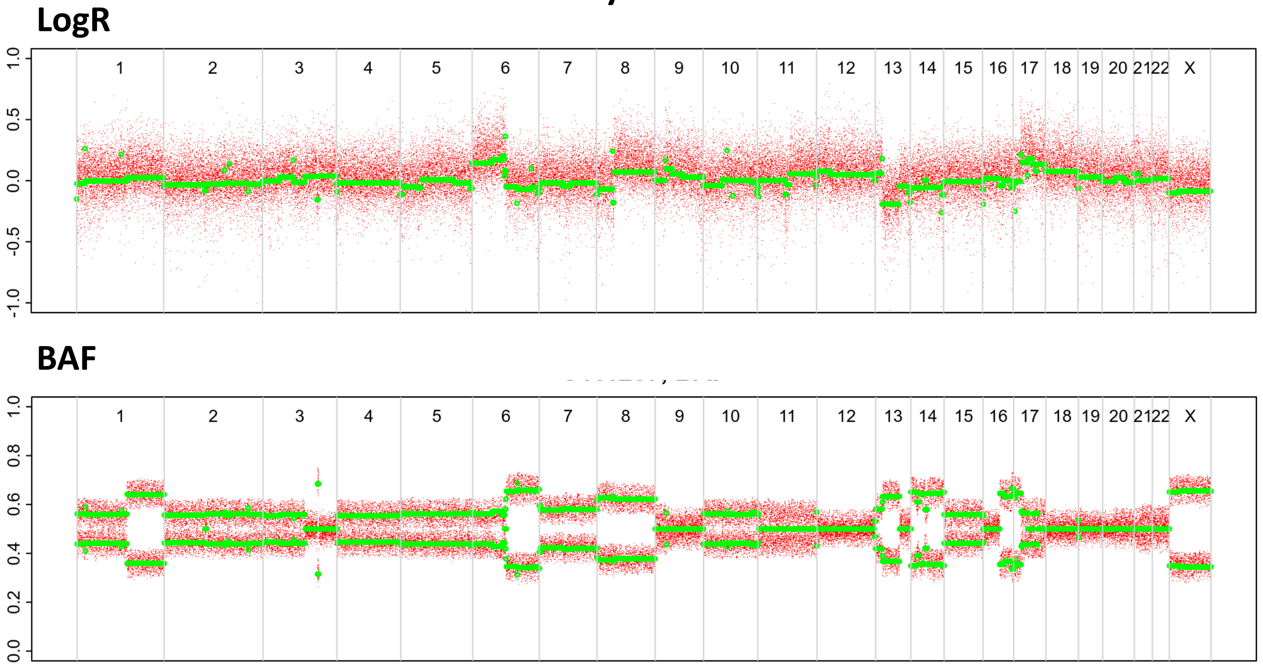

2nd event

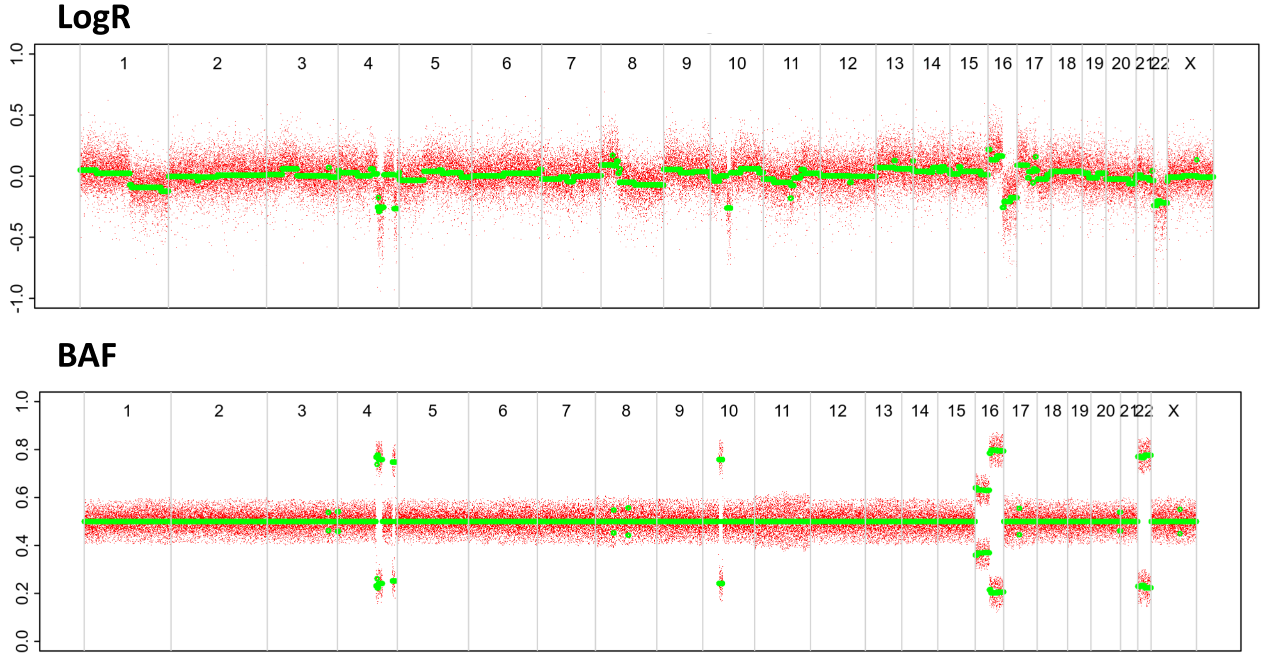

P141

Syn/Meta	Time from 1st surgery to 2nd event (Months)	Side 2nd event	Histology 2nd event	Surgery	AdjuvanT Treatment Pri (RT/ HT)	ER Pri	ER 2nd event	Her2 Pri	Her2 2nd event	Grade Pri	Grade 2nd event	Quadrant 2nd event	Margins	Screening	Clonality P value Copy N	Clonality P value Panel seq	Clonality P value WES	Final verdict
metachronous	79	Contralateral	IDC with DCIS	lumpectomy	RT	-	NA	NA	NA	3	3	NA	Clear	screen-detected	0.722610723	NA	NA	Unrelated

Primary event

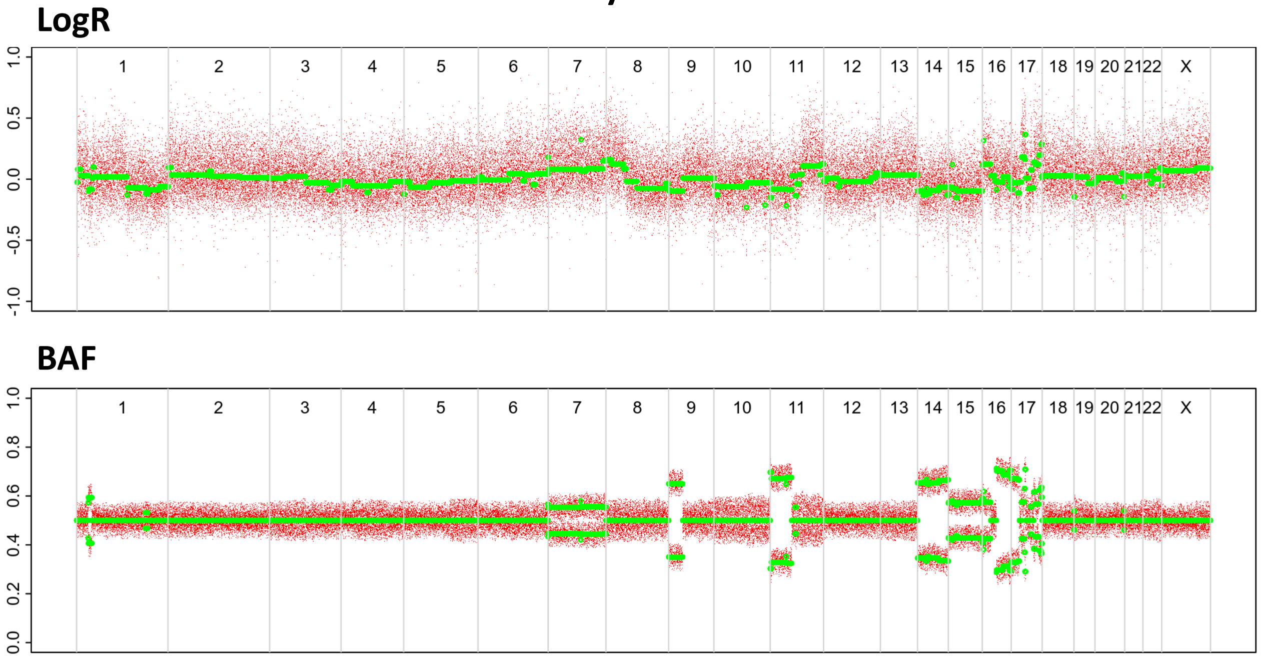

2nd event

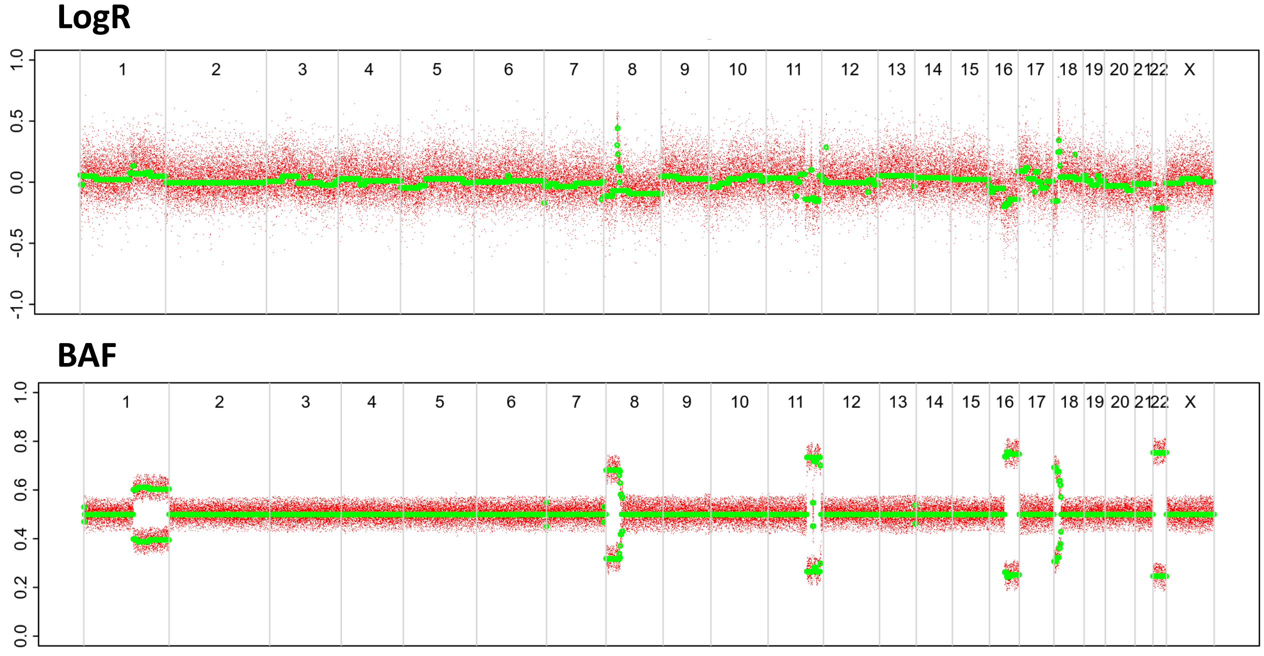

P142

Syn/Meta	Time from 1st surgery to 2nd event (Months)	Side 2nd event	Histology 2nd event	Surgery	AdjuvanT Treatment Pri (RT/ HT)	ER Pri	ER 2nd event	Her2 Pri	Her2 2nd event	Grade Pri	Grade 2nd event	Quadrant 2nd event	Margins	Screening	Clonality P value Copy N	Clonality P value Panel seq	Clonality P value WES	Final verdict
metachronous	26	Contralateral	IDC no DCIS	lumpectomy	None	-	+	-	NA	2	1	NA	Clear	screen-detected	1	0.16	NA	Unrelated

Primary event

LogR

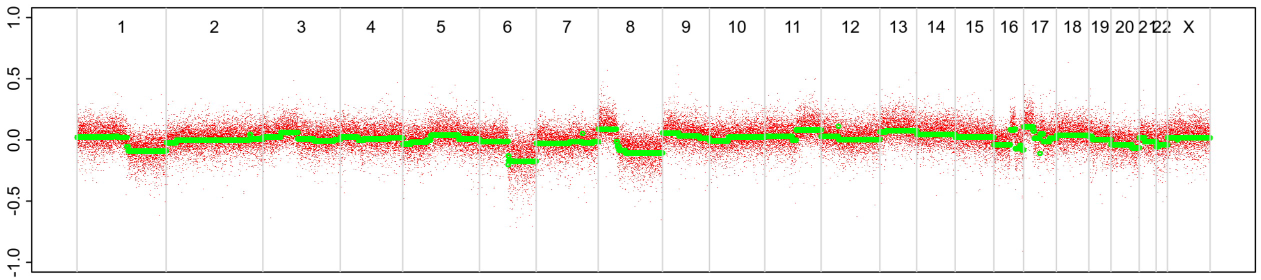

BAF

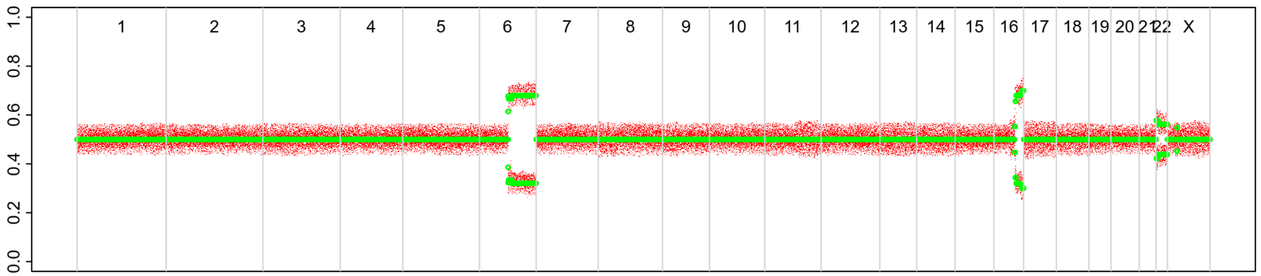

2nd event

LogR

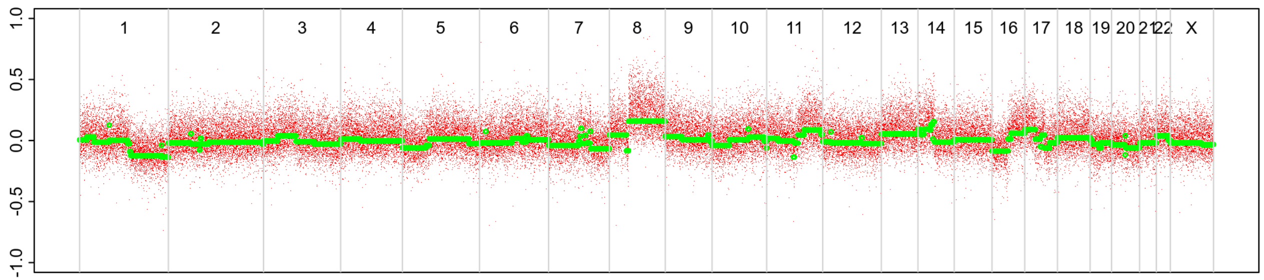

BAF

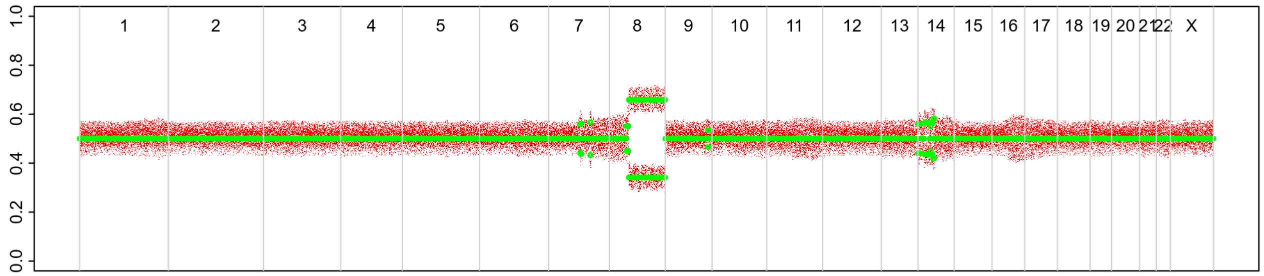

P031

Syn/Meta	Time from 1st surgery to 2nd event (Months)	Side 2nd event	Histology 2nd event	Surgery	AdjuvanT Treatment Pri (RT/ HT)	ER Pri	ER 2nd event	Her2 Pri	Her2 2nd event	Grade Pri	Grade 2nd event	Quadrant 2nd event	Margins	Screening	Clonality P value Copy N	Clonality P value Panel seq	Clonality P value WES	Final verdict
metachronous	167	Ipsilateral	Invasive	lumpectomy	None	+	+	-	-	2	2	NA	involved	screen-detected	0.000649 14	NA	NA	Related

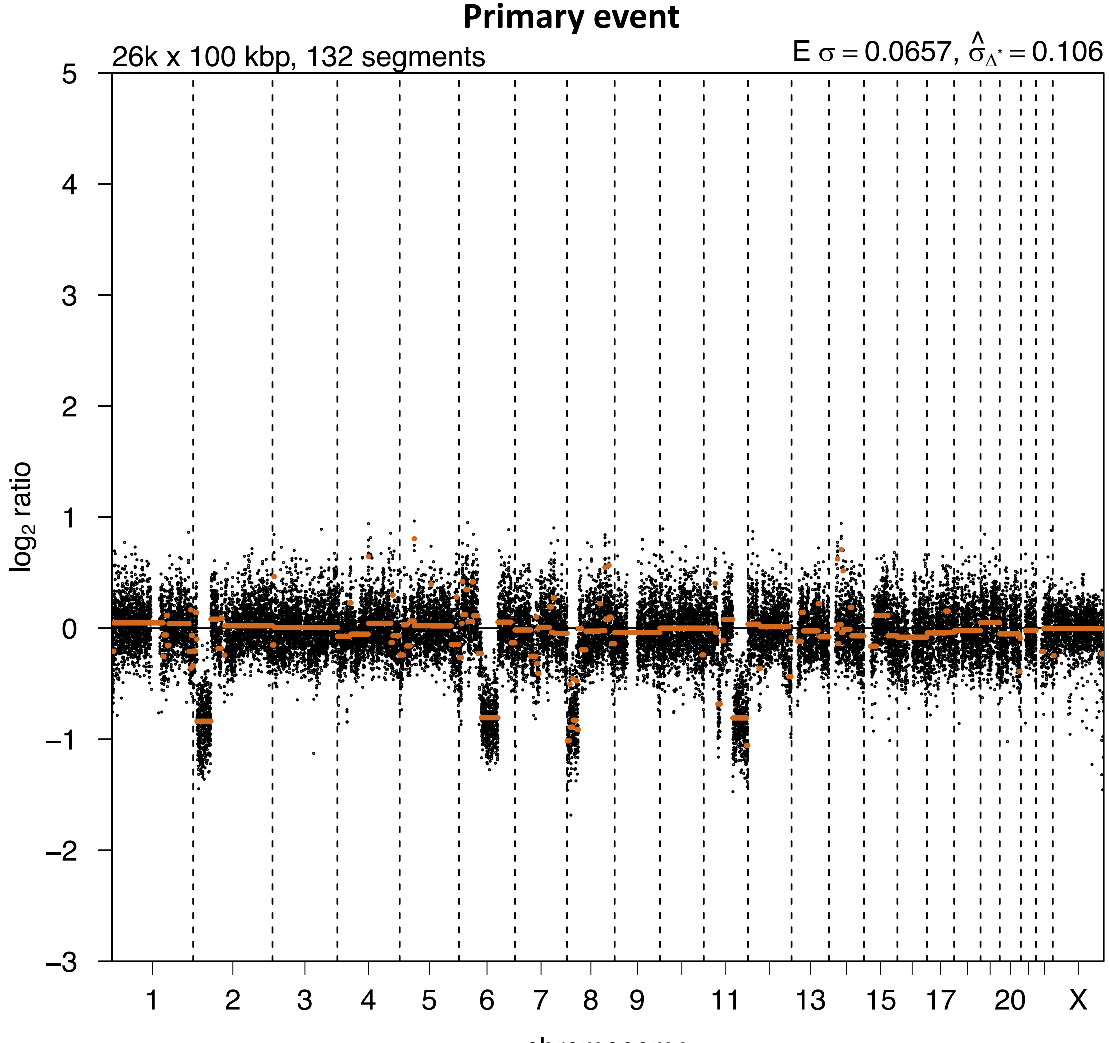

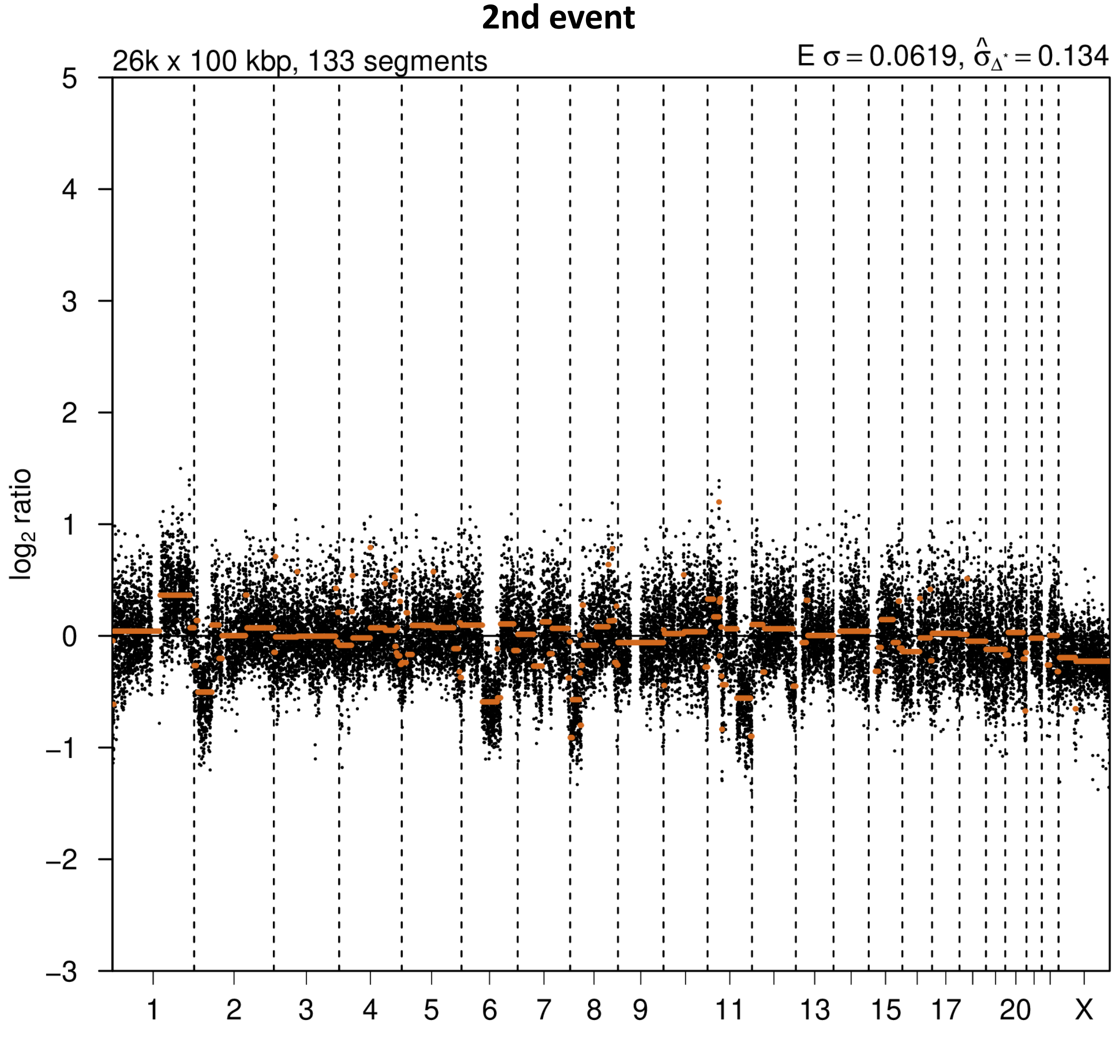

P032

Syn/Meta	Time from 1st surgery to 2nd event (Months)	Side 2nd event	Histology 2nd event	Surgery	AdjuvanT Treatment Pri (RT/ HT)	ER Pri	ER 2nd event	Her2 Pri	Her2 2nd event	Grade Pri	Grade 2nd event	Quadrant 2nd event	Margins	Screening	Clonality P value Copy N	Clonality P value Panel seq	Clonality P value WES	Final verdict
metachronous	130	Ipsilateral	IDC with DCIS	lumpectomy	None	+	+	-	-	2	3	NA	NA	screen-detected	0.000649 14	0.006	NA	Related

Primary event

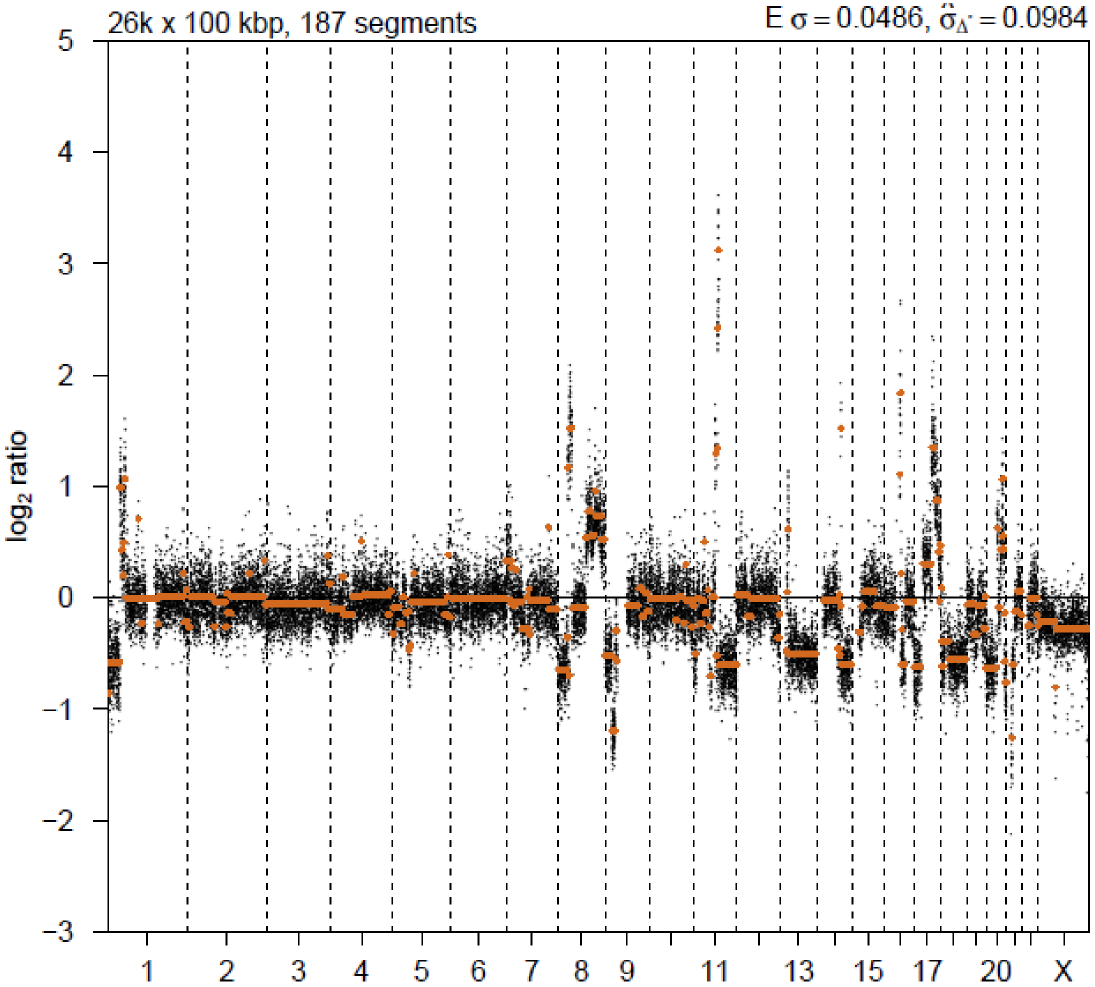

2nd event

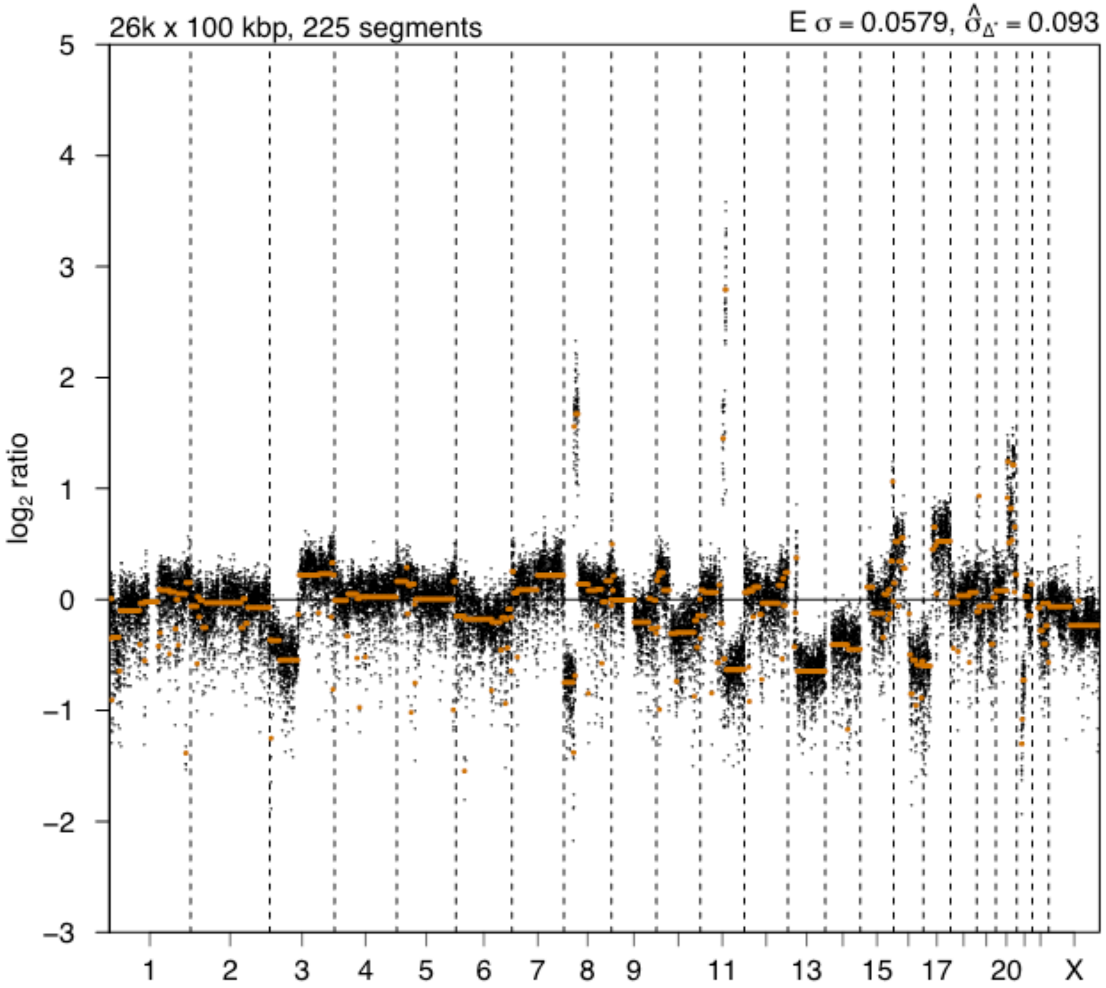

P033

Syn/Meta	Time from 1st surgery to 2nd event (Months)	Side 2nd event	Histology 2nd event	Surgery	AdjuvanT Treatment Pri (RT/ HT)	ER Pri	ER 2nd event	Her2 Pri	Her2 2nd event	Grade Pri	Grade 2nd event	Quadrant 2nd event	Margins	Screening	Clonality P value Copy N	Clonality P value Panel seq	Clonality P value WES	Final verdict
metachronous	125	Ipsilateral	IDC with DCIS	lumpectomy	None	+	+	-	-	2	2	NA	NA	screen-detected	0.003894839	0.003	NA	Related

Primary event

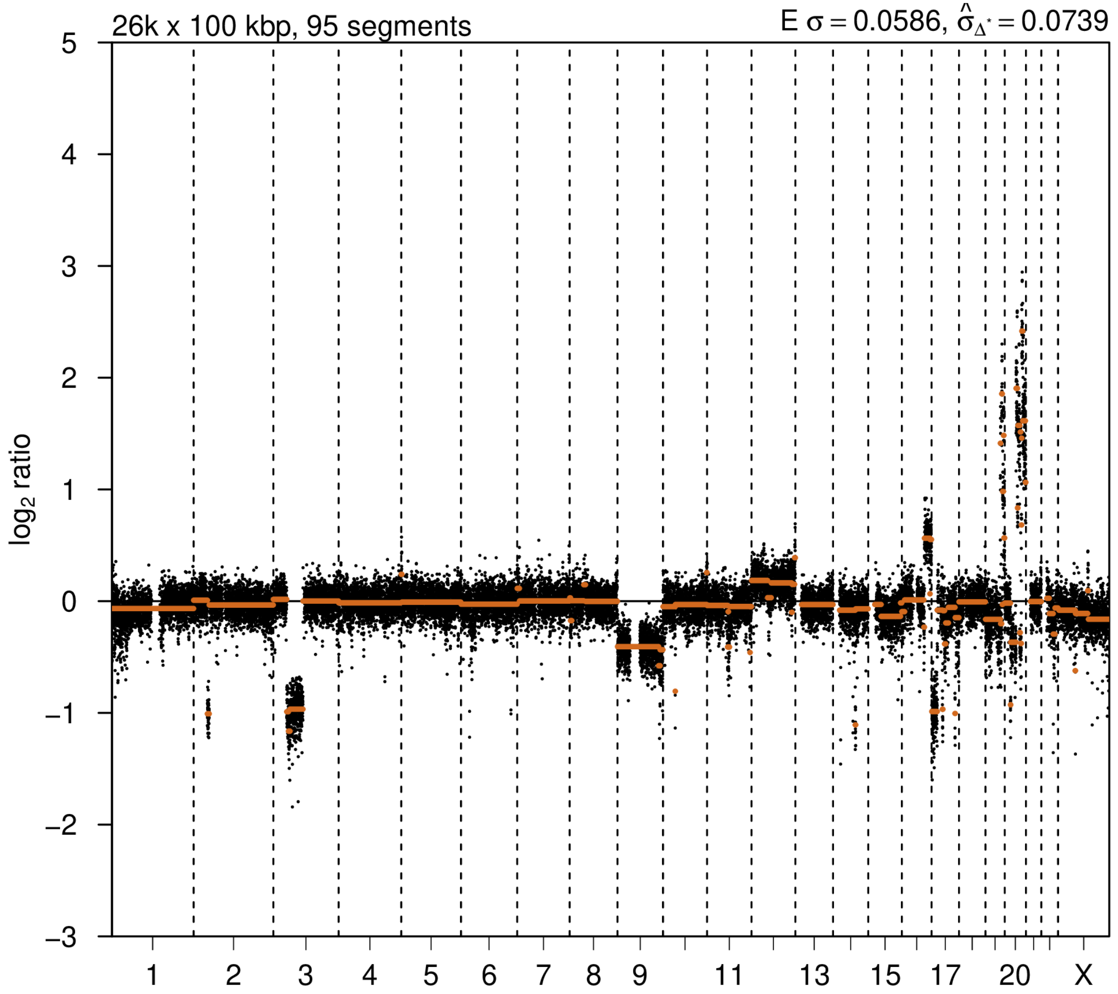

2nd event

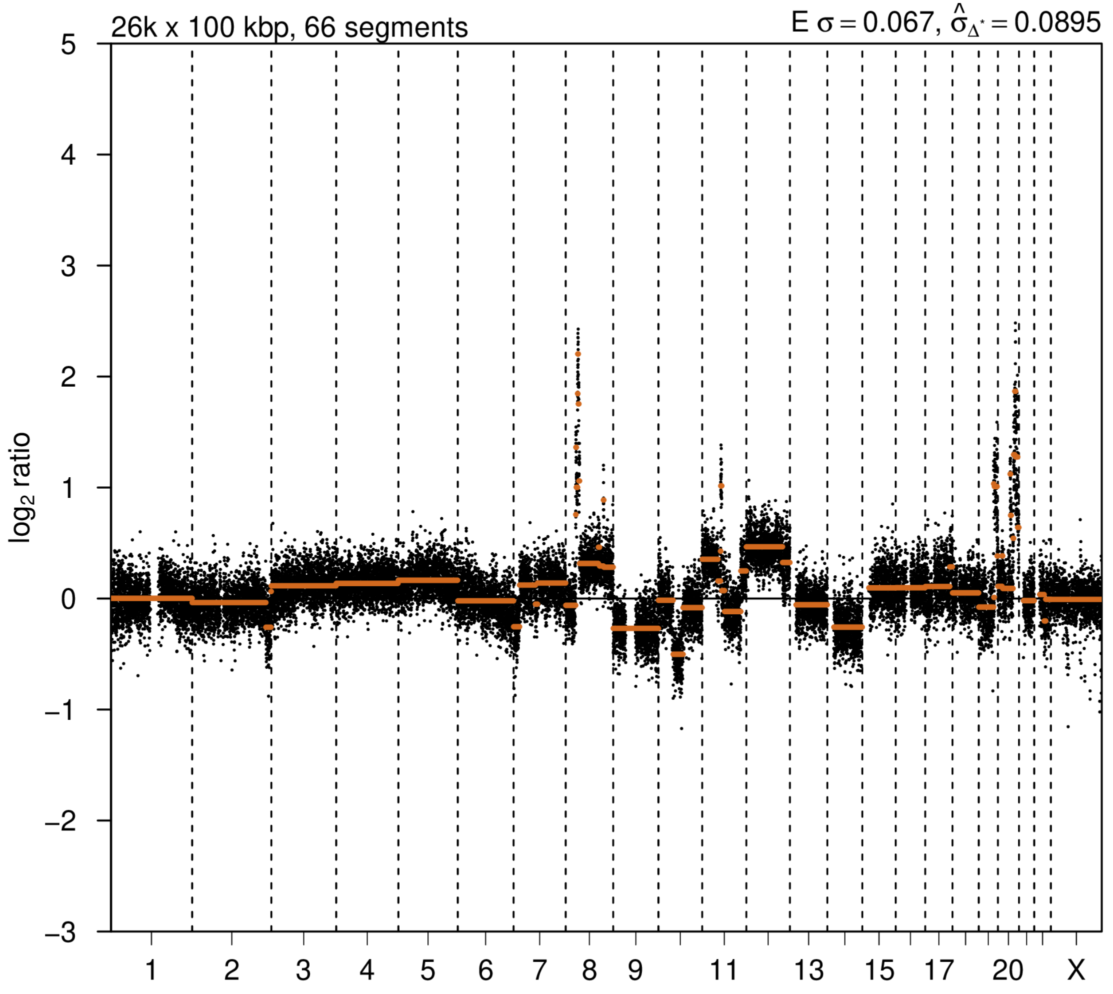

P035

Syn/Meta	Time from 1st surgery to 2nd event (Months)	Side 2nd event	Histology 2nd event	Surgery	AdjuvanT Treatment Pri (RT/ HT)	ER Pri	ER 2nd event	Her2 Pri	Her2 2nd event	Grade Pri	Grade 2nd event	Quadrant 2nd event	Margins	Screening	Clonality P value Copy N	Clonality P value Panel seq	Clonality P value WES	Final verdict
metachronous	188	Ipsilateral	Invasive	lumpectomy	None	NA	-	NA	+	3	3	NA	NA	symptomatic	0.027588445	NA	0.000752445	Related

Primary event

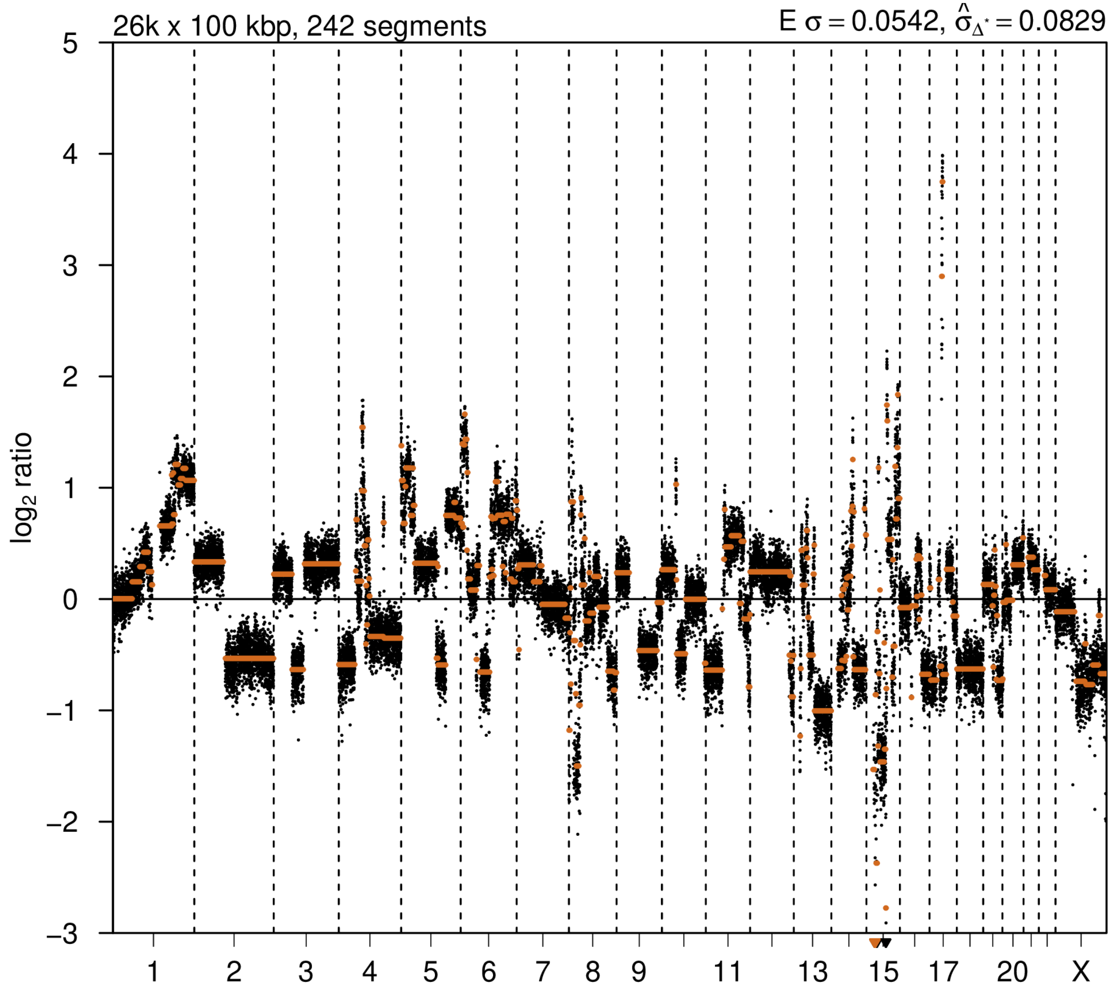

2nd event

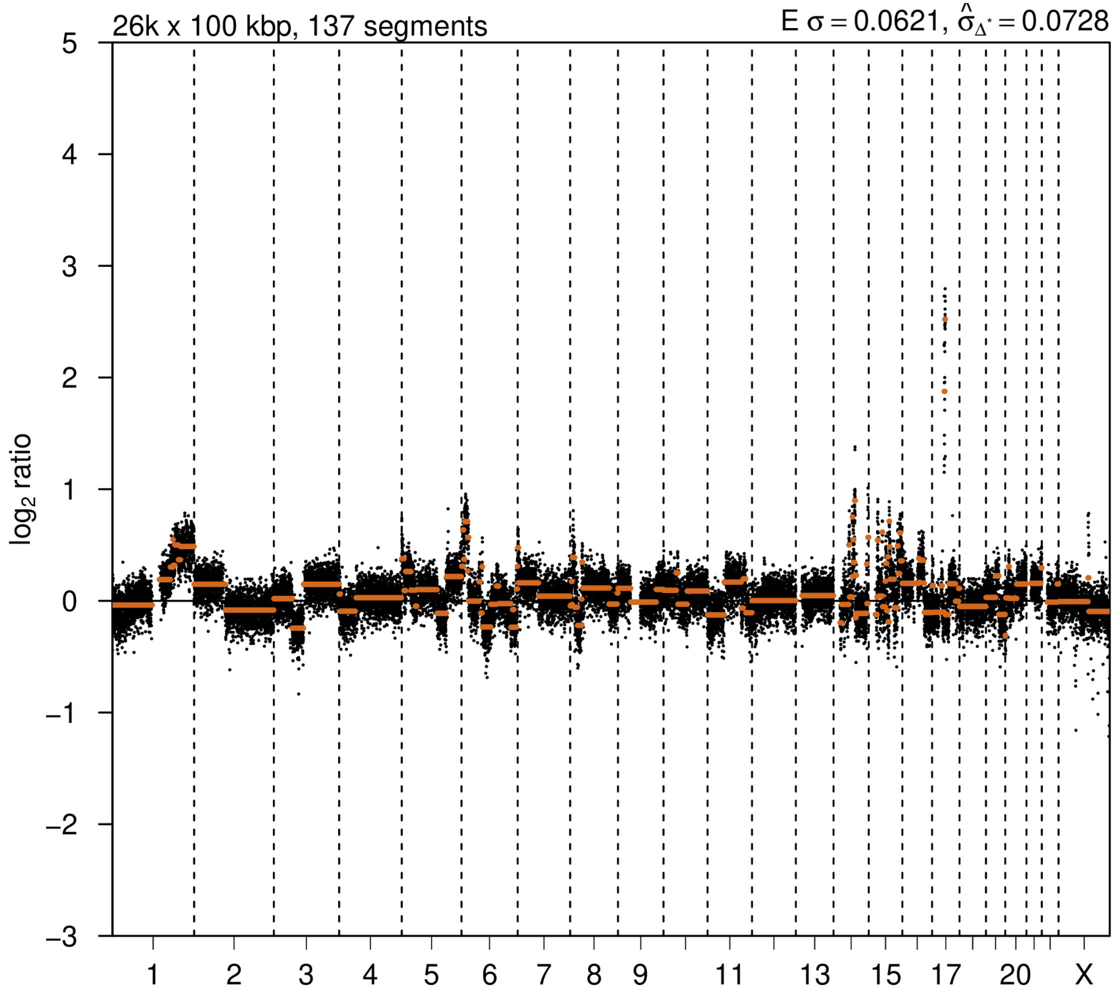

P036

Syn/Meta	Time from 1st surgery to 2nd event (Months)	Side 2nd event	Histology 2nd event	Surgery	AdjuvanT Treatment Pri (RT/ HT)	ER Pri	ER 2nd event	Her2 Pri	Her2 2nd event	Grade Pri	Grade 2nd event	Quadrant 2nd event	Margins	Screening	Clonality P value Copy N	Clonality P value Panel seq	Clonality P value WES	Final verdict
metachronous	125	Ipsilateral	IDC with DCIS	lumpectomy	None	+	+	-	-	2	2	at or adjacent to primary	Clear	screen-detected	0.000324 57	0.003	NA	Related

Primary event

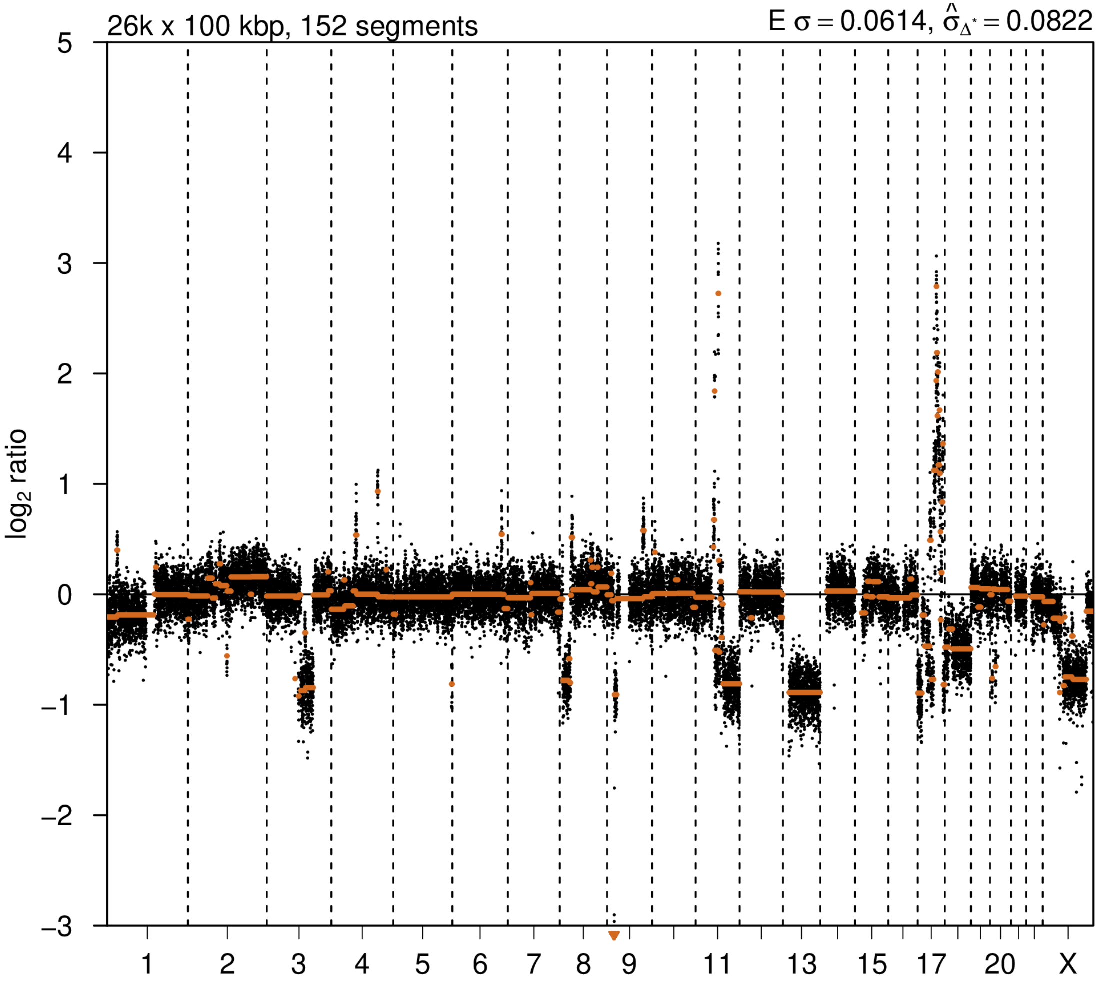

2nd event

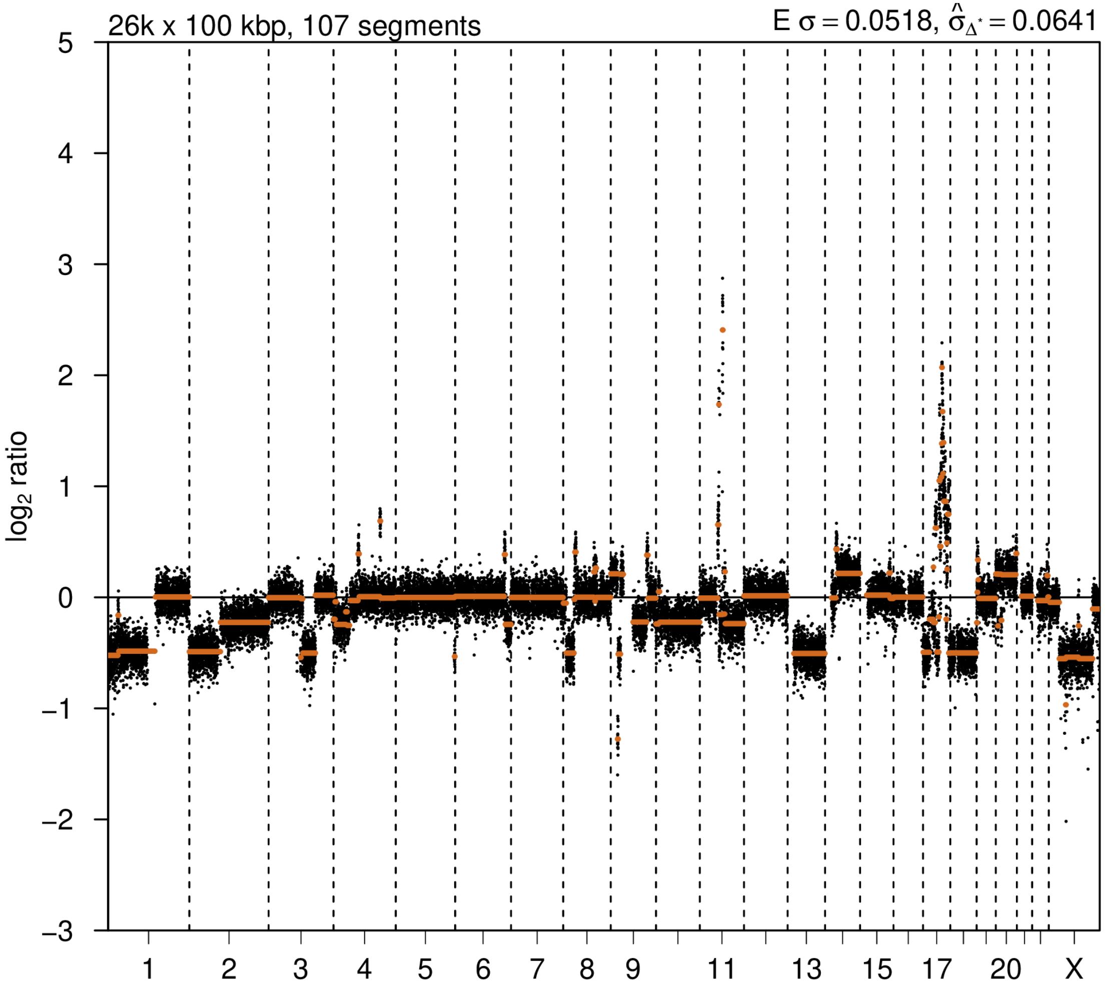

P038

Syn/Meta	Time from 1st surgery to 2nd event (Months)	Side 2nd event	Histology 2nd event	Surgery	AdjuvanT Treatment Pri (RT/ HT)	ER Pri	ER 2nd event	Her2 Pri	Her2 2nd event	Grade Pri	Grade 2nd event	Quadrant 2nd event	Margins	Screening	Clonality P value Copy N	Clonality P value Panel seq	Clonality P value WES	Final verdict
metachronous	100	Ipsilateral	IDC no DCIS	lumpectomy	None	+	+	-	-	2	3	distant from primary	involved	symptomatic	0.00032457	NA	NA	Related

Primary event

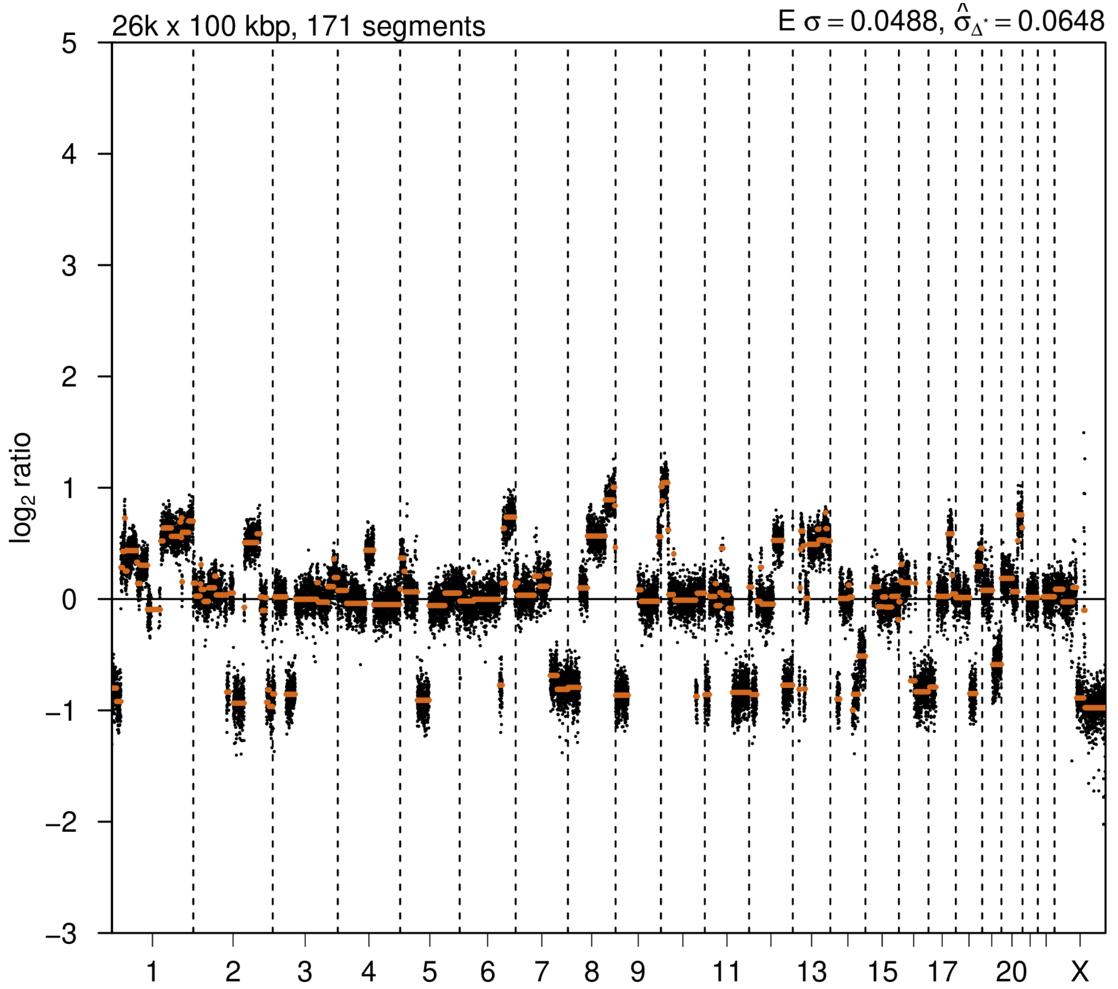

2nd event

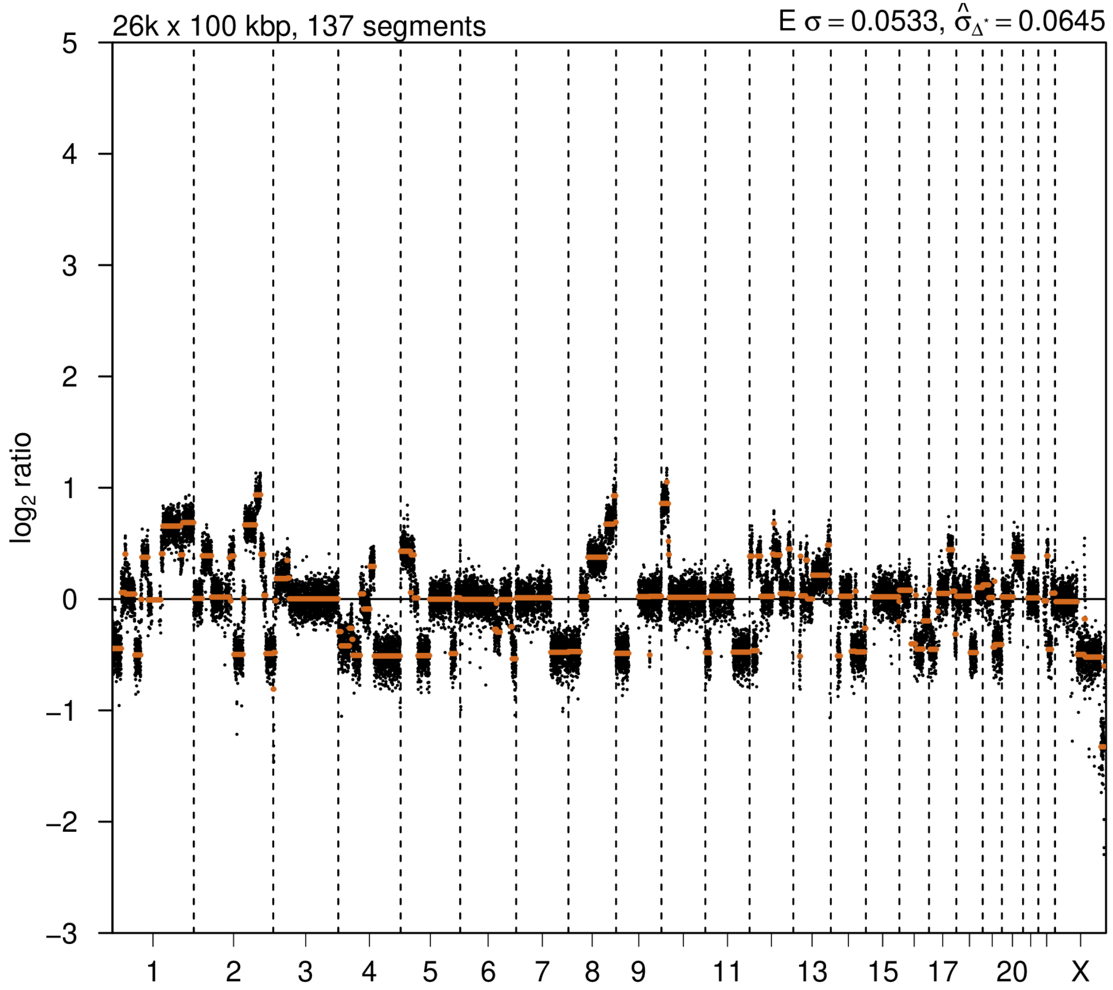

P039

Syn/Meta	Time from 1st surgery to 2nd event (Months)	Side 2nd event	Histology 2nd event	Surgery	AdjuvanT Treatment Pri (RT/ HT)	ER Pri	ER 2nd event	Her2 Pri	Her2 2nd event	Grade Pri	Grade 2nd event	Quadrant 2nd event	Margins	Screening	Clonality P value Copy N	Clonality P value Panel seq	Clonality P value WES	Final verdict
metachronous	66	Ipsilateral	IDC no DCIS	lumpectomy	None	-	-	+	+	3	3	distant from primary	Clear	symptomatic	0.00032457	0.004	NA	Related

Primary event

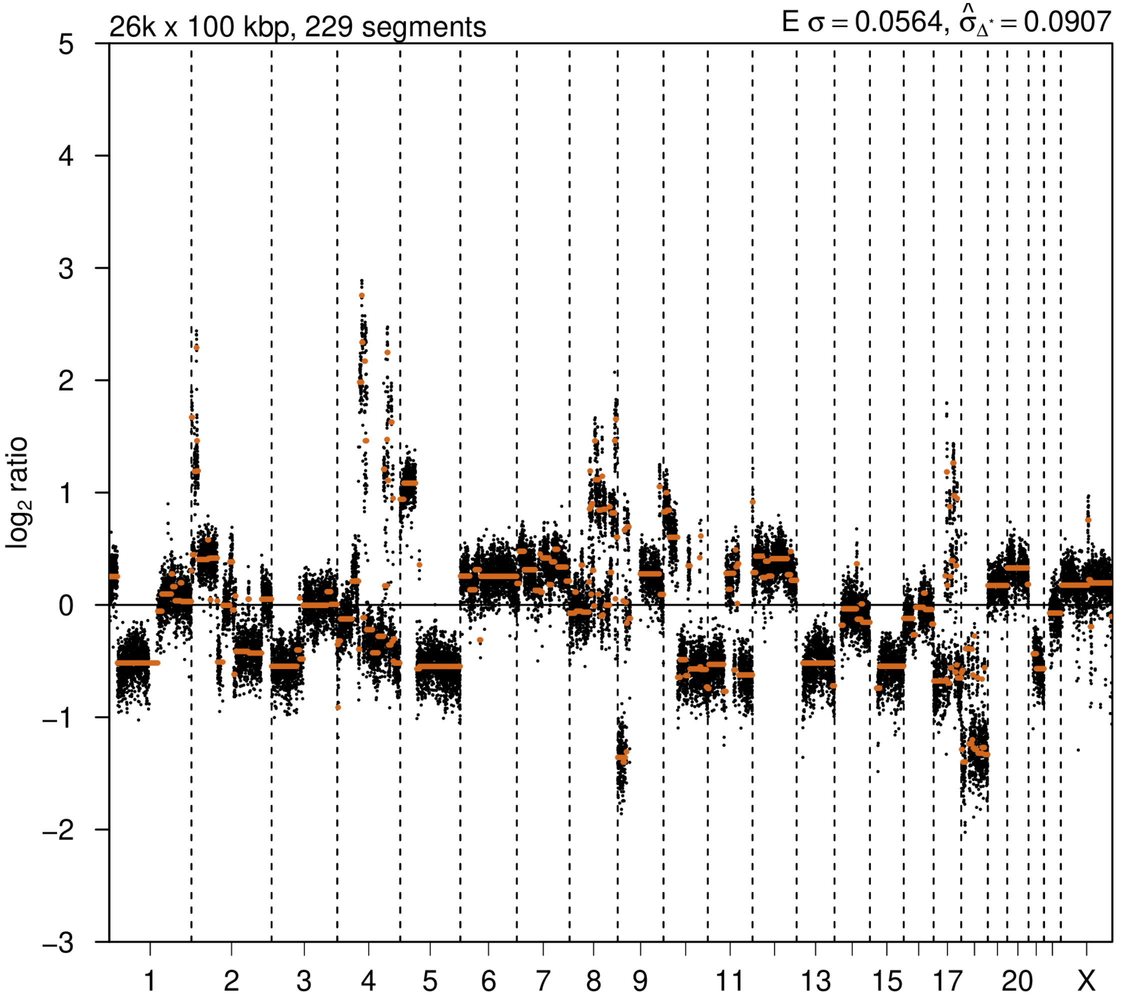

2nd event

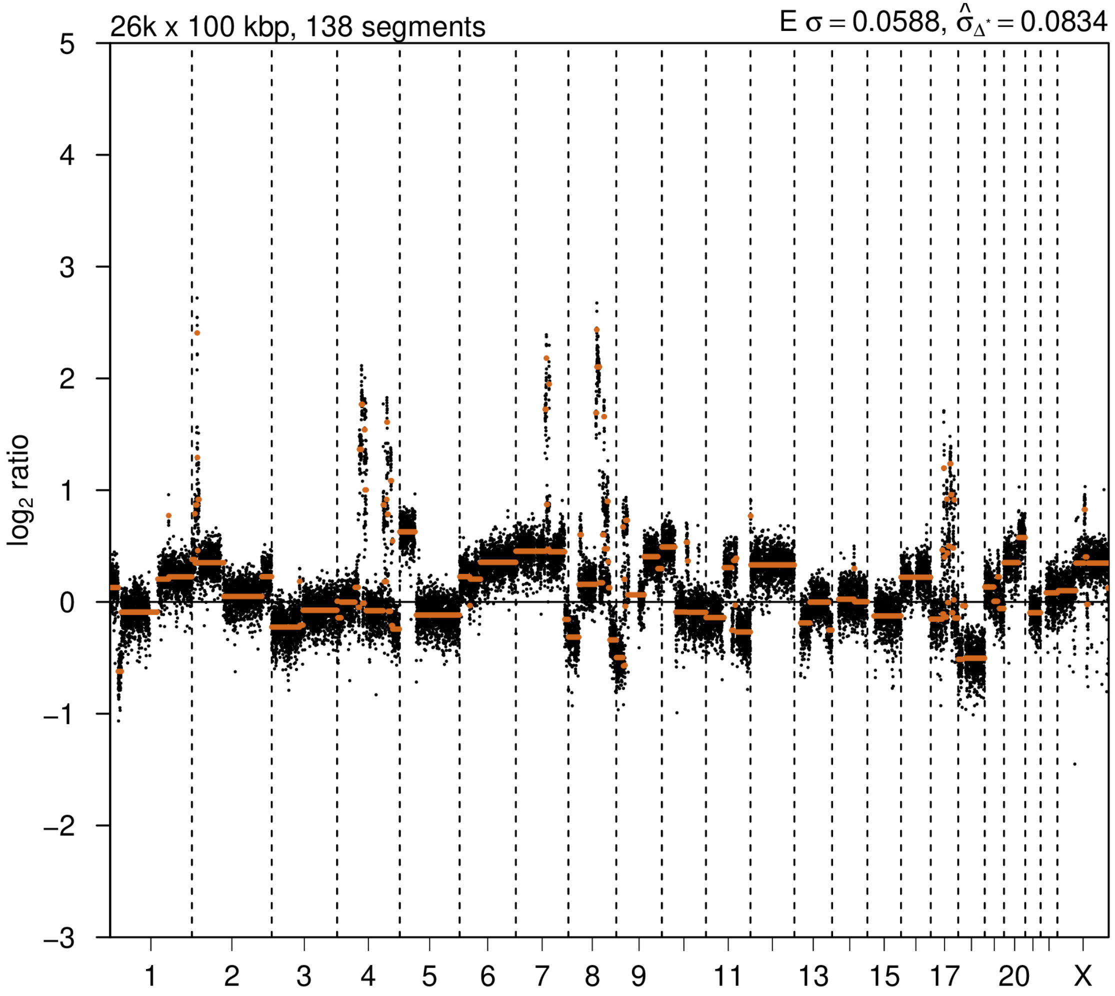

P040

Syn/Meta	Time from 1st surgery to 2nd event (Months)	Side 2nd event	Histology 2nd event	Surgery	AdjuvanT Treatment Pri (RT/ HT)	ER Pri	ER 2nd event	Her2 Pri	Her2 2nd event	Grade Pri	Grade 2nd event	Quadrant 2nd event	Margins	Screening	Clonality P value Copy N	Clonality P value Panel seq	Clonality P value WES	Final verdict
metachronous	84	Ipsilateral	IDC with DCIS	lumpectomy	None	+	+	+	+	1	2	NA	Clear	symptomatic	0.00032457	NA	0.000752445	Related

Primary event

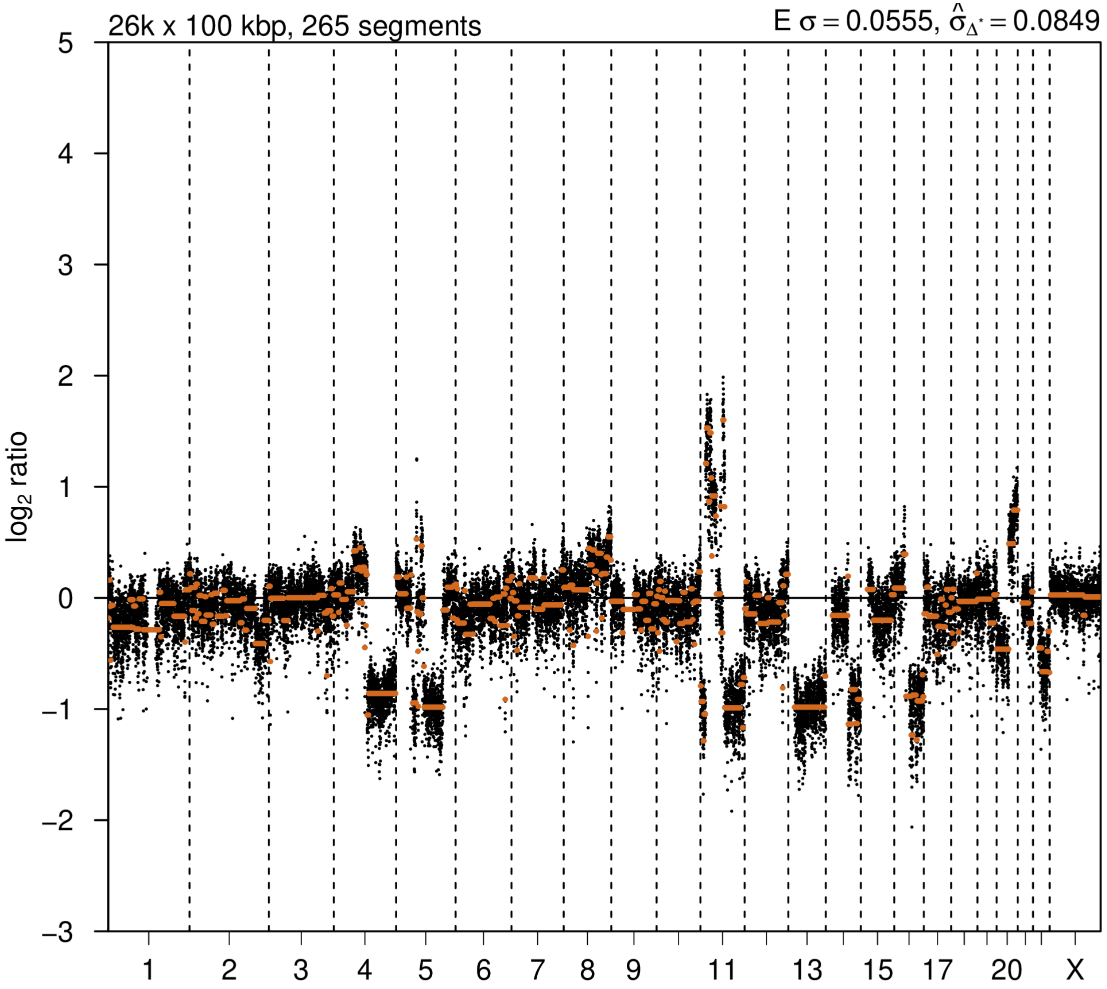

2nd event

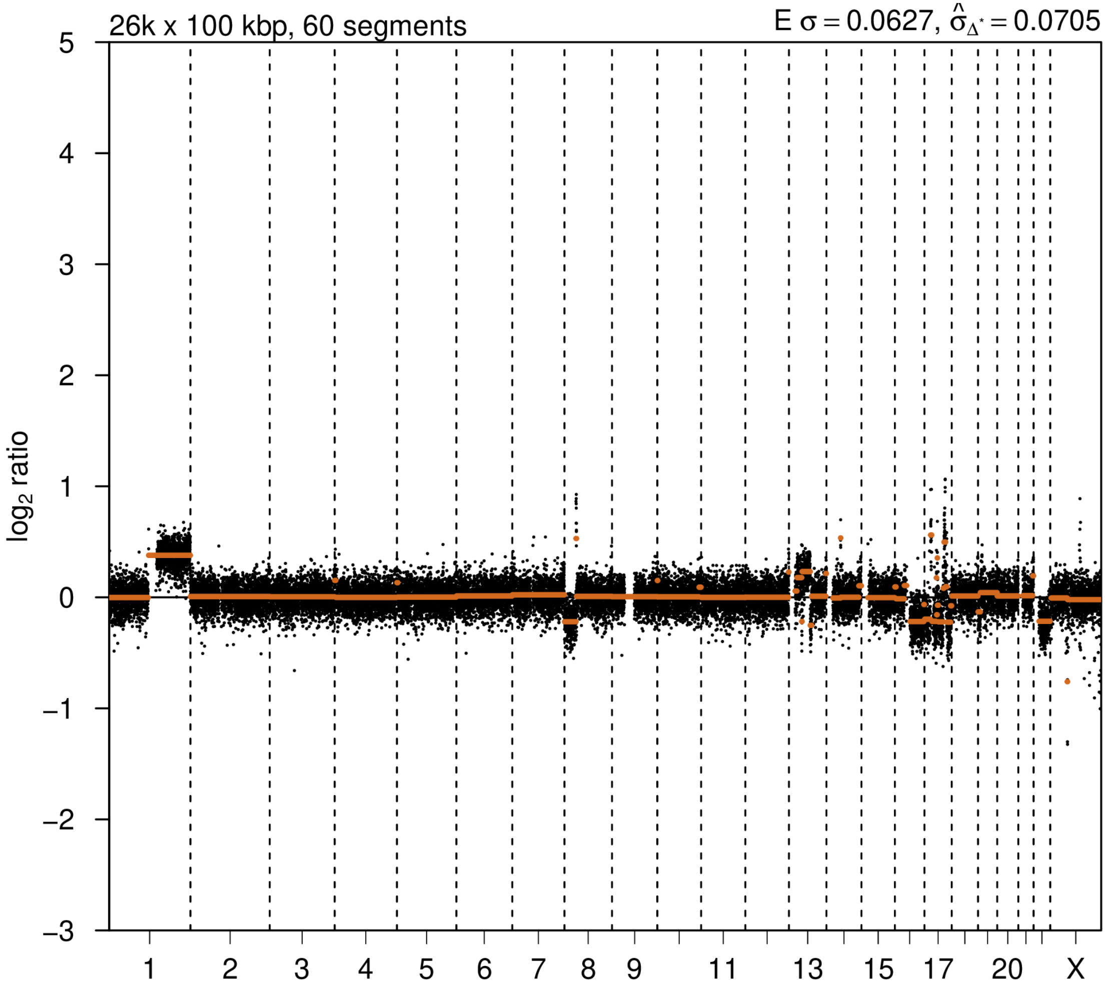

P041

Syn/Meta	Time from 1st surgery to 2nd event (Months)	Side 2nd event	Histology 2nd event	Surgery	AdjuvanT Treatment Pri (RT/ HT)	ER Pri	ER 2nd event	Her2 Pri	Her2 2nd event	Grade Pri	Grade 2nd event	Quadrant 2nd event	Margins	Screening	Clonality P value Copy N	Clonality P value Panel seq	Clonality P value WES	Final verdict
metachronous	97	Ipsilateral	IDC with DCIS	lumpectomy	None	+	+	+	+	2	2	NA	Clear	NA	0.000324 57	Single mutation - shared	NA	Related

Primary event

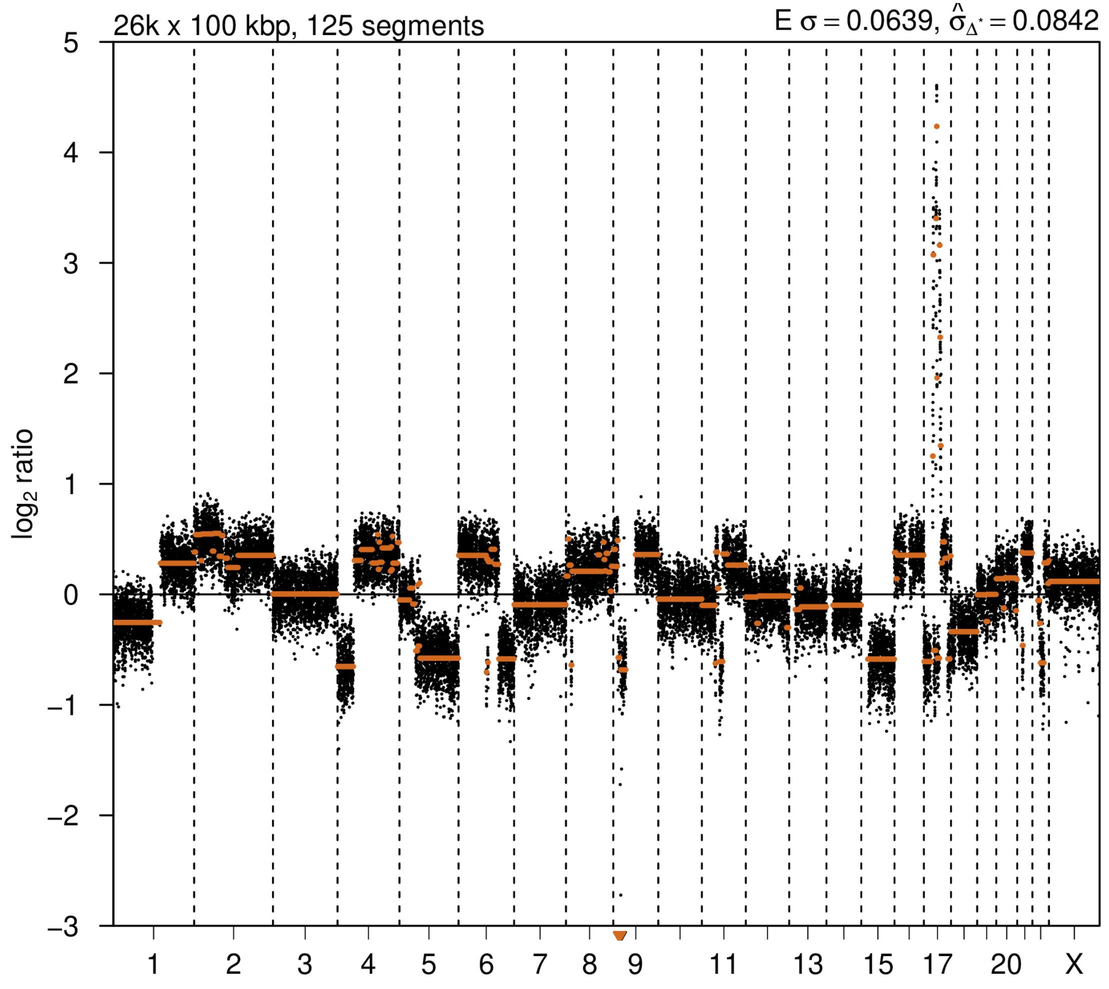

2nd event

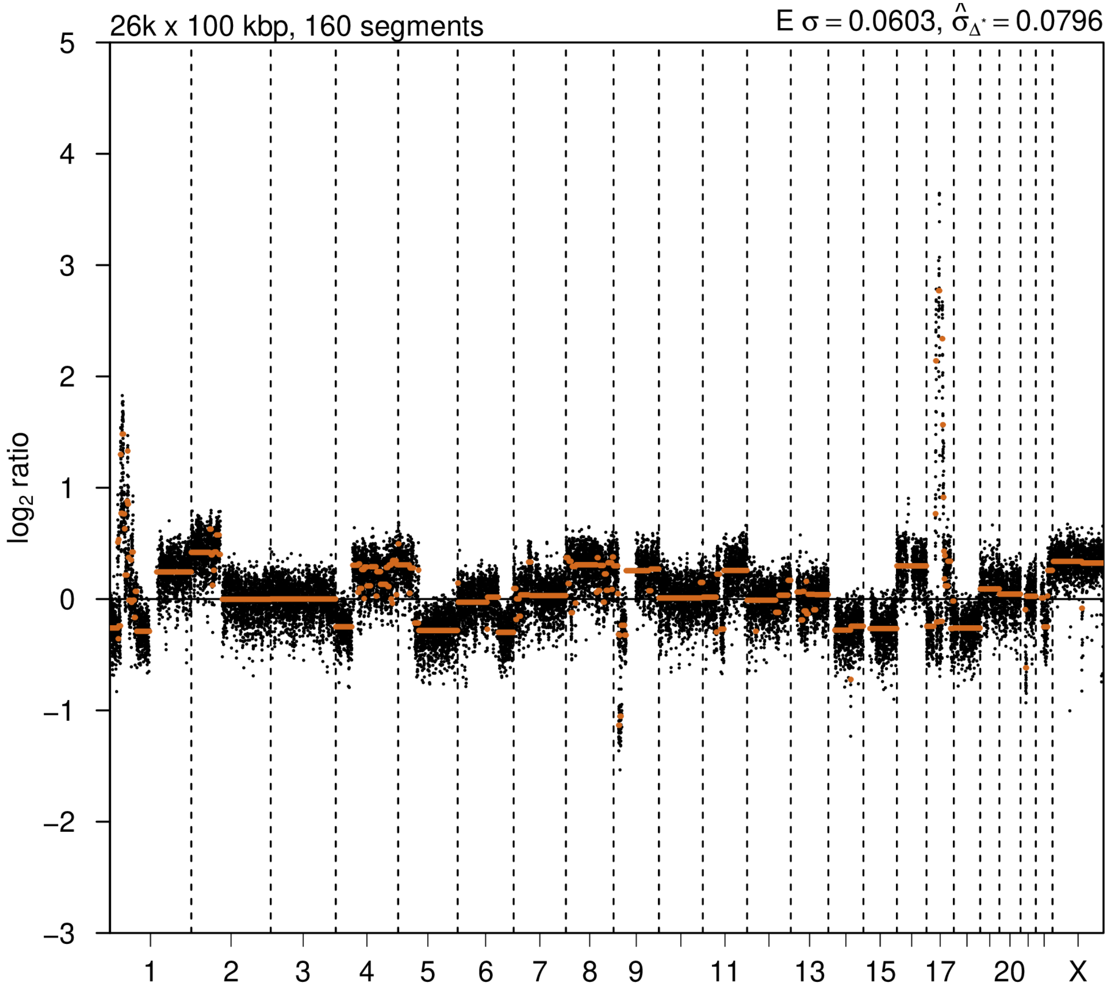

P042

Syn/Meta	Time from 1st surgery to 2nd event (Months)	Side 2nd event	Histology 2nd event	Surgery	AdjuvanT Treatment Pri (RT/ HT)	ER Pri	ER 2nd event	Her2 Pri	Her2 2nd event	Grade Pri	Grade 2nd event	Quadrant 2nd event	Margins	Screening	Clonality P value Copy N	Clonality P value Panel seq	Clonality P value WES	Final verdict
metachronous	72	Ipsilateral	DCIS only	lumpectomy	None	NA	NA	NA	NA	3	3	Unknown	Clear	screen-detected	0.003663	NA	NA	Related

Primary event

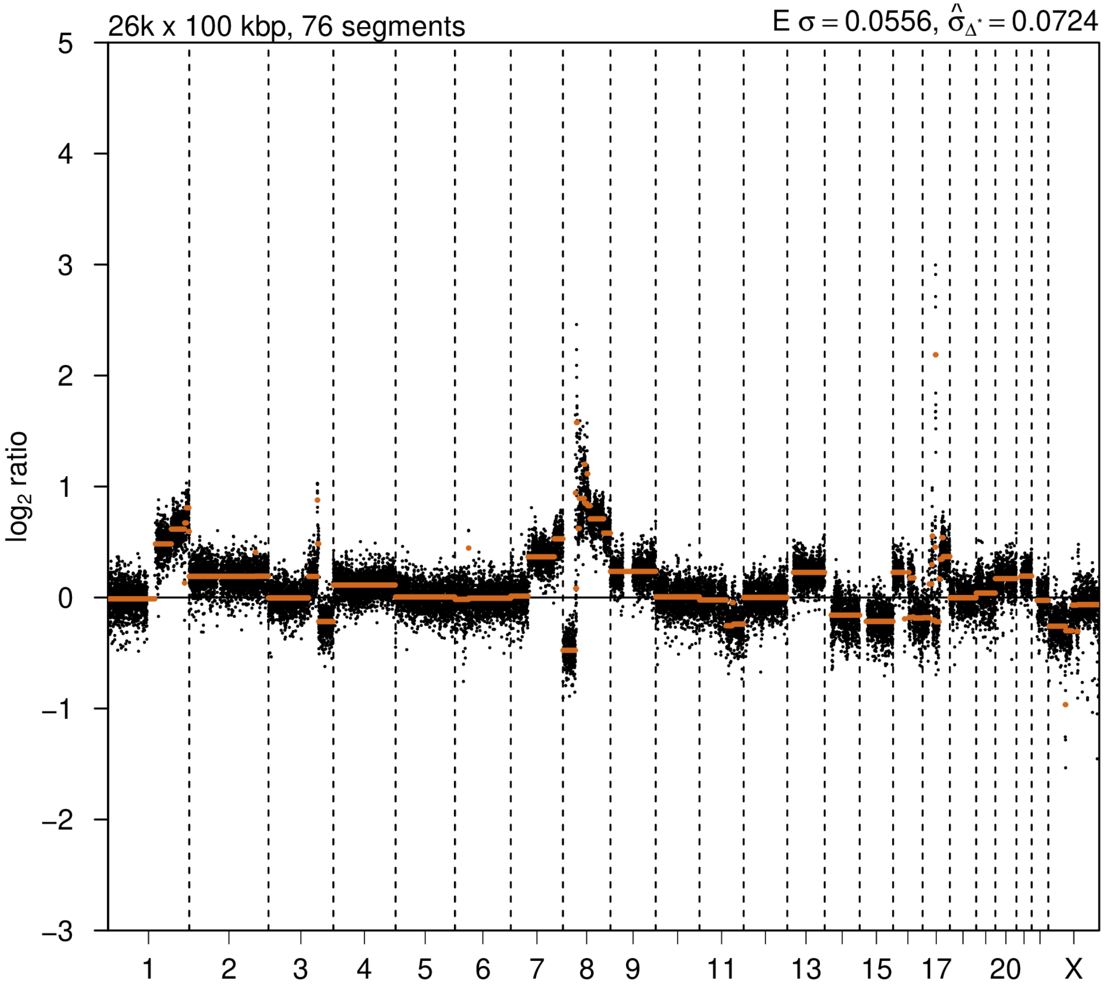

2nd event

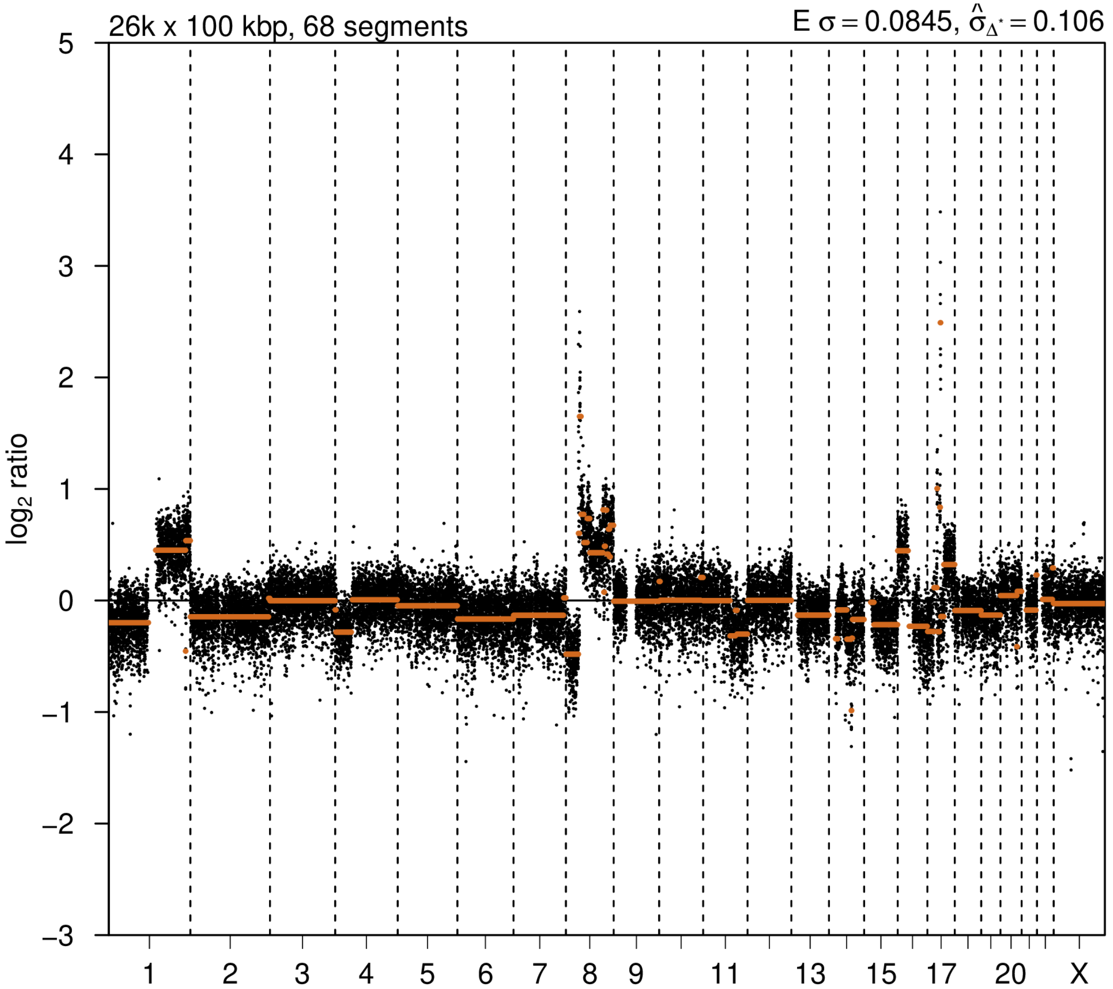

P043

Syn/Meta	Time from 1st surgery to 2nd event (Months)	Side 2nd event	Histology 2nd event	Surgery	AdjuvanT Treatment Pri (RT/ HT)	ER Pri	ER 2nd event	Her2 Pri	Her2 2nd event	Grade Pri	Grade 2nd event	Quadrant 2nd event	Margins	Screening	Clonality P value Copy N	Clonality P value Panel seq	Clonality P value WES	Final verdict
metachronous	74	Ipsilateral	IDC with DCIS	lumpectomy	None	-	+	+	+	2	3	NA	Clear	symptomatic	0.00162285	0.003	0.000752445	Related

Primary event

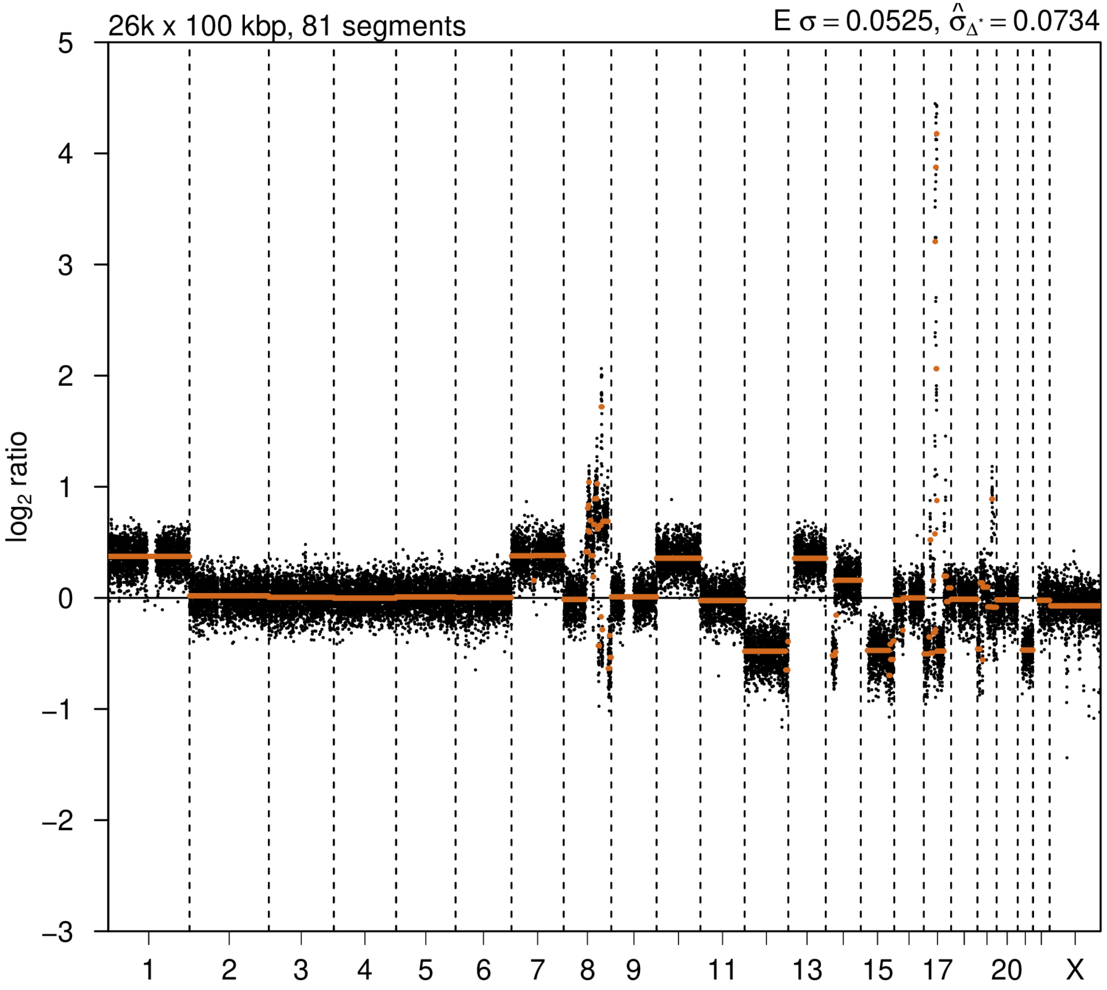

2nd event

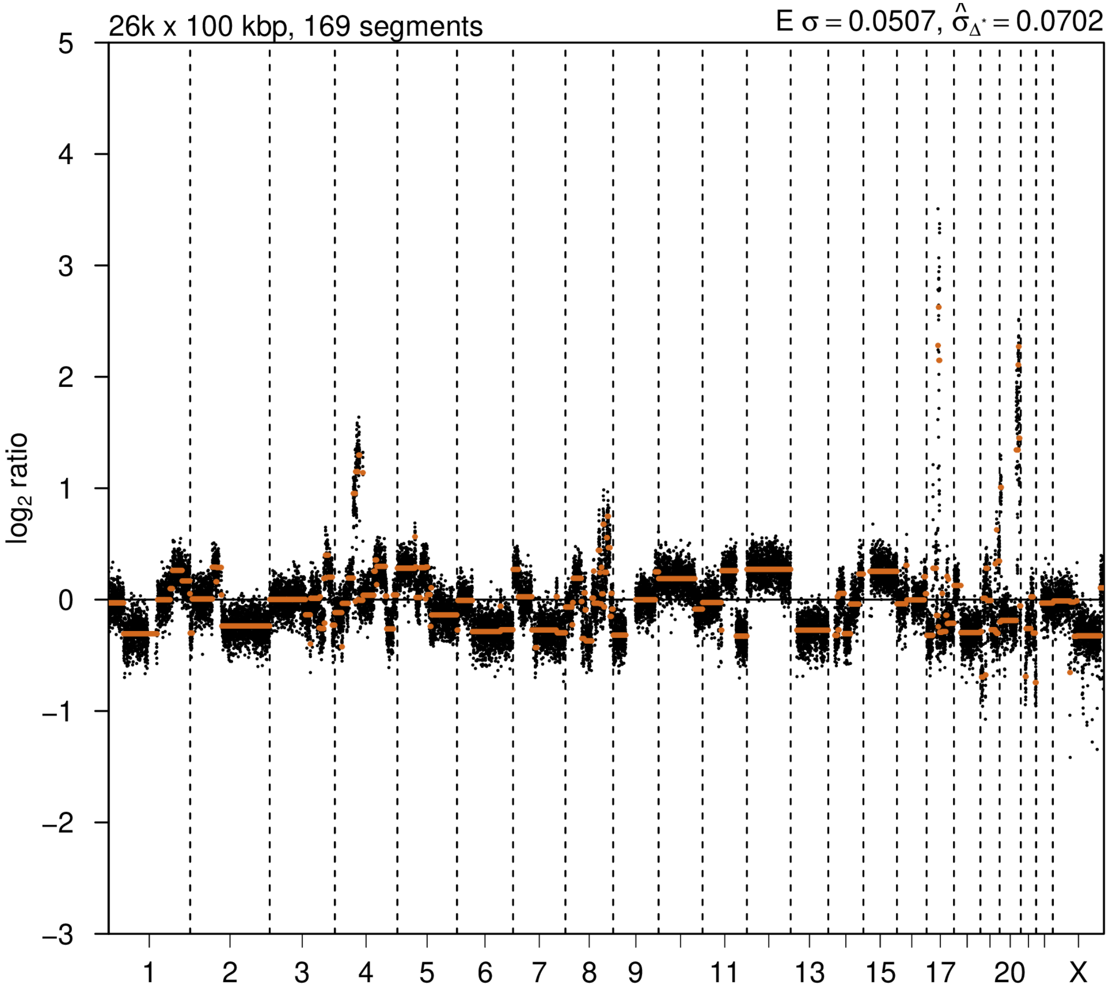

P044

Syn/Meta	Time from 1st surgery to 2nd event (Months)	Side 2nd event	Histology 2nd event	Surgery	AdjuvanT Treatment Pri (RT/ HT)	ER Pri	ER 2nd event	Her2 Pri	Her2 2nd event	Grade Pri	Grade 2nd event	Quadrant 2nd event	Margins	Screening	Clonality P value Copy N	Clonality P value Panel seq	Clonality P value WES	Final verdict
metachronous	109	Ipsilateral	IDC no DCIS	lumpectomy	None	+	+	-	-	2	3	NA	Clear	NA	0.000324 57	NA	NA	Related

Primary event

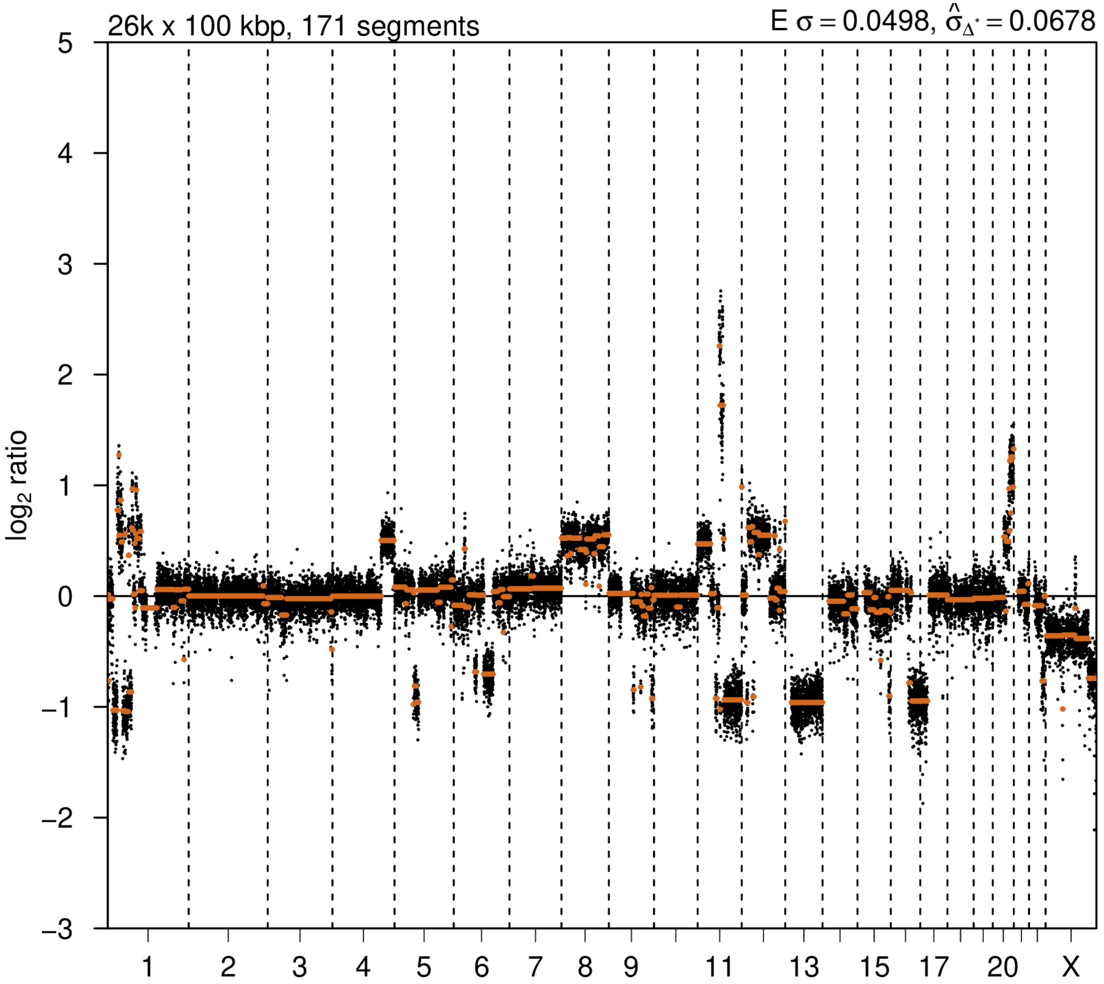

2nd event

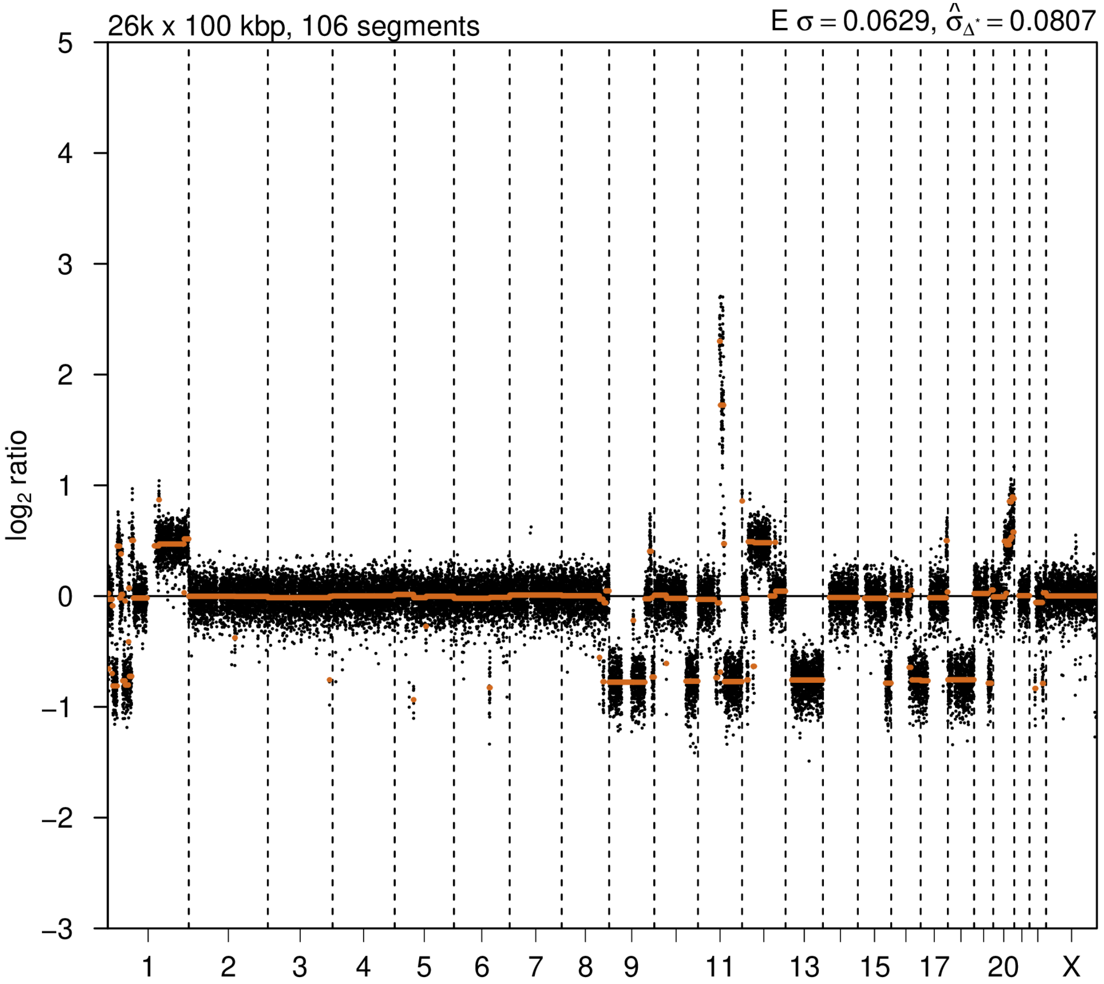

P045

Syn/Meta	Time from 1st surgery to 2nd event (Months)	Side 2nd event	Histology 2nd event	Surgery	AdjuvanT Treatment Pri (RT/ HT)	ER Pri	ER 2nd event	Her2 Pri	Her2 2nd event	Grade Pri	Grade 2nd event	Quadrant 2nd event	Margins	Screening	Clonality P value Copy N	Clonality P value Panel seq	Clonality P value WES	Final verdict
metachronous	60	Ipsilateral	IDC no DCIS	lumpectomy	None	+	+	-	-	2	2	NA	involved	symptomatic	0.00032457	NA	NA	Related

Primary event

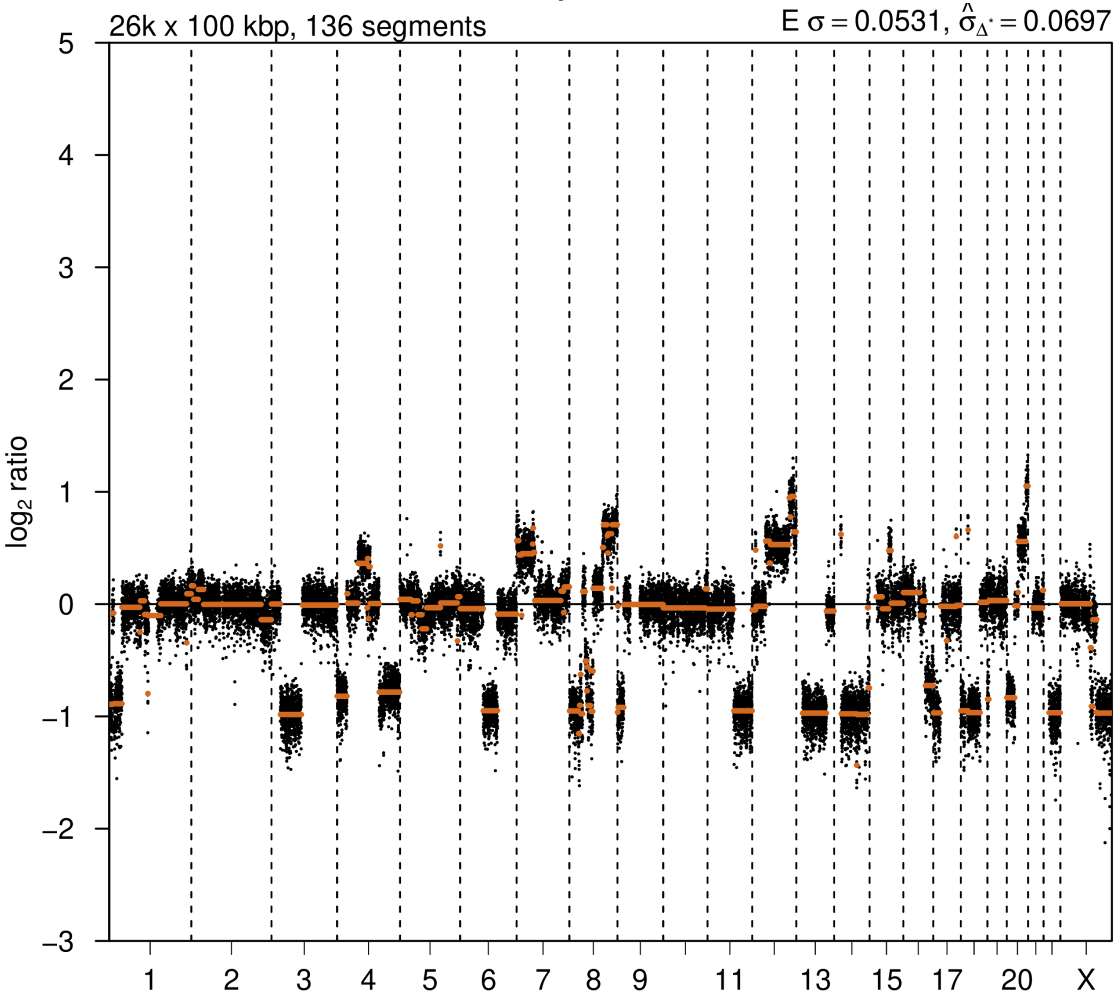

2nd event

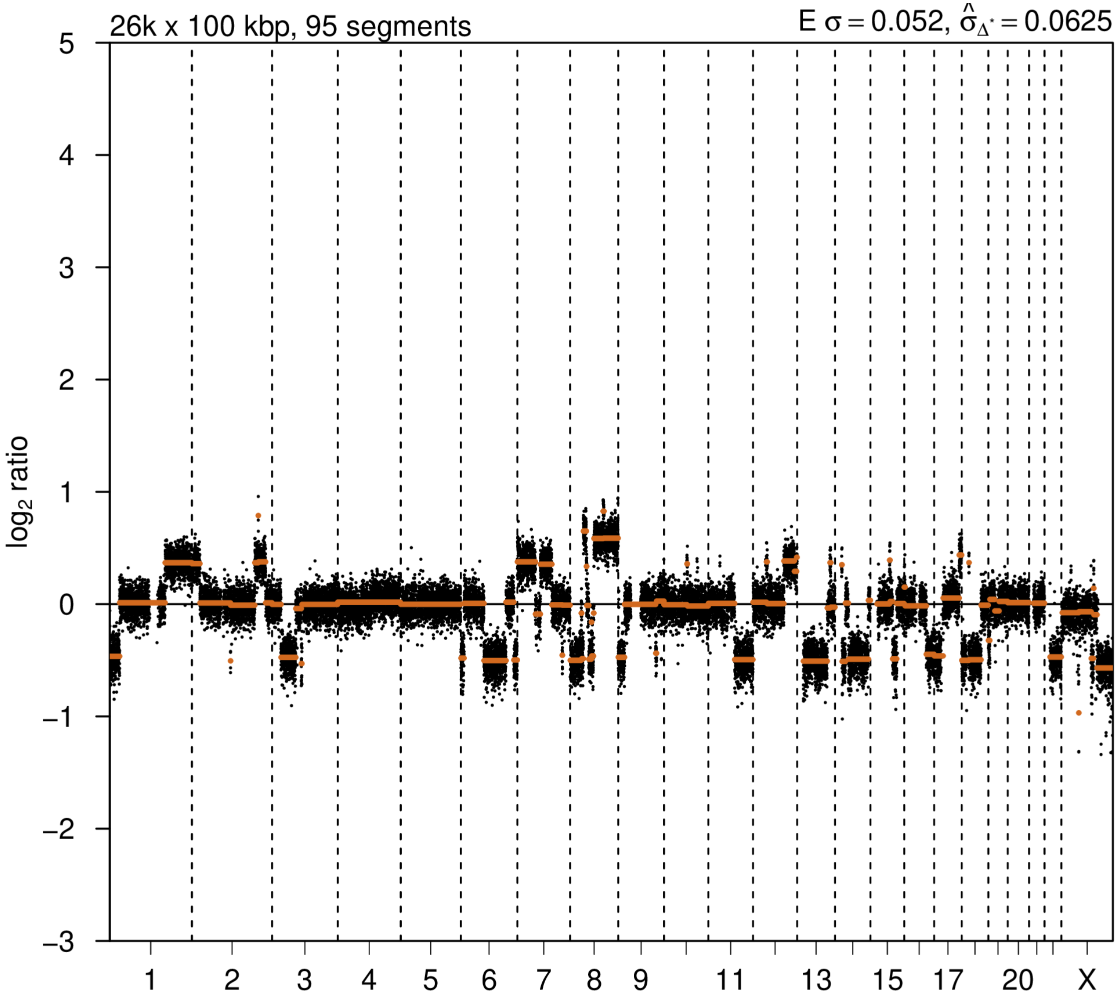

P046

Syn/Meta	Time from 1st surgery to 2nd event (Months)	Side 2nd event	Histology 2nd event	Surgery	AdjuvanT Treatment Pri (RT/ HT)	ER Pri	ER 2nd event	Her2 Pri	Her2 2nd event	Grade Pri	Grade 2nd event	Quadrant 2nd event	Margins	Screening	Clonality P value Copy N	Clonality P value Panel seq	Clonality P value WES	Final verdict
metachronous	95	Ipsilateral	IDC with DCIS	lumpectomy	None	+	+	-	-	2	2	NA	Clear	screen-detected	0.780266147	0.006	NA	Related

Primary event

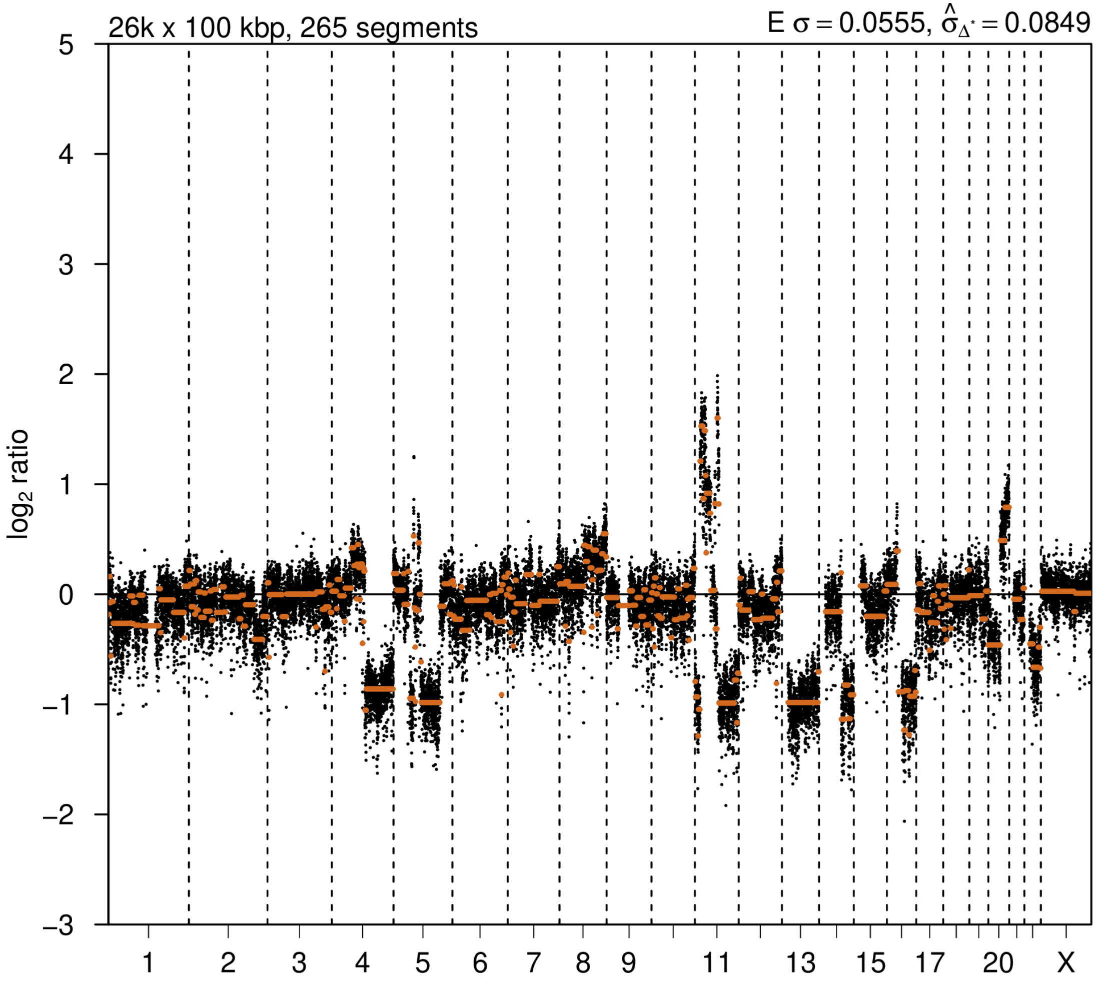

2nd event

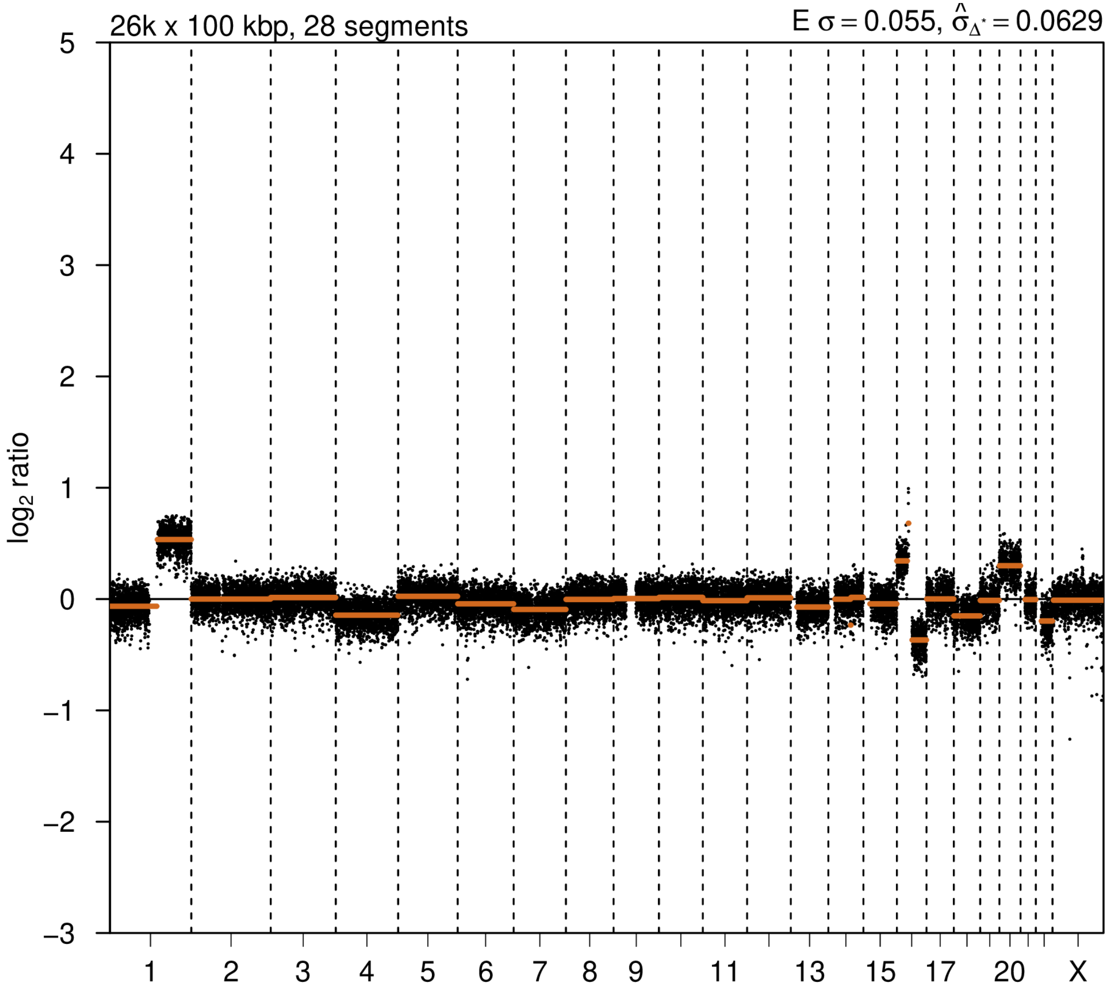

P050

Syn/Meta	Time from 1st surgery to 2nd event (Months)	Side 2nd event	Histology 2nd event	Surgery	AdjuvanT Treatment Pri (RT/ HT)	ER Pri	ER 2nd event	Her2 Pri	Her2 2nd event	Grade Pri	Grade 2nd event	Quadrant 2nd event	Margins	Screening	Clonality P value Copy N	Clonality P value Panel seq	Clonality P value WES	Final verdict
metachronous	88	Ipsilateral	Invasive	lumpectomy	None	+	+	+	+	2	3	at or adjacent to primary	Clear	screen-detected	0.000649 14	Single mutation - shared	NA	Related

Primary event

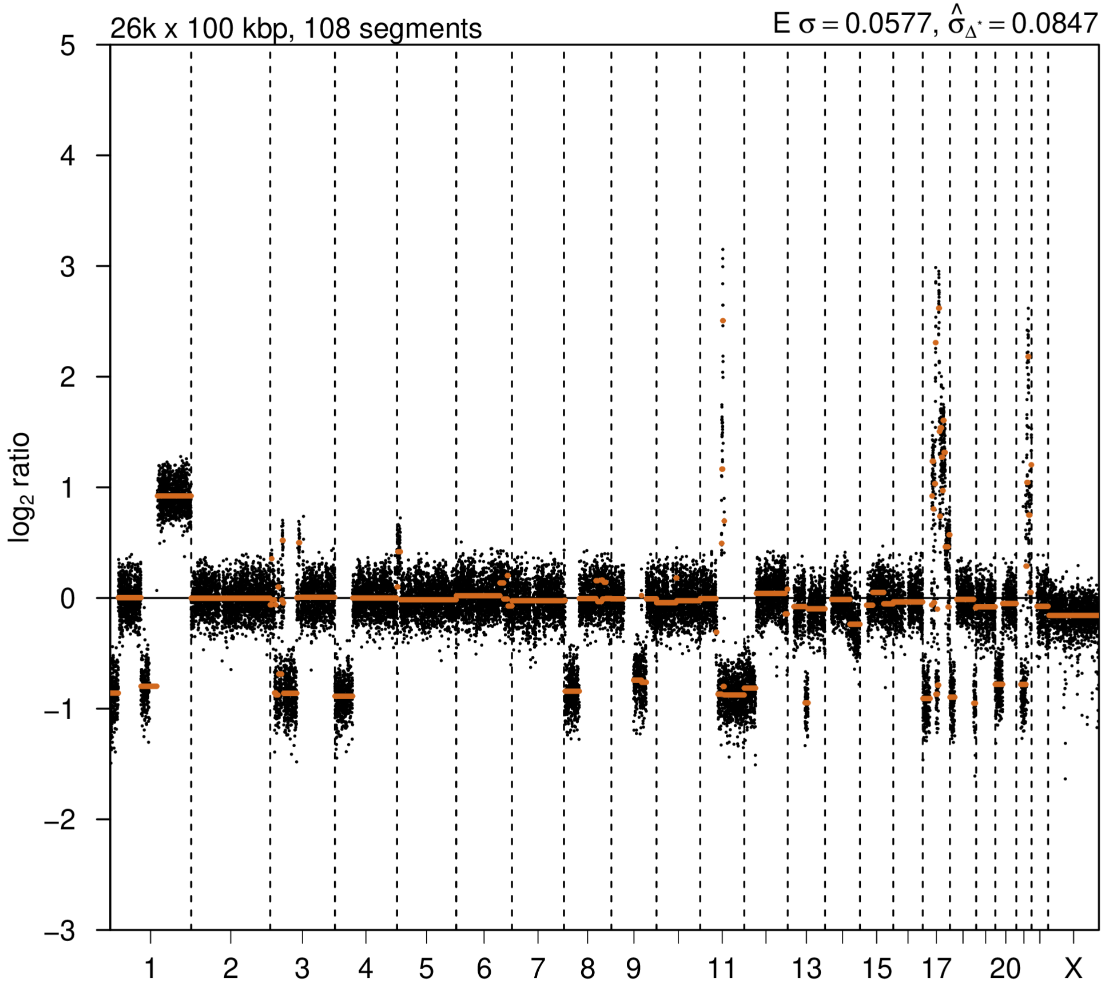

2nd event

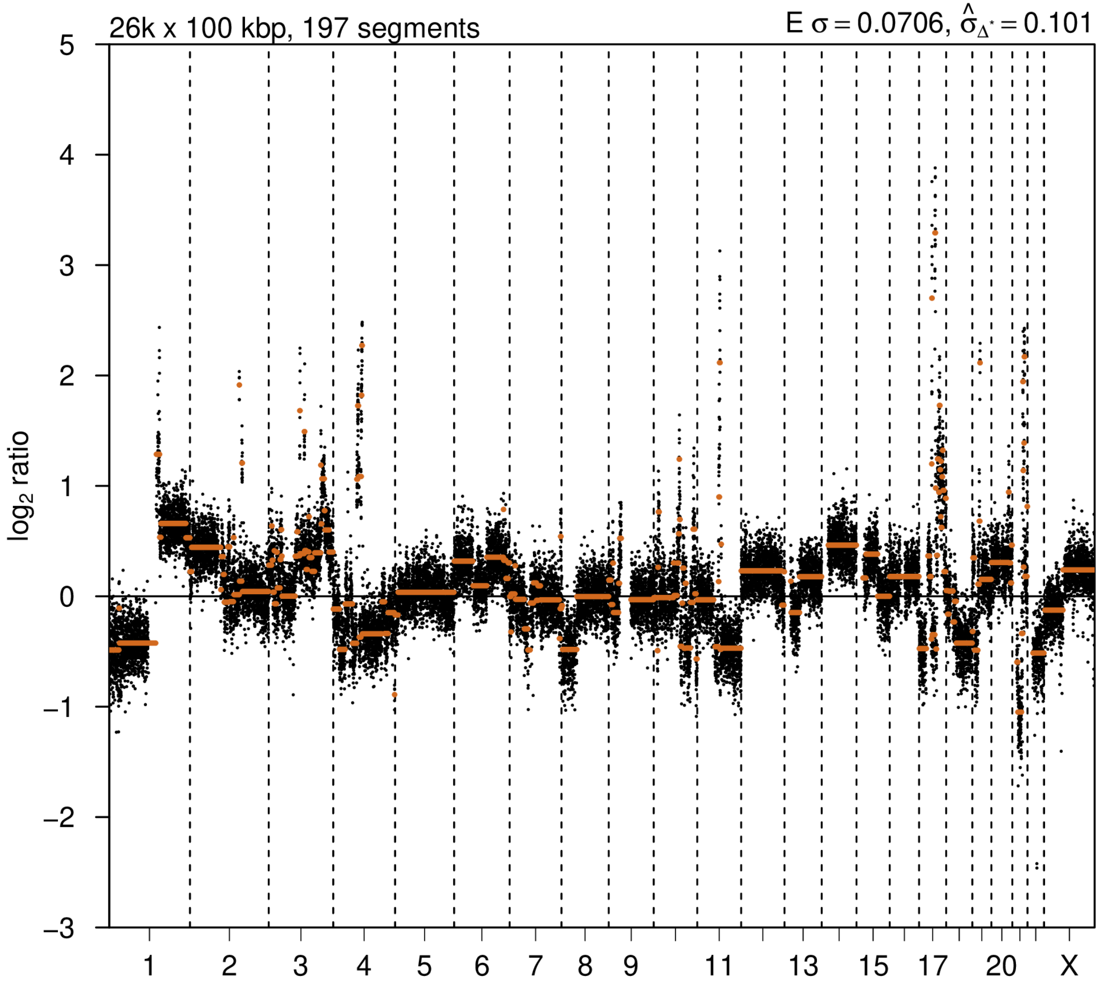

P051

Syn/Meta	Time from 1st surgery to 2nd event (Months)	Side 2nd event	Histology 2nd event	Surgery	AdjuvanT Treatment Pri (RT/ HT)	ER Pri	ER 2nd event	Her2 Pri	Her2 2nd event	Grade Pri	Grade 2nd event	Quadrant 2nd event	Margins	Screening	Clonality P value Copy N	Clonality P value Panel seq	Clonality P value WES	Final verdict
metachronous	62	Ipsilateral	IDC with DCIS	lumpectomy	None	+	+	+	+	2	3	at or adjacent to primary	Clear	screen-detected	0.000649 14	Single mutation - shared	NA	Related

Primary event

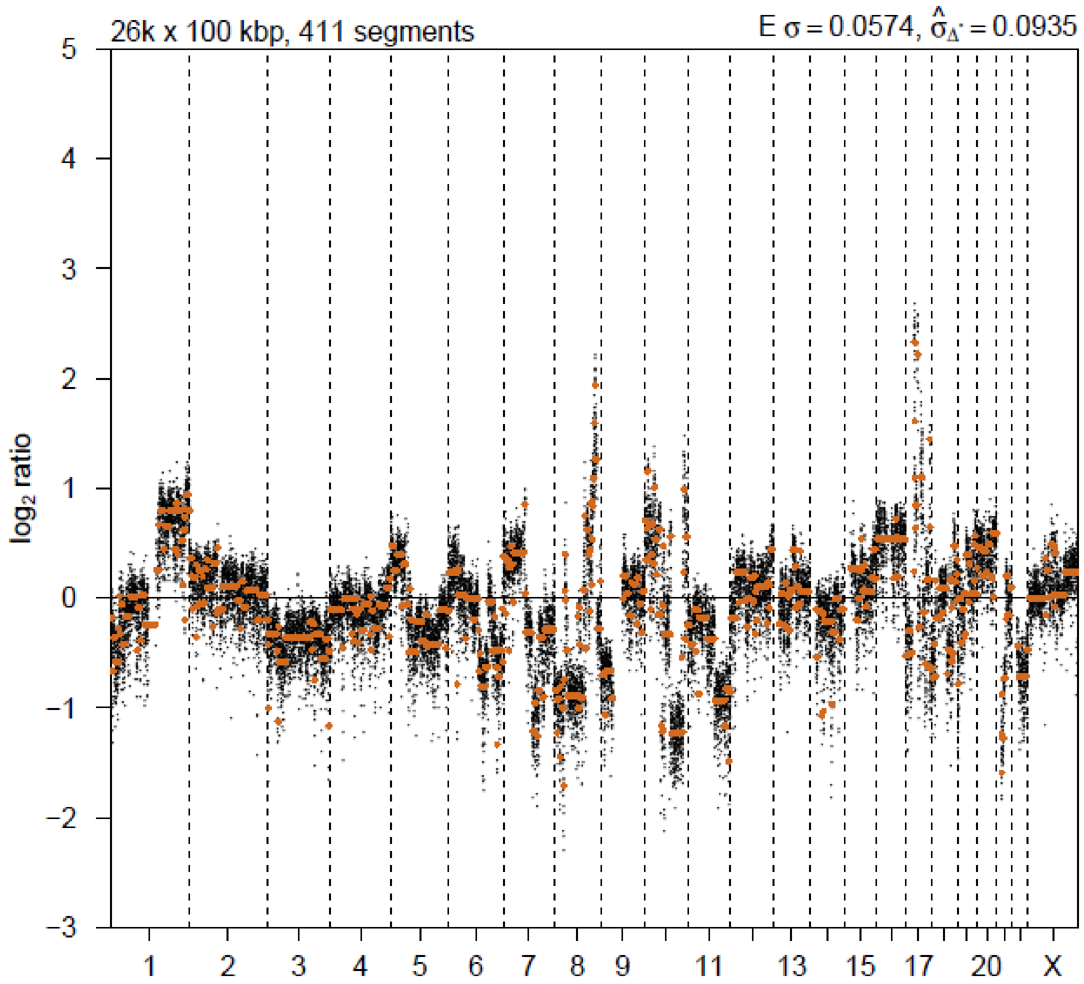

2nd event

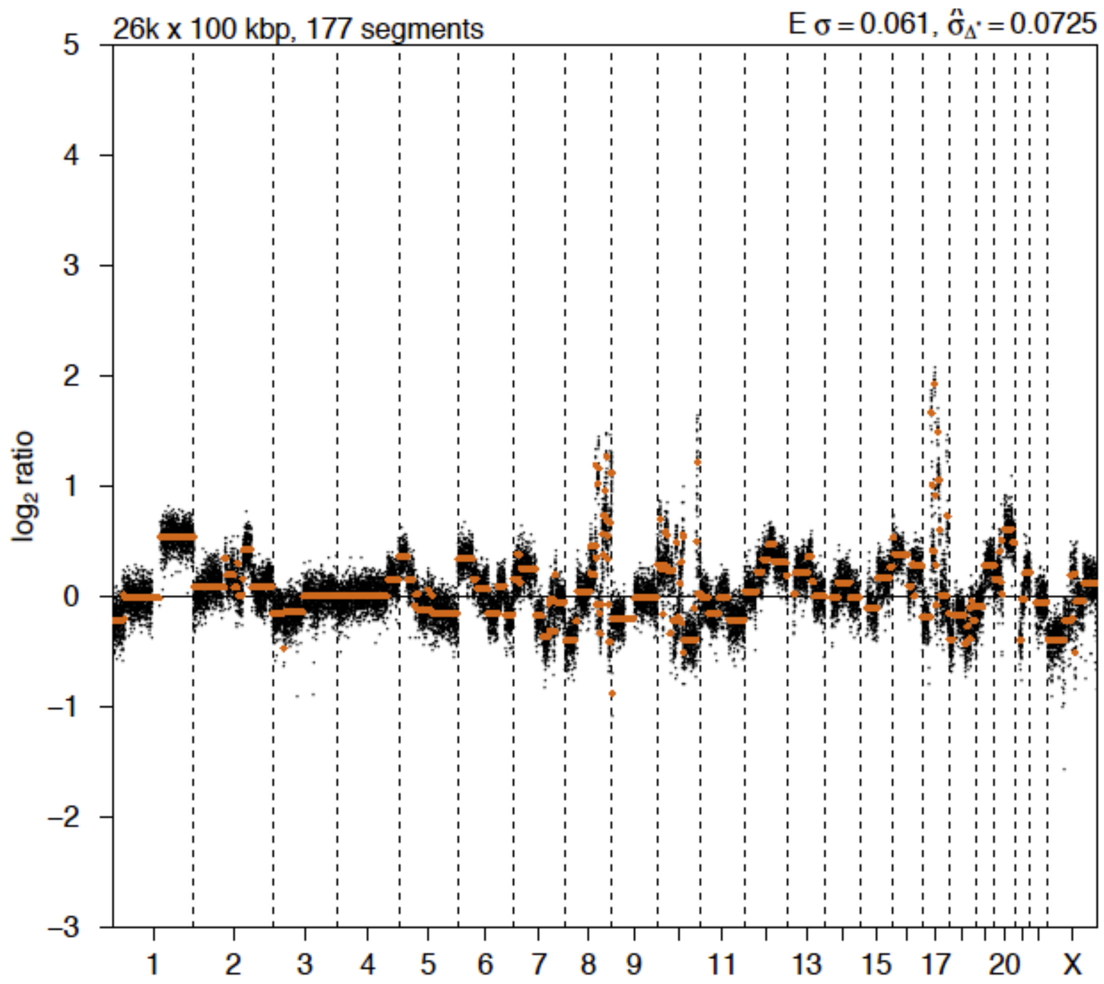

P052

Syn/Meta	Time from 1st surgery to 2nd event (Months)	Side 2nd event	Histology 2nd event	Surgery	AdjuvanT Treatment Pri (RT/ HT)	ER Pri	ER 2nd event	Her2 Pri	Her2 2nd event	Grade Pri	Grade 2nd event	Quadrant 2nd event	Margins	Screening	Clonality P value Copy N	Clonality P value Panel seq	Clonality P value WES	Final verdict
metachronous	86	Ipsilateral	IDC with DCIS	lumpectomy	None	+	+	-	-	2	2	at or adjacent to primary	Clear	symptomatic	0.00032457	0.003	NA	Related

Primary event

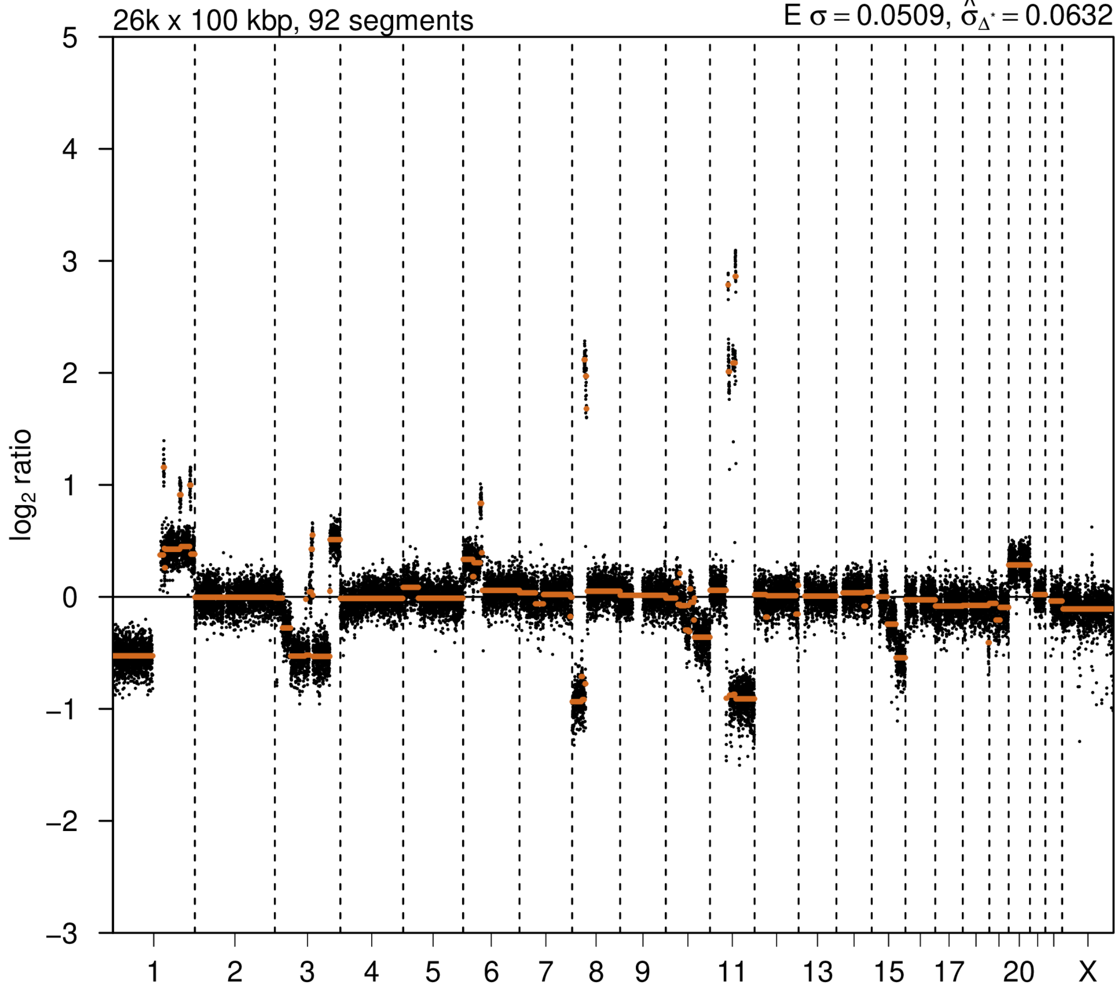

2nd event

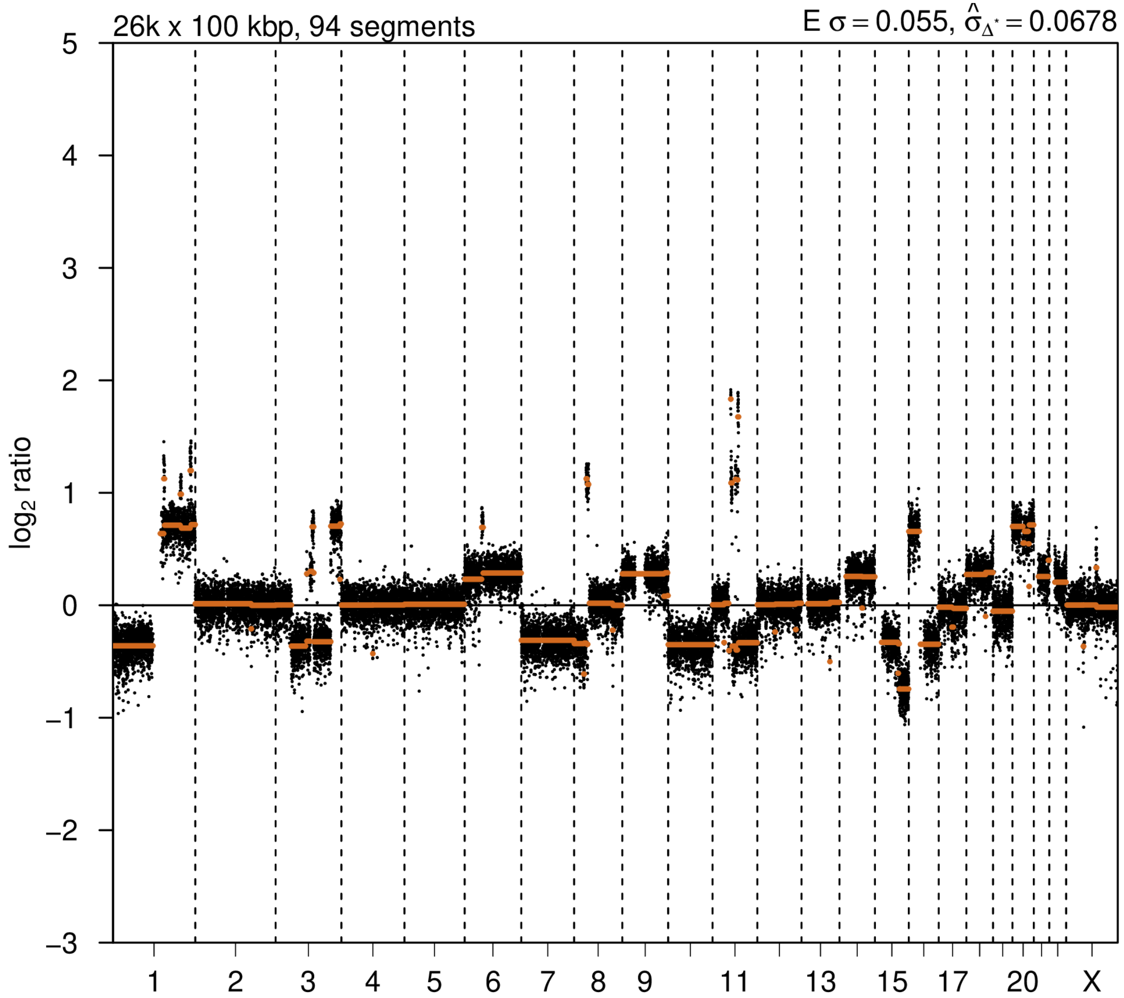

P053

Syn/Meta	Time from 1st surgery to 2nd event (Months)	Side 2nd event	Histology 2nd event	Surgery	AdjuvanT Treatment Pri (RT/ HT)	ER Pri	ER 2nd event	Her2 Pri	Her2 2nd event	Grade Pri	Grade 2nd event	Quadrant 2nd event	Margins	Screening	Clonality P value Copy N	Clonality P value Panel seq	Clonality P value WES	Final verdict
metachronous	72	Ipsilateral	DCIS only	lumpectomy	None	NA	NA	NA	NA	3	3	Unknown	Clear	screen-detected	0.003663	NA	NA	Related

Primary event

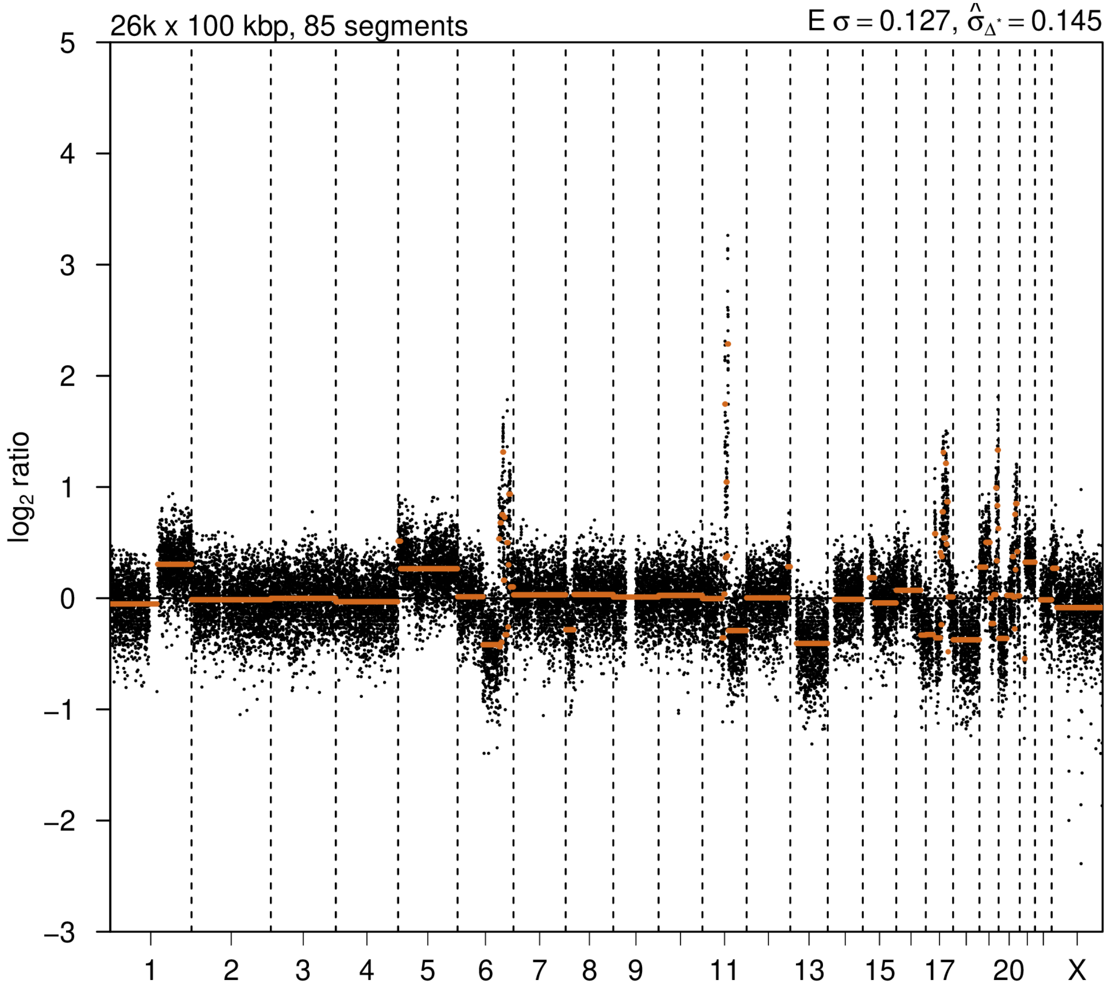

2nd event

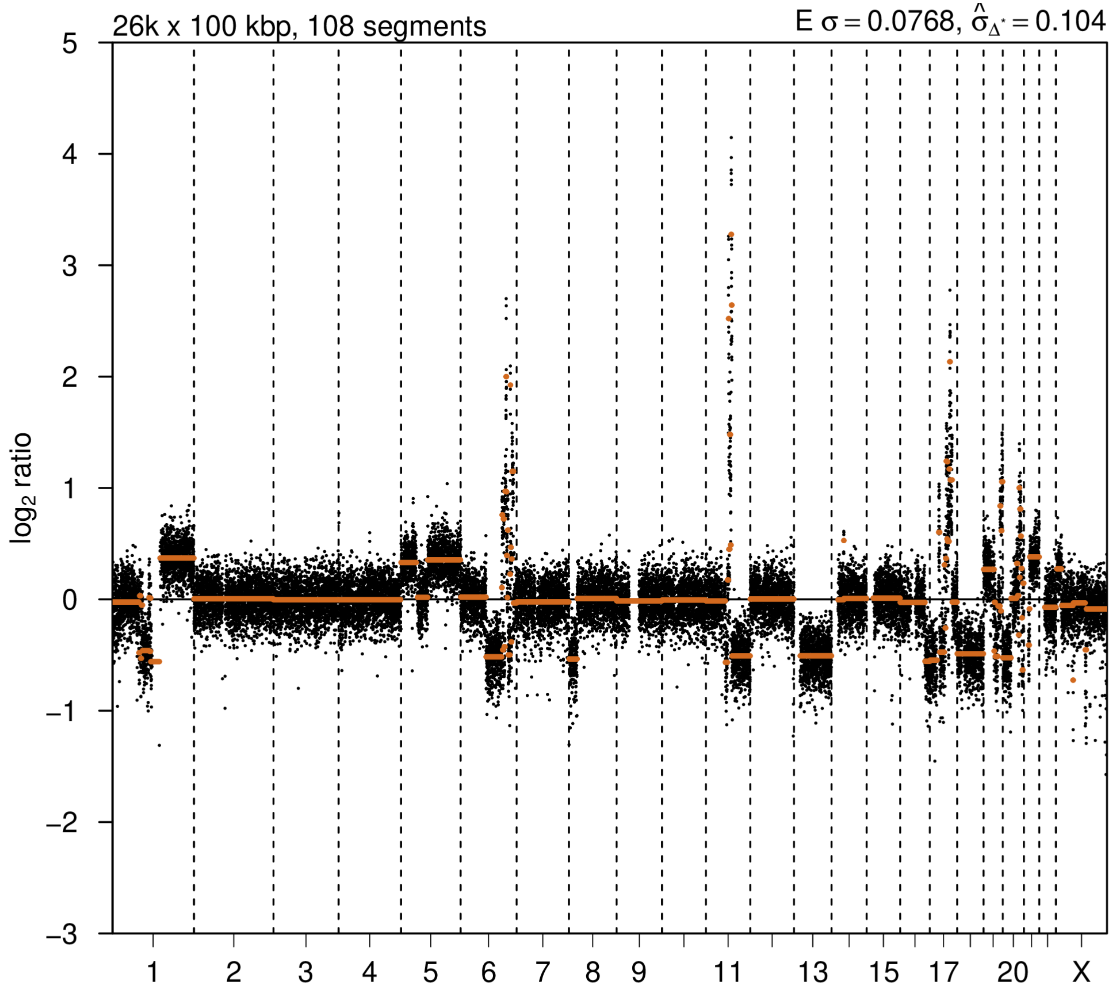

P054

Syn/Meta	Time from 1st surgery to 2nd event (Months)	Side 2nd event	Histology 2nd event	Surgery	AdjuvanT Treatment Pri (RT/ HT)	ER Pri	ER 2nd event	Her2 Pri	Her2 2nd event	Grade Pri	Grade 2nd event	Quadrant 2nd event	Margins	Screening	Clonality P value Copy N	Clonality P value Panel seq	Clonality P value WES	Final verdict
metachronous	63	Ipsilateral	IDC with DCIS	lumpectomy	None	+	+	-	-	1	3	at or adjacent to primary	NA	screen-detected	0.000324 57	NA	NA	Related

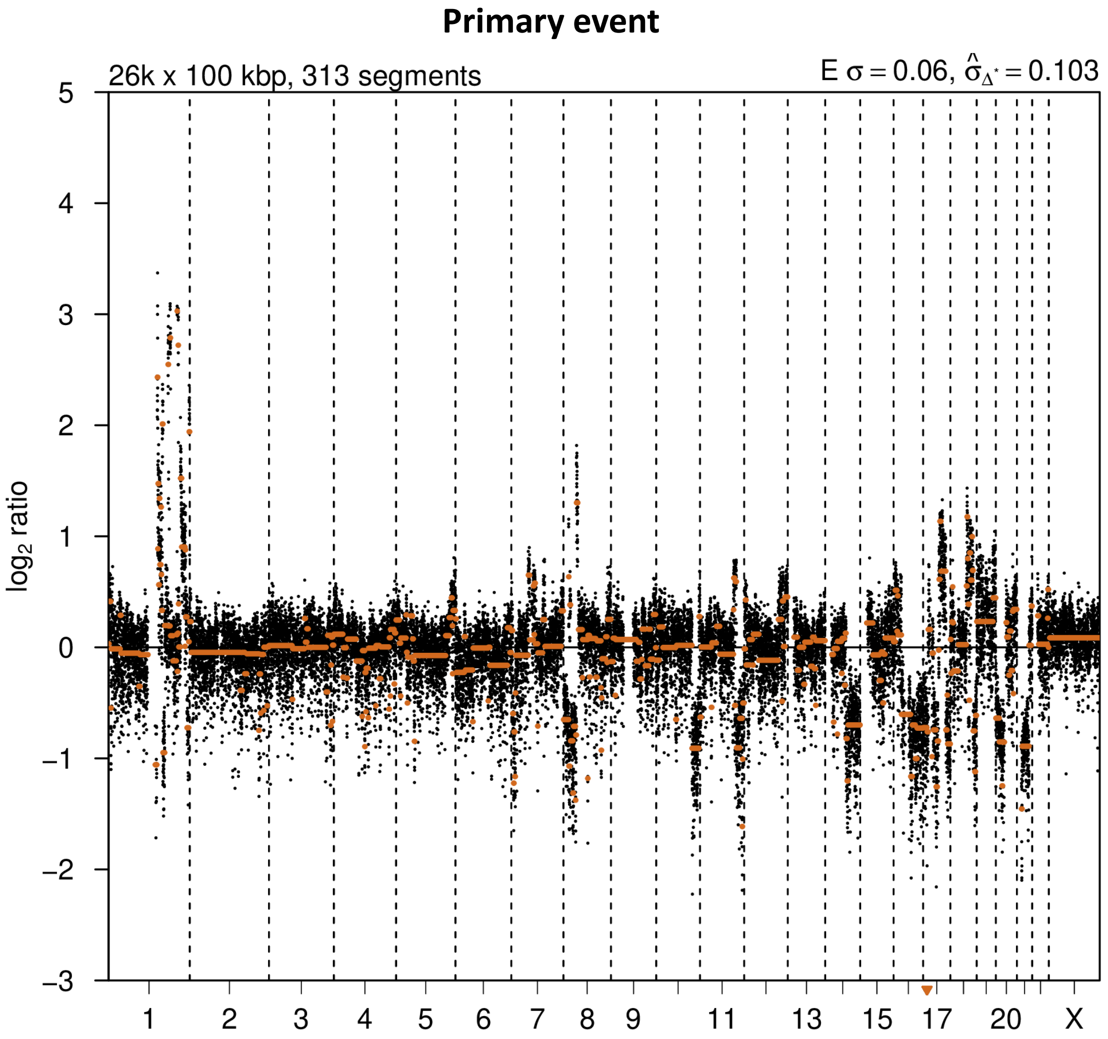

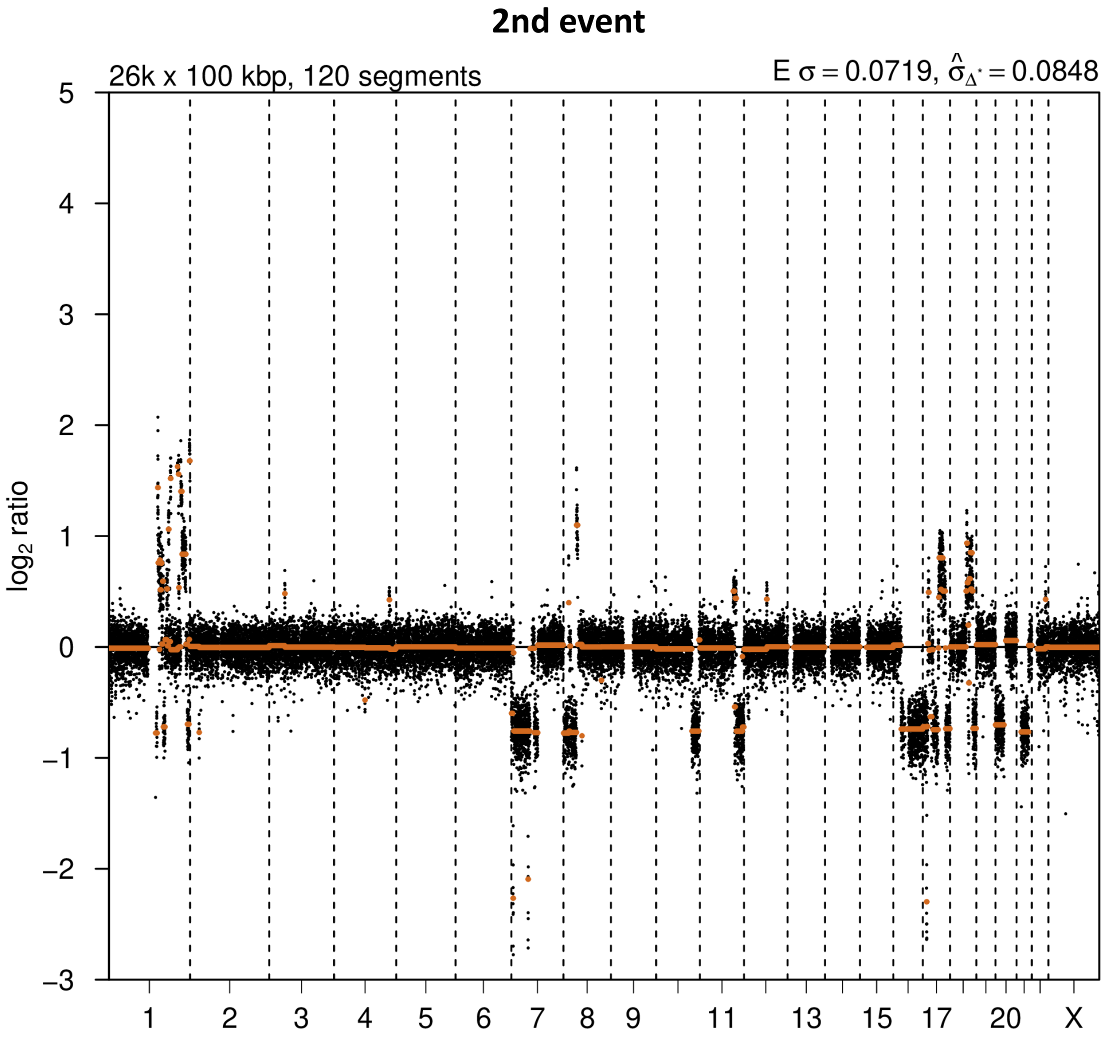

P055

Syn/Meta	Time from 1st surgery to 2nd event (Months)	Side 2nd event	Histology 2nd event	Surgery	AdjuvanT Treatment Pri (RT/ HT)	ER Pri	ER 2nd event	Her2 Pri	Her2 2nd event	Grade Pri	Grade 2nd event	Quadrant 2nd event	Margins	Screening	Clonality P value Copy N	Clonality P value Panel seq	Clonality P value WES	Final verdict
metachronous	65	Ipsilateral	IDC no DCIS	lumpectomy	None	+	+	-	-	2	2	at or adjacent to primary	Clear	symptomatic	0.06134372	0.003	NA	Related

Primary event

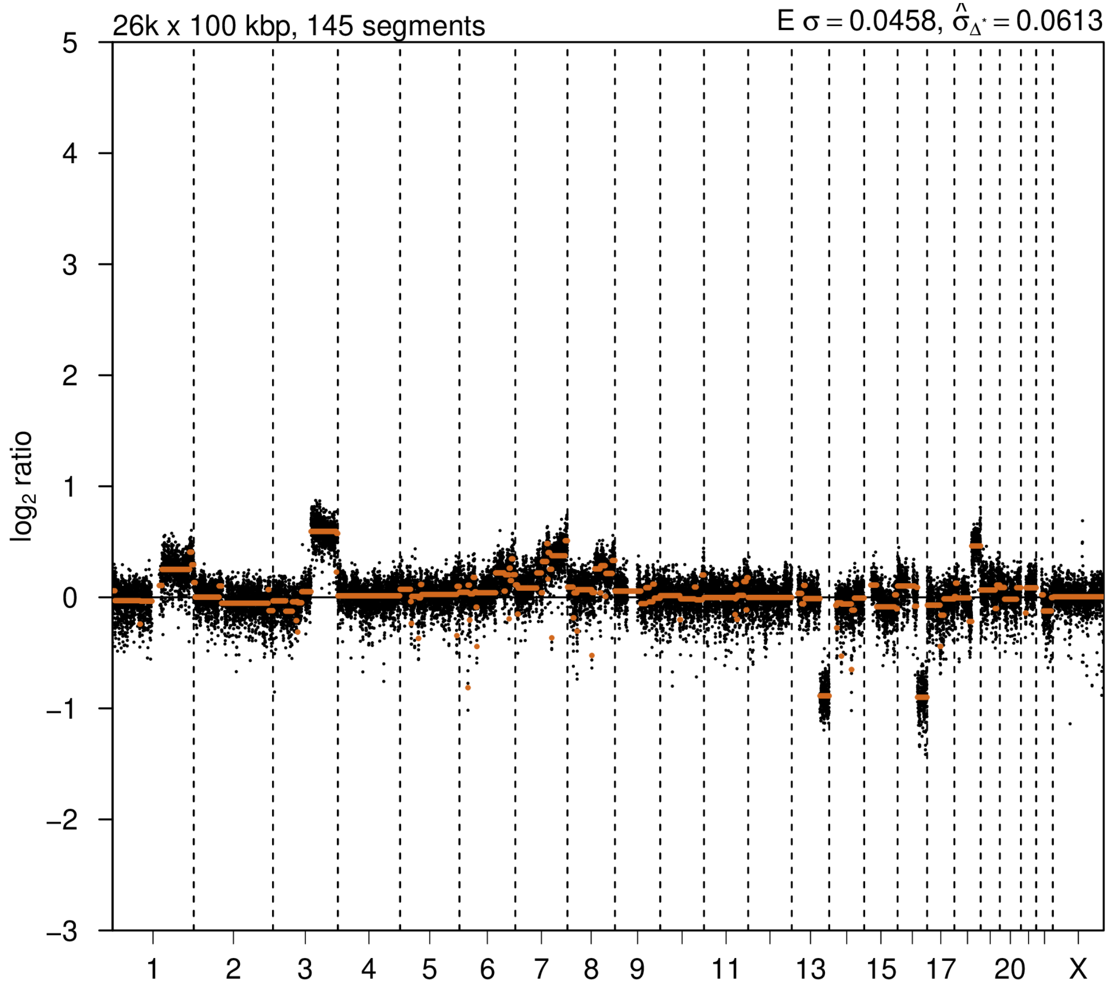

2nd event

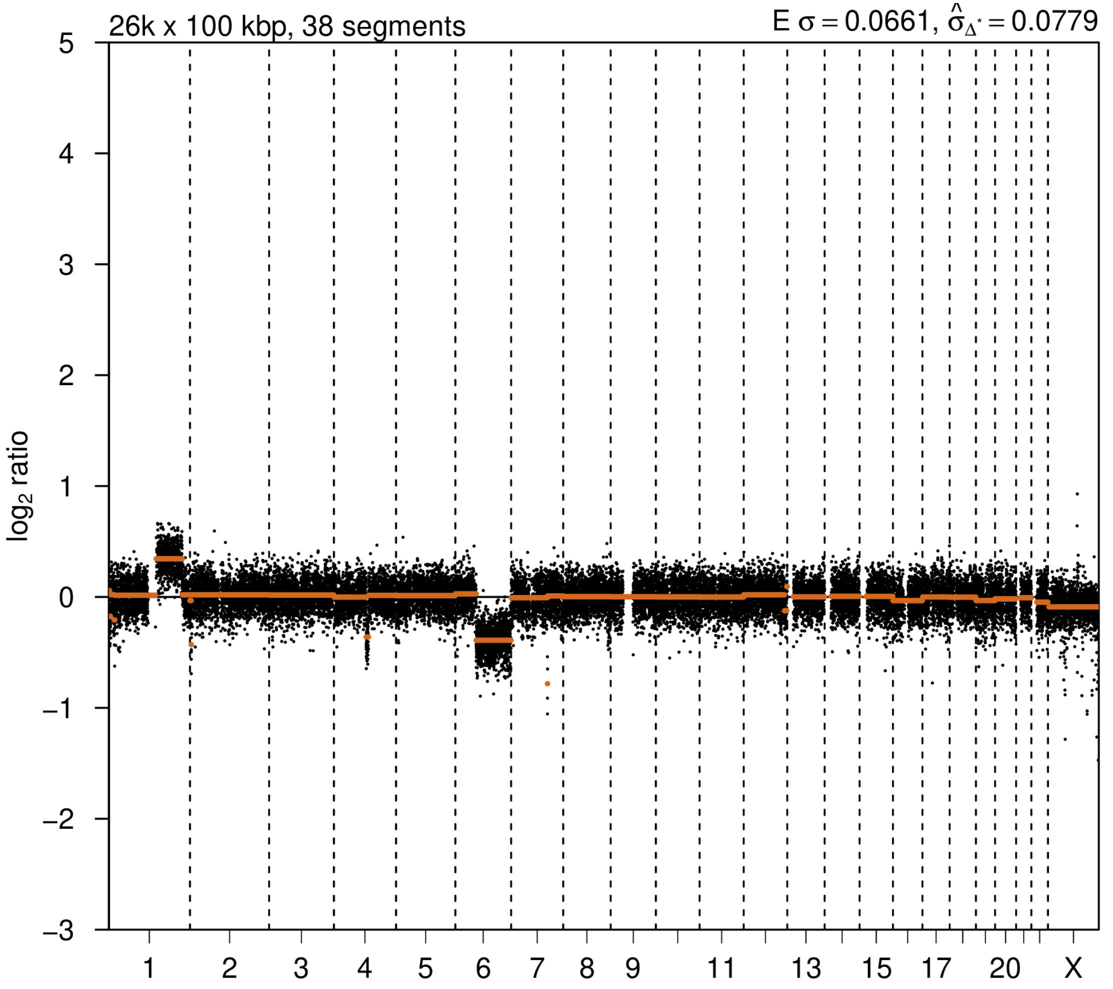

P056

Syn/Meta	Time from 1st surgery to 2nd event (Months)	Side 2nd event	Histology 2nd event	Surgery	AdjuvanT Treatment Pri (RT/ HT)	ER Pri	ER 2nd event	Her2 Pri	Her2 2nd event	Grade Pri	Grade 2nd event	Quadrant 2nd event	Margins	Screening	Clonality P value Copy N	Clonality P value Panel seq	Clonality P value WES	Final verdict
metachronous	77	Ipsilateral	IDC with DCIS	lumpectomy	None	-	+	-	-	2	3	at or adjacent to primary	NA	symptomatic	0.00064914	Single mutation - shared	NA	Related

Primary event

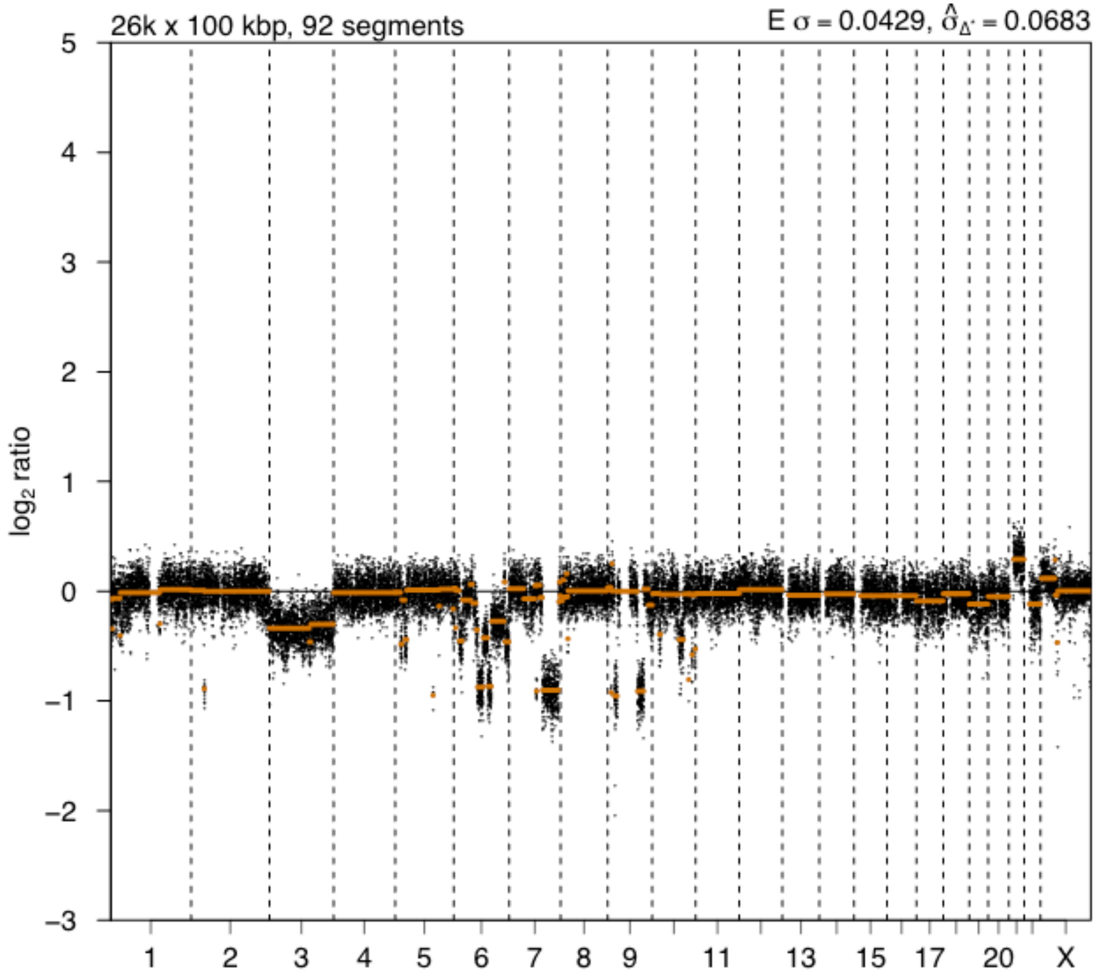

2nd event

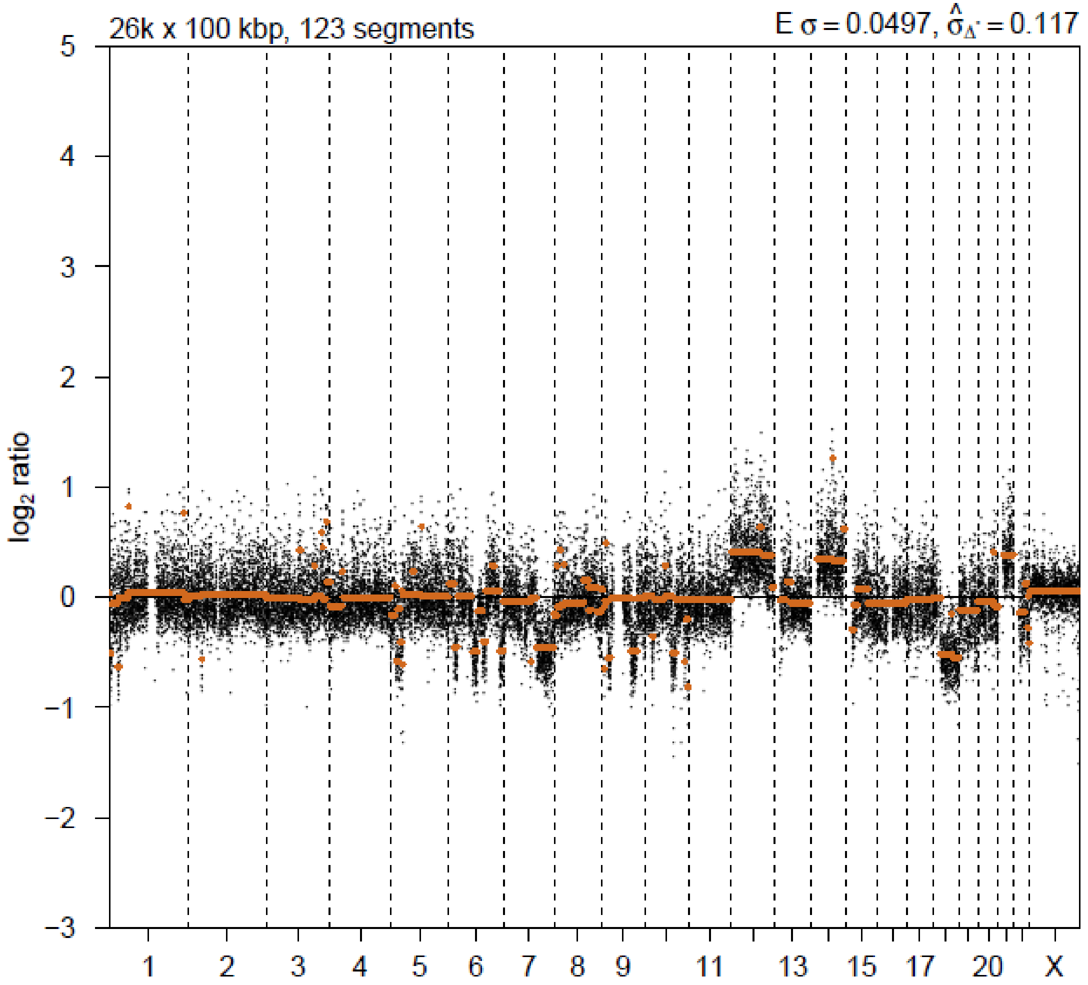

P057

Syn/Meta	Time from 1st surgery to 2nd event (Months)	Side 2nd event	Histology 2nd event	Surgery	AdjuvanT Treatment Pri (RT/ HT)	ER Pri	ER 2nd event	Her2 Pri	Her2 2nd event	Grade Pri	Grade 2nd event	Quadrant 2nd event	Margins	Screening	Clonality P value Copy N	Clonality P value Panel seq	Clonality P value WES	Final verdict
metachronous	76	Ipsilateral	IDC with DCIS	lumpectomy	None	+	+	+	+	3	3	at or adjacent to primary	involved	screen-detected	0.000324 57	Single mutation - shared	NA	Related

Primary event

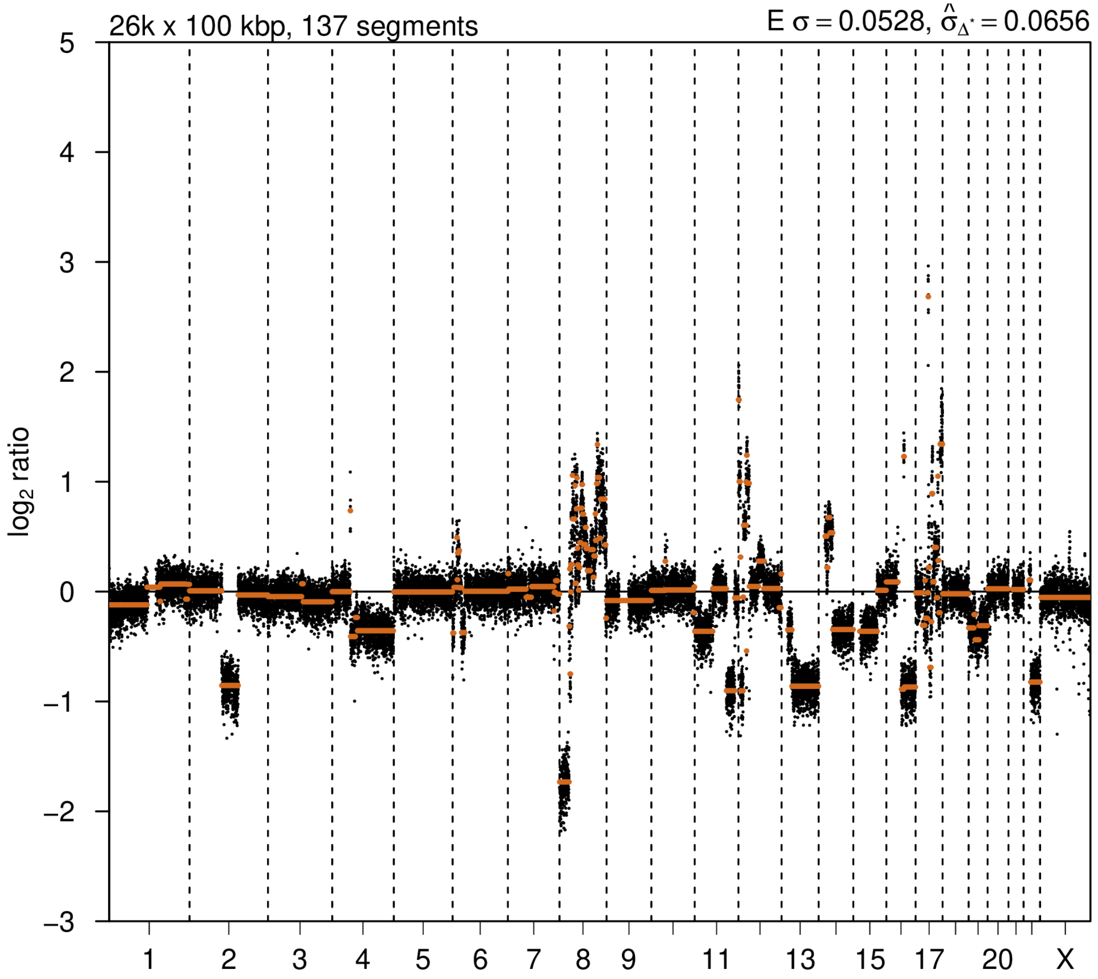

2nd event

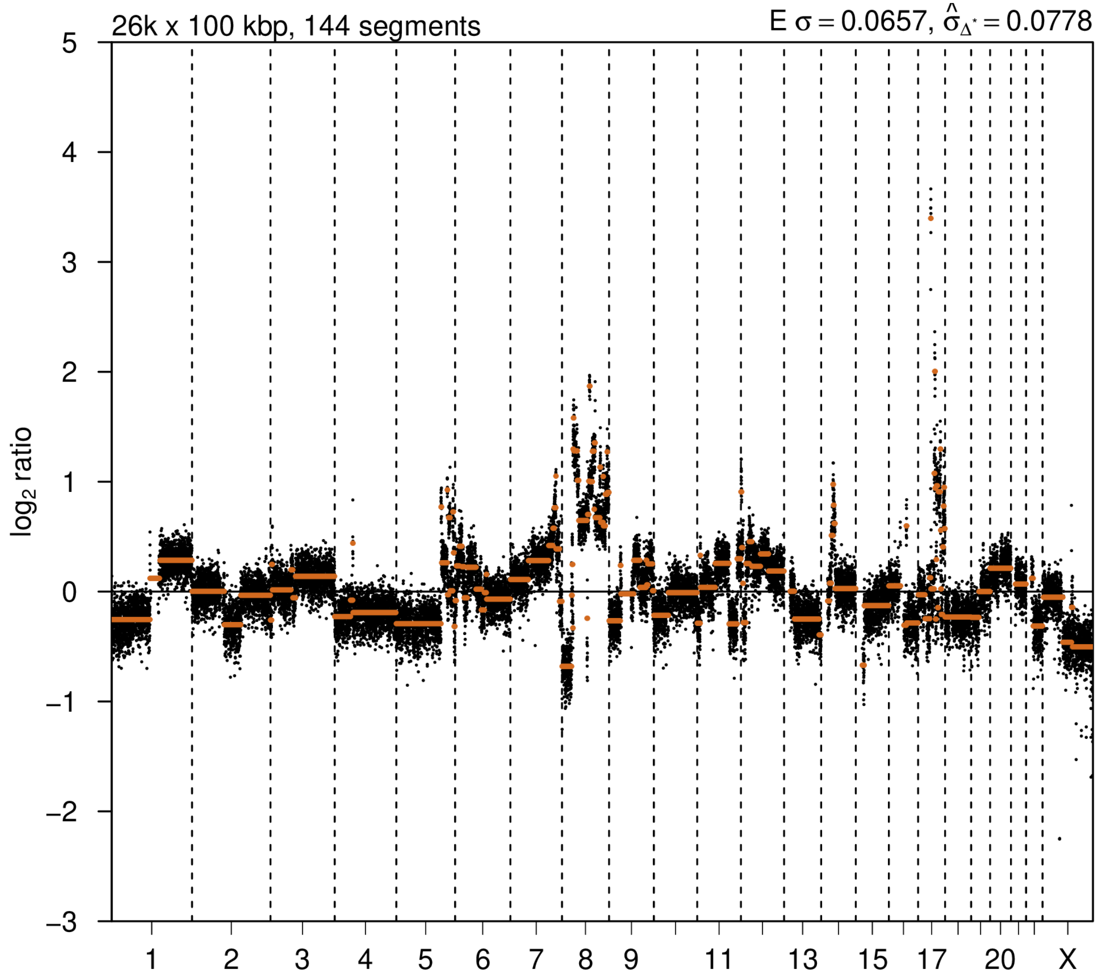

P059

Syn/Meta	Time from 1st surgery to 2nd event (Months)	Side 2nd event	Histology 2nd event	Surgery	AdjuvanT Treatment Pri (RT/ HT)	ER Pri	ER 2nd event	Her2 Pri	Her2 2nd event	Grade Pri	Grade 2nd event	Quadrant 2nd event	Margins	Screening	Clonality P value Copy N	Clonality P value Panel seq	Clonality P value WES	Final verdict
metachronous	83	Ipsilateral	IDC no DCIS	lumpectomy	None	+	+	-	+	3	3	at or adjacent to primary	Clear	screen-detected	0.000649 14	NA	NA	Related

Primary event

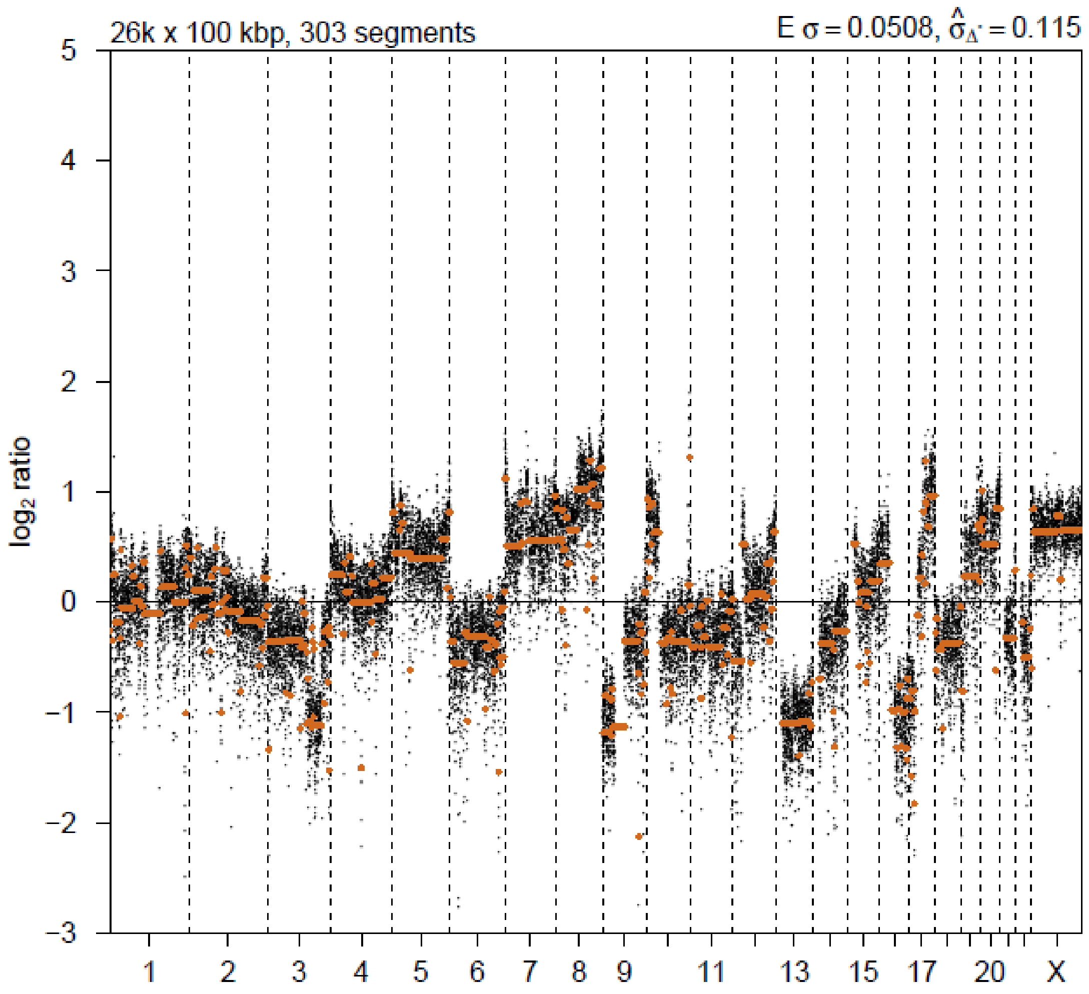

2nd event

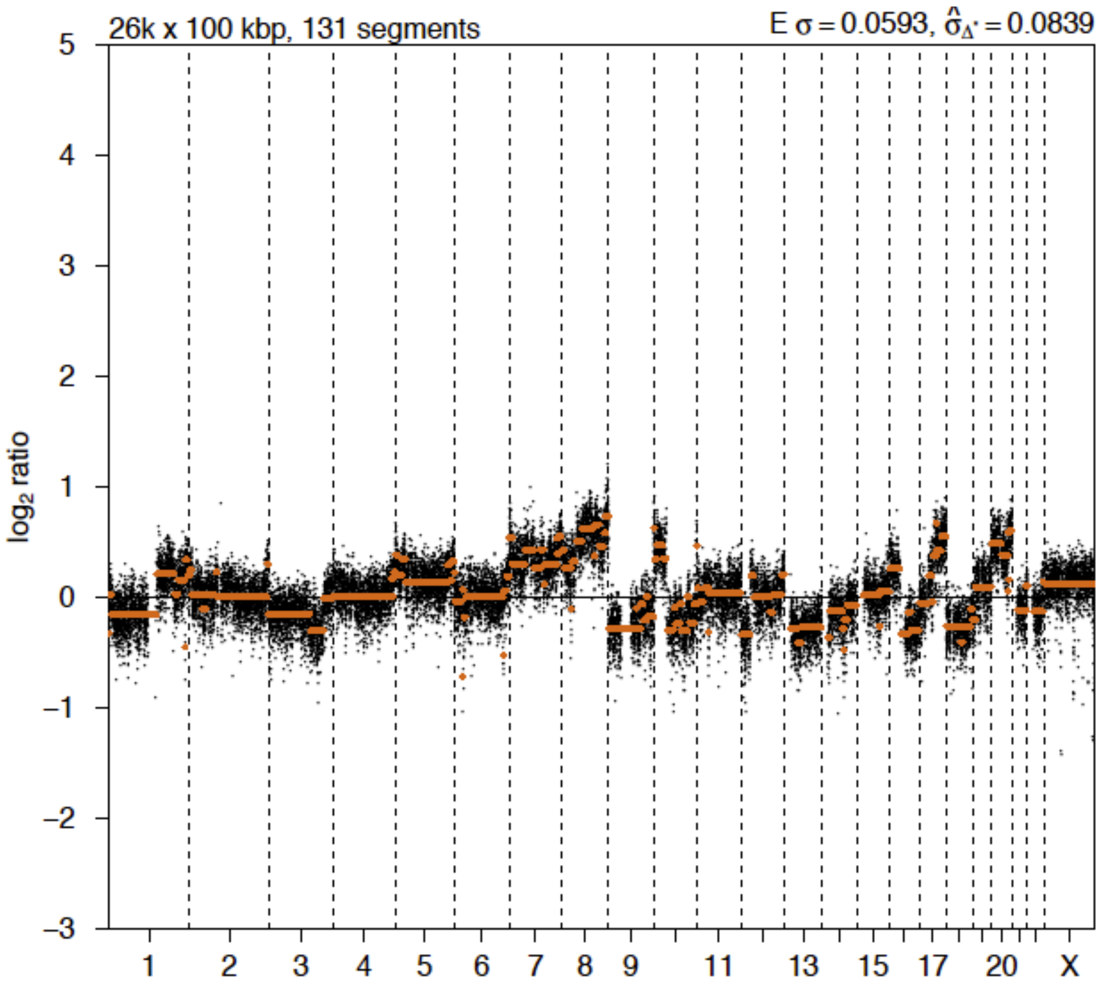

P061

Syn/Meta	Time from 1st surgery to 2nd event (Months)	Side 2nd event	Histology 2nd event	Surgery	AdjuvanT Treatment Pri (RT/ HT)	ER Pri	ER 2nd event	Her2 Pri	Her2 2nd event	Grade Pri	Grade 2nd event	Quadrant 2nd event	Margins	Screening	Clonality P value Copy N	Clonality P value Panel seq	Clonality P value WES	Final verdict
metachronous	41	Ipsilateral	IDC with DCIS	lumpectomy	None	+	+	-	-	2	2	NA	Clear	symptomatic	0.00032457	0.003	NA	Related

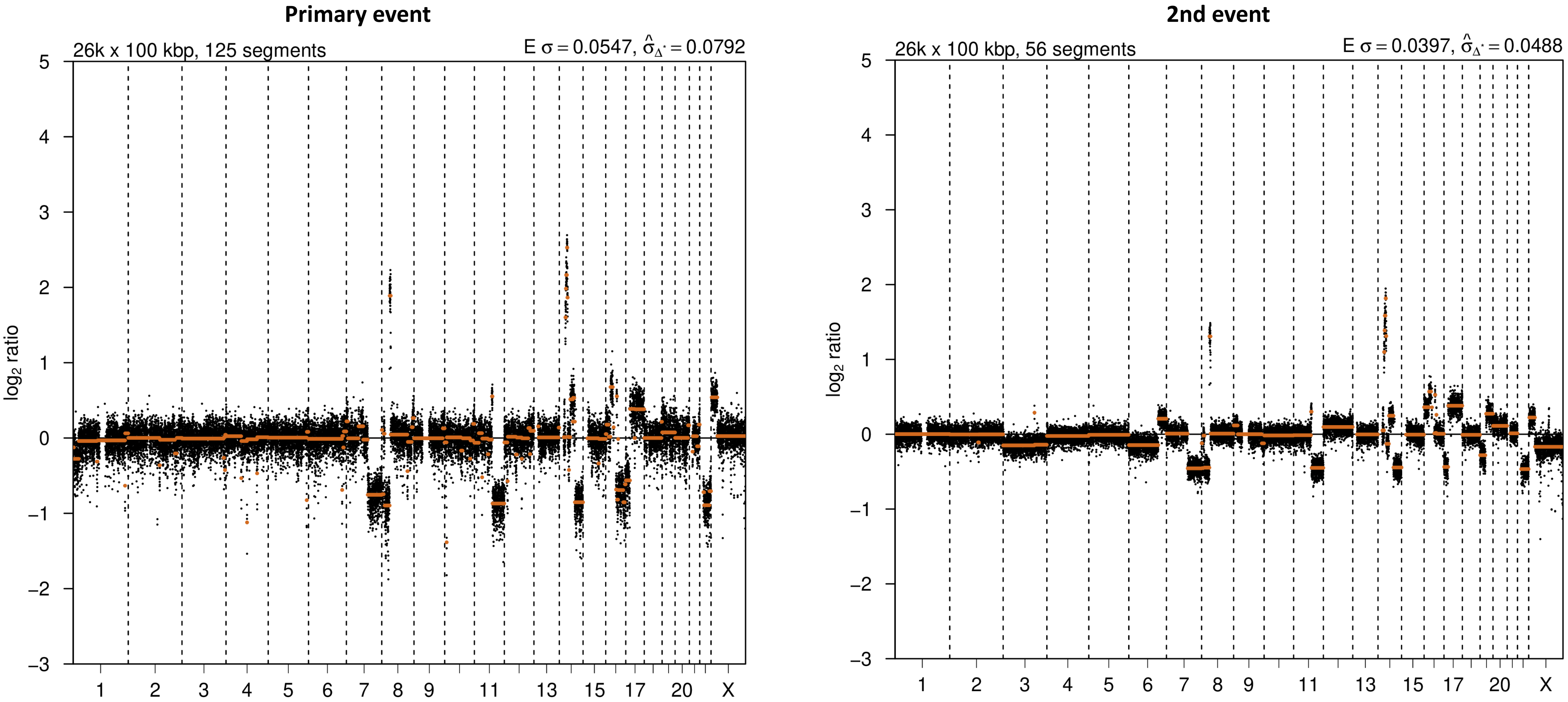

P063

Syn/Meta	Time from 1st surgery to 2nd event (Months)	Side 2nd event	Histology 2nd event	Surgery	AdjuvanT Treatment Pri (RT/ HT)	ER Pri	ER 2nd event	Her2 Pri	Her2 2nd event	Grade Pri	Grade 2nd event	Quadrant 2nd event	Margins	Screening	Clonality P value Copy N	Clonality P value Panel seq	Clonality P value WES	Final verdict
metachronous	37	Ipsilateral	IDC with DCIS	lumpectomy	None	+	+	-	-	3	1	NA	involved	screen-detected	0.000324 57	NA	NA	Related

Primary event

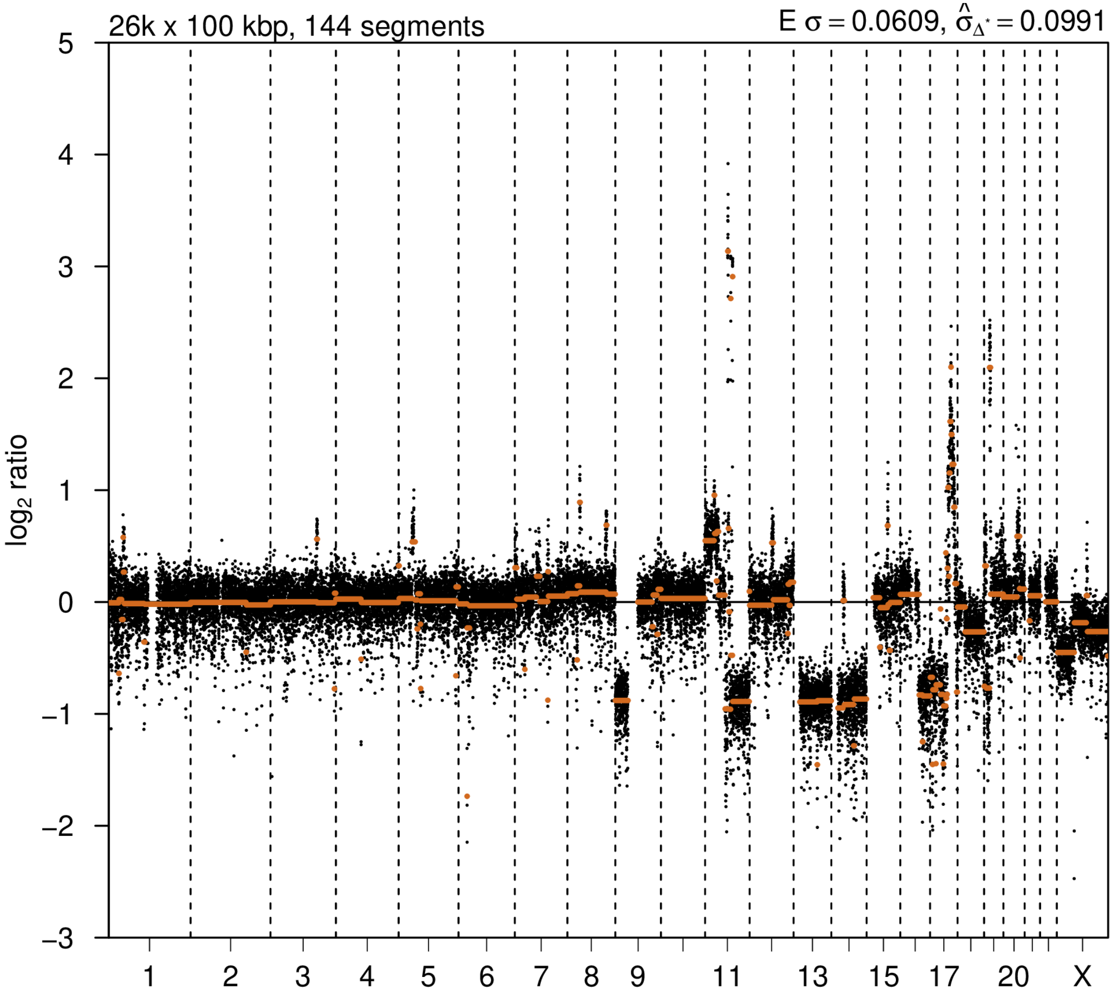

2nd event

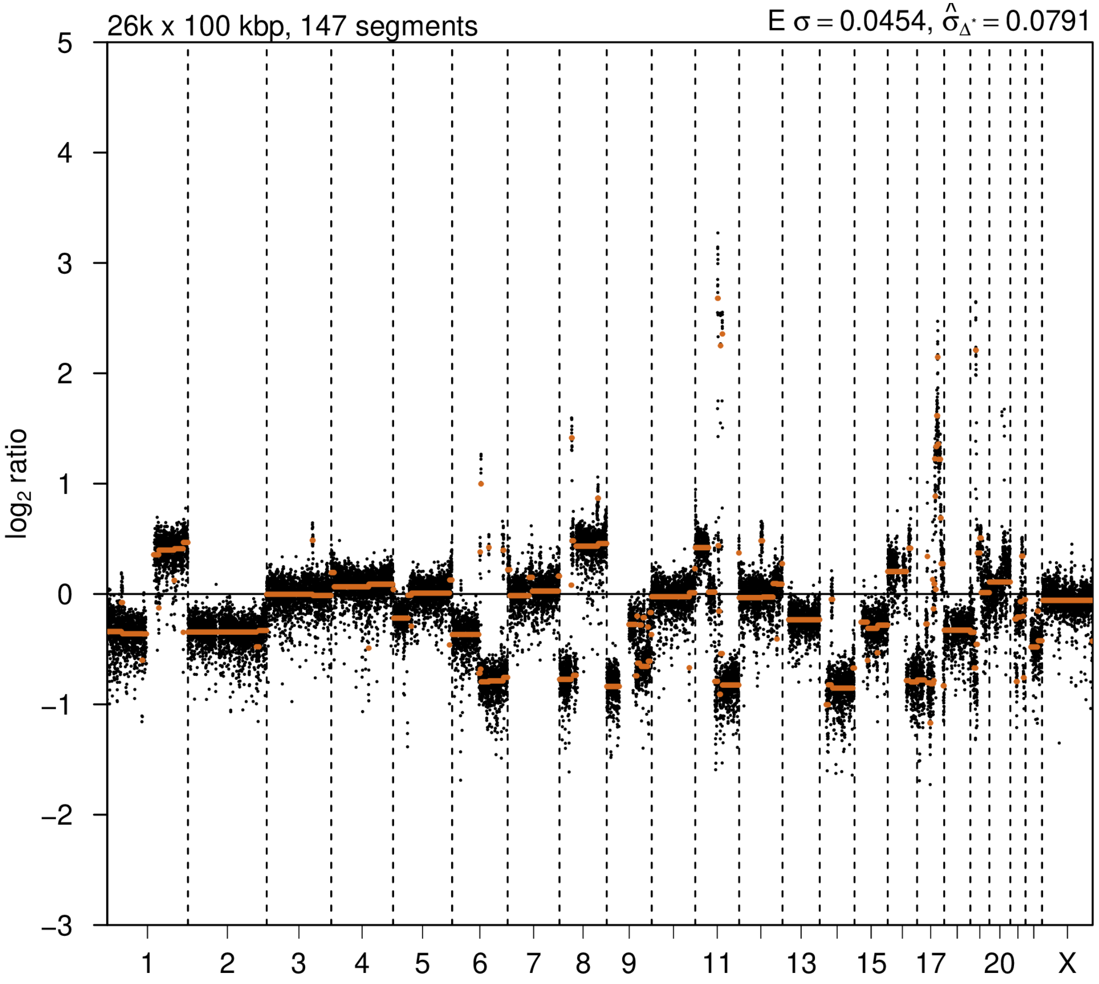

P065

Syn/Meta	Time from 1st surgery to 2nd event (Months)	Side 2nd event	Histology 2nd event	Surgery	AdjuvanT Treatment Pri (RT/ HT)	ER Pri	ER 2nd event	Her2 Pri	Her2 2nd event	Grade Pri	Grade 2nd event	Quadrant 2nd event	Margins	Screening	Clonality P value Copy N	Clonality P value Panel seq	Clonality P value WES	Final verdict
metachronous	44	Ipsilateral	IDC with DCIS	lumpectomy	None	-	-	+	+	2	1	at or adjacent to primary	involved	screen-detected	0.000324 57	NA	NA	Related

Primary event

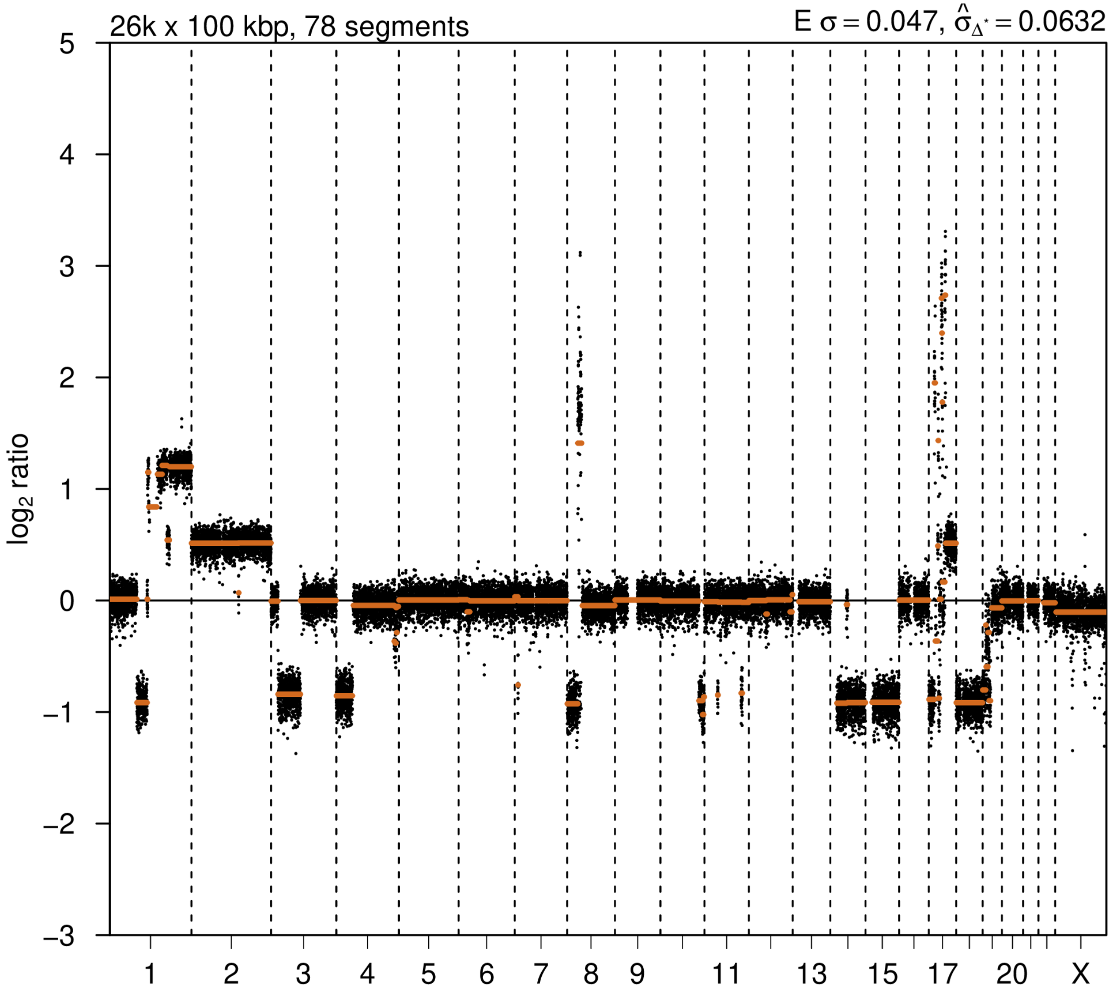

2nd event

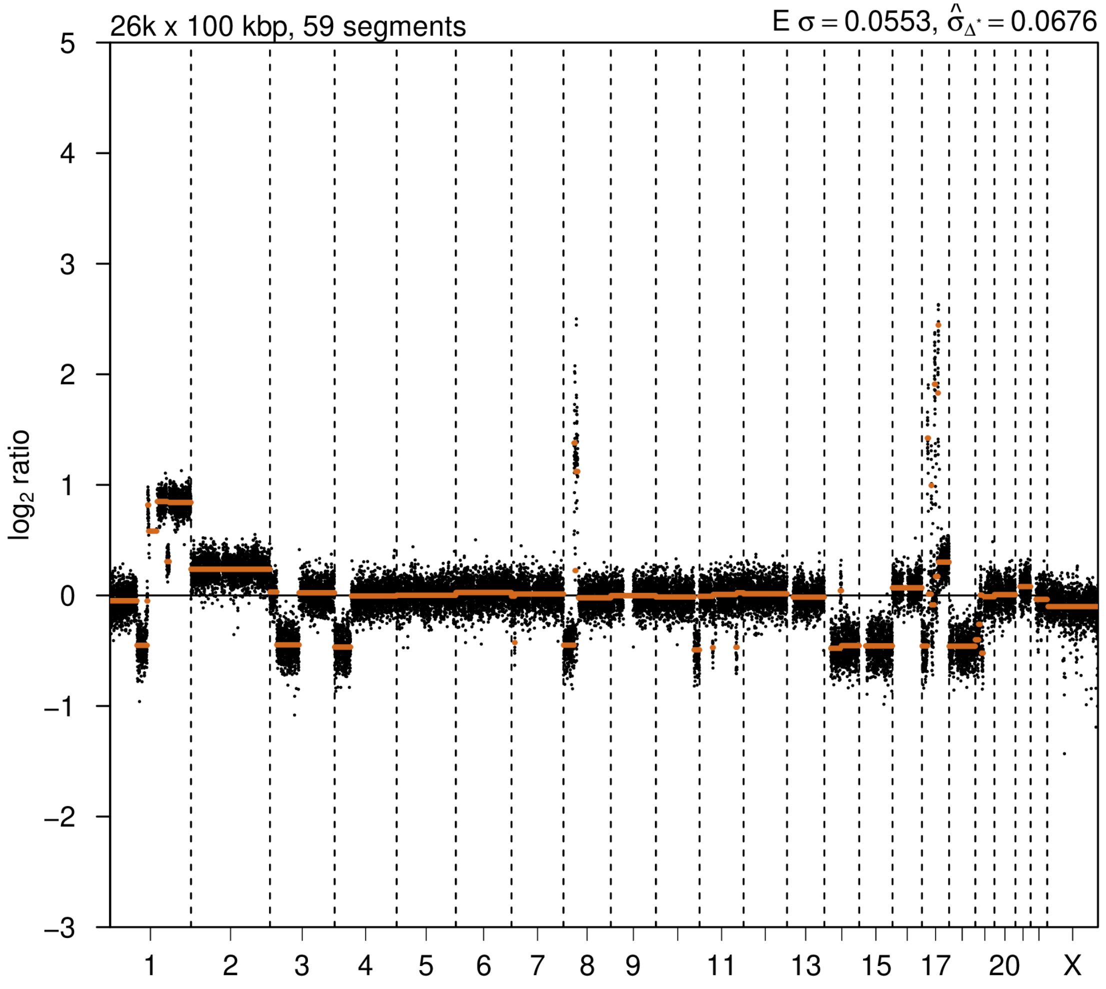

P066

Syn/Meta	Time from 1st surgery to 2nd event (Months)	Side 2nd event	Histology 2nd event	Surgery	AdjuvanT Treatment Pri (RT/ HT)	ER Pri	ER 2nd event	Her2 Pri	Her2 2nd event	Grade Pri	Grade 2nd event	Quadrant 2nd event	Margins	Screening	Clonality P value Copy N	Clonality P value Panel seq	Clonality P value WES	Final verdict
metachronous	50	Ipsilateral	IDC with DCIS	lumpectomy	None	+	+	-	-	1	2	at or adjacent to primary	Clear	screen-detected	0.000324 57	Single mutation - shared	NA	Related

Primary event

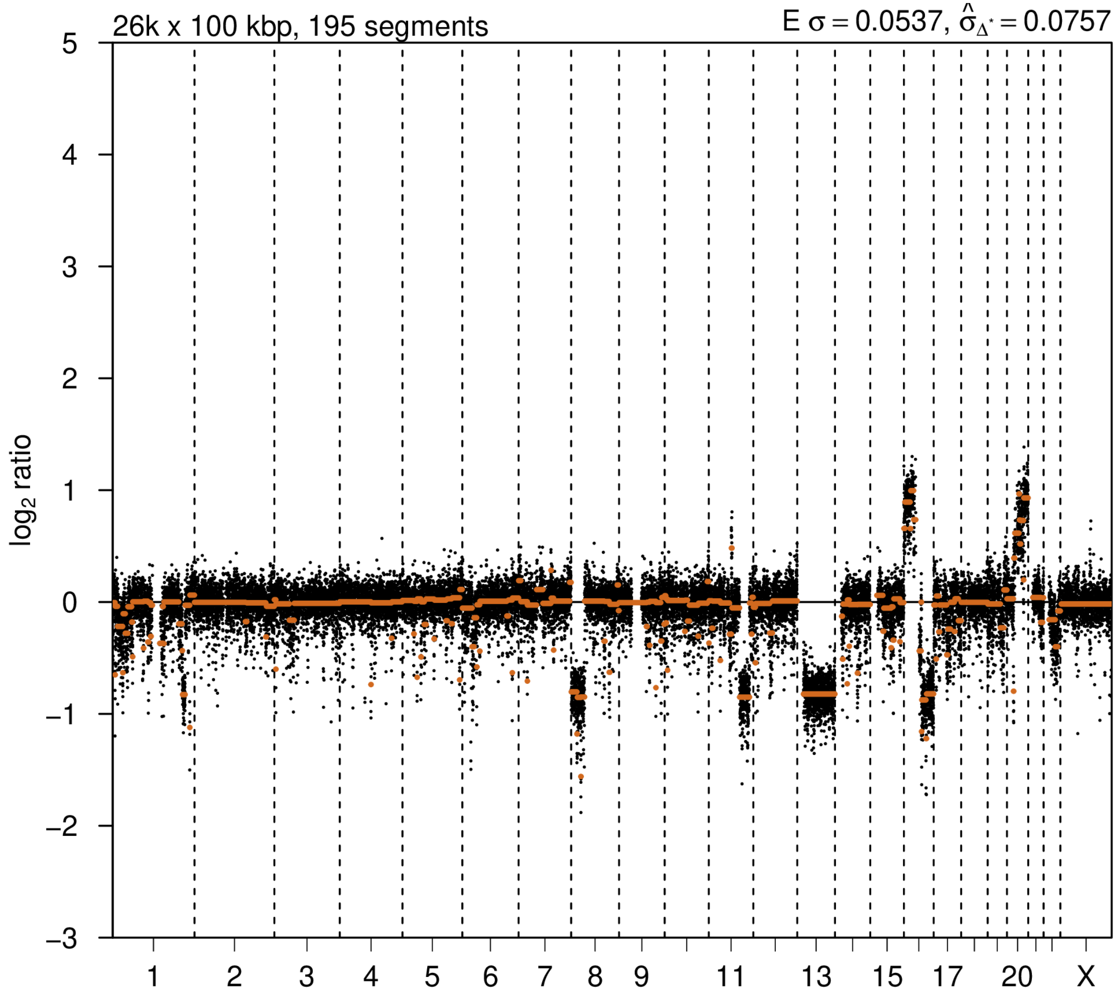

2nd event

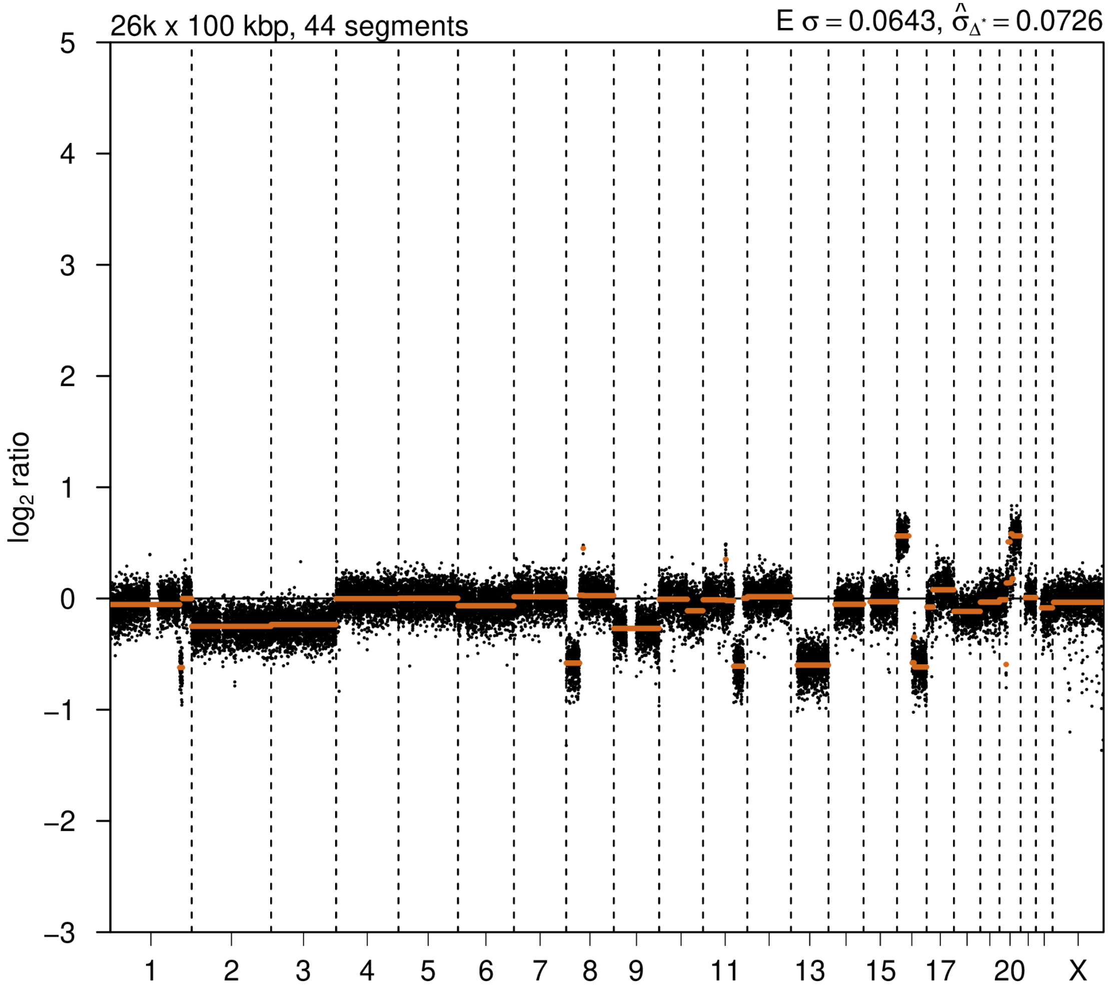

P067

Syn/Meta	Time from 1st surgery to 2nd event (Months)	Side 2nd event	Histology 2nd event	Surgery	AdjuvanT Treatment Pri (RT/ HT)	ER Pri	ER 2nd event	Her2 Pri	Her2 2nd event	Grade Pri	Grade 2nd event	Quadrant 2nd event	Margins	Screening	Clonality P value Copy N	Clonality P value Panel seq	Clonality P value WES	Final verdict
metachronous	33	Ipsilateral	IDC with DCIS	lumpectomy	None	+	+	-	-	2	2	at or adjacent to primary	NA	symptomatic	0.00032457	Single mutation - shared	NA	Related

Primary event

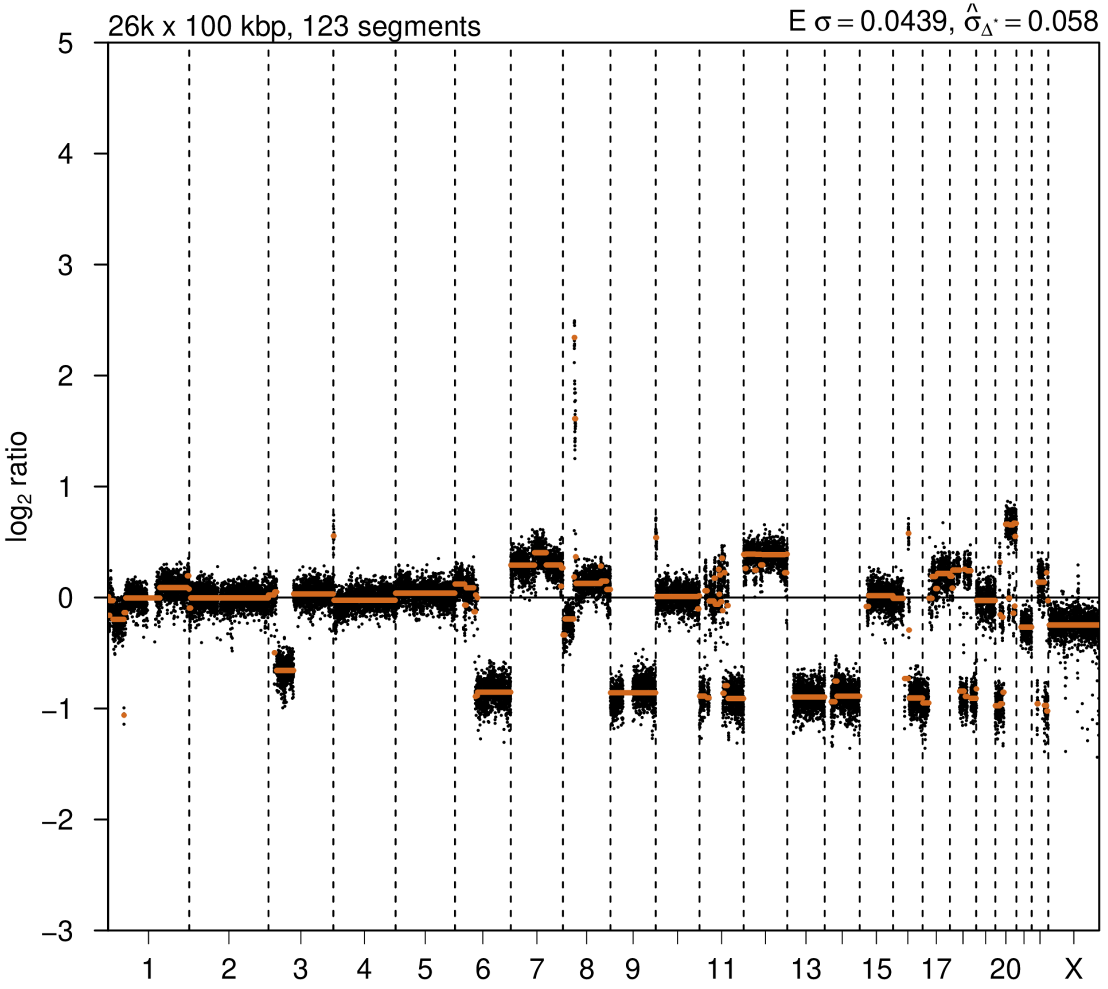

2nd event

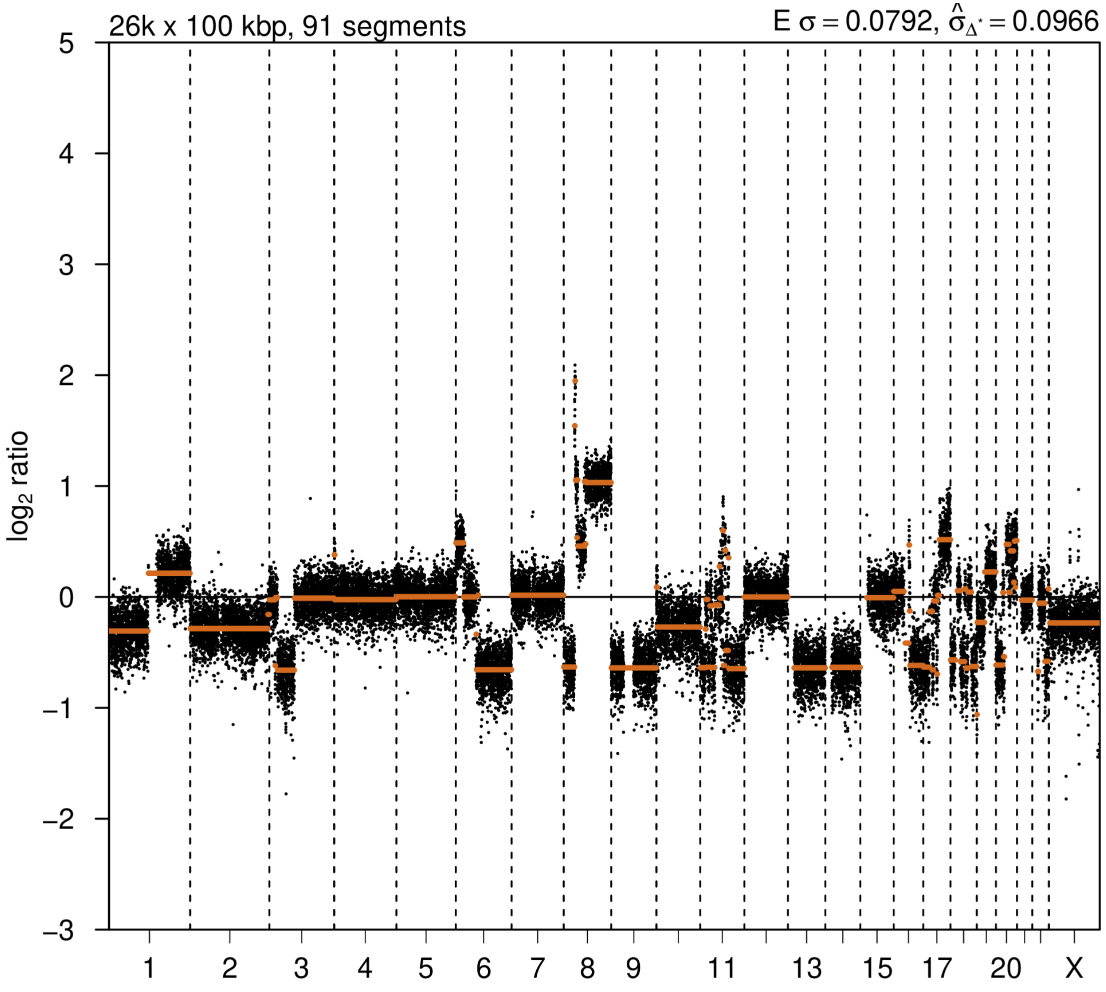

P068

Syn/Meta	Time from 1st surgery to 2nd event (Months)	Side 2nd event	Histology 2nd event	Surgery	AdjuvanT Treatment Pri (RT/ HT)	ER Pri	ER 2nd event	Her2 Pri	Her2 2nd event	Grade Pri	Grade 2nd event	Quadrant 2nd event	Margins	Screening	Clonality P value Copy N	Clonality P value Panel seq	Clonality P value WES	Final verdict
metachronous	50	Ipsilateral	IDC with DCIS	lumpectomy	None	+	+	-	-	2	2	at or adjacent to primary	Clear	screen-detected	0.000324 57	NA	0.00075244 5	Related

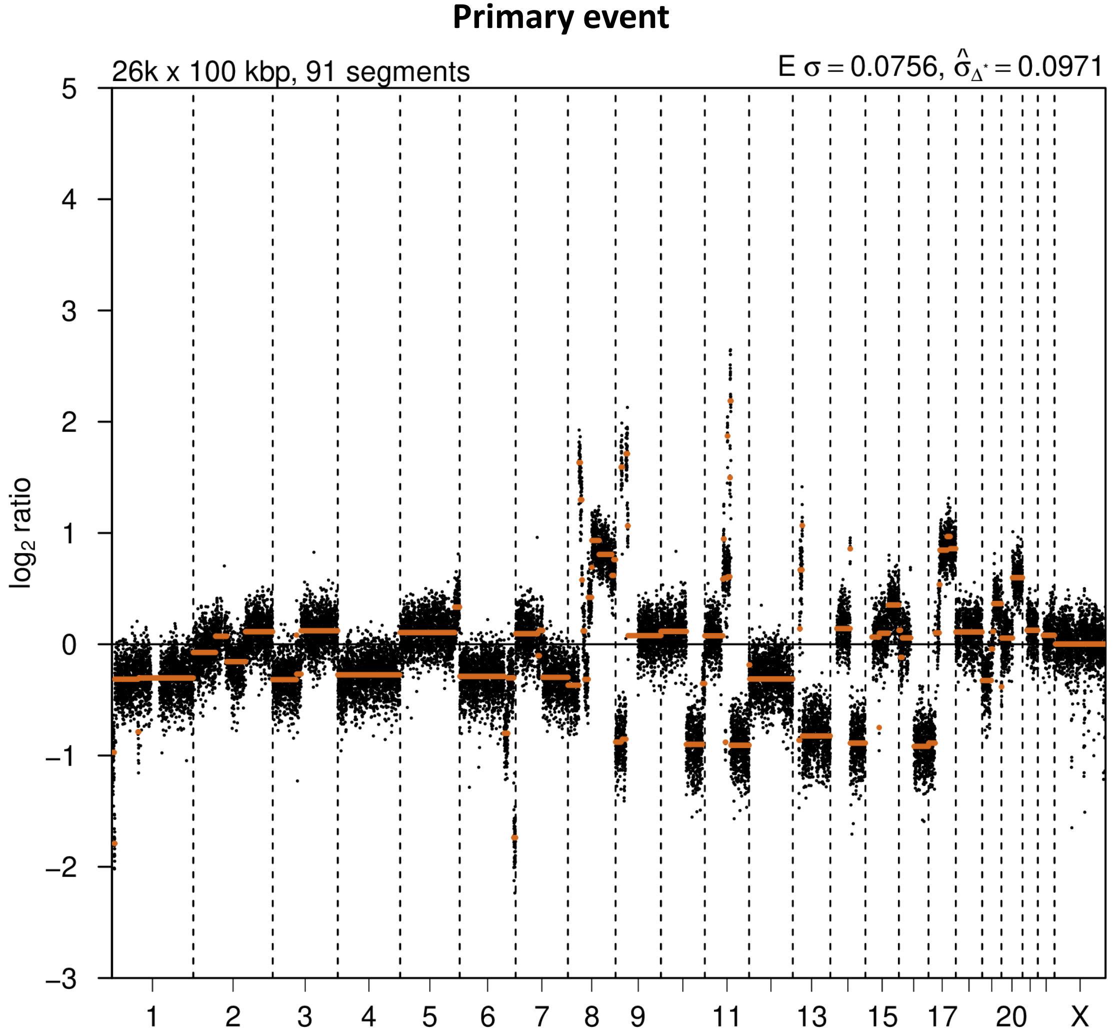

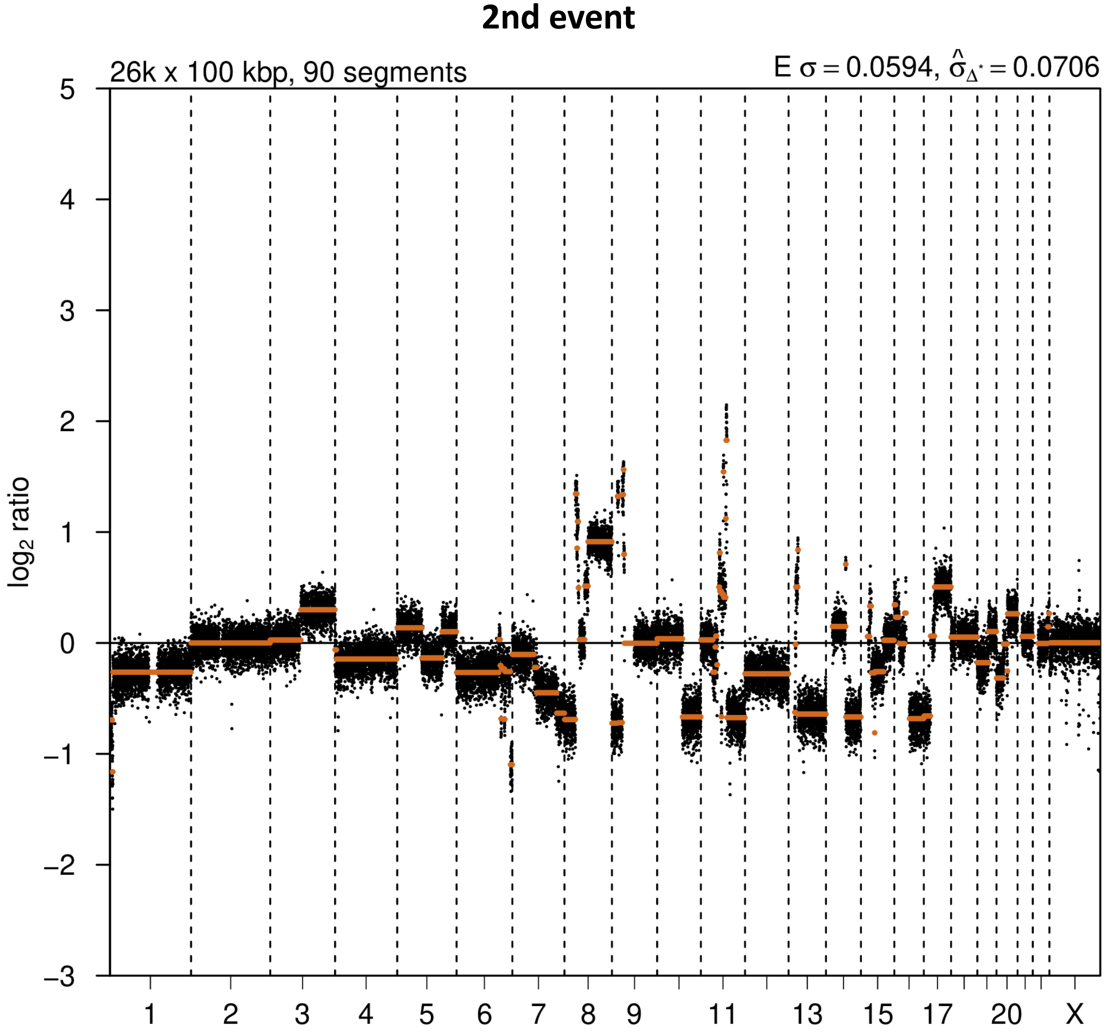

P069

Syn/Meta	Time from 1st surgery to 2nd event (Months)	Side 2nd event	Histology 2nd event	Surgery	AdjuvanT Treatment Pri (RT/ HT)	ER Pri	ER 2nd event	Her2 Pri	Her2 2nd event	Grade Pri	Grade 2nd event	Quadrant 2nd event	Margins	Screening	Clonality P value Copy N	Clonality P value Panel seq	Clonality P value WES	Final verdict
metachronous	35	Ipsilateral	IDC no DCIS	lumpectomy	None	+	+	+	+	3	3	at or adjacent to primary	Clear	symptomatic	0.00032457	0.003	NA	Related

Primary event

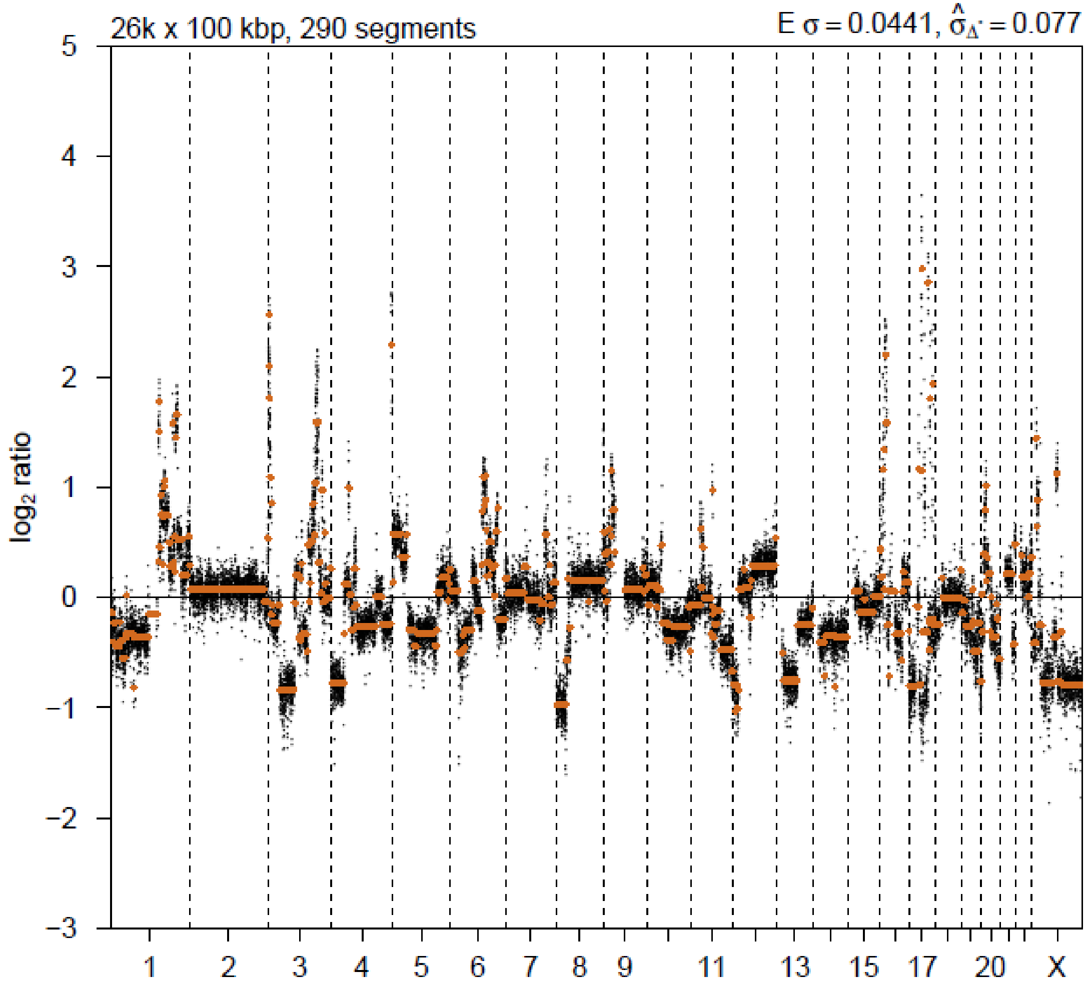

2nd event

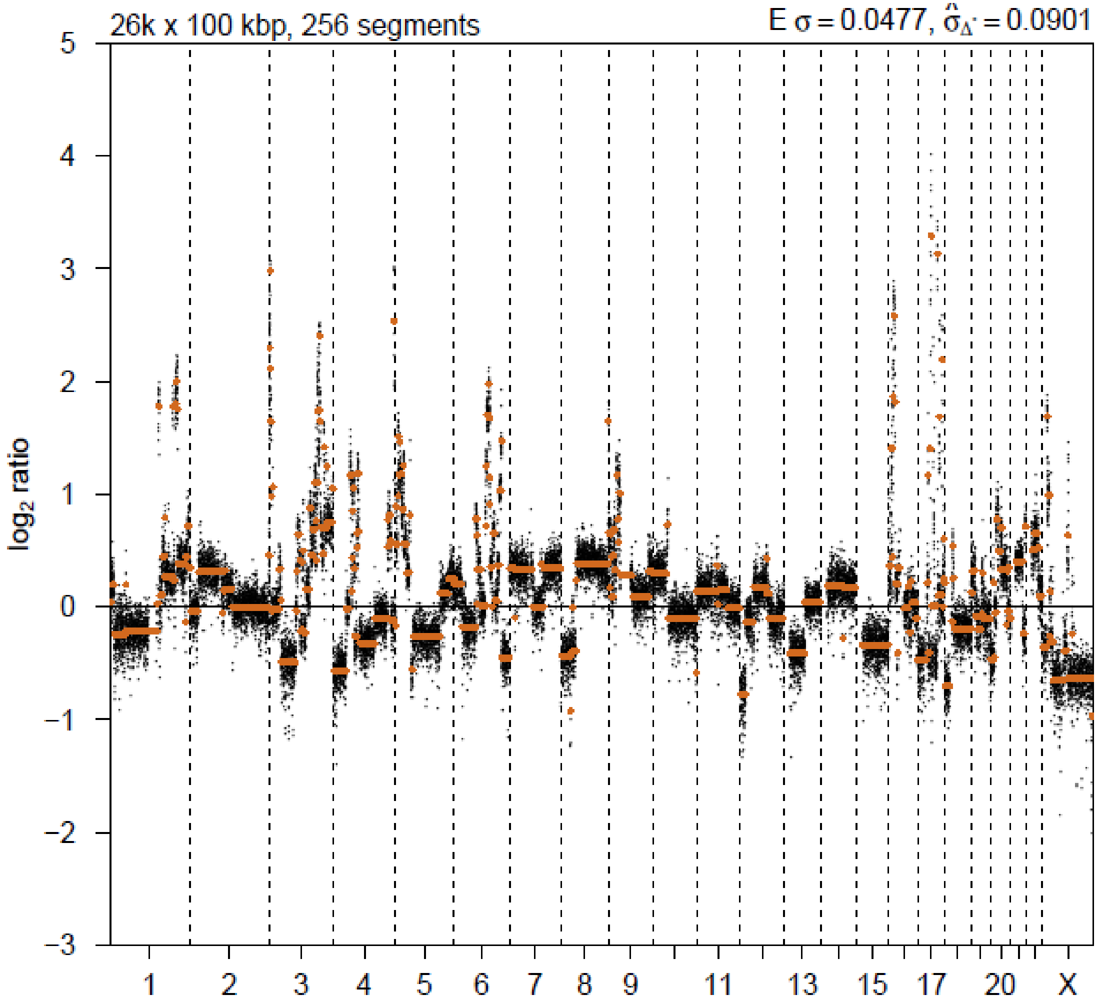

P072

Syn/Meta	Time from 1st surgery to 2nd event (Months)	Side 2nd event	Histology 2nd event	Surgery	AdjuvanT Treatment Pri (RT/ HT)	ER Pri	ER 2nd event	Her2 Pri	Her2 2nd event	Grade Pri	Grade 2nd event	Quadrant 2nd event	Margins	Screening	Clonality P value Copy N	Clonality P value Panel seq	Clonality P value WES	Final verdict
metachronous	55	Ipsilateral	IDC with DCIS	lumpectomy	None	+	+	+	+	3	3	at or adjacent to primary	NA	symptomatic	0.00292113	NA	NA	Related

Primary event

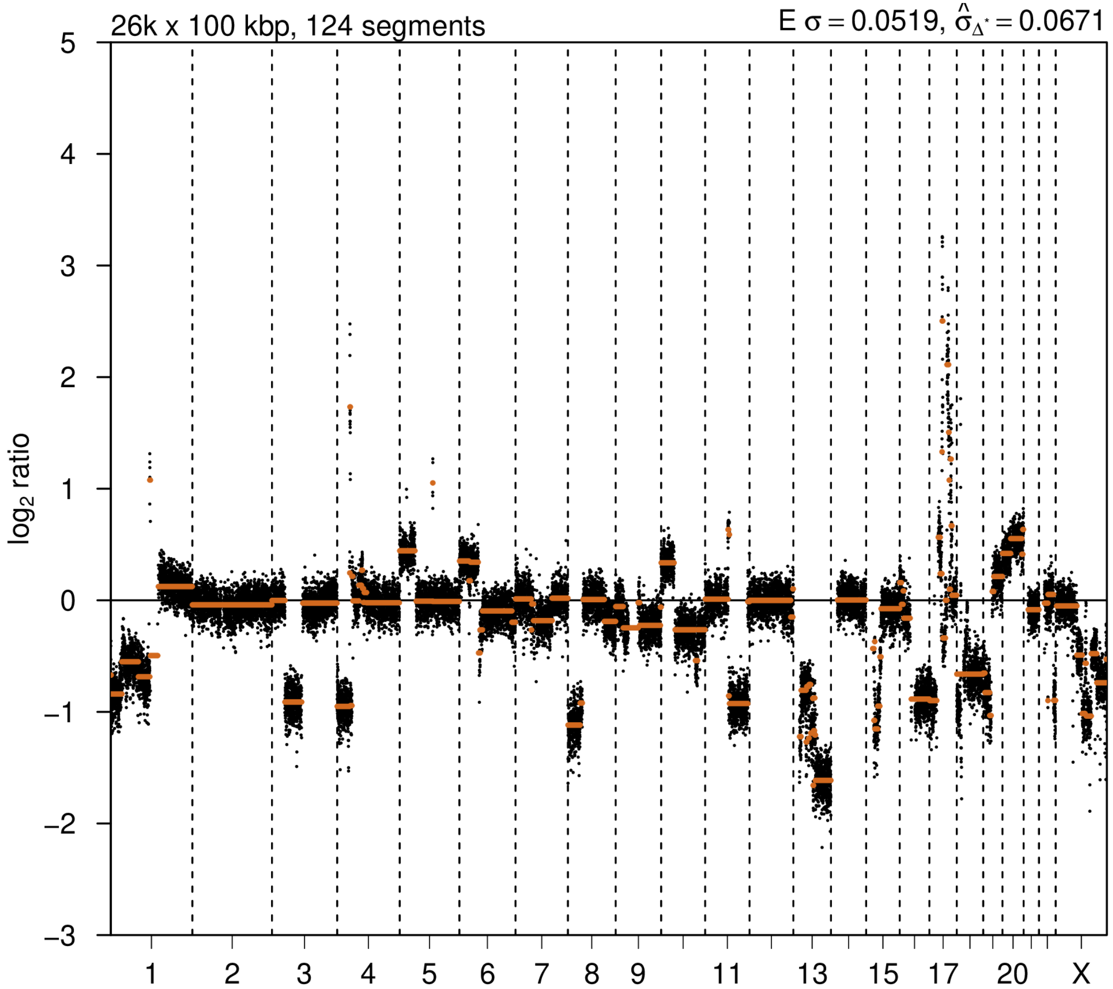

2nd event

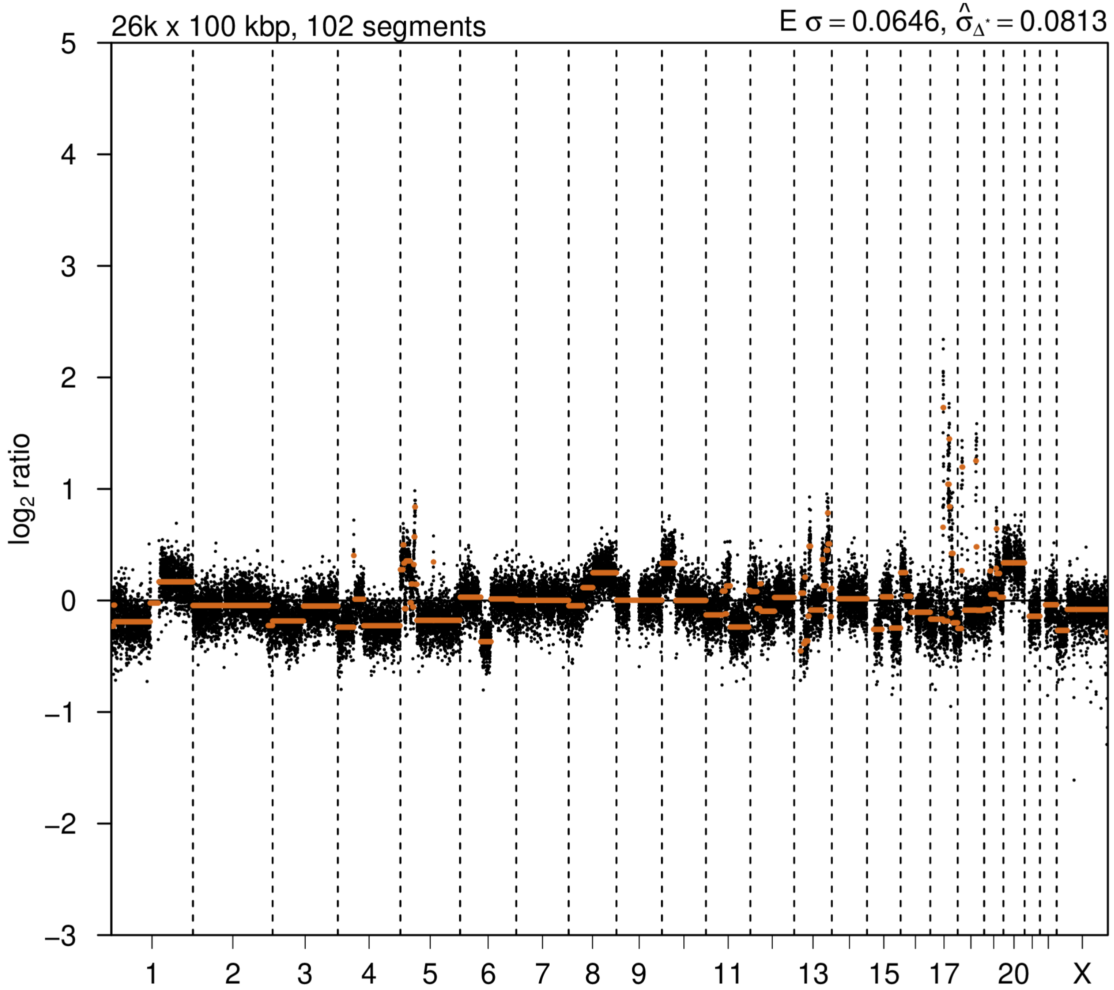

P073

Syn/Meta	Time from 1st surgery to 2nd event (Months)	Side 2nd event	Histology 2nd event	Surgery	AdjuvanT Treatment Pri (RT/ HT)	ER Pri	ER 2nd event	Her2 Pri	Her2 2nd event	Grade Pri	Grade 2nd event	Quadrant 2nd event	Margins	Screening	Clonality P value Copy N	Clonality P value Panel seq	Clonality P value WES	Final verdict
metachronous	36	Ipsilateral	IDC with DCIS	lumpectomy	None	-	-	+	+	3	3	at or adjacent to primary	Clear	screen-detected	0.000324 57	NA	NA	Related

Primary event

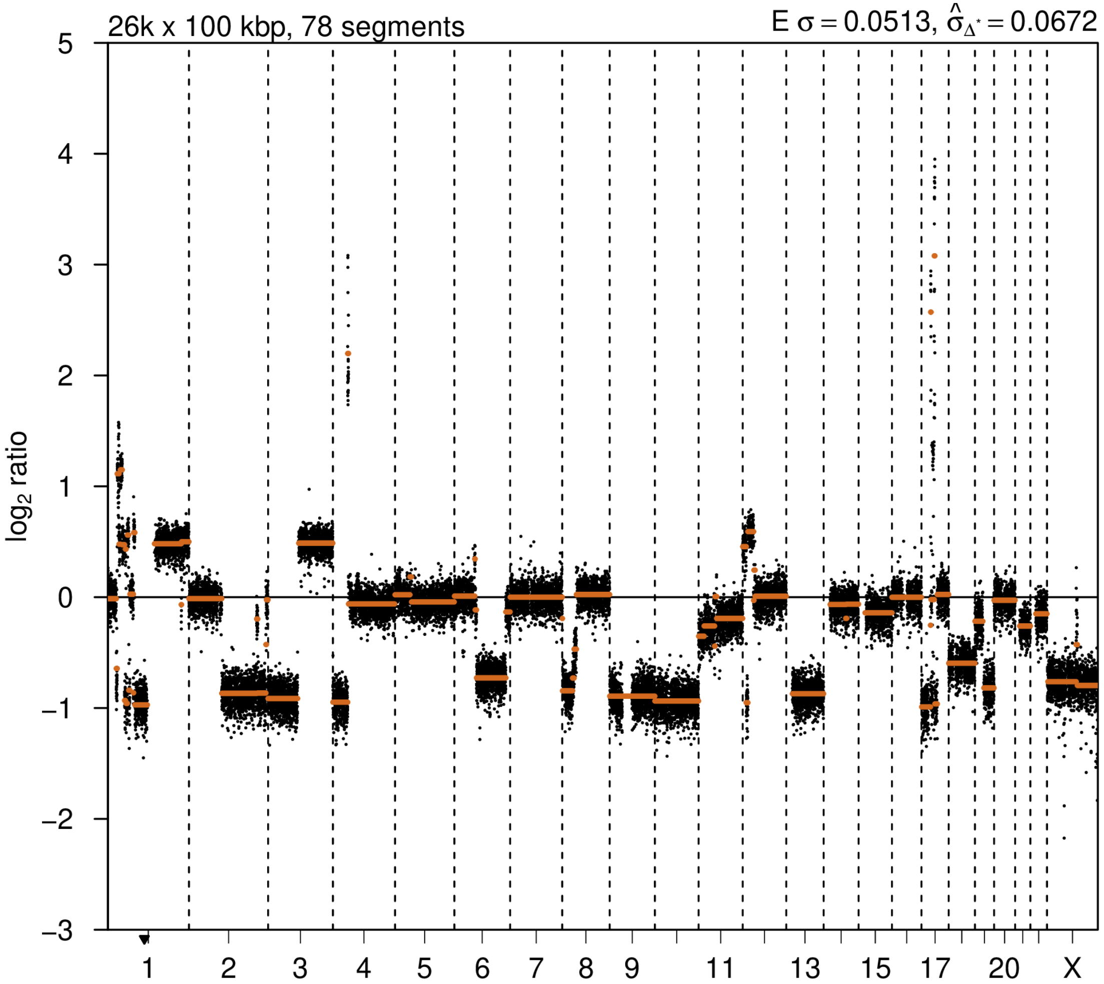

2nd event

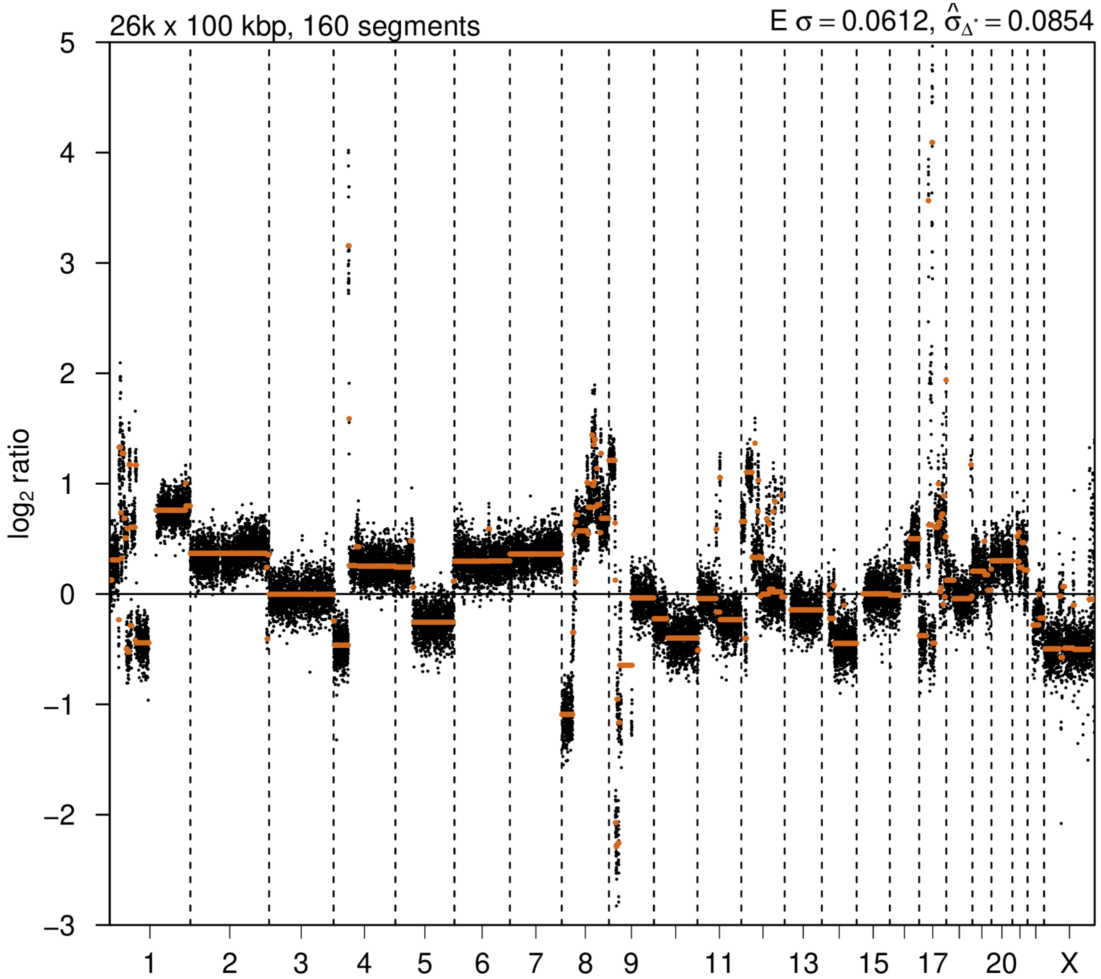

P082

Syn/Meta	Time from 1st surgery to 2nd event (Months)	Side 2nd event	Histology 2nd event	Surgery	AdjuvanT Treatment Pri (RT/ HT)	ER Pri	ER 2nd event	Her2 Pri	Her2 2nd event	Grade Pri	Grade 2nd event	Quadrant 2nd event	Margins	Screening	Clonality P value Copy N	Clonality P value Panel seq	Clonality P value WES	Final verdict
metachronous	11	Ipsilateral	IDC with DCIS	lumpectomy	None	+	+	+	-	2	2	distant from primary	involved	symptomatic	0.00032457	Single mutation - shared	NA	Related

Primary event

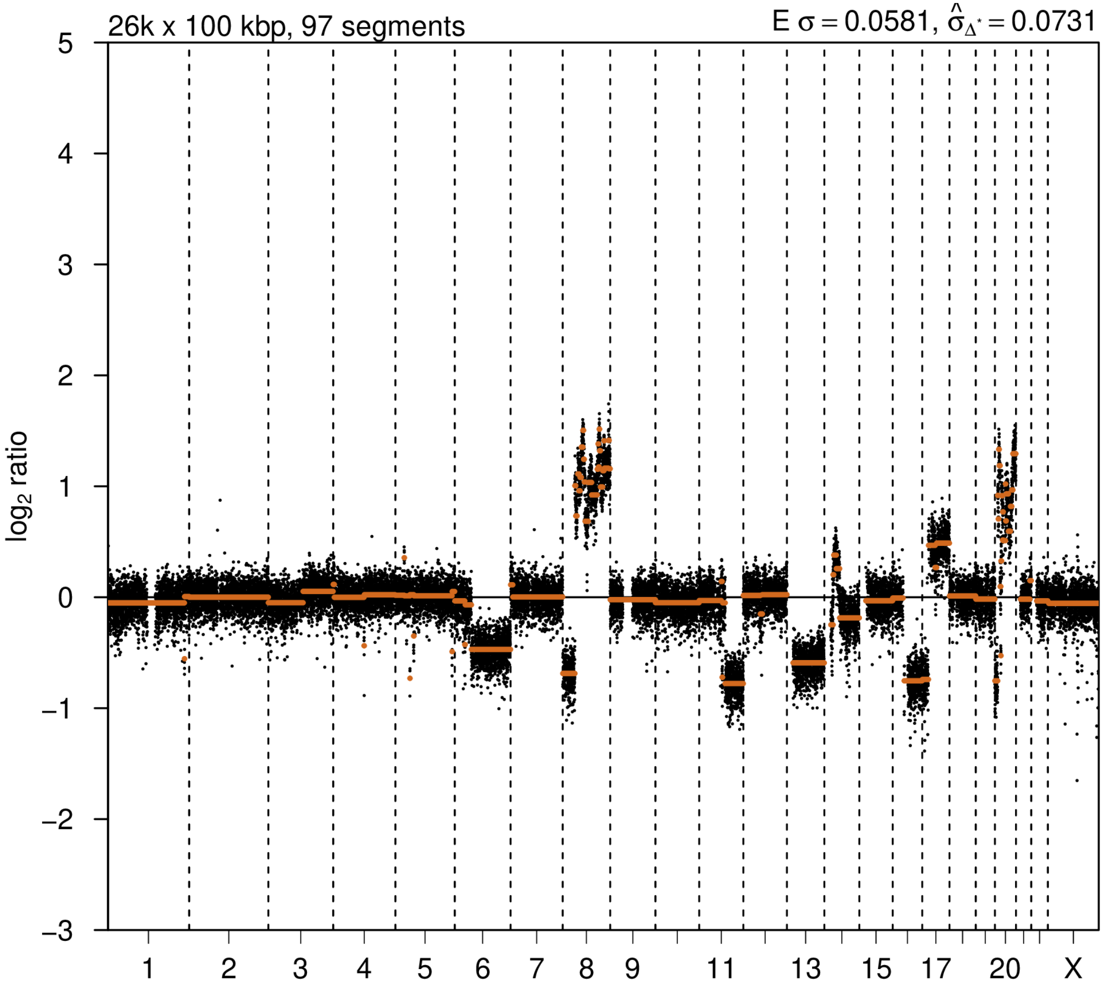

2nd event

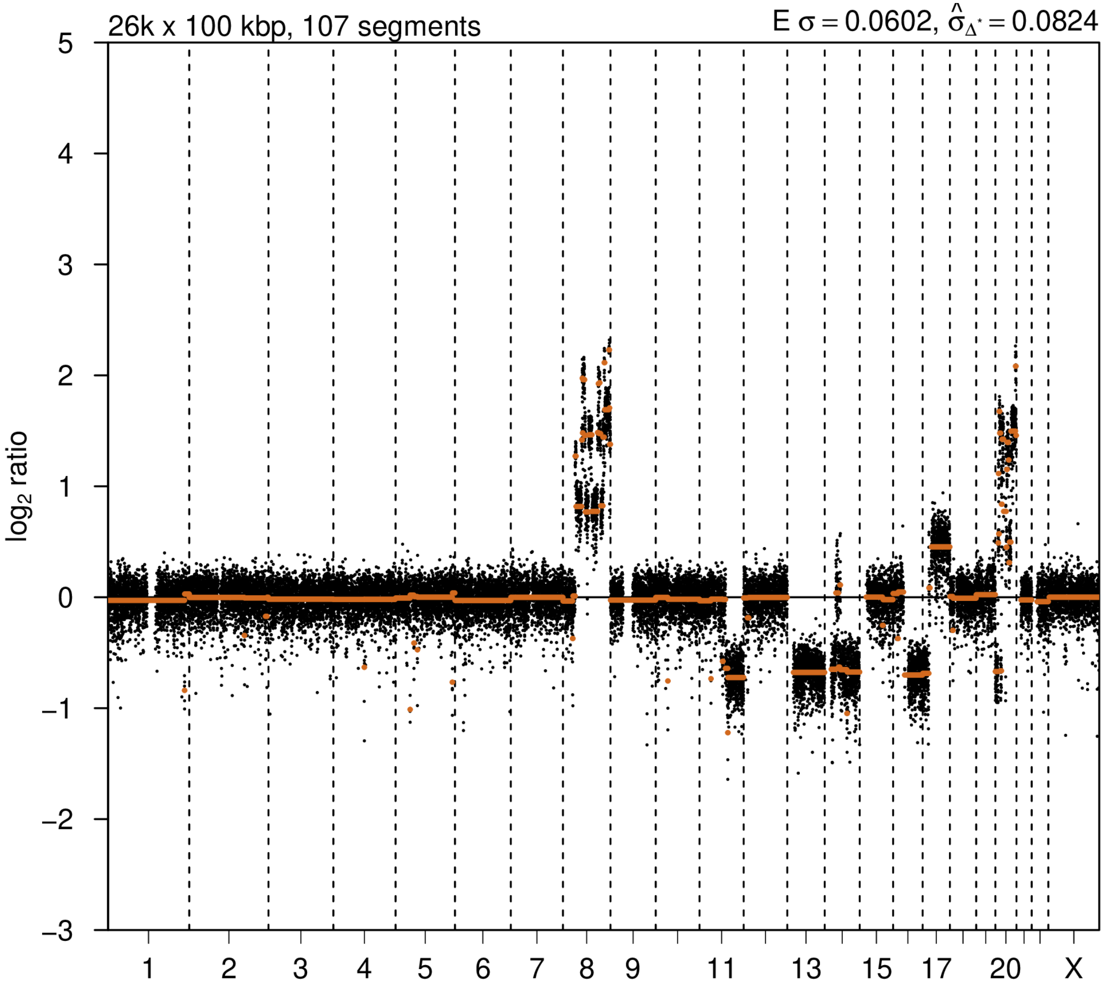

P083

Syn/Meta	Time from 1st surgery to 2nd event (Months)	Side 2nd event	Histology 2nd event	Surgery	AdjuvanT Treatment Pri (RT/ HT)	ER Pri	ER 2nd event	Her2 Pri	Her2 2nd event	Grade Pri	Grade 2nd event	Quadrant 2nd event	Margins	Screening	Clonality P value Copy N	Clonality P value Panel seq	Clonality P value WES	Final verdict
metachronous	13	Ipsilateral	IDC with DCIS	lumpectomy	None	-	-	+	+	3	3	distant from primary	Clear	symptomatic	0.000649 14	0.003	NA	Related

Primary event

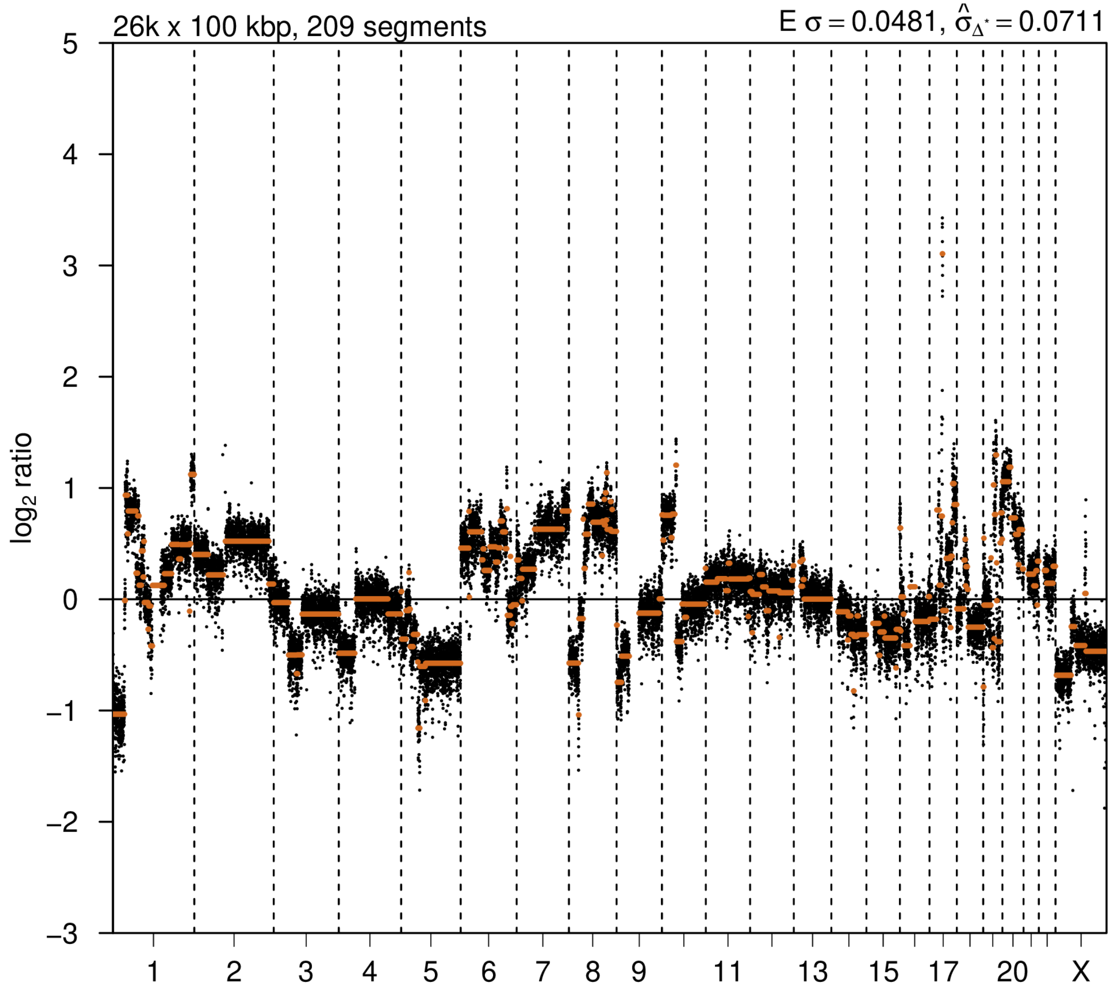

2nd event

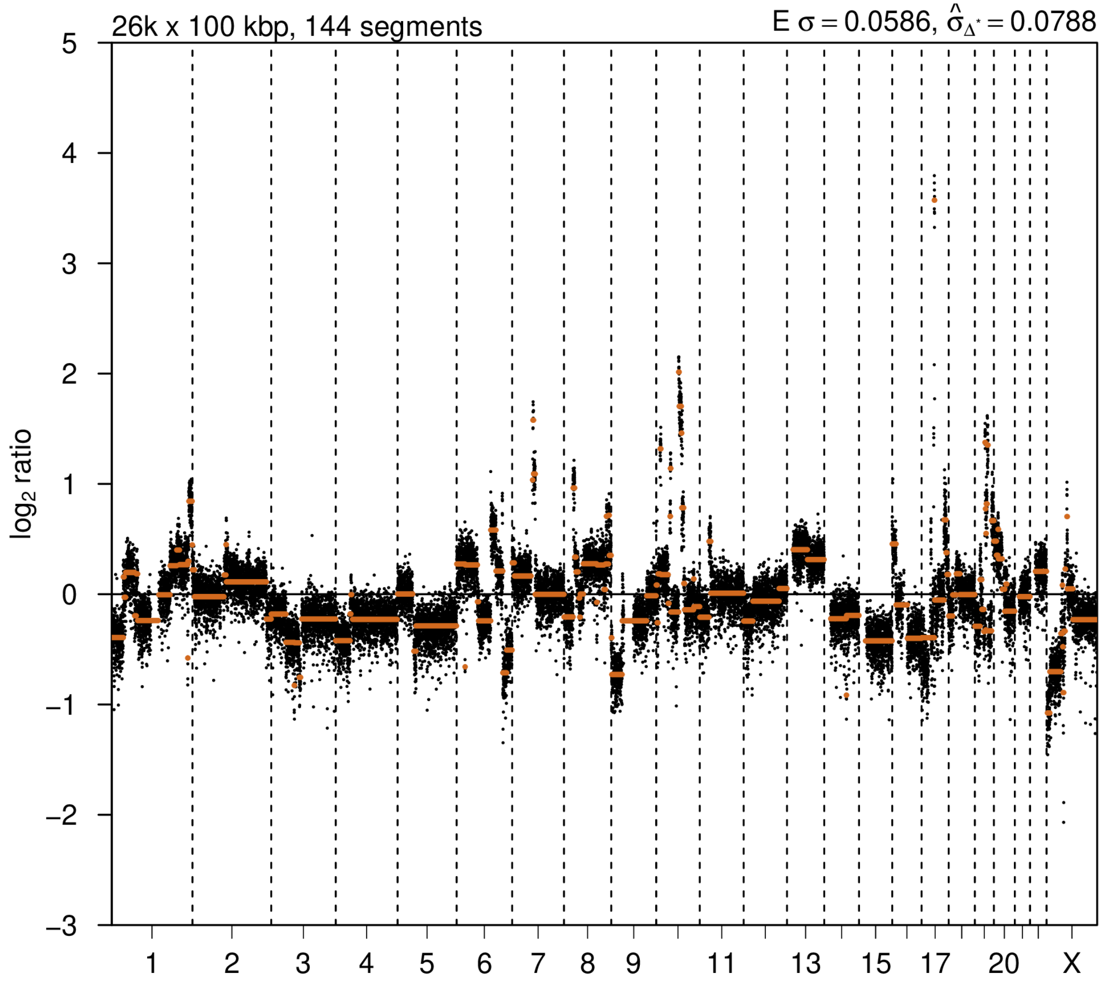

P084

Syn/Meta	Time from 1st surgery to 2nd event (Months)	Side 2nd event	Histology 2nd event	Surgery	AdjuvanT Treatment Pri (RT/ HT)	ER Pri	ER 2nd event	Her2 Pri	Her2 2nd event	Grade Pri	Grade 2nd event	Quadrant 2nd event	Margins	Screening	Clonality P value Copy N	Clonality P value Panel seq	Clonality P value WES	Final verdict
metachronous	13	Ipsilateral	IDC no DCIS	lumpectomy	None	NA	-	NA	-	3	3	distant from primary	Clear	NA	0.007789679	NA	NA	Related

Primary event

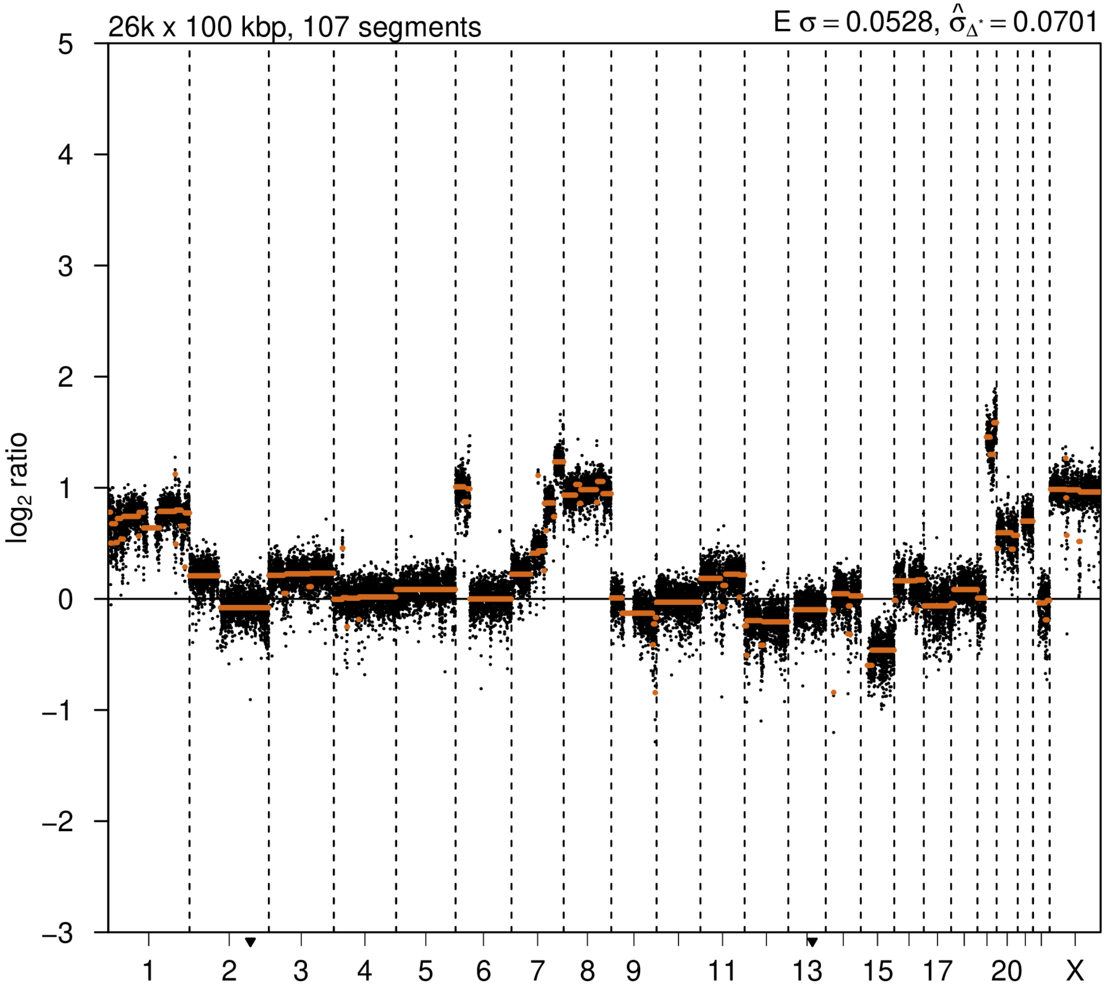

2nd event

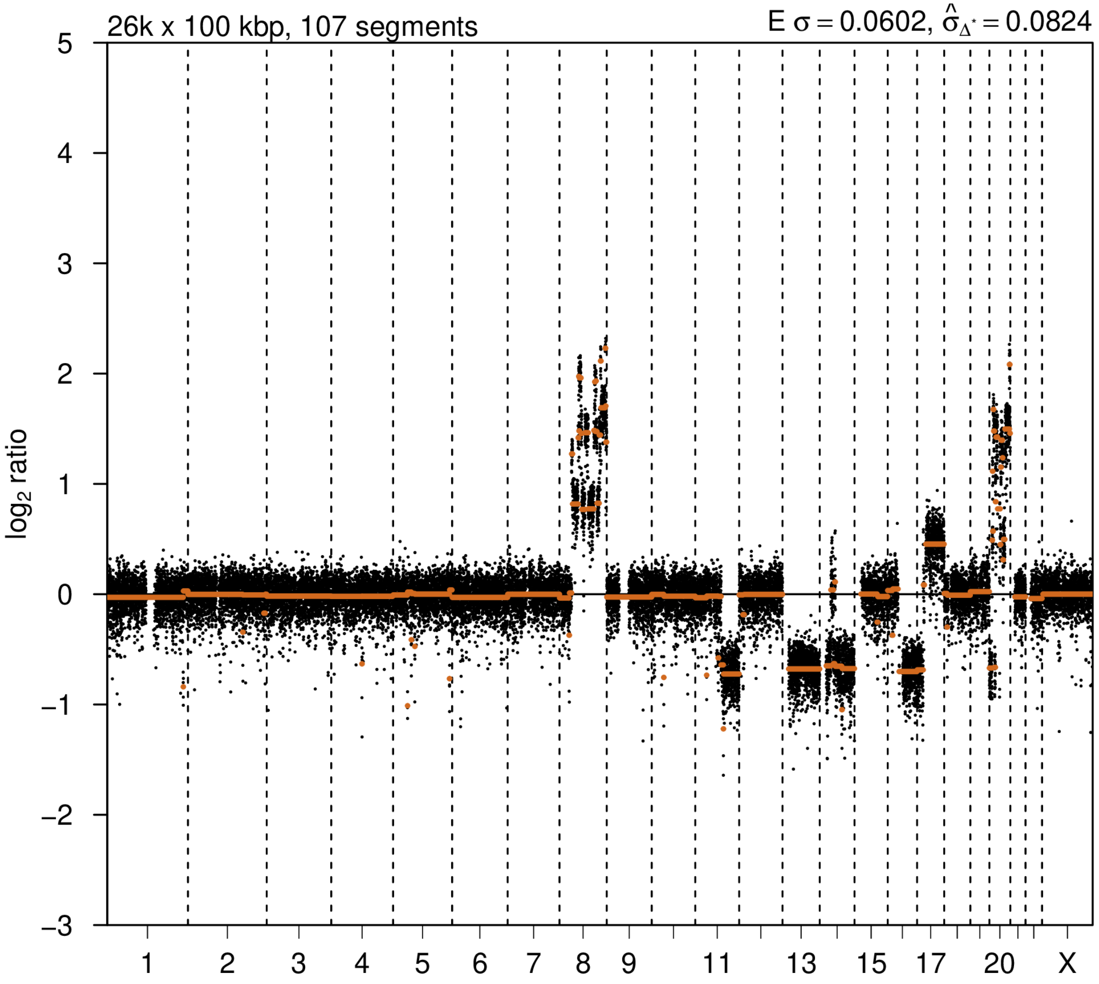

P085

Syn/Meta	Time from 1st surgery to 2nd event (Months)	Side 2nd event	Histology 2nd event	Surgery	AdjuvanT Treatment Pri (RT/ HT)	ER Pri	ER 2nd event	Her2 Pri	Her2 2nd event	Grade Pri	Grade 2nd event	Quadrant 2nd event	Margins	Screening	Clonality P value Copy N	Clonality P value Panel seq	Clonality P value WES	Final verdict
metachronous	26	Ipsilateral	IDC with DCIS	lumpectomy	None	-	-	+	+	2	2	NA	Clear	screen-detected	0.000324 57	0.013	NA	Related

Primary event

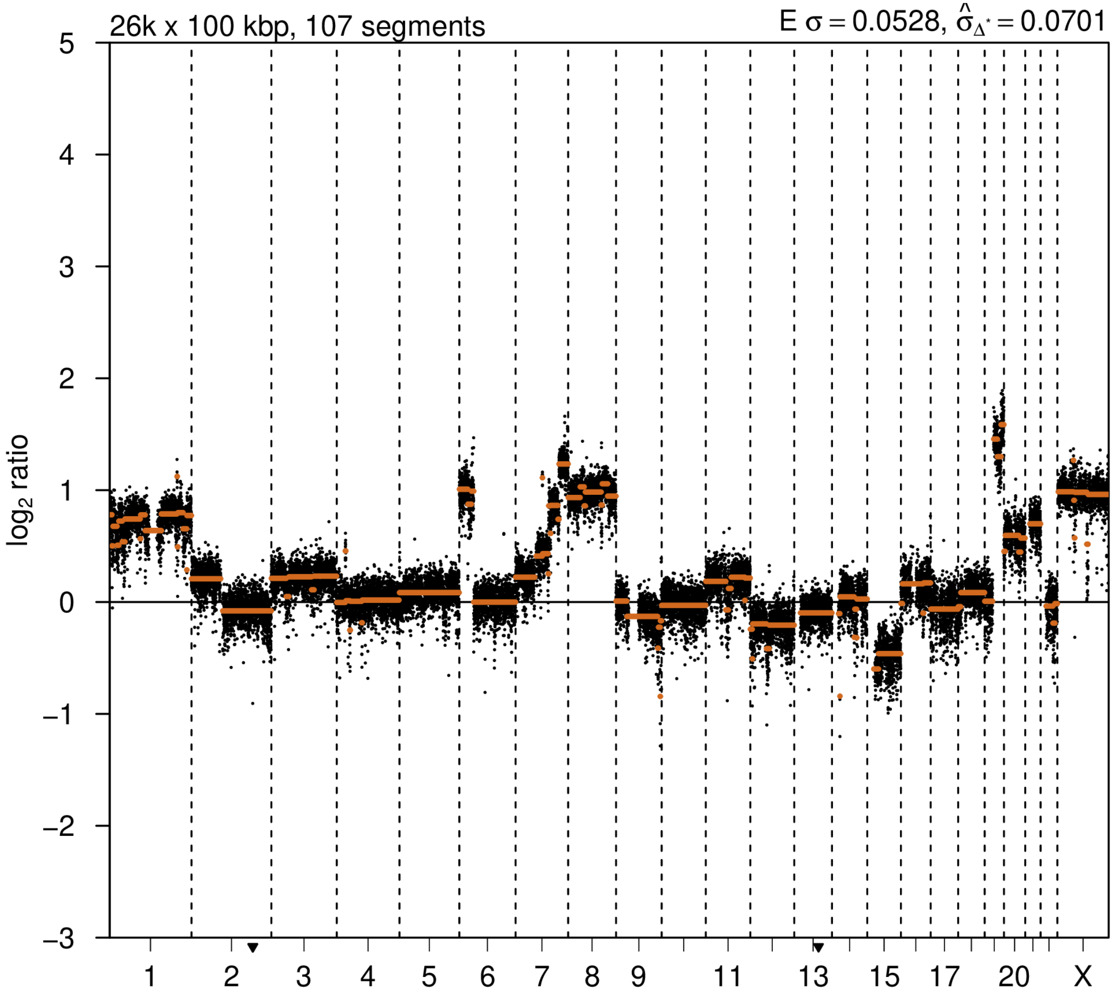

2nd event

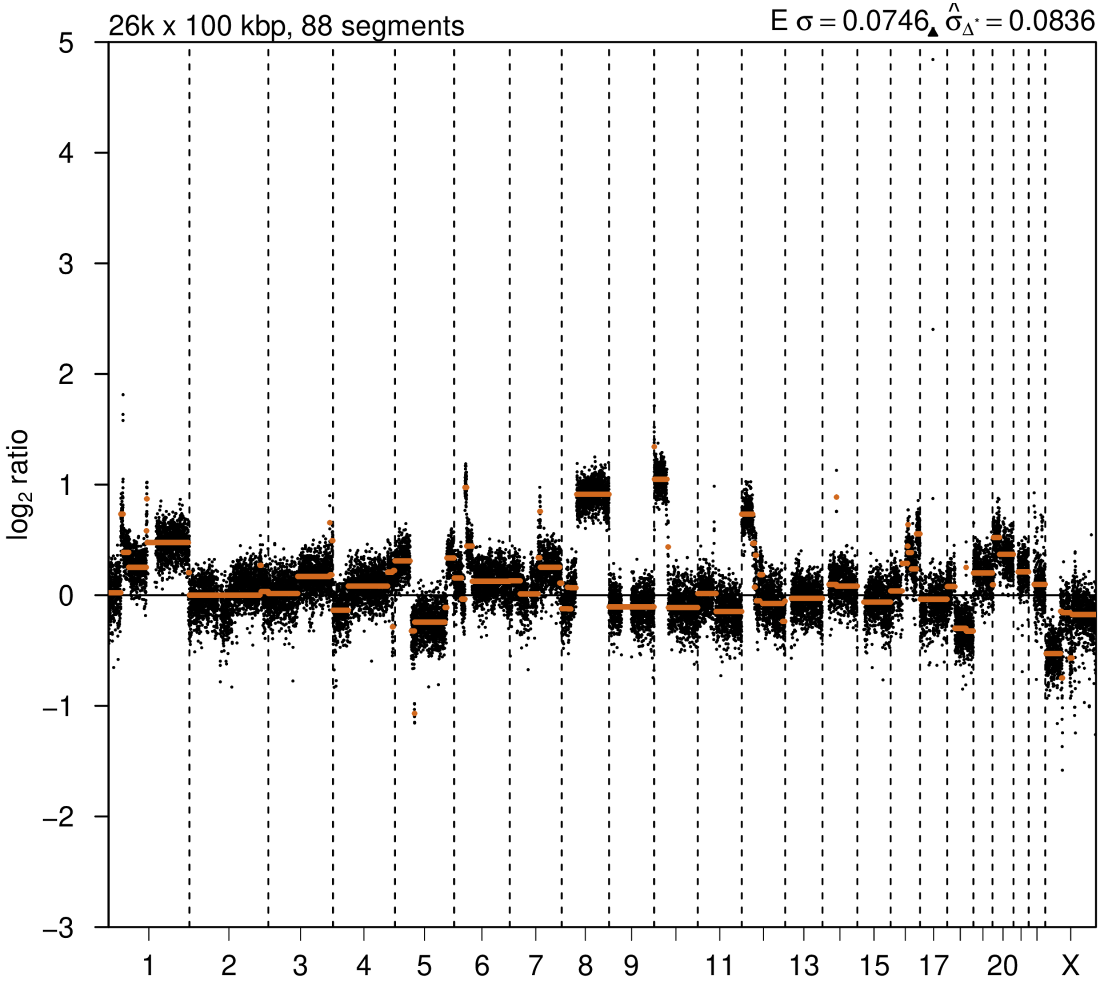

P086

Syn/Meta	Time from 1st surgery to 2nd event (Months)	Side 2nd event	Histology 2nd event	Surgery	AdjuvanT Treatment Pri (RT/ HT)	ER Pri	ER 2nd event	Her2 Pri	Her2 2nd event	Grade Pri	Grade 2nd event	Quadrant 2nd event	Margins	Screening	Clonality P value Copy N	Clonality P value Panel seq	Clonality P value WES	Final verdict
metachronous	27	Ipsilateral	IDC with DCIS	lumpectomy	None	-	-	+	-	2	2	NA	NA	screen-detected	0.107757222	0.003	NA	Related

Primary event

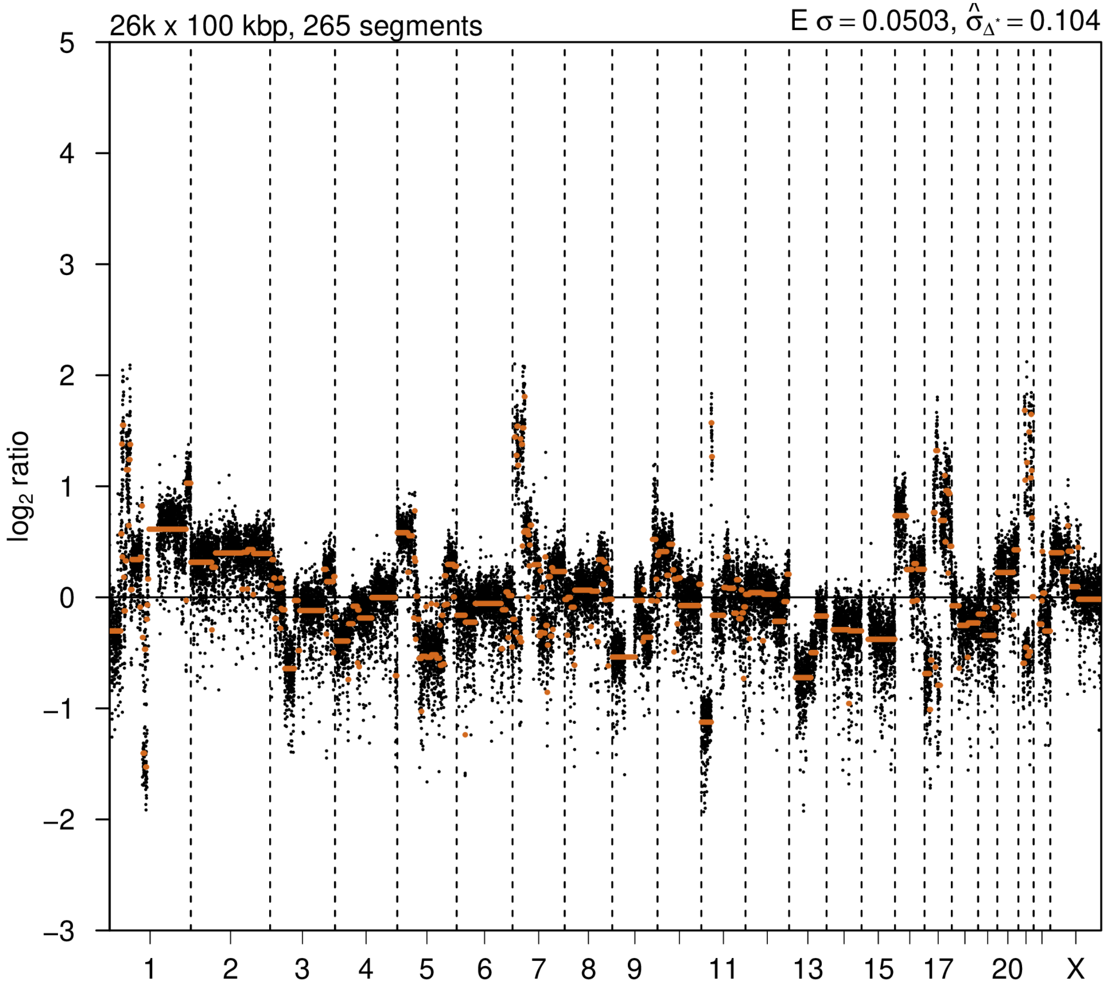

2nd event

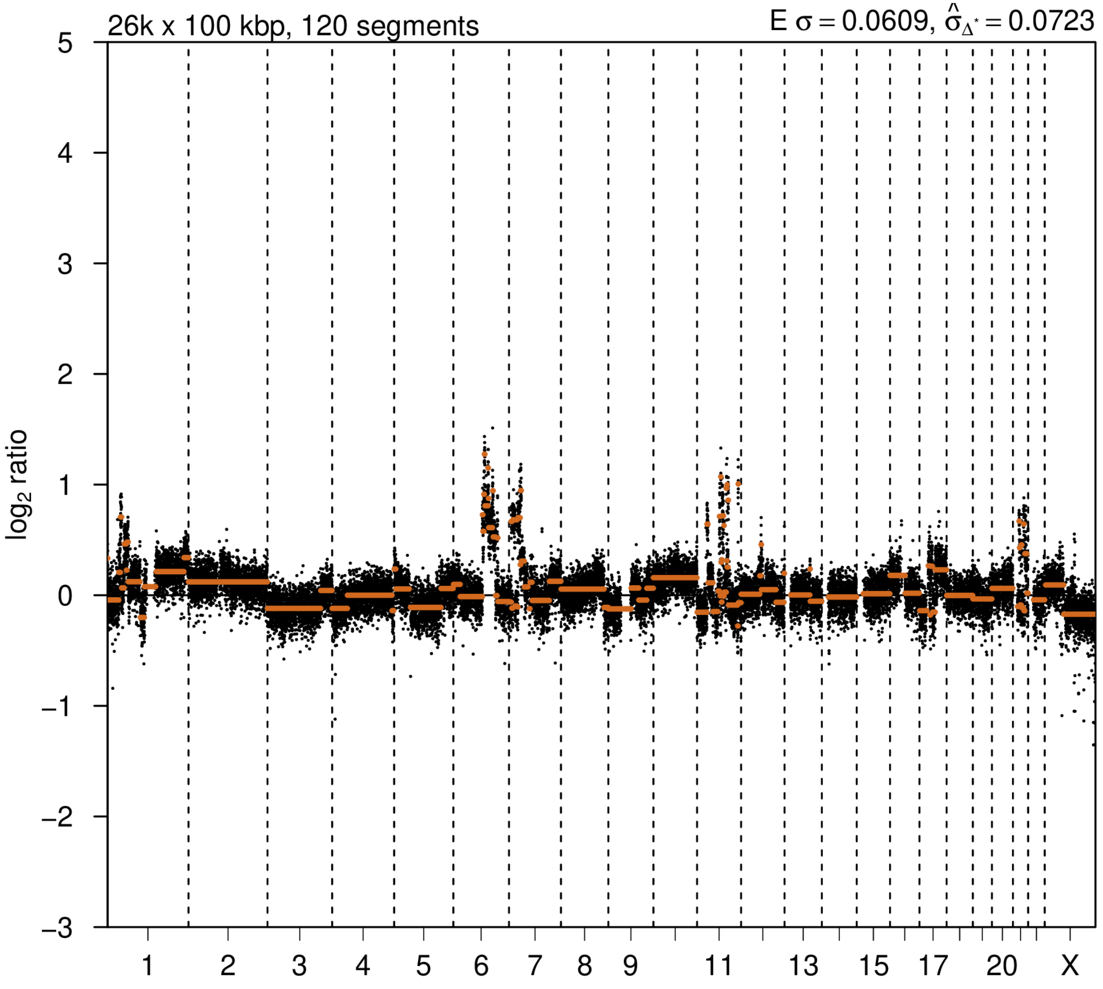

P088

Syn/Meta	Time from 1st surgery to 2nd event (Months)	Side 2nd event	Histology 2nd event	Surgery	AdjuvanT Treatment Pri (RT/ HT)	ER Pri	ER 2nd event	Her2 Pri	Her2 2nd event	Grade Pri	Grade 2nd event	Quadrant 2nd event	Margins	Screening	Clonality P value Copy N	Clonality P value Panel seq	Clonality P value WES	Final verdict
metachronous	28	Ipsilateral	IDC with DCIS	lumpectomy	None	+	+	-	-	2	3	NA	Clear	symptomatic	0.00032457	Single mutation - shared	NA	Related

Primary event

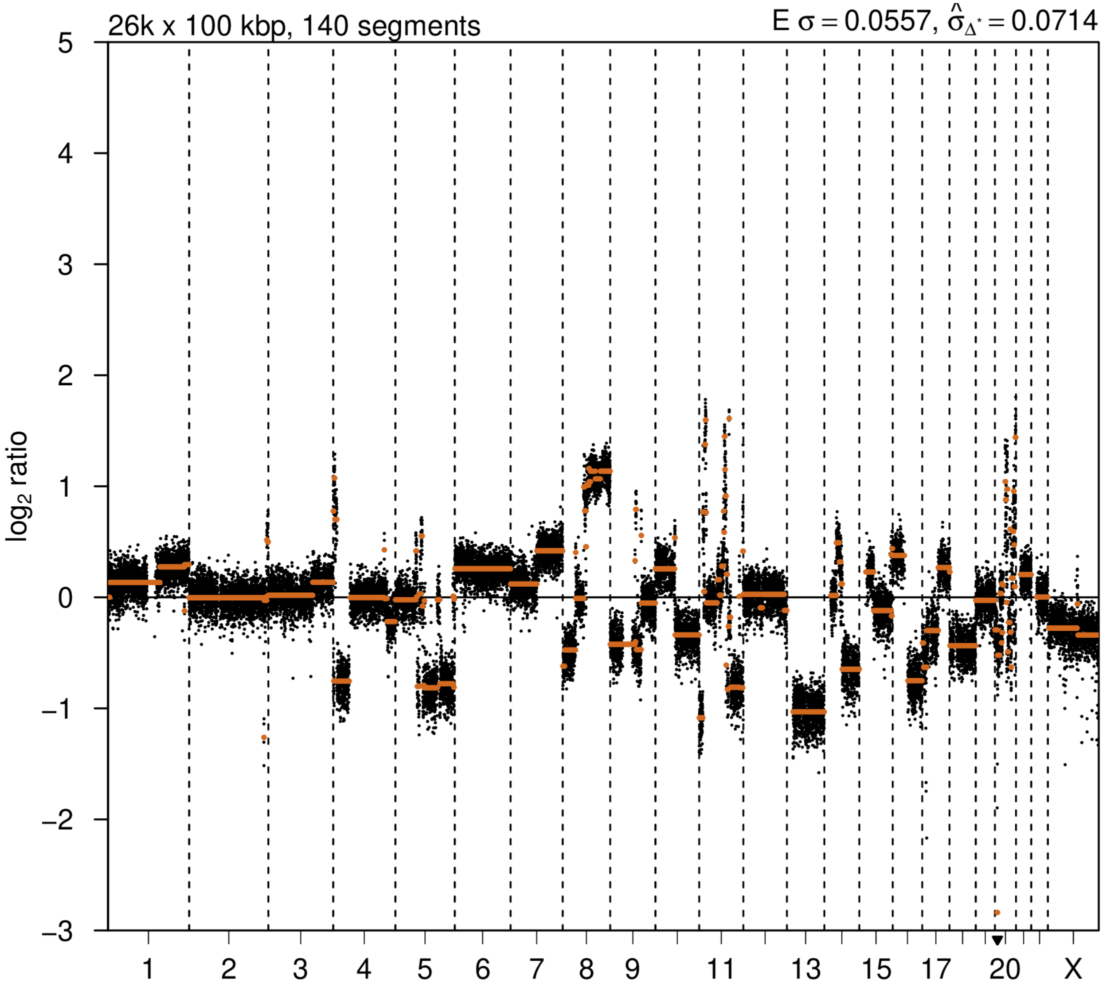

2nd event

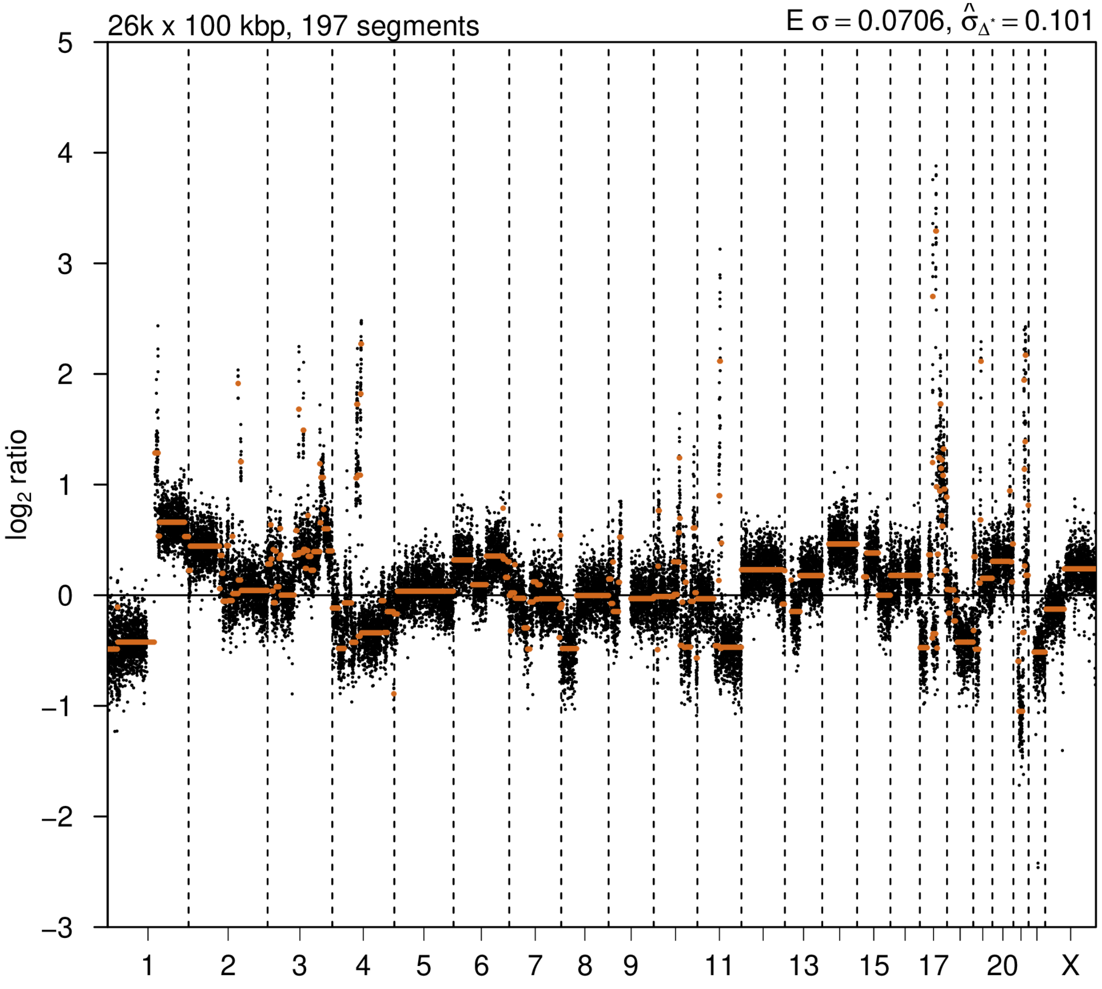

P094

Syn/Meta	Time from 1st surgery to 2nd event (Months)	Side 2nd event	Histology 2nd event	Surgery	AdjuvanT Treatment Pri (RT/ HT)	ER Pri	ER 2nd event	Her2 Pri	Her2 2nd event	Grade Pri	Grade 2nd event	Quadrant 2nd event	Margins	Screening	Clonality P value Copy N	Clonality P value Panel seq	Clonality P value WES	Final verdict
metachronous	29	Ipsilateral	IDC no DCIS	lumpectomy	None	+	+	-	-	2	2	at or adjacent to primary	Clear	symptomatic	0.00032457	NA	NA	Related

Primary event

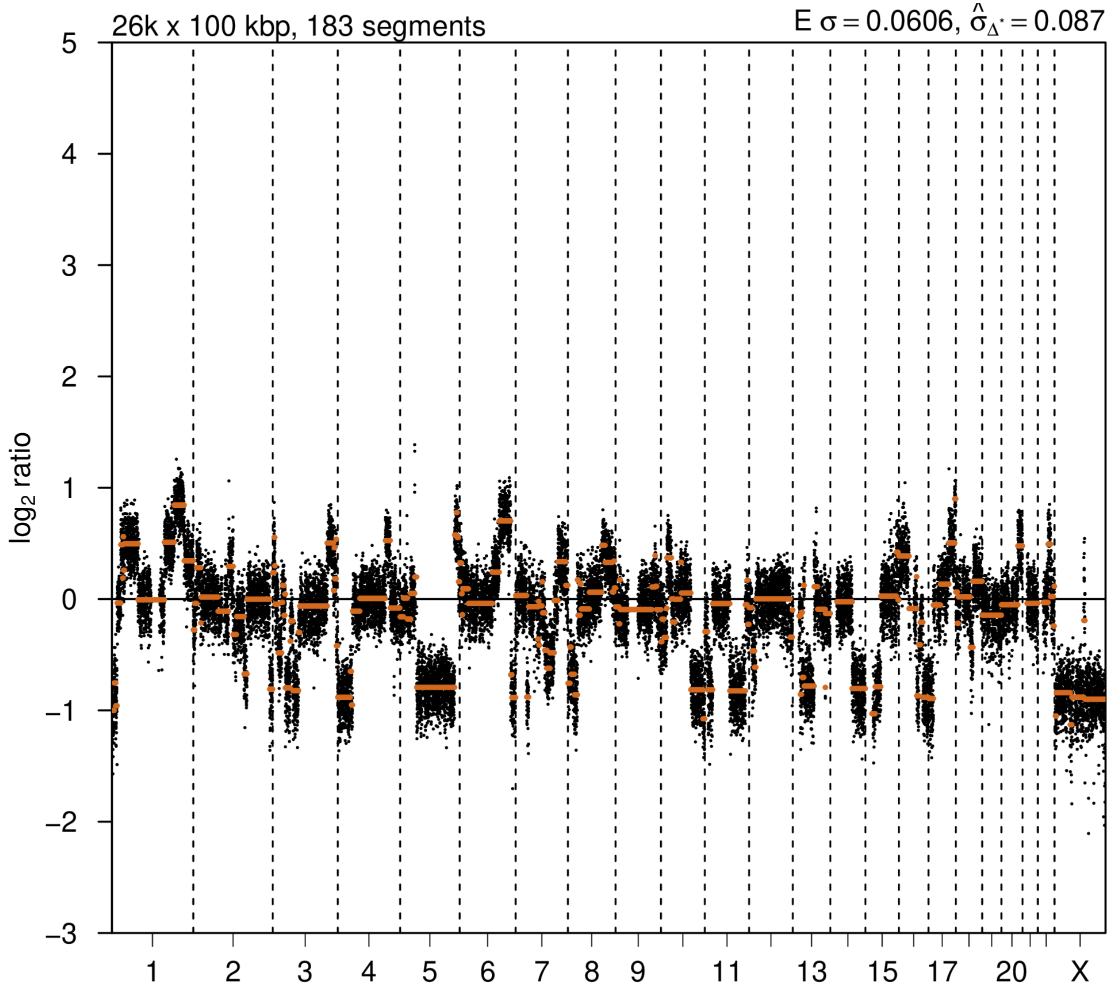

2nd event

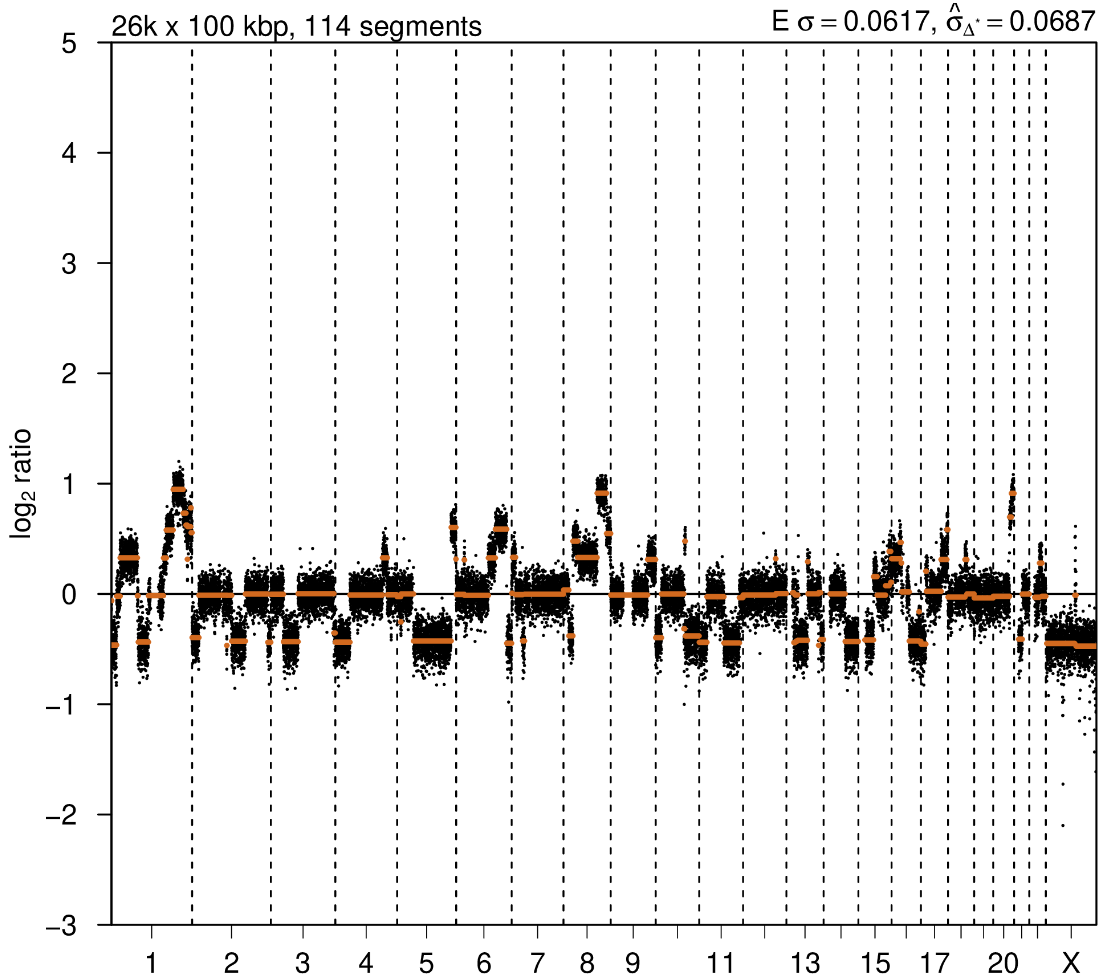

P095

Syn/Meta	Time from 1st surgery to 2nd event (Months)	Side 2nd event	Histology 2nd event	Surgery	AdjuvanT Treatment Pri (RT/ HT)	ER Pri	ER 2nd event	Her2 Pri	Her2 2nd event	Grade Pri	Grade 2nd event	Quadrant 2nd event	Margins	Screening	Clonality P value Copy N	Clonality P value Panel seq	Clonality P value WES	Final verdict
metachronous	25	Ipsilateral	IDC with DCIS	lumpectomy	None	+	+	-	-	2	1	at or adjacent to primary	Clear	screen-detected	0.000324 57	0.006	NA	Related

Primary event

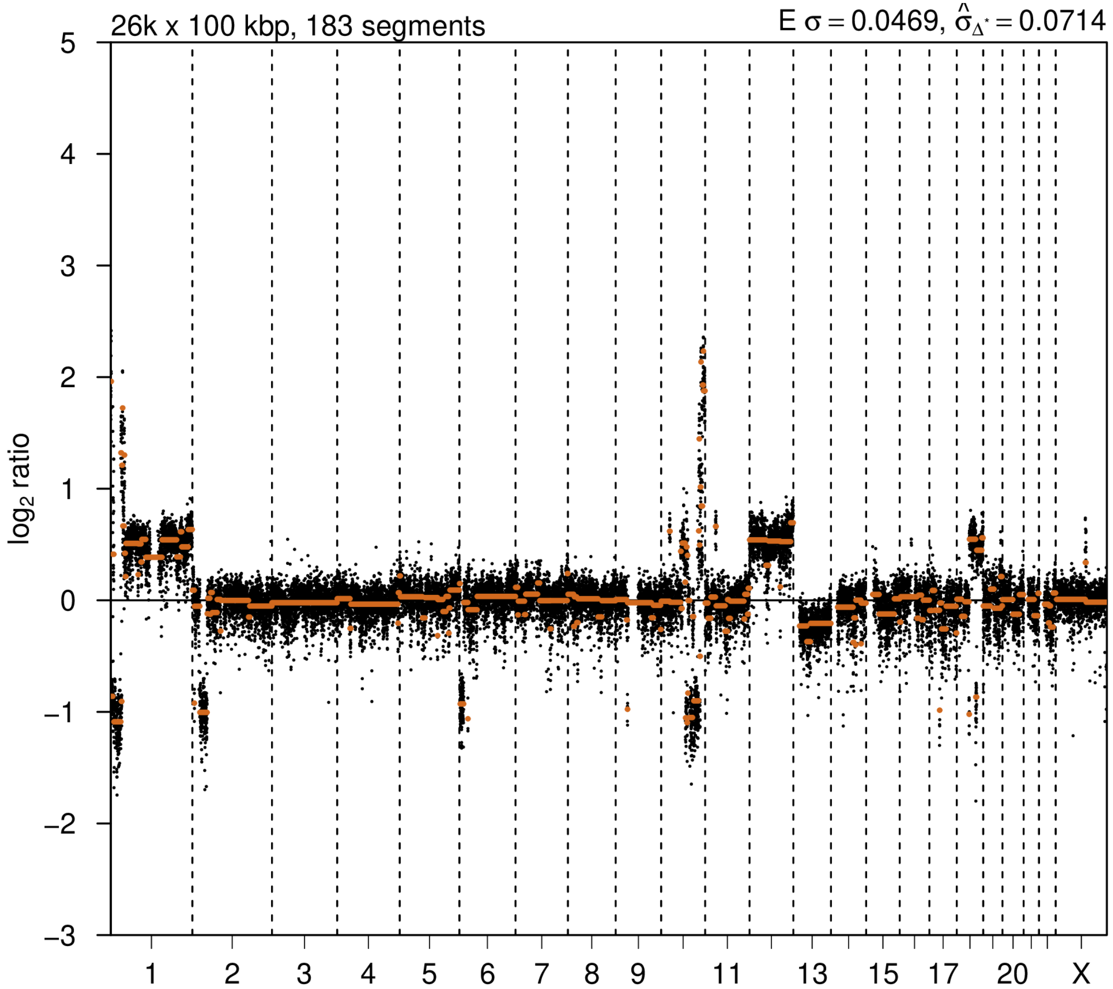

2nd event

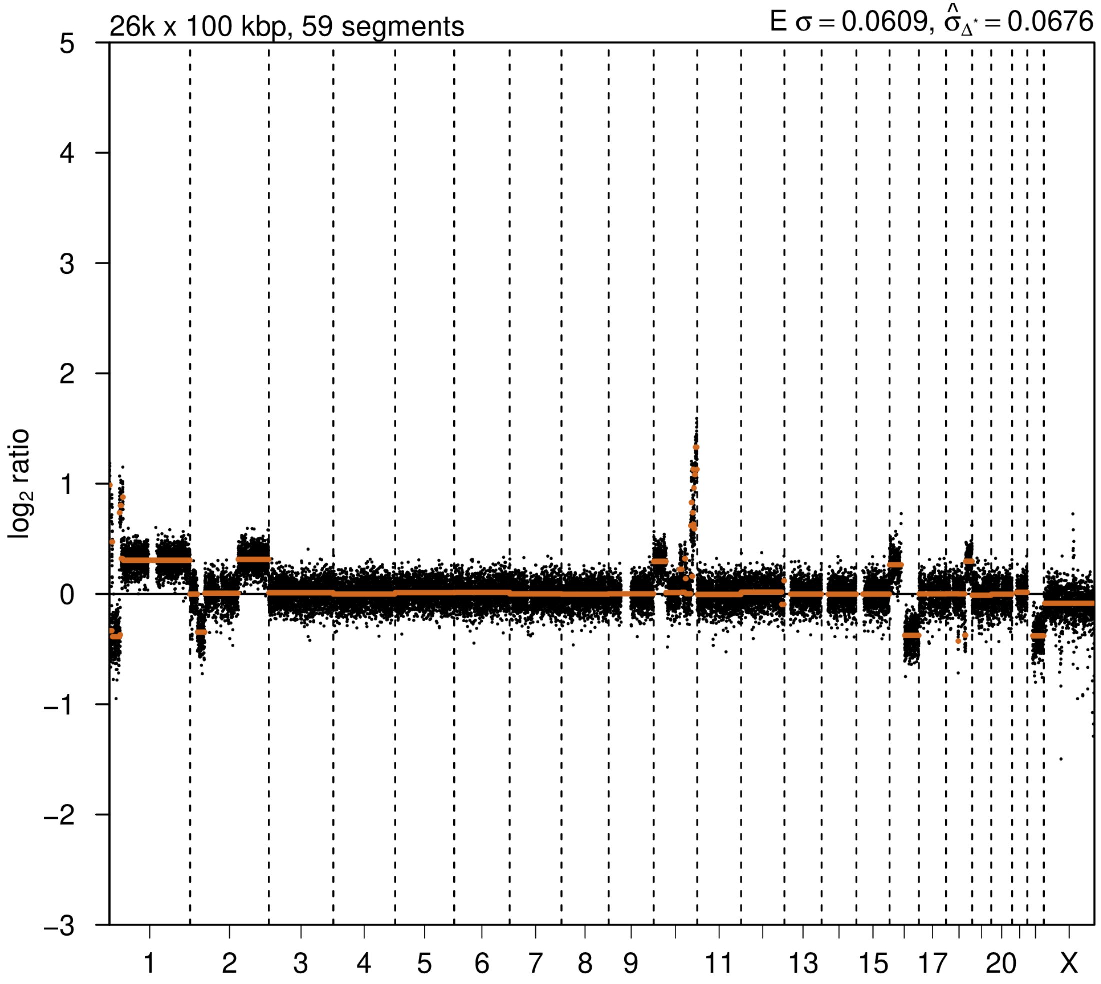

P096

Syn/Meta	Time from 1st surgery to 2nd event (Months)	Side 2nd event	Histology 2nd event	Surgery	AdjuvanT Treatment Pri (RT/ HT)	ER Pri	ER 2nd event	Her2 Pri	Her2 2nd event	Grade Pri	Grade 2nd event	Quadrant 2nd event	Margins	Screening	Clonality P value Copy N	Clonality P value Panel seq	Clonality P value WES	Final verdict
metachronous	23	Ipsilateral	IDC with DCIS	lumpectomy	None	+	+	-	-	2	3	at or adjacent to primary	Clear	symptomatic	0.00032457	NA	0.000752445	Related

Primary event

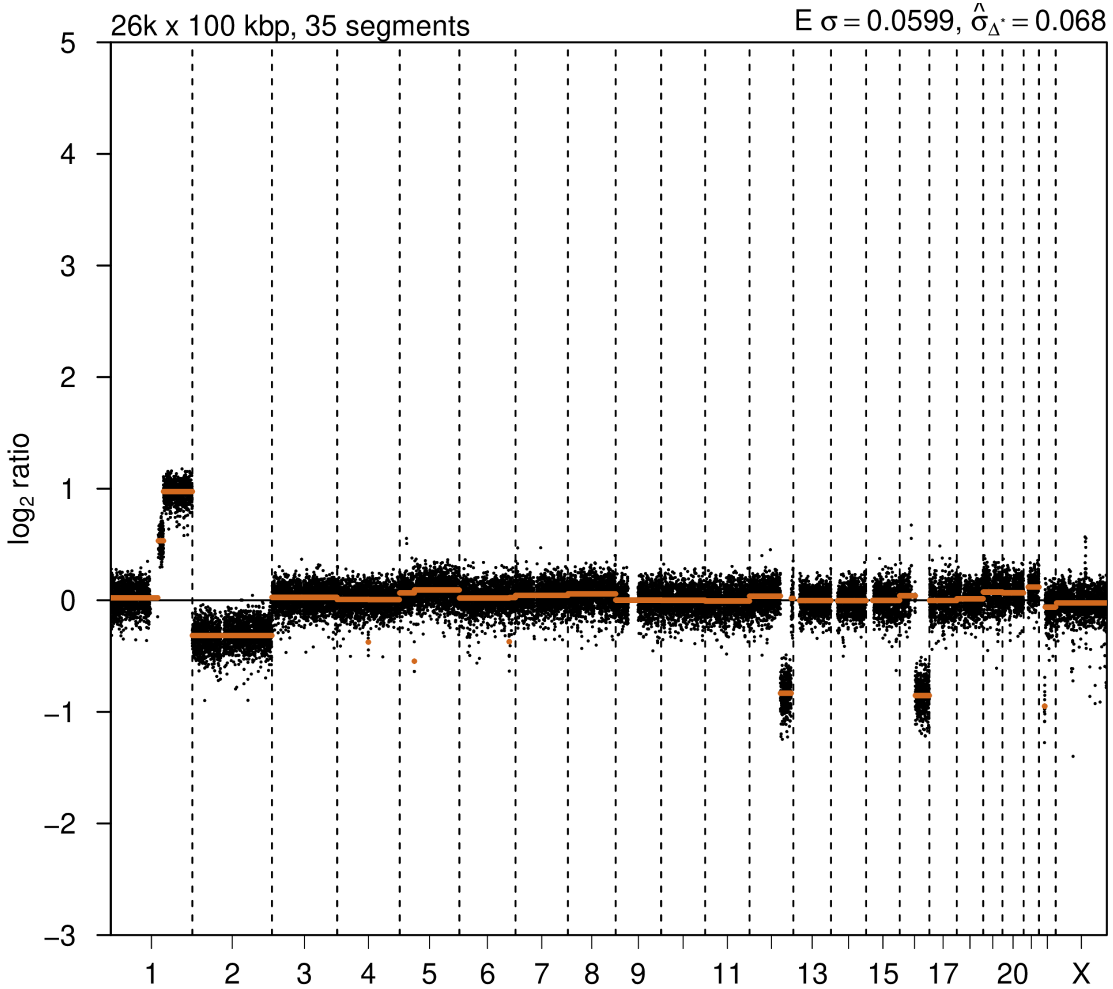

2nd event

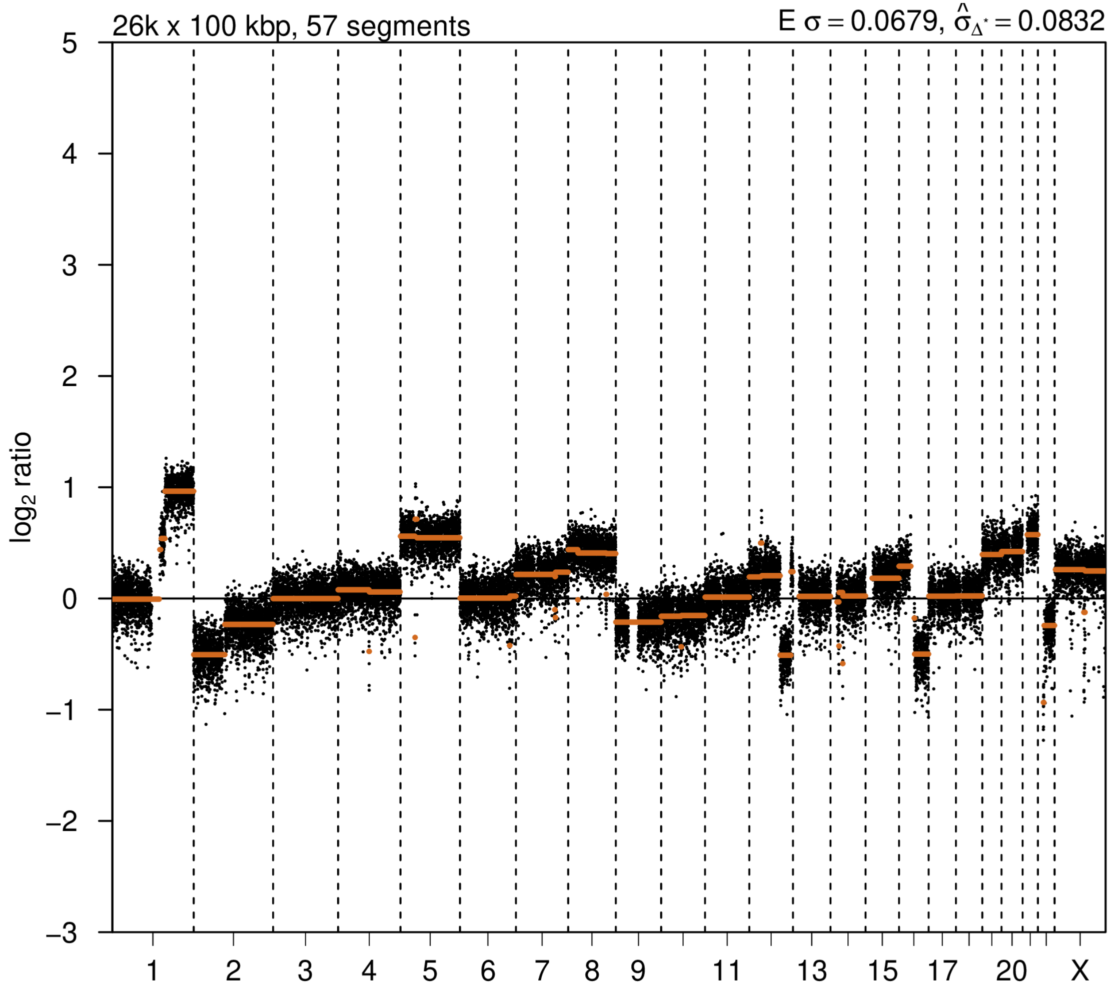

P102

Syn/Meta	Time from 1st surgery to 2nd event (Months)	Side 2nd event	Histology 2nd event	Surgery	AdjuvanT Treatment Pri (RT/ HT)	ER Pri	ER 2nd event	Her2 Pri	Her2 2nd event	Grade Pri	Grade 2nd event	Quadrant 2nd event	Margins	Screening	Clonality P value Copy N	Clonality P value Panel seq	Clonality P value WES	Final verdict
metachronous	184	Ipsilateral	IDC no DCIS	lumpectomy	None	+	+	-	-	1	2	NA	NA	symptomatic	0.003570269	Single mutation - shared	1	Equivocal

Primary event

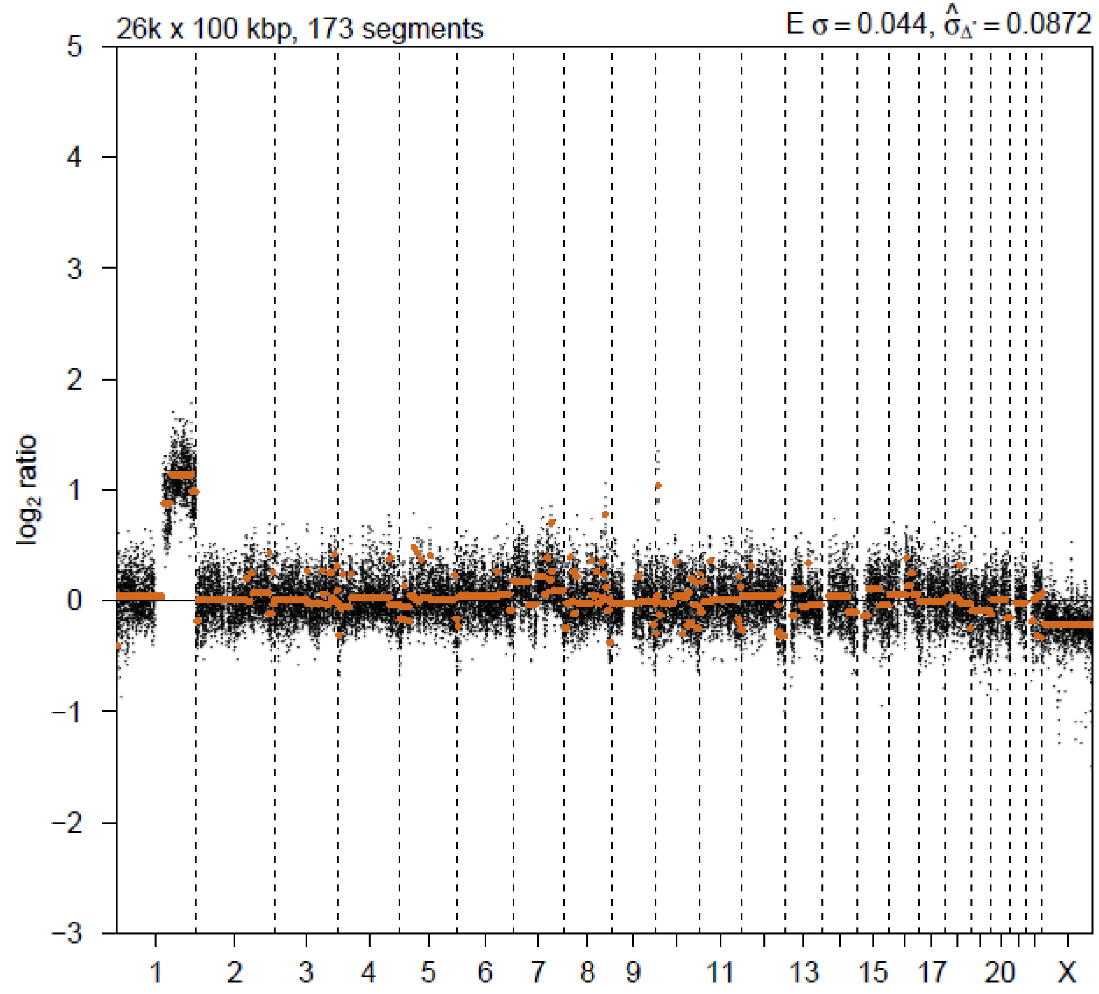

2nd event

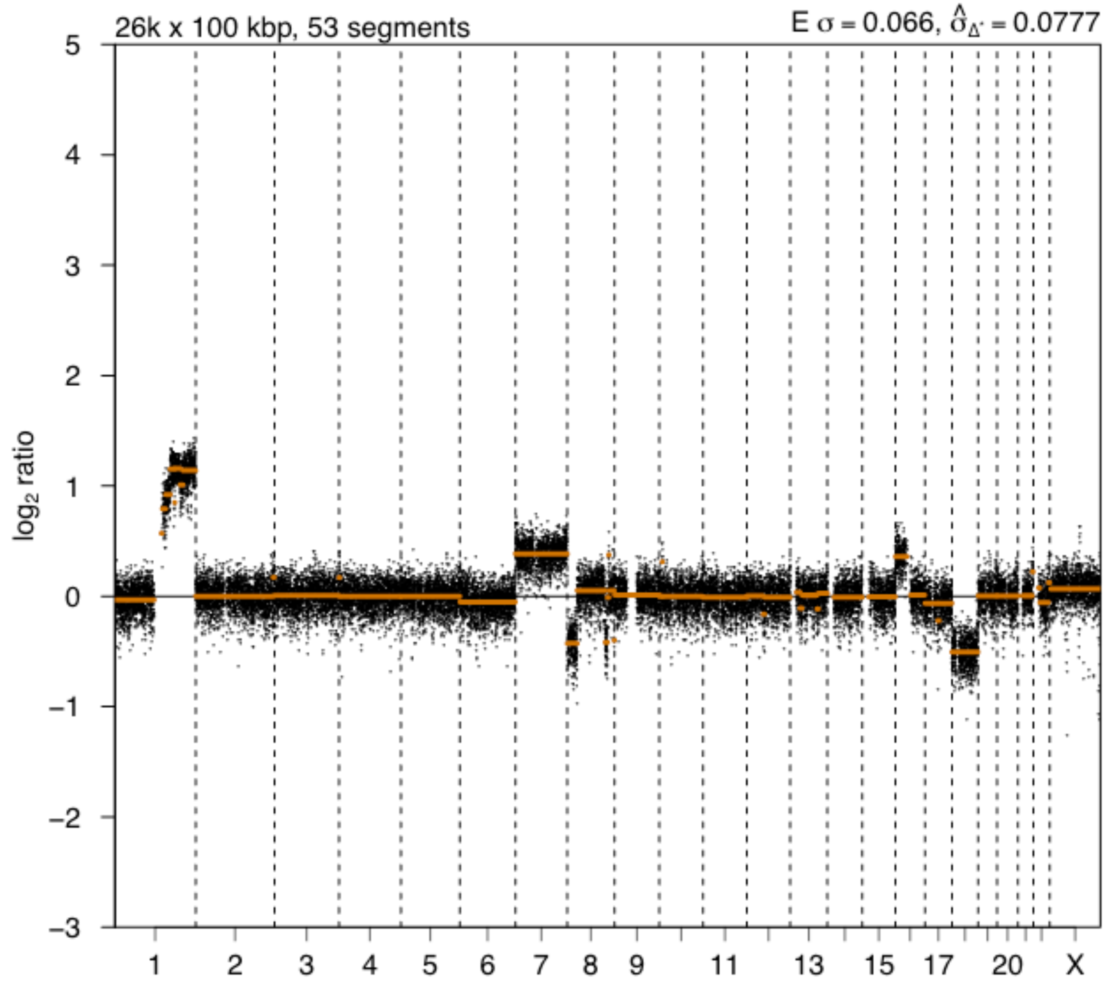

P103

Syn/Meta	Time from 1st surgery to 2nd event (Months)	Side 2nd event	Histology 2nd event	Surgery	AdjuvanT Treatment Pri (RT/ HT)	ER Pri	ER 2nd event	Her2 Pri	Her2 2nd event	Grade Pri	Grade 2nd event	Quadrant 2nd event	Margins	Screening	Clonality P value Copy N	Clonality P value Panel seq	Clonality P value WES	Final verdict
metachronous	119	Ipsilateral	IDC with DCIS	lumpectomy	None	+	+	-	-	2	3	distant from primary	Clear	symptomatic	0.214540734	0.025	NA	Equivocal

Primary event

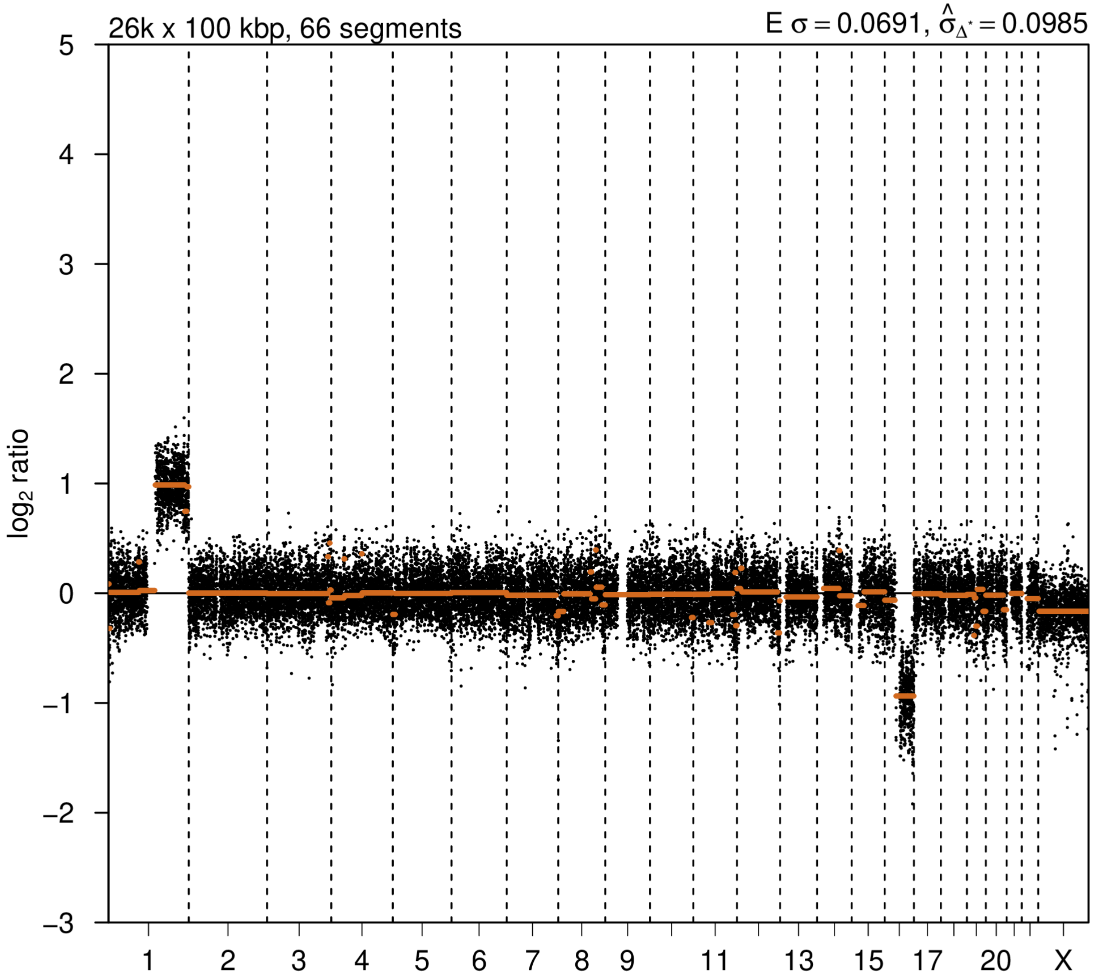

2nd event

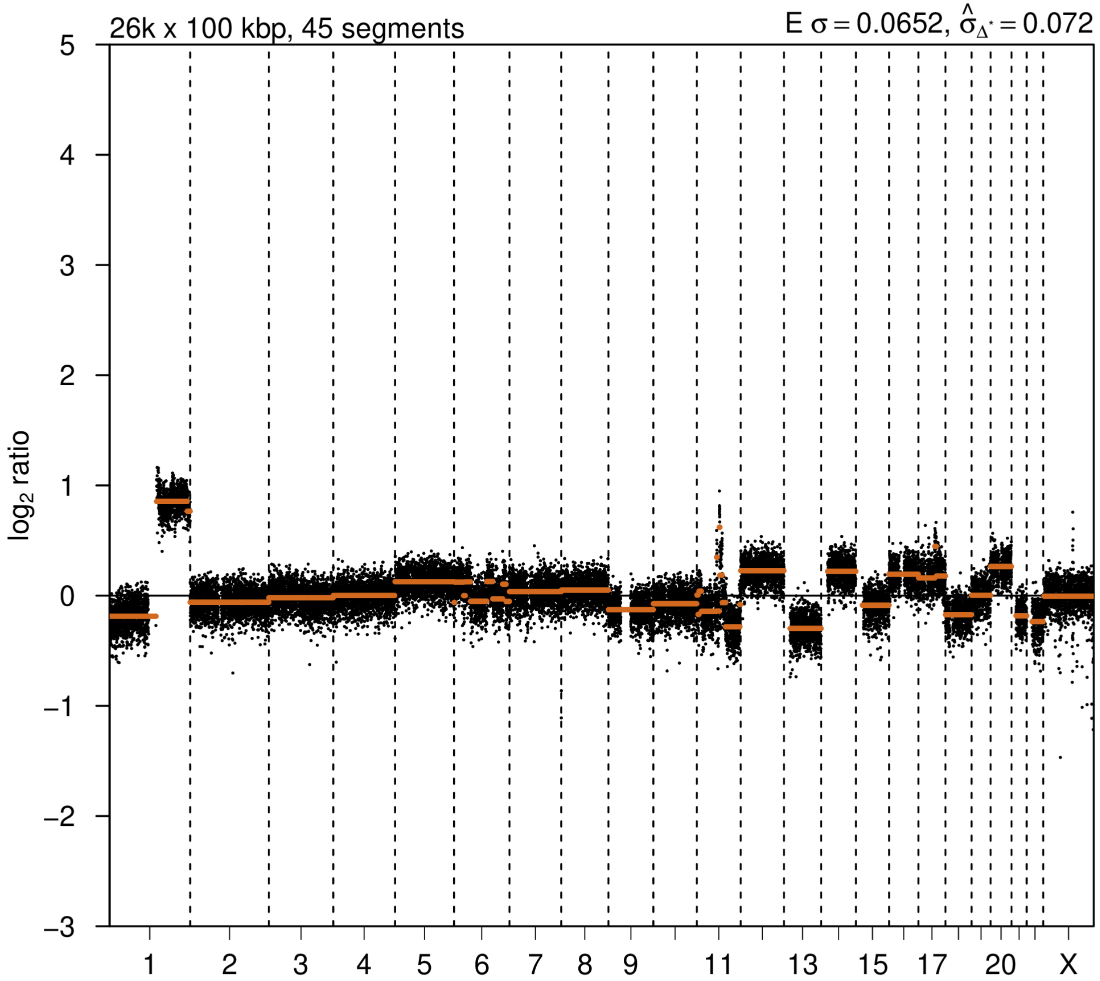

P104

Syn/Meta	Time from 1st surgery to 2nd event (Months)	Side 2nd event	Histology 2nd event	Surgery	AdjuvanT Treatment Pri (RT/ HT)	ER Pri	ER 2nd event	Her2 Pri	Her2 2nd event	Grade Pri	Grade 2nd event	Quadrant 2nd event	Margins	Screening	Clonality P value Copy N	Clonality P value Panel seq	Clonality P value WES	Final verdict
metachronous	104	Ipsilateral	IDC with DCIS	lumpectomy	None	+	+	-	-	1	1	at or adjacent to primary	Clear	symptomatic	0.045439792	0.02	NA	Equivocal

Primary event

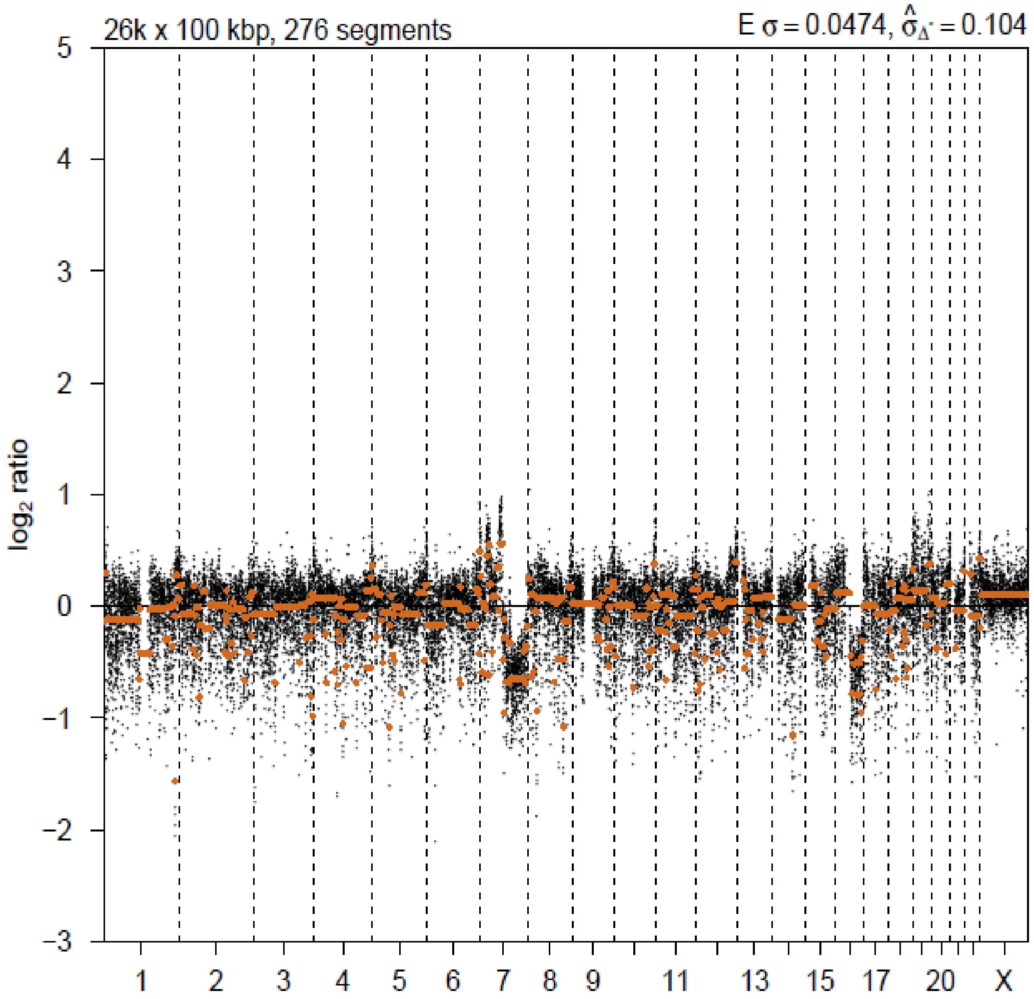

2nd event

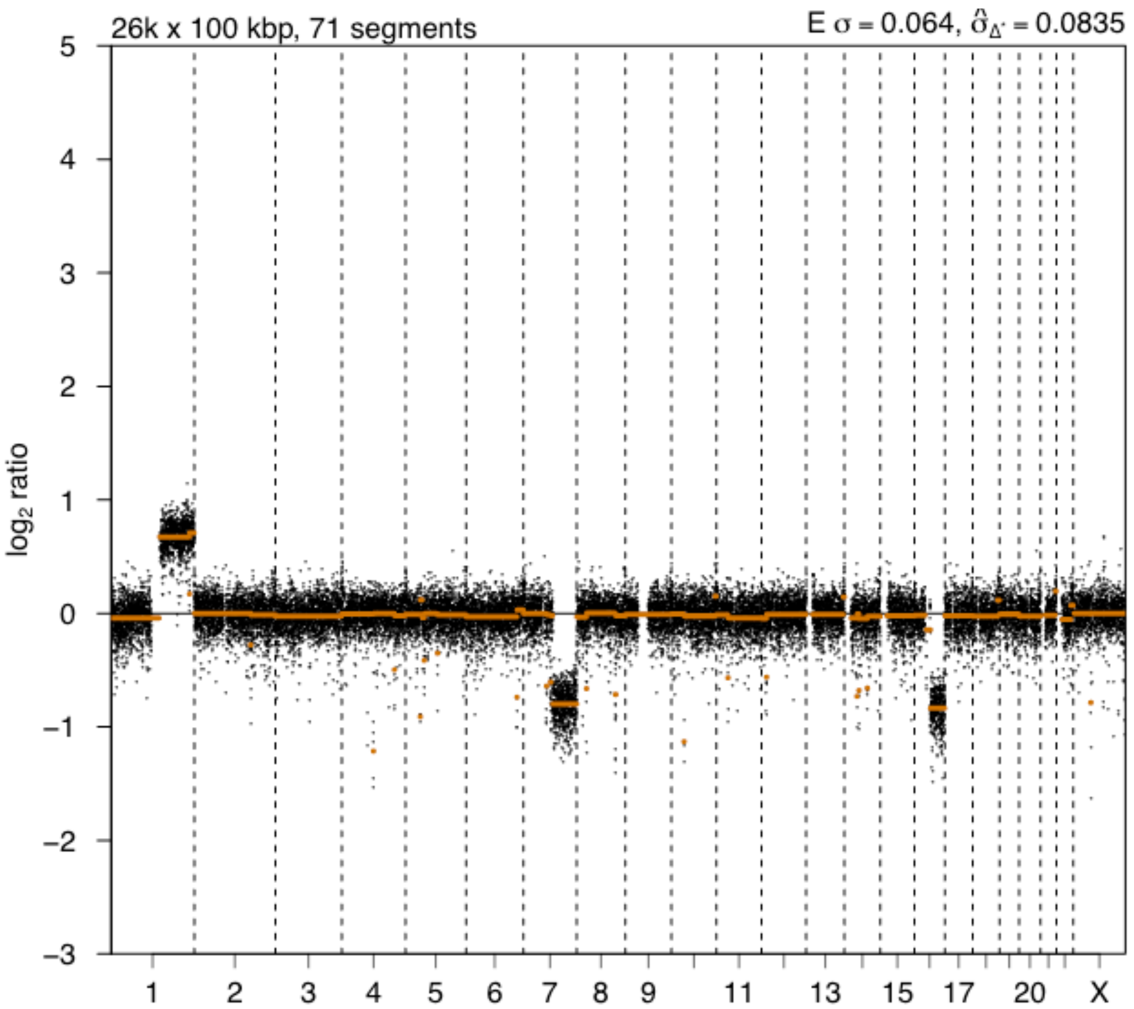

P105

Syn/Meta	Time from 1st surgery to 2nd event (Months)	Side 2nd event	Histology 2nd event	Surgery	AdjuvanT Treatment Pri (RT/ HT)	ER Pri	ER 2nd event	Her2 Pri	Her2 2nd event	Grade Pri	Grade 2nd event	Quadrant 2nd event	Margins	Screening	Clonality P value Copy N	Clonality P value Panel seq	Clonality P value WES	Final verdict
metachronous	90	Ipsilateral	IDC no DCIS	lumpectomy	None	+	+	-	-	2	3	at or adjacent to primary	Clear	screen-detected	0.127880558	Single mutation - shared	1	Equivocal

Primary event

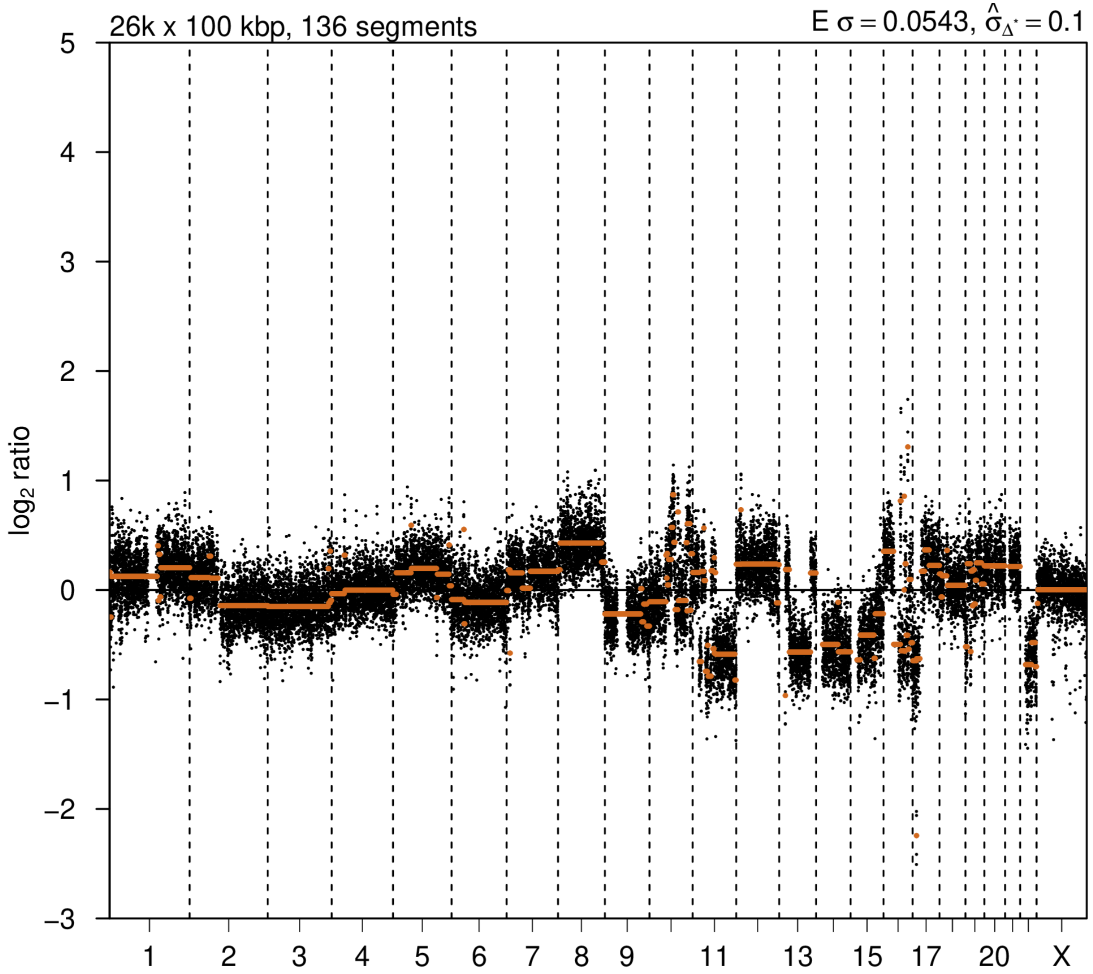

2nd event

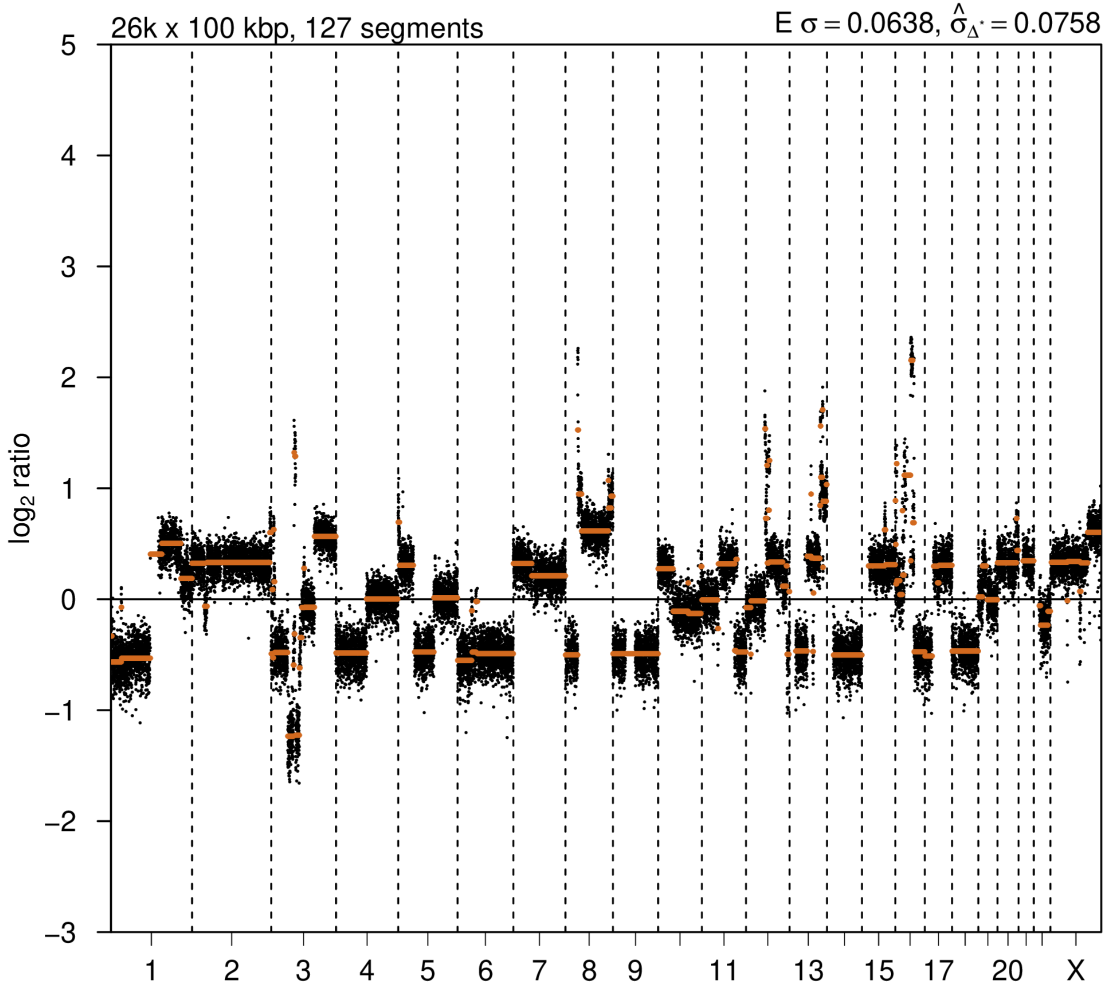

P106

Syn/Meta	Time from 1st surgery to 2nd event (Months)	Side 2nd event	Histology 2nd event	Surgery	AdjuvanT Treatment Pri (RT/ HT)	ER Pri	ER 2nd event	Her2 Pri	Her2 2nd event	Grade Pri	Grade 2nd event	Quadrant 2nd event	Margins	Screening	Clonality P value Copy N	Clonality P value Panel seq	Clonality P value WES	Final verdict
metachronous	80	Ipsilateral	IDC with DCIS	lumpectomy	None	+	+	-	-	2	1	at or adjacent to primary	involved	screen-detected	0.271989614	0.013	NA	Equivocal

Primary event

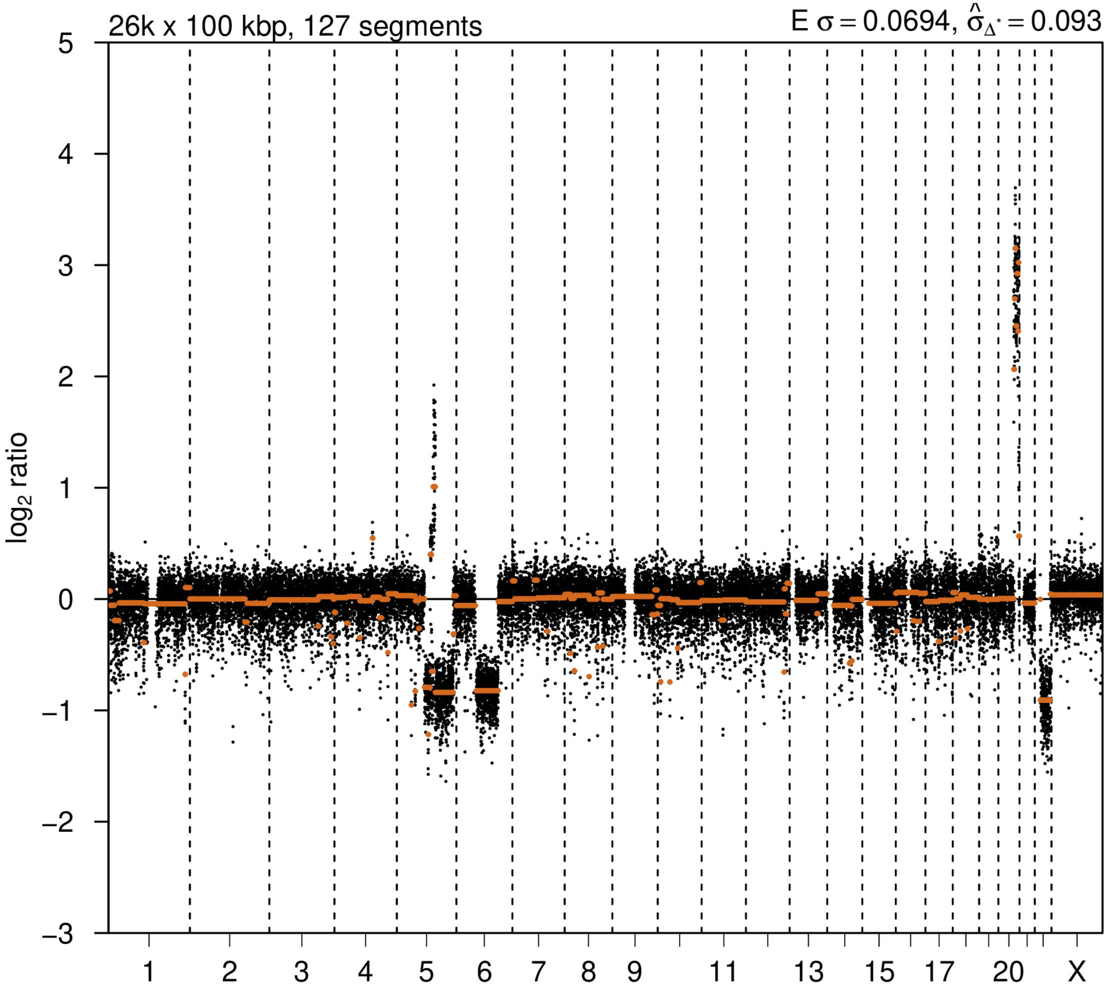

2nd event

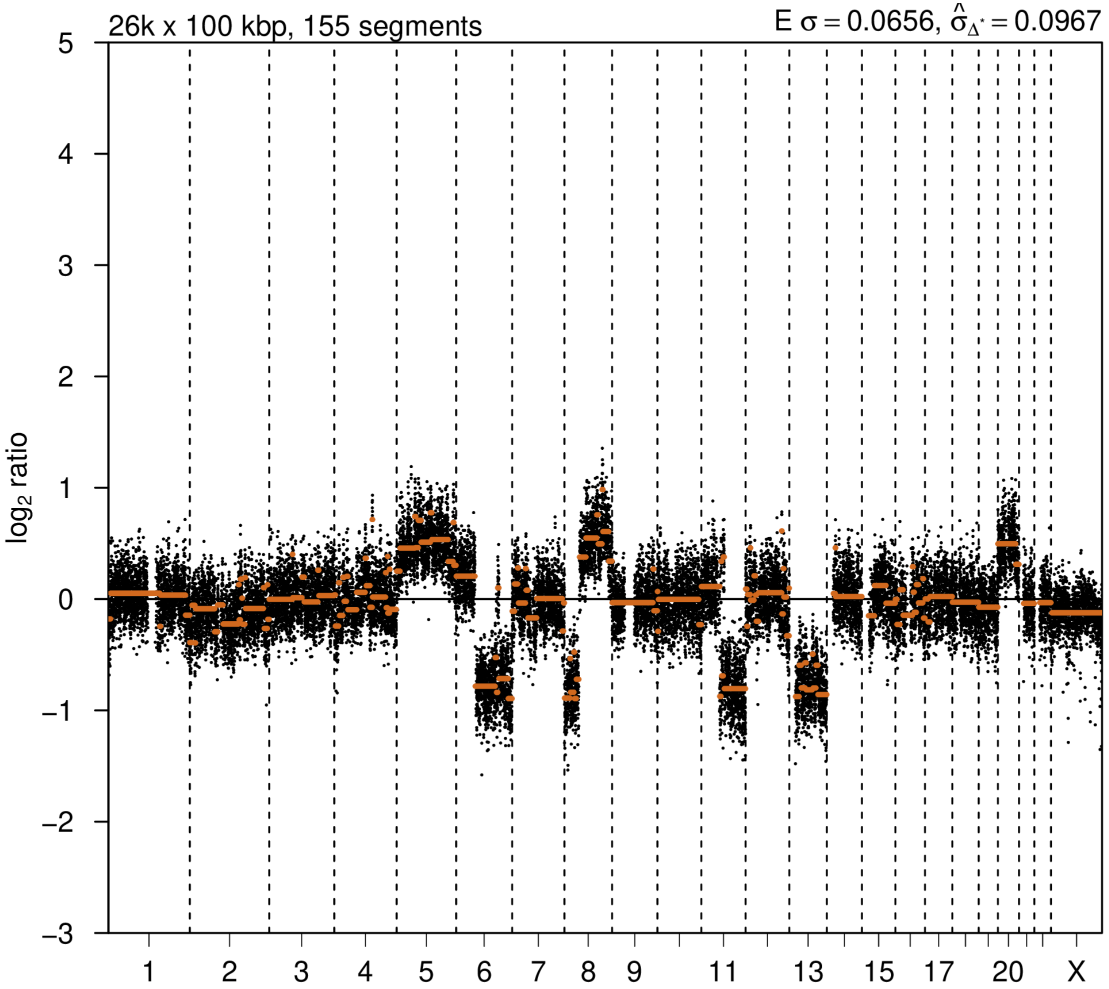

P113

Syn/Meta	Time from 1st surgery to 2nd event (Months)	Side 2nd event	Histology 2nd event	Surgery	AdjuvanT Treatment Pri (RT/ HT)	ER Pri	ER 2nd event	Her2 Pri	Her2 2nd event	Grade Pri	Grade 2nd event	Quadrant 2nd event	Margins	Screening	Clonality P value Copy N	Clonality P value Panel seq	Clonality P value WES	Final verdict
metachronous	173	Ipsilateral	IDC with DCIS	lumpectomy	None	-	+	+	+	2	2	distant from primary	Clear	NA	0.163907822	NA	NA	Unrelated

Primary event

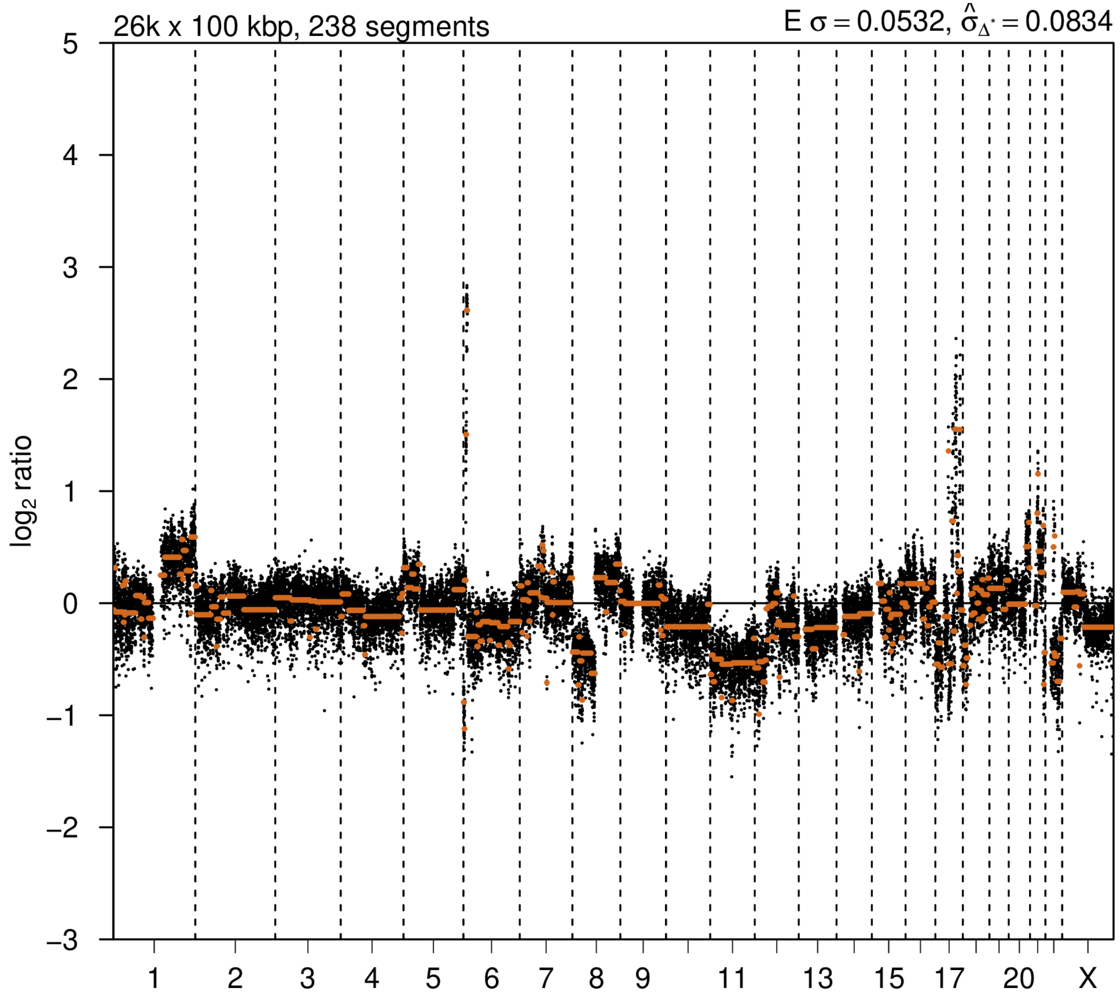

2nd event

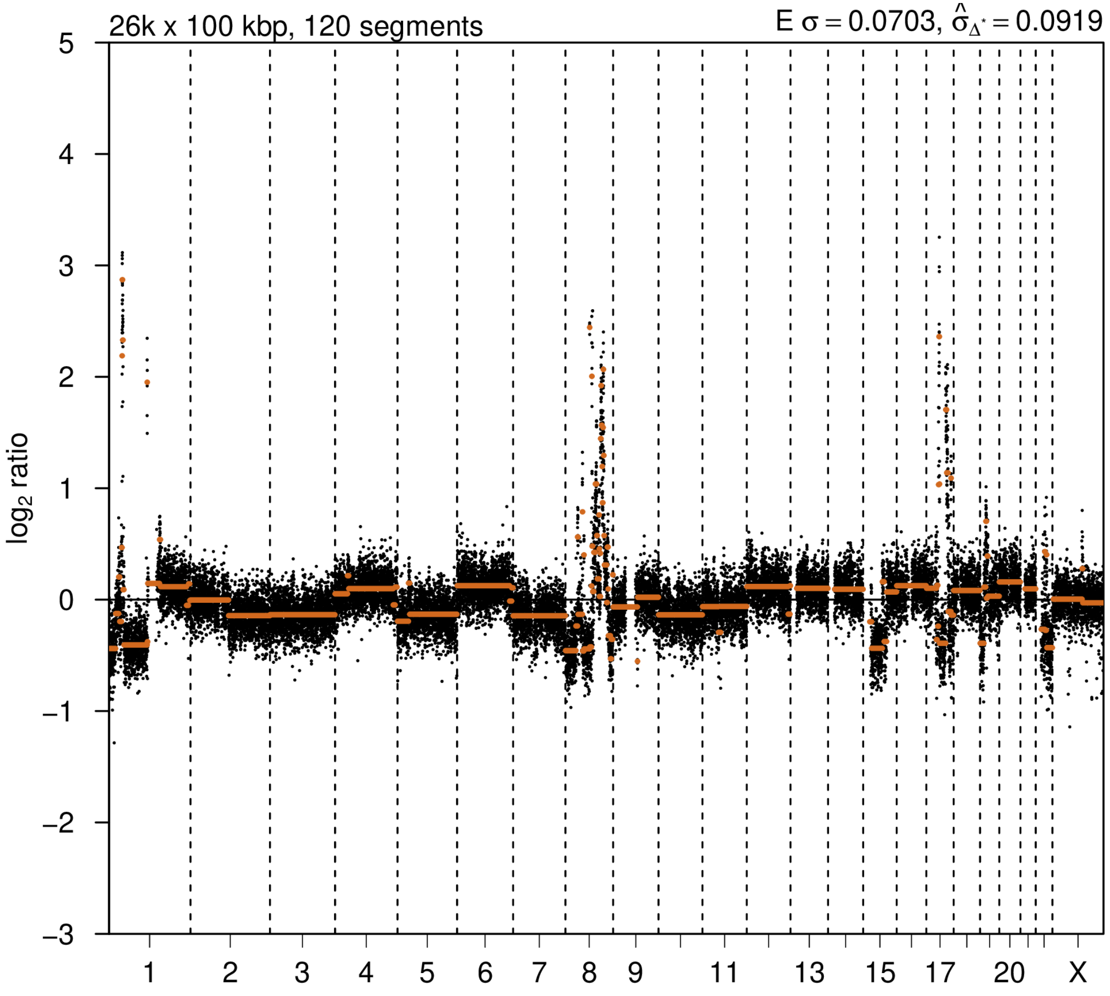

P114

Syn/Meta	Time from 1st surgery to 2nd event (Months)	Side 2nd event	Histology 2nd event	Surgery	AdjuvanT Treatment Pri (RT/ HT)	ER Pri	ER 2nd event	Her2 Pri	Her2 2nd event	Grade Pri	Grade 2nd event	Quadrant 2nd event	Margins	Screening	Clonality P value Copy N	Clonality P value Panel seq	Clonality P value WES	Final verdict
metachronous	165	Ipsilateral	IDC with DCIS	lumpectomy	None	+	+	-	-	2	3	distant from primary	Clear	symptomatic	0.310613437	NA	NA	Unrelated

Primary event

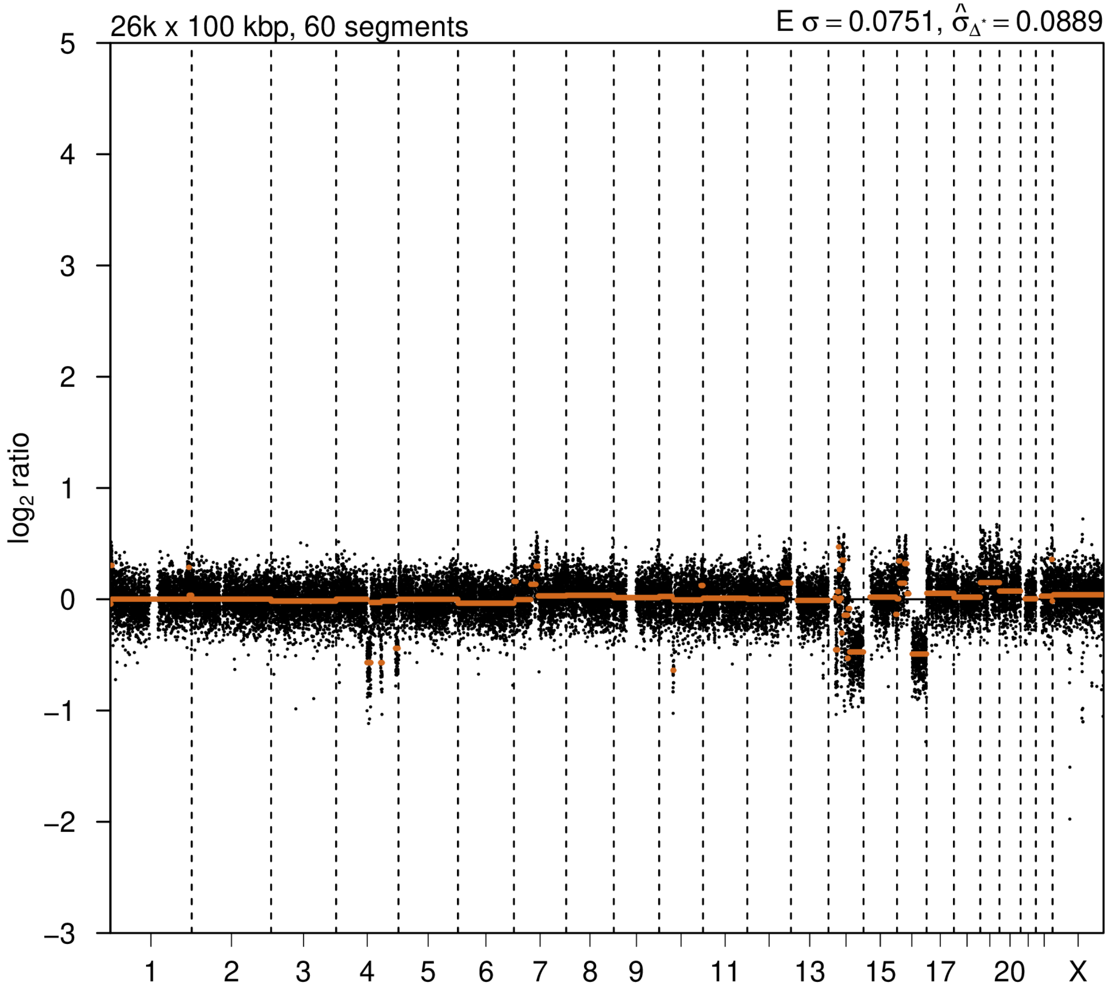

2nd event

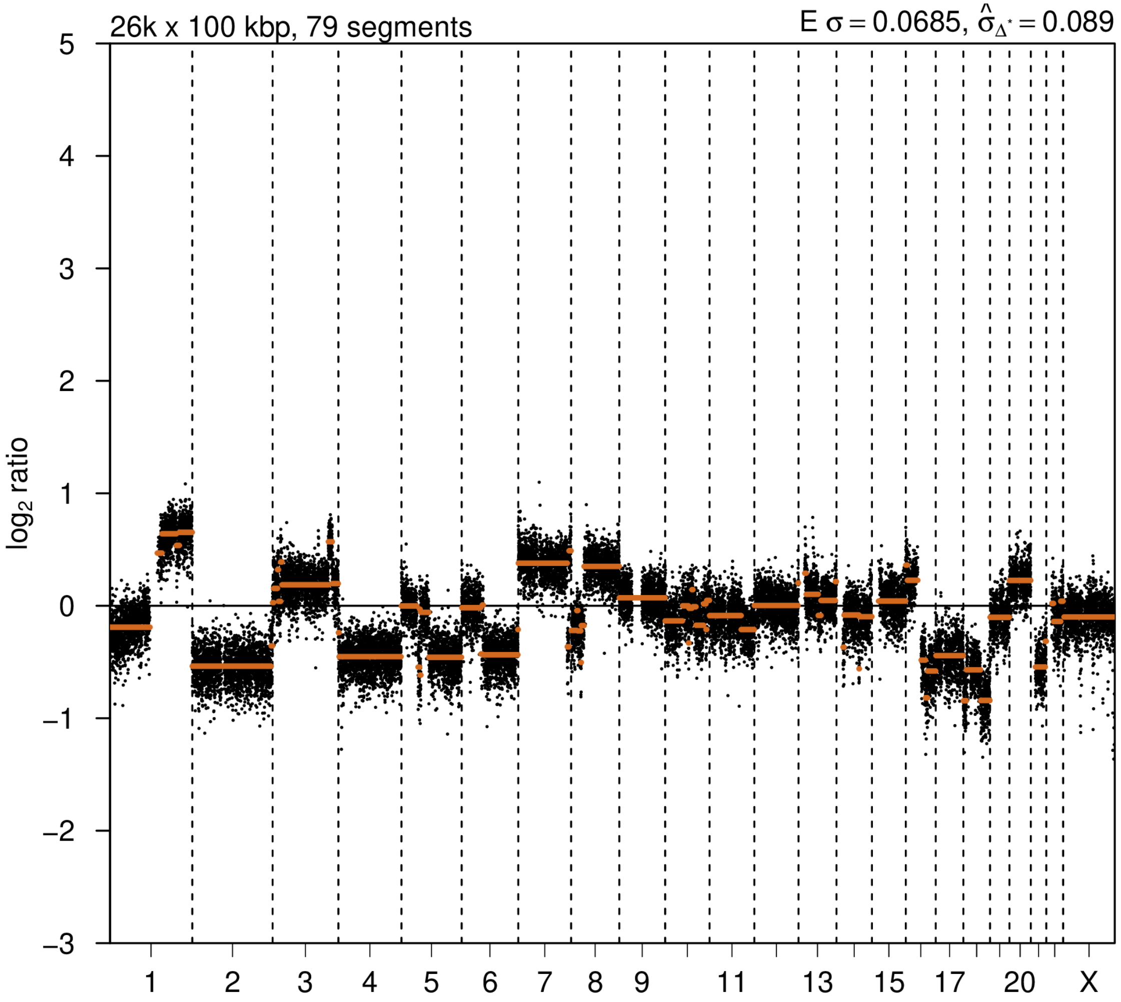

P117

Syn/Meta	Time from 1st surgery to 2nd event (Months)	Side 2nd event	Histology 2nd event	Surgery	AdjuvanT Treatment Pri (RT/ HT)	ER Pri	ER 2nd event	Her2 Pri	Her2 2nd event	Grade Pri	Grade 2nd event	Quadrant 2nd event	Margins	Screening	Clonality P value Copy N	Clonality P value Panel seq	Clonality P value WES	Final verdict
metachronous	105	Ipsilateral	IDC with DCIS	lumpectomy	None	+	-	-	-	2	3	NA	Clear	symptomatic	0.317429406	NA	NA	Unrelated

Primary event

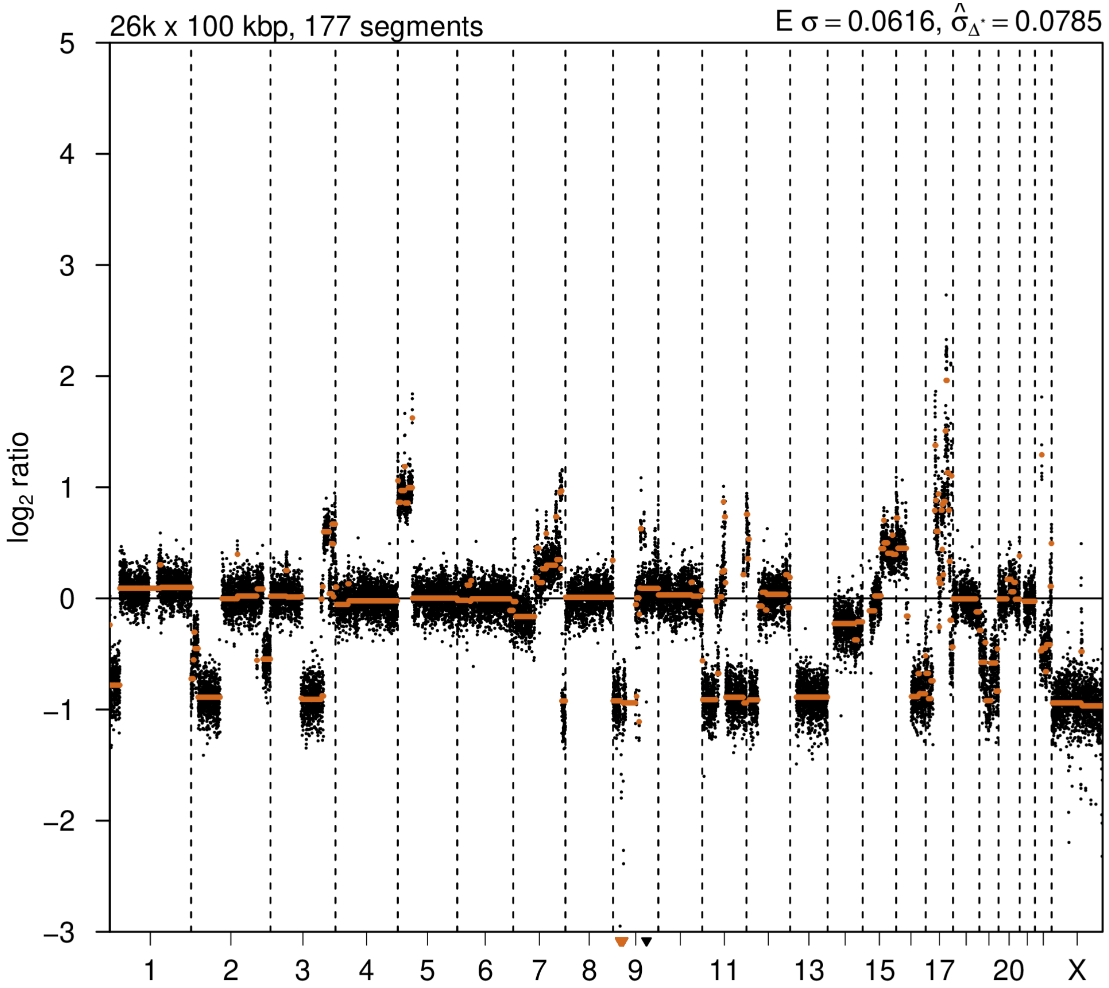

2nd event

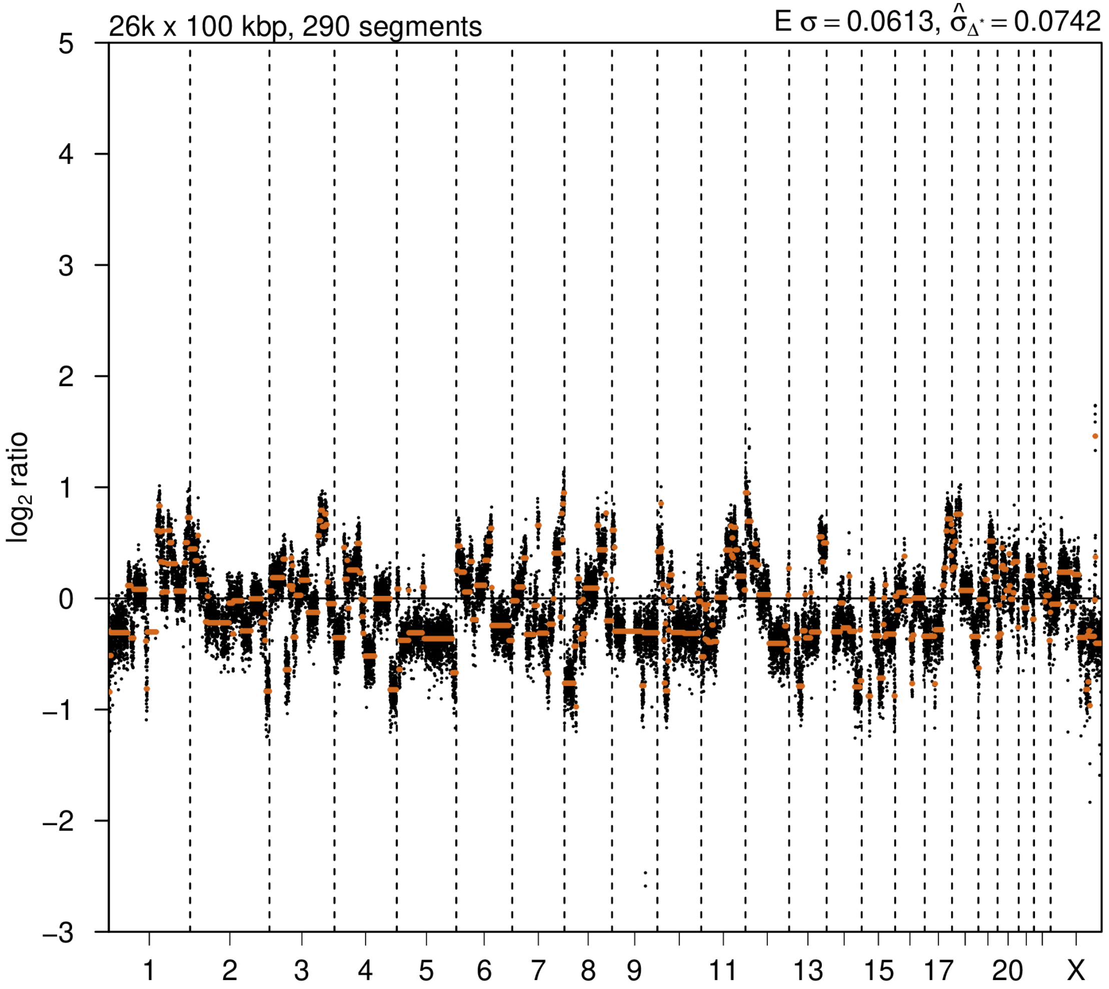

P119

Syn/Meta	Time from 1st surgery to 2nd event (Months)	Side 2nd event	Histology 2nd event	Surgery	AdjuvanT Treatment Pri (RT/ HT)	ER Pri	ER 2nd event	Her2 Pri	Her2 2nd event	Grade Pri	Grade 2nd event	Quadrant 2nd event	Margins	Screening	Clonality P value Copy N	Clonality P value Panel seq	Clonality P value WES	Final verdict
metachronous	66	Ipsilateral	IDC with DCIS	lumpectomy	None	+	-	+	-	2	3	at or adjacent to primary	Clear	screen-detected	0.214540734	NA	NA	Unrelated

Primary event

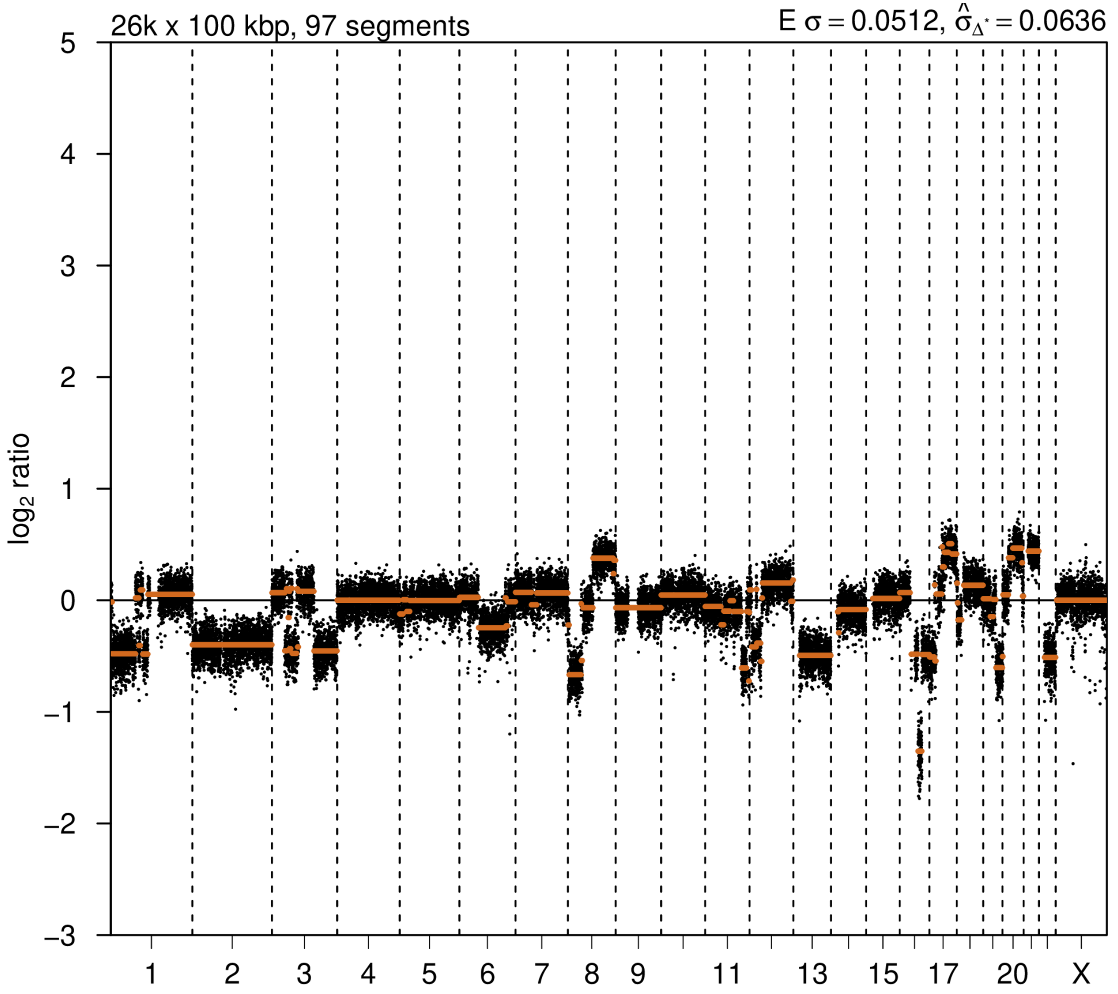

2nd event

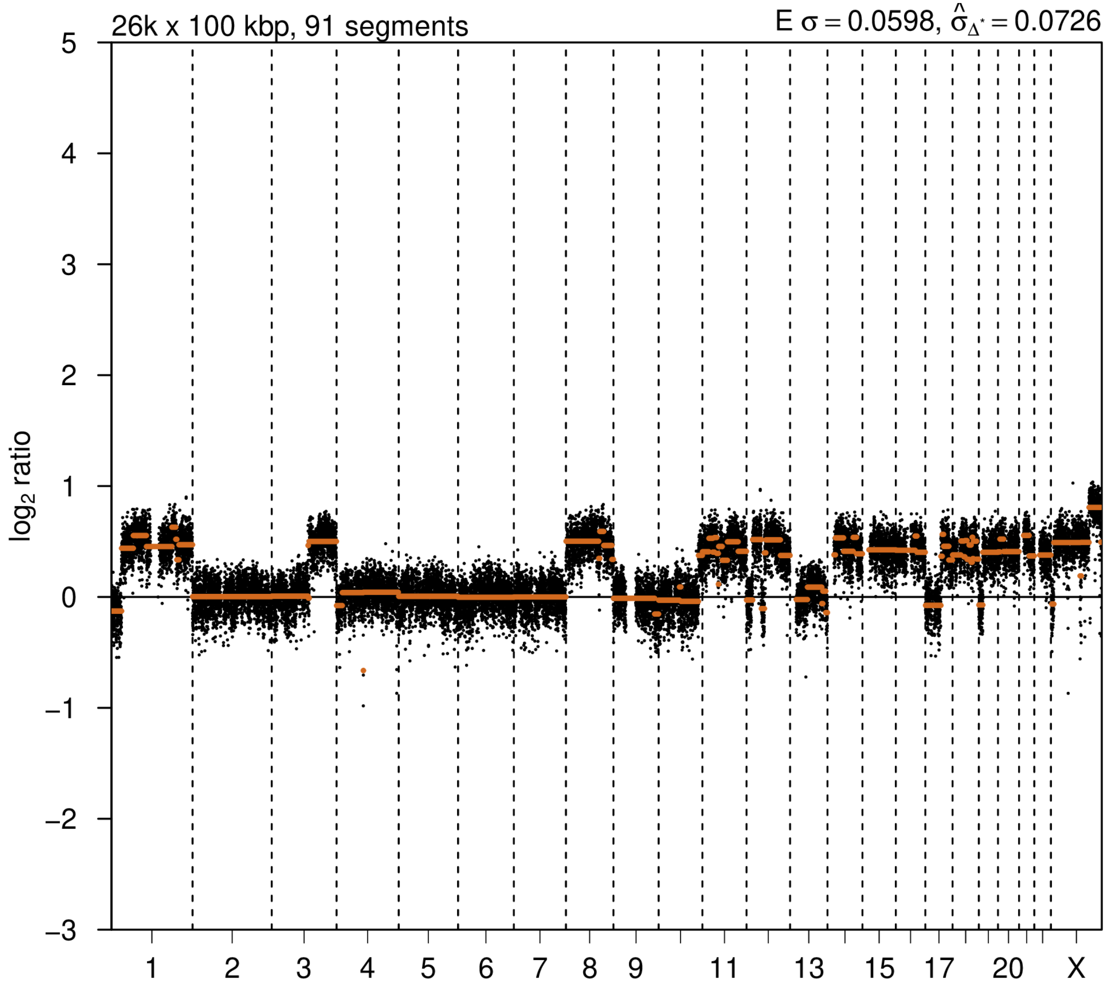

P120

Syn/Meta	Time from 1st surgery to 2nd event (Months)	Side 2nd event	Histology 2nd event	Surgery	AdjuvanT Treatment Pri (RT/ HT)	ER Pri	ER 2nd event	Her2 Pri	Her2 2nd event	Grade Pri	Grade 2nd event	Quadrant 2nd event	Margins	Screening	Clonality P value Copy N	Clonality P value Panel seq	Clonality P value WES	Final verdict
metachronous	64	Ipsilateral	IDC no DCIS	lumpectomy	None	+	+	-	-	1	1	at or adjacent to primary	Clear	screen-detected	0.148003895	NA	NA	Unrelated

Primary event

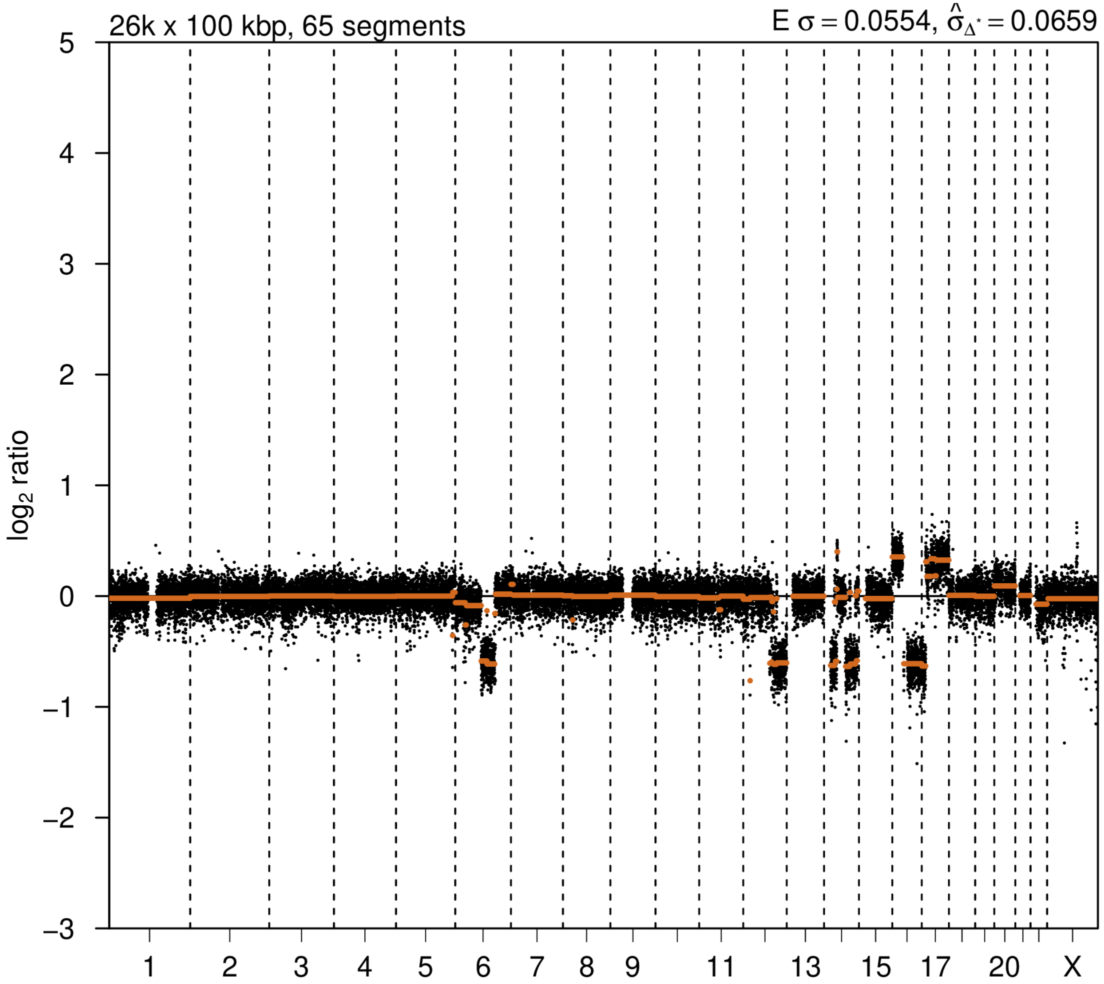

2nd event

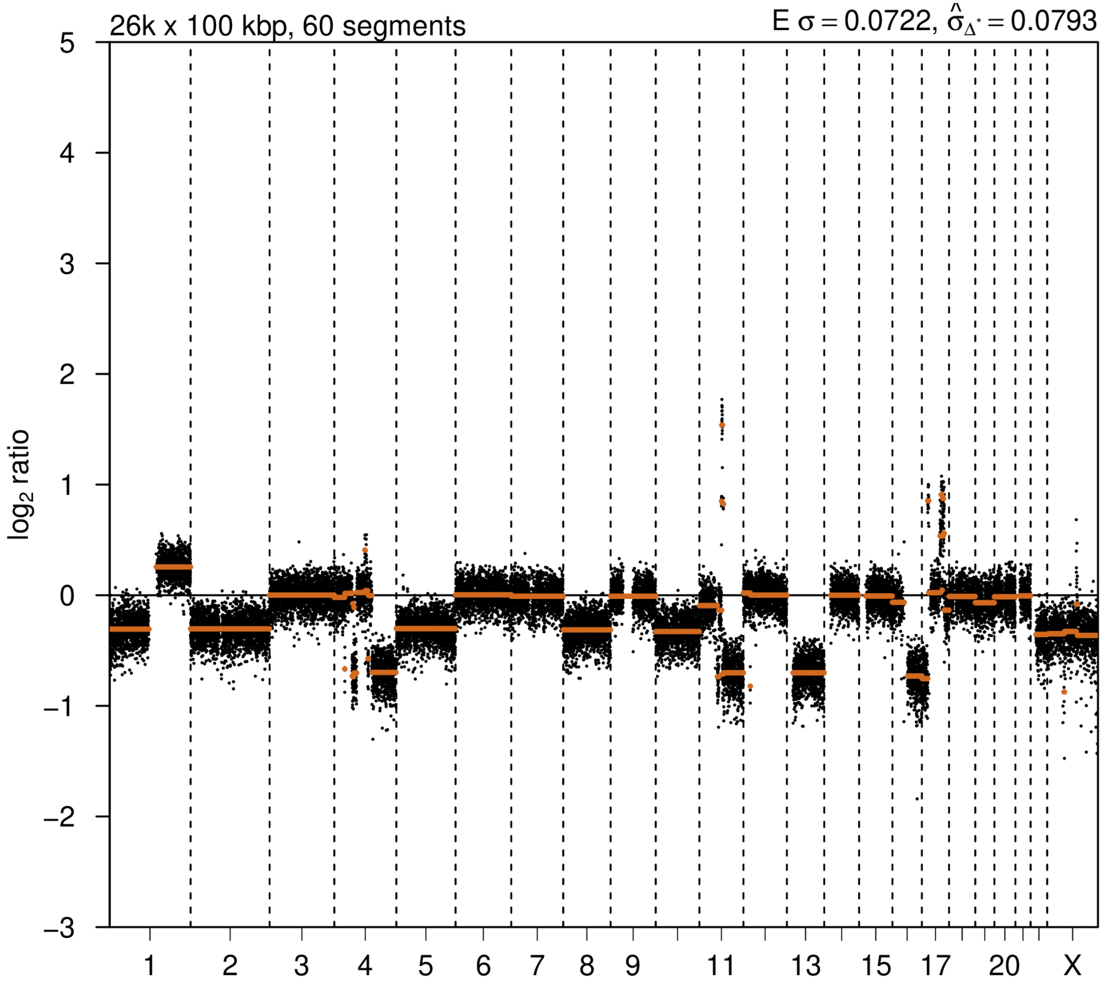

P121

Syn/Meta	Time from 1st surgery to 2nd event (Months)	Side 2nd event	Histology 2nd event	Surgery	AdjuvanT Treatment Pri (RT/ HT)	ER Pri	ER 2nd event	Her2 Pri	Her2 2nd event	Grade Pri	Grade 2nd event	Quadrant 2nd event	Margins	Screening	Clonality P value Copy N	Clonality P value Panel seq	Clonality P value WES	Final verdict
metachronous	85	Ipsilateral	IDC no DCIS	lumpectomy	None	+	-	-	+	1	2	at or adjacent to primary	involved	screen-detected	0.804933 463	1	NA	Unrelated

Primary event

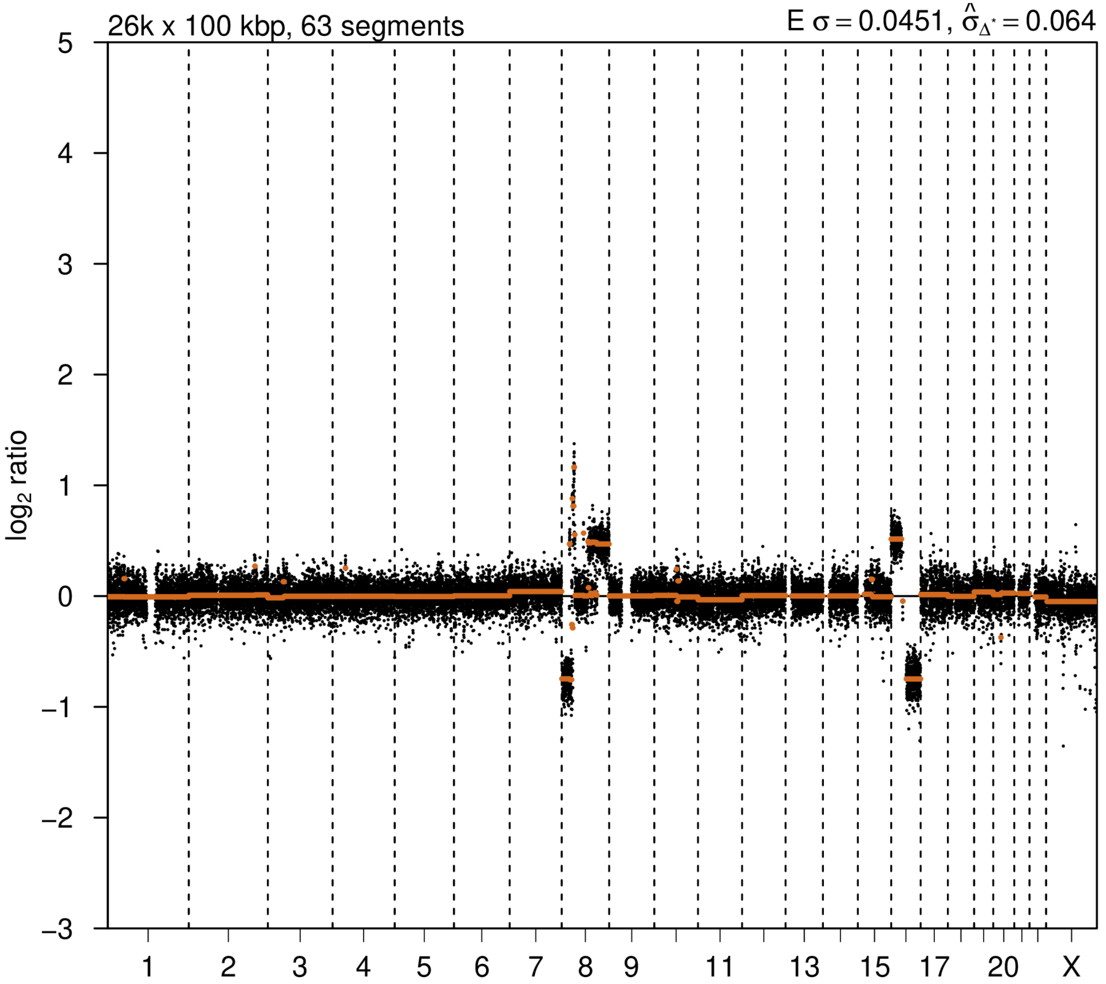

2nd event

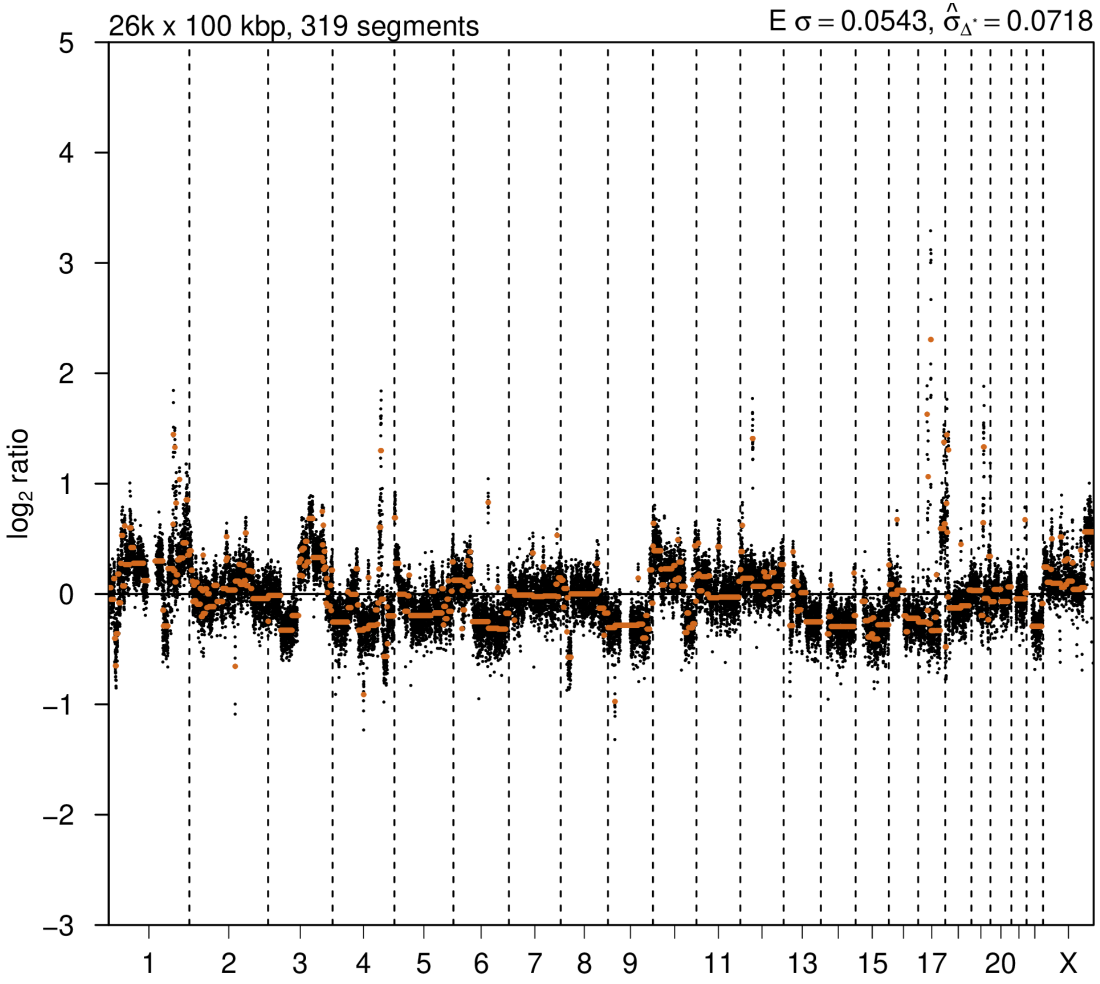

P123

Syn/Meta	Time from 1st surgery to 2nd event (Months)	Side 2nd event	Histology 2nd event	Surgery	AdjuvanT Treatment Pri (RT/ HT)	ER Pri	ER 2nd event	Her2 Pri	Her2 2nd event	Grade Pri	Grade 2nd event	Quadrant 2nd event	Margins	Screening	Clonality P value Copy N	Clonality P value Panel seq	Clonality P value WES	Final verdict
metachronous	36	Ipsilateral	IDC no DCIS	lumpectomy	None	+	+	-	-	1	3	distant from primary	Clear	screen-detected	0.331061344	NA	NA	Unrelated

Primary event

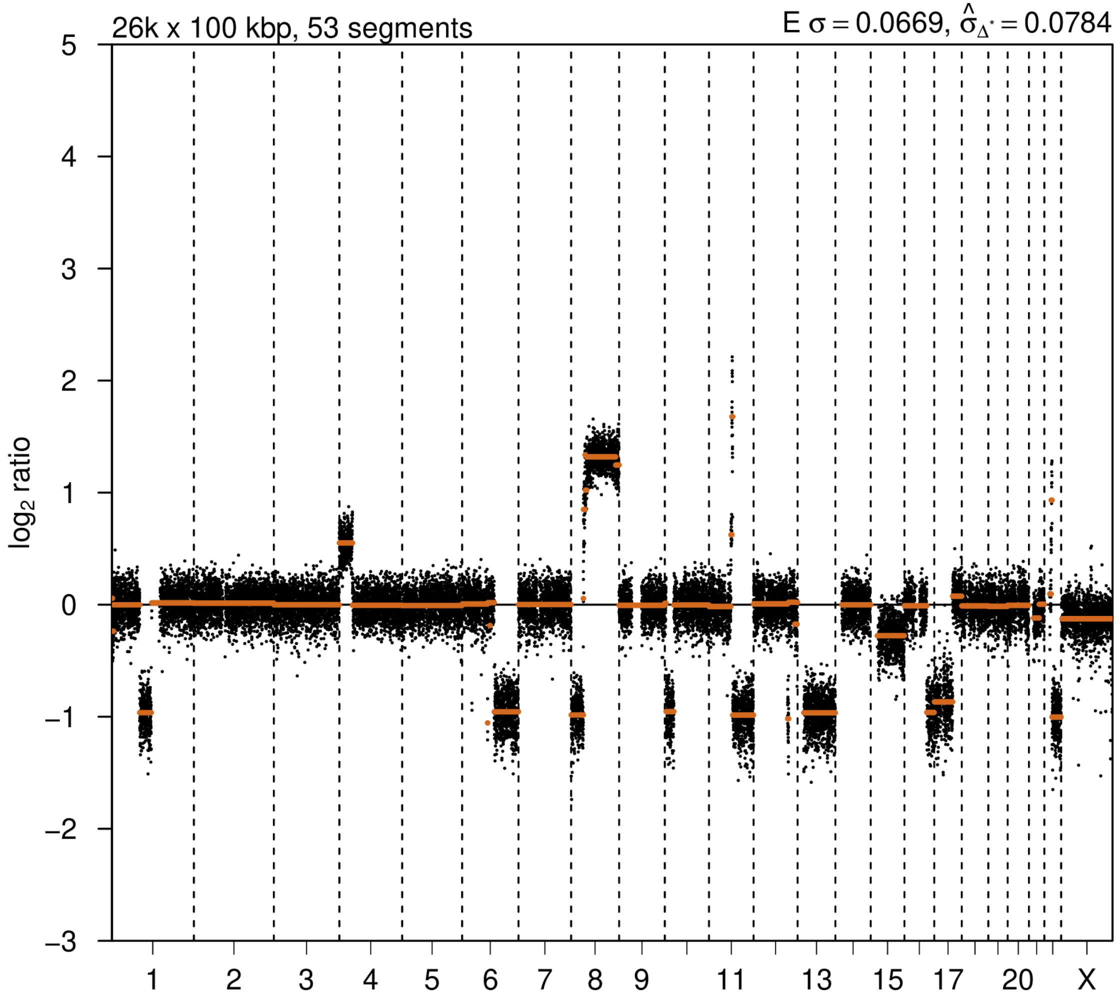

2nd event

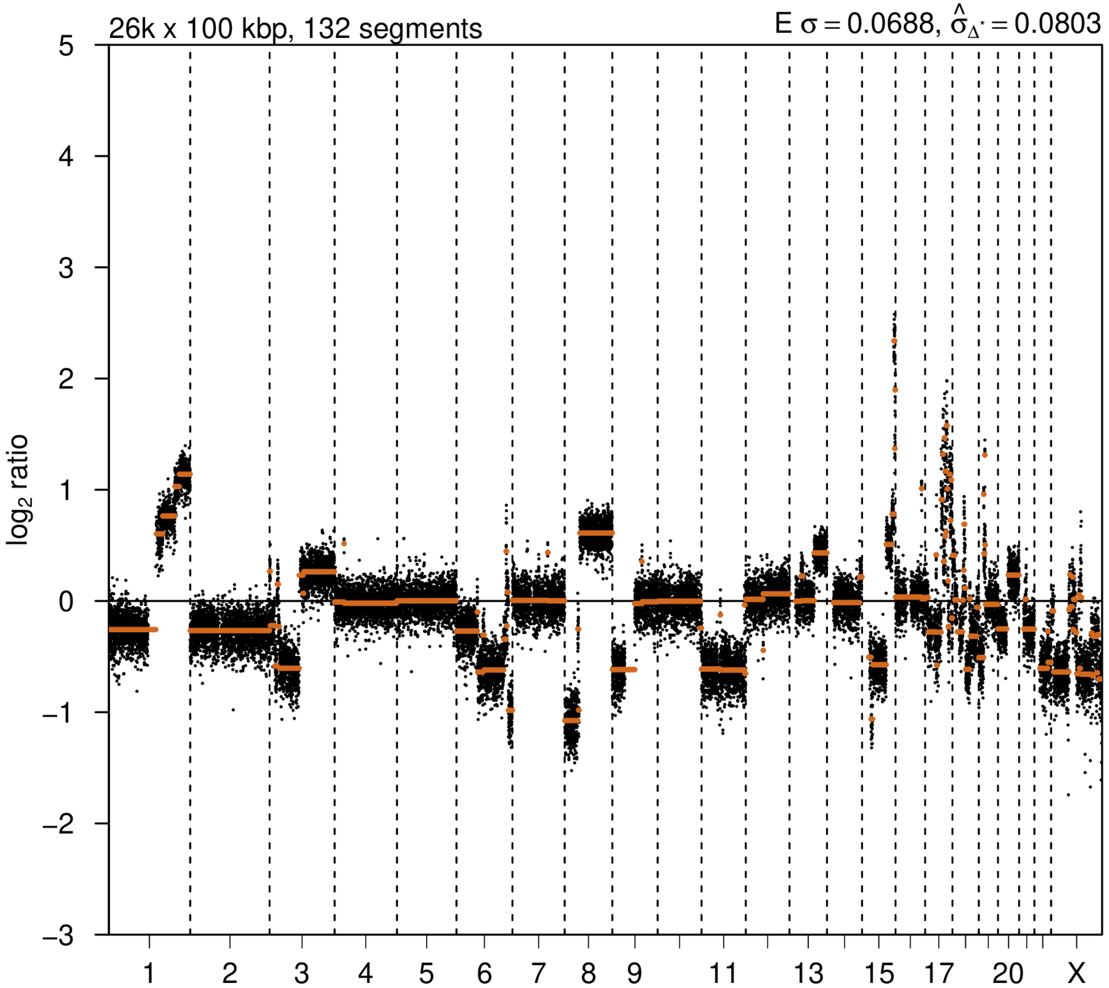

P125

Syn/Meta	Time from 1st surgery to 2nd event (Months)	Side 2nd event	Histology 2nd event	Surgery	AdjuvanT Treatment Pri (RT/ HT)	ER Pri	ER 2nd event	Her2 Pri	Her2 2nd event	Grade Pri	Grade 2nd event	Quadrant 2nd event	Margins	Screening	Clonality P value Copy N	Clonality P value Panel seq	Clonality P value WES	Final verdict
metachronous	52	Ipsilateral	IDC with DCIS	lumpectomy	None	+	+	-	-	2	1	NA	Clear	symptomatic	0.64459591	1	NA	Unrelated

Primary event

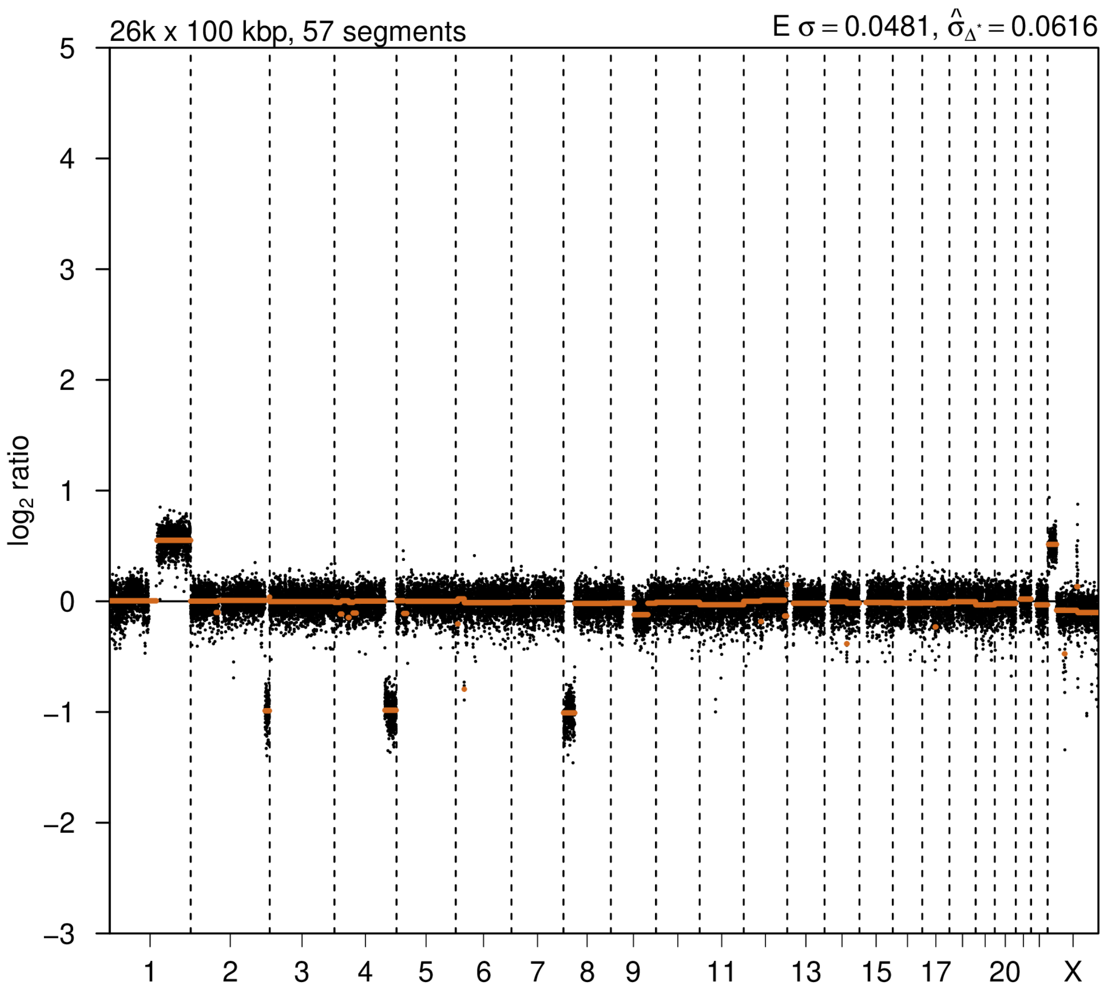

2nd event

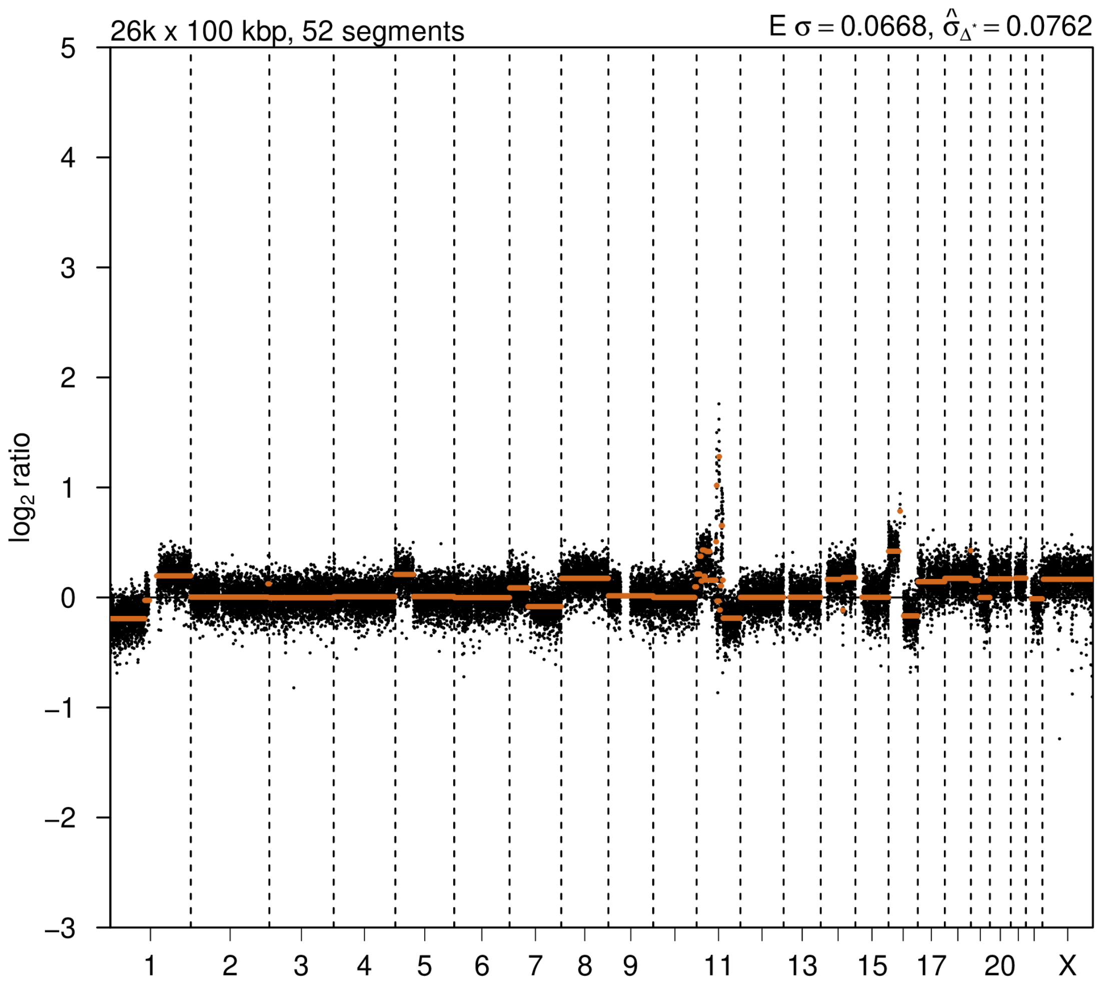

P126

Syn/Meta	Time from 1st surgery to 2nd event (Months)	Side 2nd event	Histology 2nd event	Surgery	AdjuvanT Treatment Pri (RT/ HT)	ER Pri	ER 2nd event	Her2 Pri	Her2 2nd event	Grade Pri	Grade 2nd event	Quadrant 2nd event	Margins	Screening	Clonality P value Copy N	Clonality P value Panel seq	Clonality P value WES	Final verdict
metachronous	55	Ipsilateral	IDC with DCIS	lumpectomy	None	-	+	+	-	3	3	at or adjacent to primary	Clear	screen-detected	0.931191 172	NA	NA	Unrelated

Primary event

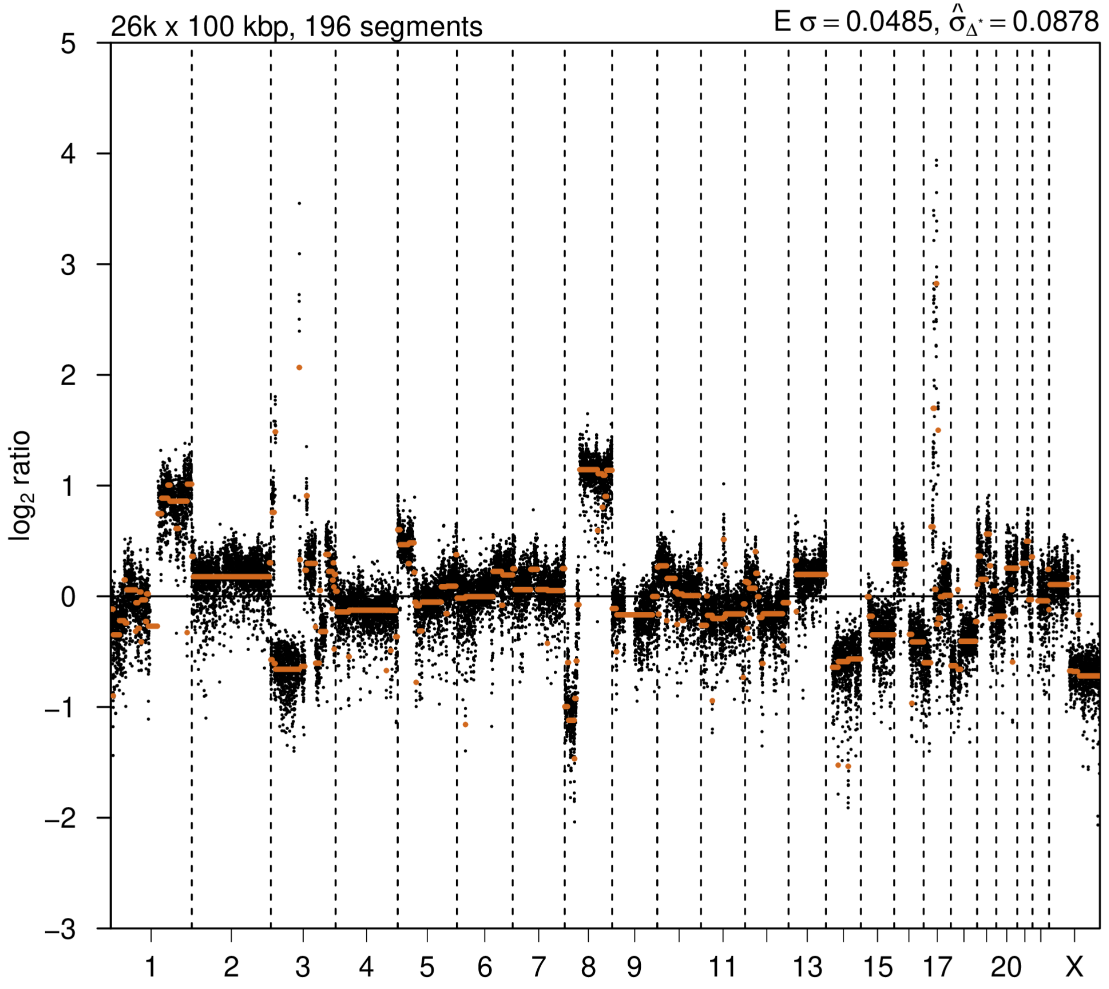

2nd event

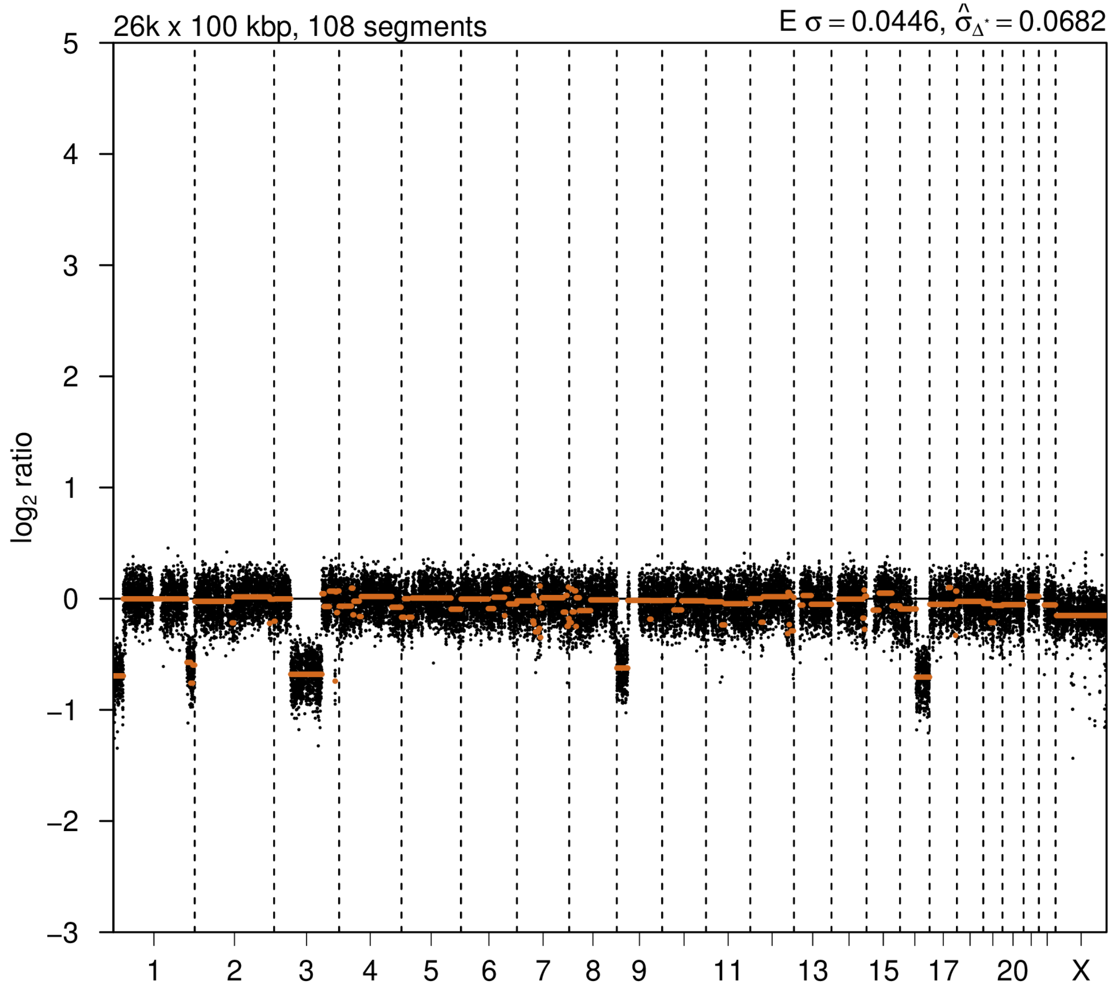

P129

Syn/Meta	Time from 1st surgery to 2nd event (Months)	Side 2nd event	Histology 2nd event	Surgery	AdjuvanT Treatment Pri (RT/ HT)	ER Pri	ER 2nd event	Her2 Pri	Her2 2nd event	Grade Pri	Grade 2nd event	Quadrant 2nd event	Margins	Screening	Clonality P value Copy N	Clonality P value Panel seq	Clonality P value WES	Final verdict
metachronous	23	Ipsilateral	IDC with DCIS	lumpectomy	None	-	-	+	-	3	2	NA	Clear	screen-detected	0.581953911	1	1	Unrelated

Primary event

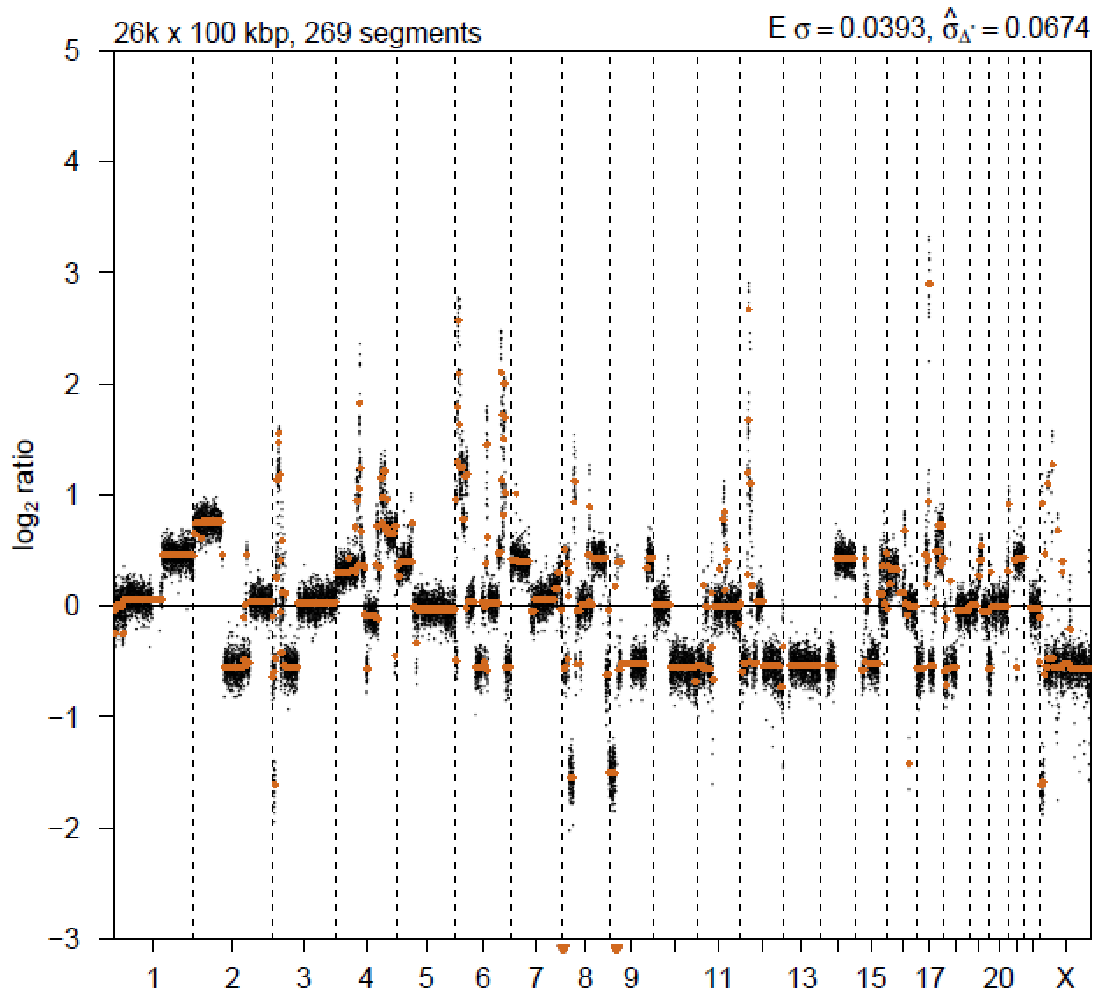

2nd event

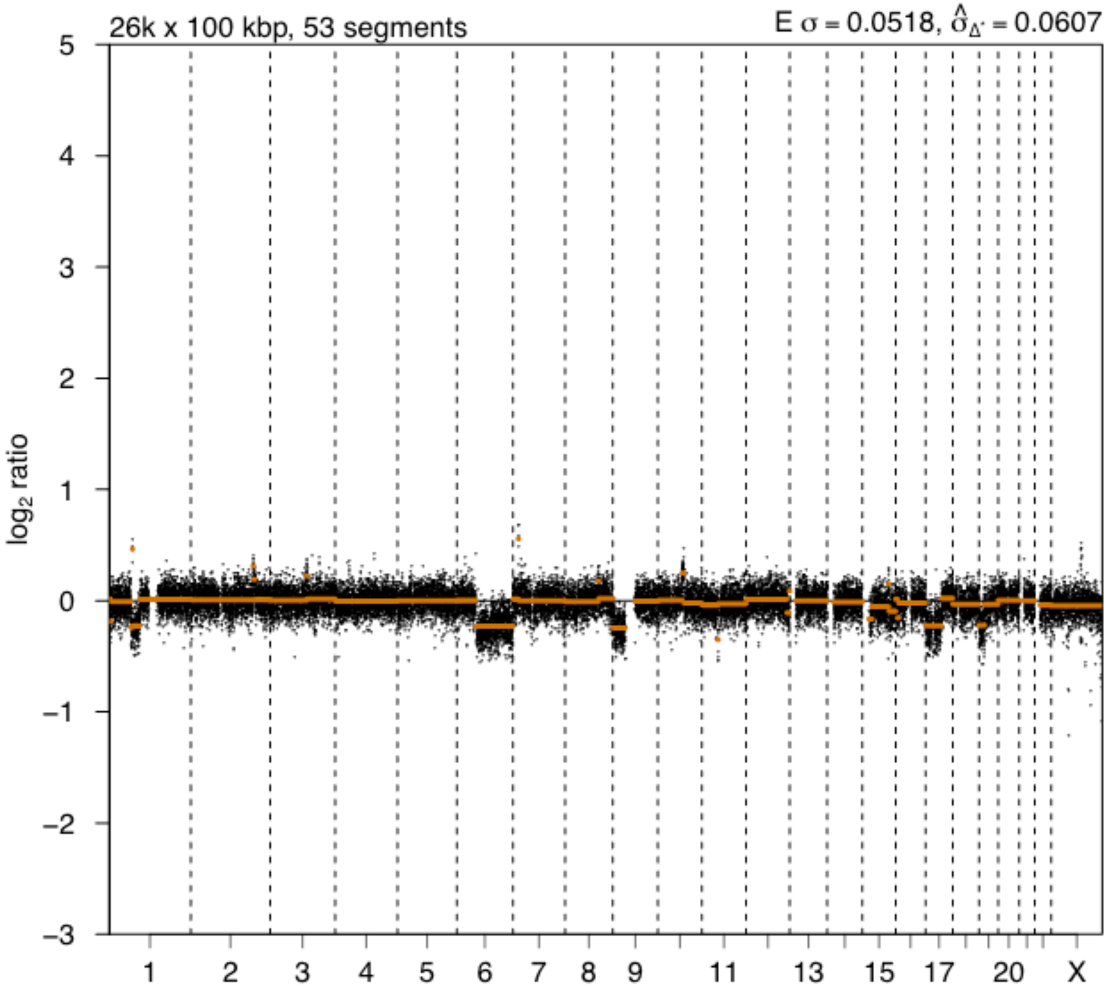

Supplementary Note 1: PanelSeq performance and filtering strategy for NKI samples

Performance PanelSeq

Figure SF1 and SF2 show a representative example of the performance of the custom 53-gene panel. The graphs contain the data of 40 DCIS samples.

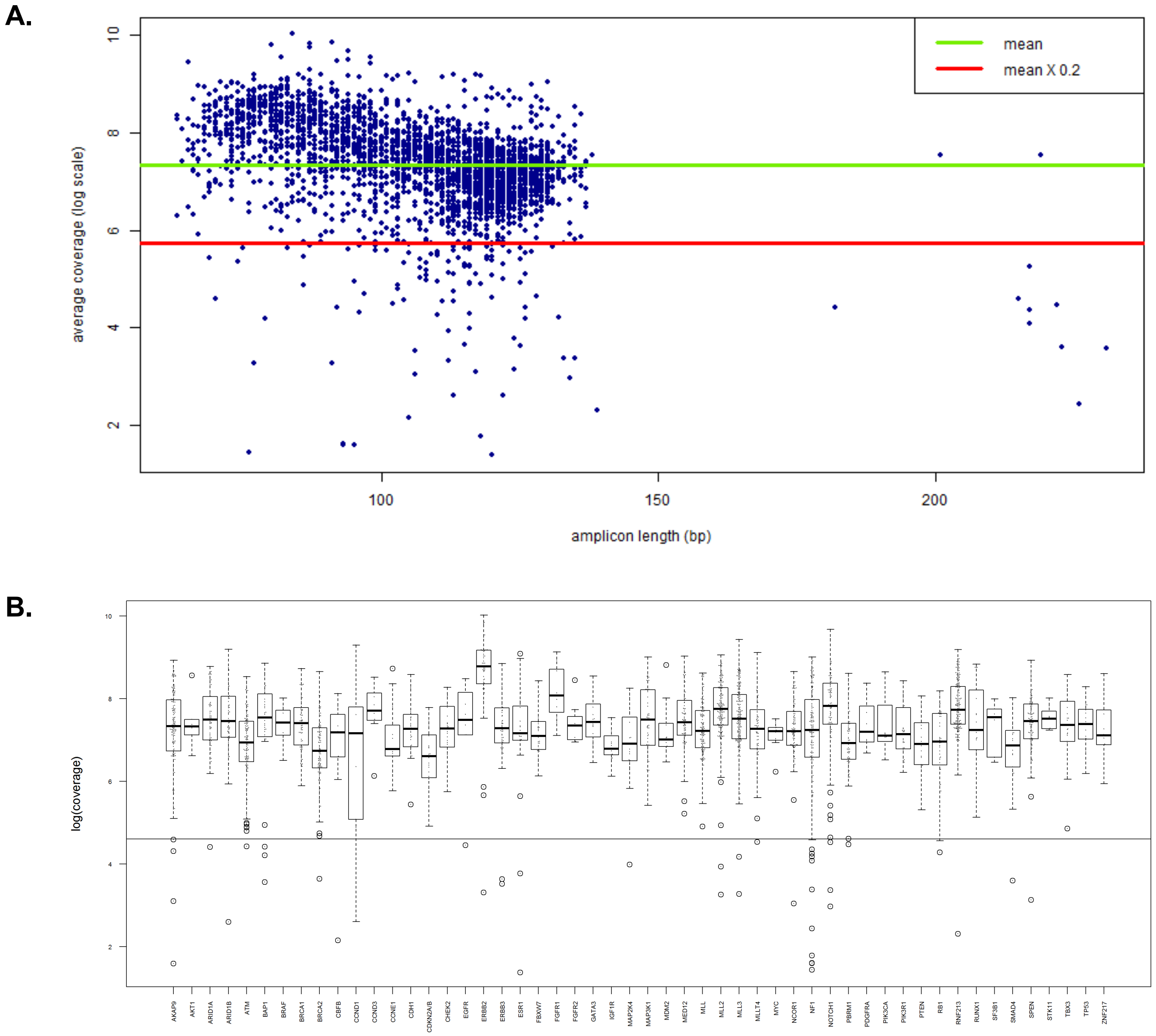

Figure SF1. Performance of the custom 53-gene panel in n=40 DCIS samples. **A.** Amplicon coverage by amplicon length; **B.** Amplicon coverage per gene. Black line represents a coverage of 100X. Average coverage >2000X.

Filtering strategy PanelSeq data

To narrow down the IonTorrent sequencing data to the true somatic variants found in the DCIS and IBC samples, the following steps were taken. First, we included variants with a variant allele frequency >5%, coverage >100x, and a quality (QUAL) of >1000 (variants of QUAL <1000 were mostly low frequency variants, see Figure SF2A). Variants not found in GNOMAD and GoNL and found in somatic ClinVar were included. Figure SF2B). Variants found in <90% of the samples and located within the coding sequence of the genome were included. All included somatic variants were merged to one variant list. This list of variants was subjected to a final check via manual assessment in IGV. Lastly, the positions of variants called solely in the IBC lesions were checked in the matching DCIS lesion and vice versa, to reassure the absence of this variant in the paired sample.

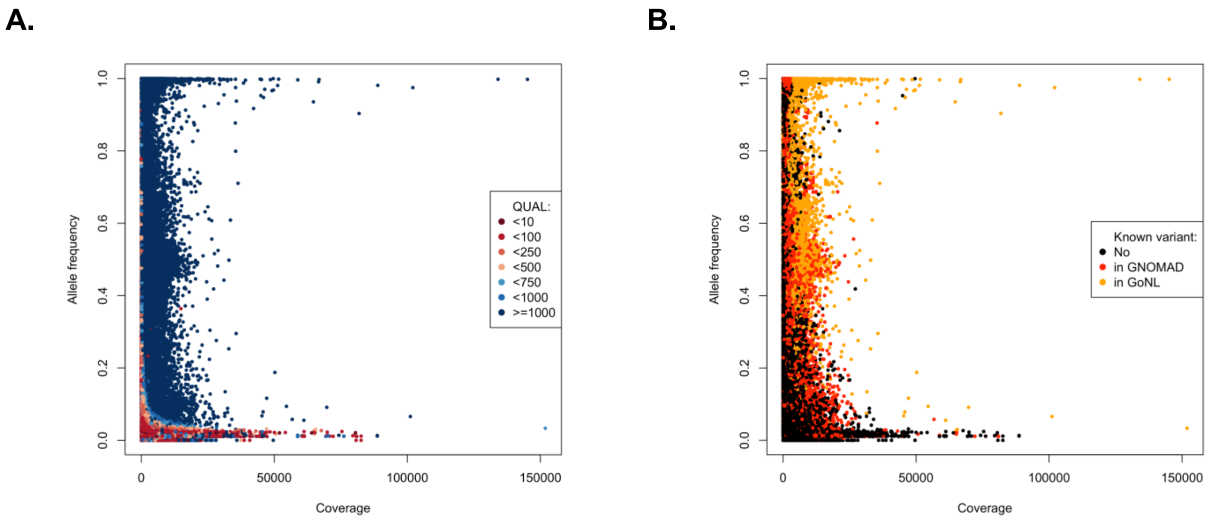

Figure SF2. Visualization of variants selected for exclusion in n=40 DCIS samples. **A.** Variants with a quality (QUAL) of <1000 were excluded (all colors except dark blue). **B.** Variants found in in GNOMAD (red) and GoNL (orange) were excluded.

Supplementary Note 2: FACS Methodology

Sample preparation - FFPE Tissue Dissociation Kit from MACS (Cat#130-118-052)

Instrument - BD FACSMelody

Software - BD FACSChorus software

Cell population abundance -DAPI counts vs DAPI area plots were used to determine relative population abundance

Gating strategy - DAPI (area) fluorescence intensities were used to determine which nuclei populations were flow-sorted. We gate from 2N peaks and from > 2N peaks to enrich tumor cells based on DAPI fluorescent signal as demonstrated below.

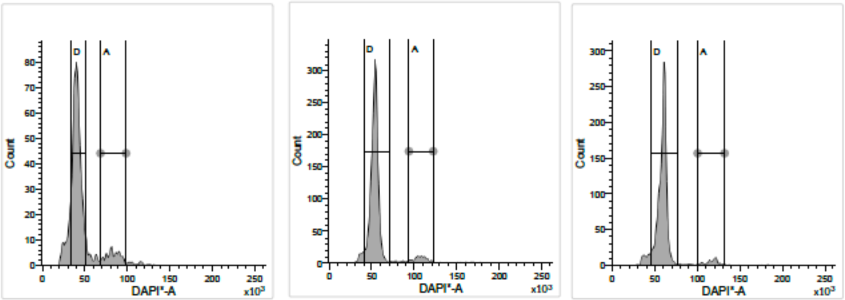

Supplementary Note 3: Comparison of the different clonality algorithms

In order to assess the added usefulness of our method, we applied the relevant copy number and mutation-based functions in the previously published Clonality package[12,13] to our samples where copy number was ascertained by SNP array (see figure below). We observed with the estimate of the number of clonally related pairs was lower for the clonality package compared to our proposed method, as well as suggesting a number of the contralateral recurrences were clonally related. Visual inspection of the contralateral samples that were called clonal showed that they were generally genomically stable samples, with few major aberrations. When those samples do share aberrations on the same chromosomal arms, they are considered clonally related by the Clonality package which, by design, relies on fewer events, but not by our method which relies on the presence of multiple copy number events.

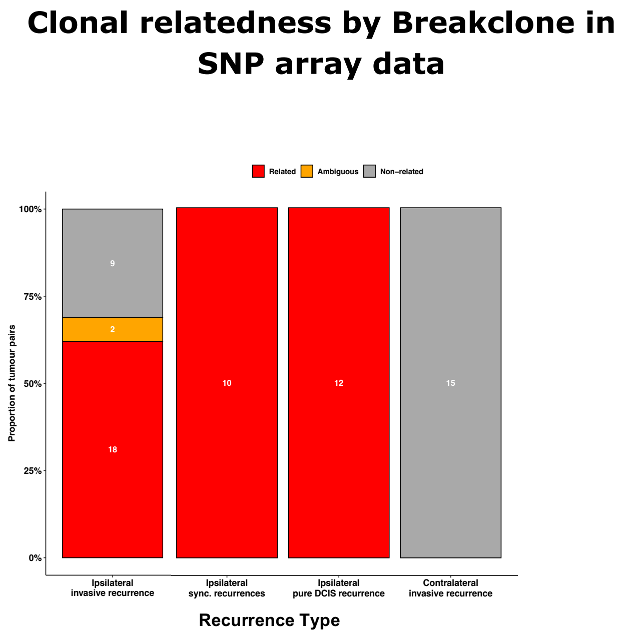

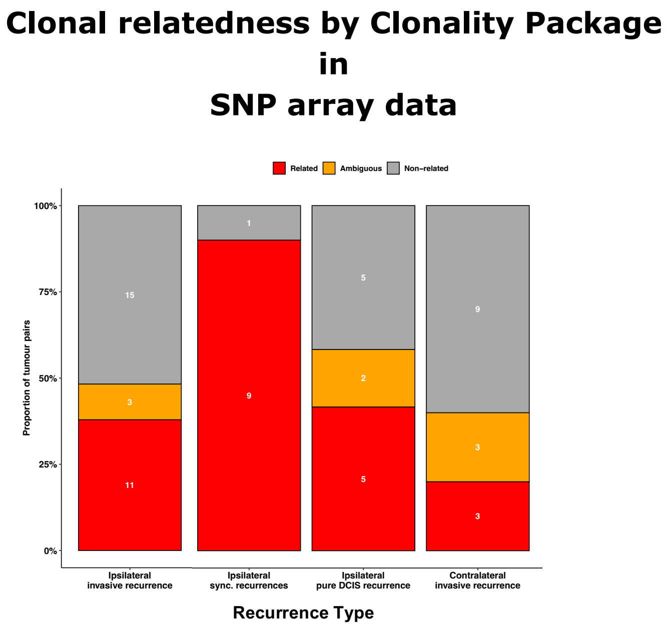

Supplement: Supplementary file 1 — Supplementary Fig. 1 and Notes 1–3. [file 41588_2022_1082_MOESM1_ESM.pdf]
