# Supplementary material for: Genomic analysis defines clonal relationships of ductal carcinoma in situ and recurrent invasive breast cancer
Source: Nat Genet. 2022 Jun 9;54(6):850–60. doi: 10.1038/s41588-022-01082-3 (PMC9197769; doi:10.1038/s41588-022-01082-3)
Supplement: Supplementary file 2 — Reporting Summary [file 41588_2022_1082_MOESM2_ESM.pdf]

nature portfolio

Corresponding author(s): Elinor Sawyer
Last updated by author(s): Jan 15 2022

Reporting Summary

Nature Portfolio wishes to improve the reproducibility of the work that we publish. This form provides structure for consistency and transparency in reporting. For further information on Nature Portfolio policies, see our Editorial Policies and the Editorial Policy Checklist.

Statistics

For all statistical analyses, confirm that the following items are present in the figure legend, table legend, main text, or Methods section.

n/a	Confirmed	
☐	☒	The exact sample size (*n*) for each experimental group/condition, given as a discrete number and unit of measurement
☐	☒	A statement on whether measurements were taken from distinct samples or whether the same sample was measured repeatedly
☐	☒	The statistical test(s) used AND whether they are one- or two-sided<br>*Only common tests should be described solely by name; describe more complex techniques in the Methods section.*
☐	☒	A description of all covariates tested
☐	☒	A description of any assumptions or corrections, such as tests of normality and adjustment for multiple comparisons
☐	☒	A full description of the statistical parameters including central tendency (e.g. means) or other basic estimates (e.g. regression coefficient) AND variation (e.g. standard deviation) or associated estimates of uncertainty (e.g. confidence intervals)
☐	☒	For null hypothesis testing, the test statistic (e.g. *F*, *t*, *r*) with confidence intervals, effect sizes, degrees of freedom and *P* value noted<br>*Give P values as exact values whenever suitable.*
☒	☐	For Bayesian analysis, information on the choice of priors and Markov chain Monte Carlo settings
☒	☐	For hierarchical and complex designs, identification of the appropriate level for tests and full reporting of outcomes
☐	☒	Estimates of effect sizes (e.g. Cohen's *d*, Pearson's *r*), indicating how they were calculated

Our web collection on statistics for biologists contains articles on many of the points above.

Software and code

Policy information about availability of computer code

Data collection	A detailed description of the data collection has been provided in the methods section. All associated analyses have been reported on github: https://github.com/argymeg/precision-clonality-code.<br>Single cel sequencing code can be found at: https://github.com/navinlabcode/PRECISION_clonality_sc<br>The following datasets were used to generate the data in the manuscript:<br>TCGA Pan-Cancer Atlas breast cancer mutation calls (https://www.cancer.gov/about-nci/organization/ccg/research/structural-genomics/tcga)<br>hg19 (NCBI Build 37) (https://www.ncbi.nlm.nih.gov/assembly/GCF_000001405.13/_)<br>GRCh38 (hg38) (https://www.ncbi.nlm.nih.gov/assembly/GCF_000001405.26/)
Data analysis	A detailed description of the data analyses methods has been provided in the methods section. All associated analyses have been reported on github: https://github.com/argymeg/precision-clonality-code<br>The following data analysis packages were used to generate the data in the manuscript: PLINK v1.07, BWA 0.7.17 , Samtools 1.9, Picard 2.18.3, QDNAseq 1.22.0, CGHcall 2.48.0, BWA-MEM, MuTect2 from the Genome Analysis Toolkit (version 4.1.0.0), Torrent Variant Caller (TVC) version 5.6, bcftools, VEP, SAMtools (0.1.16), UMAP, bowtie2 (2.1.0) , R Bioconductor multipcf package, R package 'uwot' v0.1.8, R Bioconductor package scran (v1.14.6), R package 'dbscan' v1.1-5, R package ComplexHeatmap v2.2.0, Breakclone, Clonality package.

For manuscripts utilizing custom algorithms or software that are central to the research but not yet described in published literature, software must be made available to editors and reviewers. We strongly encourage code deposition in a community repository (e.g. GitHub). See the Nature Portfolio guidelines for submitting code & software for further information.

Data

Policy information about availability of data

All manuscripts must include a data availability statement. This statement should provide the following information, where applicable:

- Accession codes, unique identifiers, or web links for publicly available datasets
- A description of any restrictions on data availability
- For clinical datasets or third party data, please ensure that the statement adheres to our policy

Sequence data has been deposited at the European Genome-phenome Archive (EGA), which is hosted by the EBI and the CRG, under accession number EGAS00001005784 and can be accessed once a data sharing agreement had been signed. Contact details for the Access Committee are available on the EGA webpage.
Further information about EGA can be found on https://ega-archive.org "The European Genome-phenome Archive of human data consented for biomedical research"(http://www.nature.com/ng/journal/v47/n7/full/ng.3312.html).
A detailed description of the data collection has been provided in the methods section. All associated analyses have been reported on github: https://github.com/argymeg/precision-clonality-code
The following datasets were used to generate the data in the manuscript:
TCGA Pan-Cancer Atlas breast cancer mutation calls (https://www.cancer.gov/about-nci/organization/ccg/research/structural-genomics/tcga)
hg19 (NCBI Build 37) (https://www.ncbi.nlm.nih.gov/assembly/GCF_000001405.13/_)
GRCh38 (hg38) (https://www.ncbi.nlm.nih.gov/assembly/GCF_000001405.26/)

Field-specific reporting

Please select the one below that is the best fit for your research. If you are not sure, read the appropriate sections before making your selection.

☒ Life sciences ☐ Behavioural & social sciences ☐ Ecological, evolutionary & environmental sciences

For a reference copy of the document with all sections, see nature.com/documents/nr-reporting-summary-flat.pdf

Life sciences study design

All studies must disclose on these points even when the disclosure is negative.

Sample size	The research question we addressed ('What percentage of primary DCIS is clonally related to invasive recurrences?') has never been addressed on a large scale before. Therefore, we collected all available paired samples from three large cohorts. As invasive recurrences after DCIS are rare, long follow up time is needed, old FFPE materials have poor DNA quality, and the amount of DCIS tissue can be limited, it was very difficult to find suitable sample pairs for deep genomic characterization. By combining three international cohorts, we have the largest sample series to date and the best available sample series to answer our research question.
Data exclusions	Samples were excluded due to the following reasons:<br>- Only one sample of a pair was available<br>- DNA quantity was not sufficient<br>- QC analyses for either WES, CN or panel seq failed
Replication	We combined four different methods to assess clonality: WES, panel seq, CN analyses and single cell sequencing. All methods showed high concordance, and we are therefore convinced that our data is robust.
Randomization	Randomization was not applied here, as there was no intervention in this study. Outcome was already known and all samples were genomically profiled to assess clonality.
Blinding	Blinding was not necessary for data collection as we only collected samples with the same outcome - all had recurred). The lab technicians and the data analysts were blinded to the clonality score of the different technologies used.

Reporting for specific materials, systems and methods

We require information from authors about some types of materials, experimental systems and methods used in many studies. Here, indicate whether each material, system or method listed is relevant to your study. If you are not sure if a list item applies to your research, read the appropriate section before selecting a response.

Materials & experimental systems

n/a	Involved in the study
☒	☐ Antibodies
☒	☐ Eukaryotic cell lines
☒	☐ Palaeontology and archaeology
☒	☐ Animals and other organisms
☐	☒ Human research participants
☐	☒ Clinical data
☒	☐ Dual use research of concern

Methods

n/a	Involved in the study
☒	☐ ChIP-seq
☐	☒ Flow cytometry
☒	☐ MRI-based neuroimaging

Human research participants

Policy information about studies involving human research participants

Population characteristics

Women with pure DCIS who developed a subsequent invasive or DCIS recurrence

Recruitment

The research participants were recruited through three studies:

The Sloane project, United Kingdom National Health Service Breast Screening Programme, was approved by the UK Health Research Authority (Ethical approval REF 08/S0703/147, 19/LO/0648)

The Dutch DCIS cohort study, a Netherlands Cancer Registry (NCR; ref. no 12.281), nationwide network and registry of histology and cytopathology in the Netherlands (PALGA; ref. no. LZV990) approved by the Central Committee on Research Involving Human Subjects in the Netherlands and the Institutional Review Board of the Netherlands Cancer Institute (CFMPB166, CFMPB393 and CFMPB688).

The Duke Hospital cohort approved by Duke University Health System Institutional Review Board, USA (Pro00054877, Pro00068646).

There was no participant compensation.

Ethics oversight

UK Health Research Authority
The Central Committee on Research Involving Human Subjects of the Netherlands
Institutional Review Board of the Netherlands Cancer Institute
Duke University Health System Institutional Review Board, USA

Note that full information on the approval of the study protocol must also be provided in the manuscript.

Clinical data

Policy information about clinical studies
All manuscripts should comply with the ICMJE guidelines for publication of clinical research and a completed CONSORT checklist must be included with all submissions.

Clinical trial registration

The Sloane project, United Kingdom National Health Service Breast Screening Programme (REF 08/S0703/147, 19/LO/0648)

The Dutch DCIS cohort study, a Netherlands Cancer Registry (NCR; ref. no 12.281), nationwide network and registry of histology and cytopathology in the Netherlands (PALGA; ref. no. LZV990). IRB of the Netherlands Cancer Institute (CFMPB166, CFMPB393 and CFMPB688)

The Duke Hospital cohort (IRB approvals: Pro00054877, Pro00068646)

Study protocol

These datasets are not part of clinical trials. Study protocols are available from the corresponding author.

Data collection

The Sloane project, a national audit of women with non-invasive neoplasia within the United Kingdom National Health Service Breast Screening Programme. Data collected between 2003 and 2012.

The Dutch DCIS cohort study, a nation-wide, population-based patient cohort derived from the Netherlands Cancer Registry (NCR), in which all women diagnosed with primary DICS between 1989 and 2004 were included.

The Duke Hospital cohort, a hospital-based study of women (age 40-75 years) diagnosed with DCIS between 1998 and 2016.

Outcomes

Outcome data of our clinical cohorts consisted of any ipsilateral recurrence, i.e. an in situ or an invasive recurrence; at least 5 months after the diagnosis of a primary DCIS lesion.

March 2021

Flow Cytometry

Plots

Confirm that:

- ☐ The axis labels state the marker and fluorochrome used (e.g. CD4-FITC).
- ☐ The axis scales are clearly visible. Include numbers along axes only for bottom left plot of group (a 'group' is an analysis of identical markers).
- ☐ All plots are contour plots with outliers or pseudocolor plots.
- ☐ A numerical value for number of cells or percentage (with statistics) is provided.

Methodology

Sample preparation	FFPE Tissue Dissociation Kit from MACS (Cat#130-118-052)
Instrument	BD FACSMelody
Software	BD FACSChorus software
Cell population abundance	DAPI counts vs DAPI area plots were used to determine relative population abundance
Gating strategy	DAPI (area) fluorescence intensities were used to determine which nuclei populations were flow-sorted. We gate from 2N peaks and from > 2N peaks to enrich tumor cells based on DAPI fluorescent signal.

- ☒ Tick this box to confirm that a figure exemplifying the gating strategy is provided in the Supplementary Information.
